# Stereoselective, borane-catalysed synthesis of *syn*-β-hydroxyketones from α,β-unsaturated ketones

**DOI:** 10.1039/d6sc03052a

**Published:** 2026-05-12

**Authors:** Julie Macleod, Alastair J. Nimmo, Joseph H. P. Cockcroft, Paula Dominguez-Molano, Gary S. Nichol, Stephen P. Thomas

**Affiliations:** a EaStCHEM School of Chemistry, Joseph Black Building, The University of Edinburgh David Brewster Road Edinburgh EH9 3FJ UK stephen.thomas@ed.ac.uk

## Abstract

The reductive aldol reaction is a powerful tool for the regiocontrolled coupling of α,β-unsaturated compounds with aldehydes. The use of stoichiometric organoborane reductants has previously been reported. Here these reagents have been rendered catalytic through B–O transborylation (B–O/B–H metathesis). This one-pot dialkylborane-catalysed method allows for the synthesis of β-hydroxycarbonyl compounds in good yields with high diastereo- and enantioselectivity. This protocol was applied across a broad substrate scope including those containing reducible functional groups and intramolecular coupled examples.

## Abstract 2

Boron catalysis and transborylation have enabled the chemo- and stereoselective coupling of enones and aldehydes to give aldol-type products.

## Graphical abstract

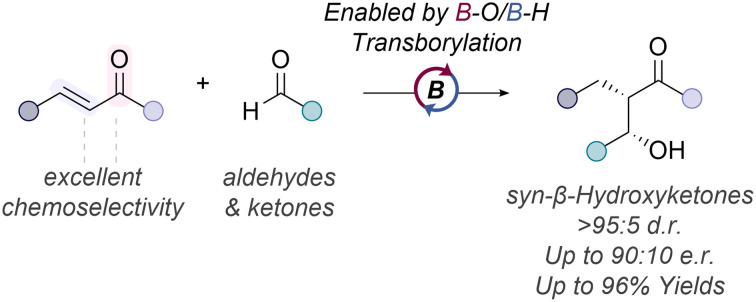

## Introduction

**Fig. 1:**
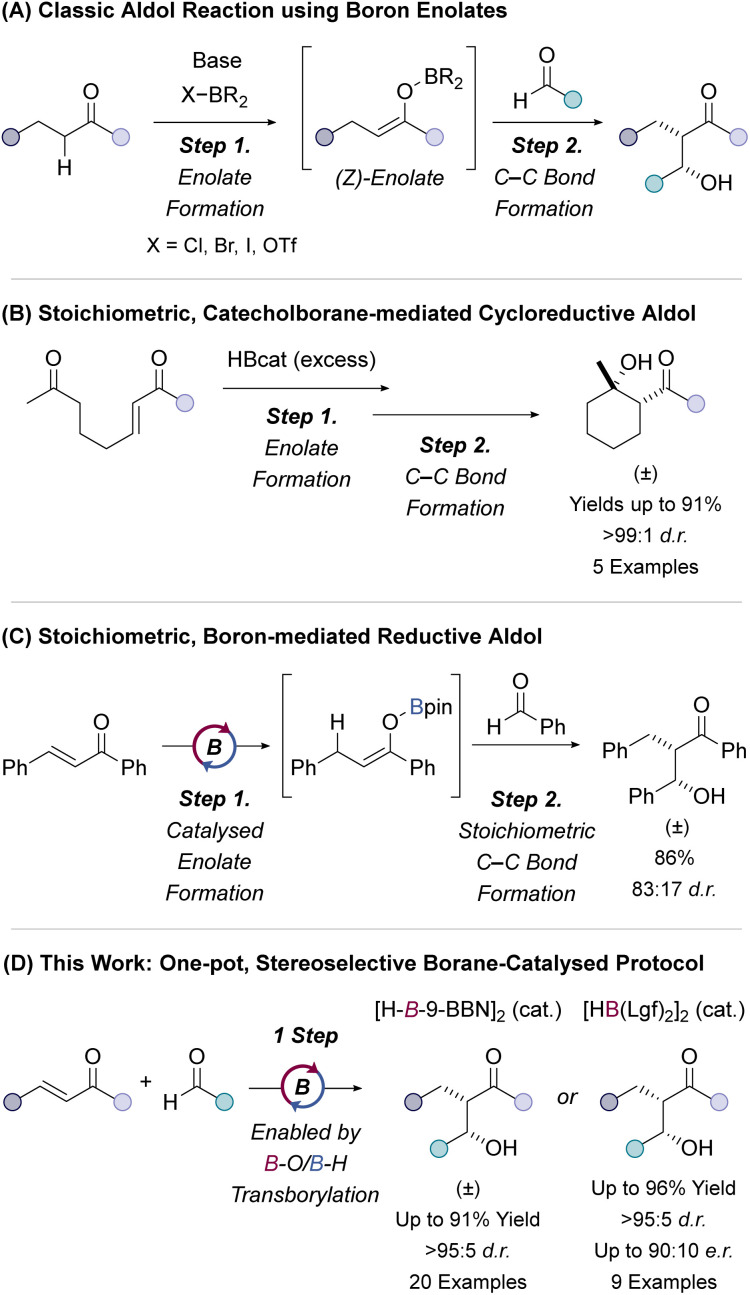
Synthesis of β-hydroxy ketones. (A) Two-step, base-mediated aldol reactions using boron enolate intermediates. (B) Stoichiometric catecholborane-mediated 1,4-hydroboration (formation of boron enolate) followed by C–C bond formation to form cyclic aldol products. (C) Stoichiometric, enantioselective reductive aldol of α,β-unsaturated amides. (D) Two-step reductive aldol reaction by borane-catalysed 1,4-hydroboration and C–C bond formation. (E) This work: one-pot, diastereo- and enantioselective methodologies for the borane-catalysed coupling of α,β-unsaturated ketones with aldehydes.

Discovered over 150 years ago, the aldol reaction is still one of the most fundamental and useful methods for regio- and stereoselective carbon–carbon bond formation.^1–4^ Aldol reactions traditionally involve the coupling of two carbonyl compounds (ketone/aldehydes) to form a β-hydroxycarbonyl product.^5^ The geometry, and maintenance of this geometry, of the intermediate enolate species is key to achieving high stereoselectivity in aldol reactions.^1,5^ The reaction of stereodefined boron-enolates with aldehydes can be highly diastereoselective for *syn*- and *anti*-aldol products,^6,7^ with higher stereoselectivities typically observed when using boron enolates compared to metal enolates.^6–9^ Boron enolates are generated in the vast majority of cases by deprotonation of a carbonyl compound followed by trapping with a suitable dialkylboron reagent bearing a leaving group, X–BR_2_ (X = Cl, Br, I, OTf *etc.*) (Fig. 1A).^6,7,10–14^

The regioselective deprotonation of non-symmetrical (alkyl) ketones is a notable challenge posed in classical aldol reactions.^3^ Reductive aldol-type methodologies for the coupling of α,β-unsaturated carbonyl compounds with aldehydes have been used to mitigate these selectivity issues.^1,3,15,16^ The use of α,β-unsaturated ketones (enones) enables regiocontrolled enolate formation in cases where enolisation (by deprotonation) of the corresponding ketone would give a mixture of enolate regioisomers.^12,17^ Reductive aldol couplings of enones with aldehydes/ketones have been catalysed by rhodium,^17–23^ palladium,^24^ copper,^25^ and indium^26,27^ complexes, Lewis acid catalysts^28–32^ and by photochemical protocols.^33^

Intramolecular reductive aldol reactions are also possible through chemoselective reduction of bifunctional substrates where the reduction of an enone generates a nucleophilic enolate which reacts with the tethered electrophile.^34^ Intramolecular aldol cycloreductions have previously been catalysed by organocatalysts,^30,35^ rhodium complexes,^19,36^ a copper hydride complex^37^ and indium^26^ reagents. A stoichiometric, borane-mediated aldol cycloreduction was reported by Krische using excess catecholborane (HBcat) for the synthesis of six-membered cyclic aldol products in excellent yields (from 80% to 91%) and diastereoselectivity (>99 : 1 d.r.) (Fig. 1B).^38^

Hayashi reported a two-step, stoichiometric coupling of α,β-unsaturated ketones with benzaldehyde, where 1,4-hydroboration using HBcat or 9-borabicyclo[3.3.1]nonane, [H–*B*-9-BBN]_2_ (also referred to as 9-BBN) was followed by electrophilic trapping.^39^ Quantitative yields were achieved using both organoboranes, however lower diastereoselectivity was observed using HBcat (75 : 25 d.r.) compared to [H–*B*-9-BBN]_2_ (>96 : 4 d.r).^39^ Roush used stoichiometric diisopino-campheylborane ((−)-[HB(Ipc)_2_]_2_) for the enantioselective reductive aldol-type reaction of α,β-unsaturated esters and amides (Fig. 1C).^40–42^ Thomas and Nicholson established a borane-catalysed reduction and hydrofunctionalisation of α,β-unsaturated ketones.^31,32^ This work included an example of a [H–*B*-9-BBN]_2_-catalysed 1,4-hydroboration of chalcone and trapping of the boron enolate with benzaldehyde in good yield (86%) but poor diastereoselectivity (83 : 17 d.r.), presumably due to the generation of an O–Bpin enolate (Fig. 1D).^31^ The O–*B*-9-BBN enolate undergoes facile B–O transborylation (B–O/B–H metathesis) with HBpin which enables catalytic turnover but ensures O–Bpin enolate formation prior to reaction with benzaldehyde. Alkyl borane enolates, O–BR_2_, are more reactive and offer higher levels of stereocontrol in aldol reactions with the potential for a catalytic enantioselective method. We therefore sought to develop a catalytic protocol which would access the O–BR_2_ enolate and leverage greater stereoselectivity.^31,39,43^ This is not without challenge however as the use of more reactive alkyl boranes (HBR_2_) increases the possibility of direct (1,2-)reduction of the aldehyde and alkene 1,2-hydroboration of the α,β-unsaturated ketone. The former is further complicated in a one-pot reaction with inclusion of the aldehyde from the start. Aldehyde reduction is usually circumvented by a 2-step enolate formation and aldol reaction sequence.

Herein, we report one-pot, dialkylborane-catalysed ([H–*B*-9-BBN]_2_ or [HB(Lgf)_2_]_2_), diastereoselective and enantioselective protocols for the reductive coupling of α,β-unsaturated ketones with aldehydes (Fig. 1E).

## Results and discussions

Investigations began by screening various borane reagents to determine the optimal borane catalyst for the coupling of a model α,β-unsaturated ketone 1a and aldehyde 2a using pinacolborane (HBpin) as a turnover reagent. Low amounts of product (14%) were observed in the absence of a catalyst (Table 1, Entry 1). The use of Me_2_S·BH_3_ as the catalyst showed no chemoselectivity for 1,4-hydroboration with both of the C=O and C=C bonds of the enone 1a reduced and no product formation observed (Entry 2). Piers' borane, [HB(C_6_F_5_)_2_]_2_, was selective for 1,4-hydroboration, over alkene and carbonyl 1,2-hydroboration, but full conversion from the boron-enolate to the *syn*-β-hydroxy ketone product 3a was not observed (34%, 95 : 5 d.r., Entry 3). Any unreacted boron enolate resulted in the formation of the ketone 4, by 1,4-reduction and protonation of the boron enolate on workup. A slight improvement in yield was observed using disiamylborane–tetrahydrofuran complex, (THF HBSia_2_), but with lower diastereoselectivity (41%, 92 : 8 d.r., Entry 4). An improved yield and excellent diastereoselectivity was observed when dicyclohexylborane, [HBCy_2_]_2_, was used as the catalyst (68%, >95 : 5 d.r., Entry 5). Use of [H–*B*-9-BBN]_2_ resulted in an excellent yield and diastereoselectivity (>95%, >95 : 5 d.r.) of the *syn*-β-hydroxy ketone 3a (Entry 6). For comparison, an excellent yield and diastereoselectivity (>95%, >95 : 5 d.r.) was also observed using stoichiometric [H–*B*-9-BBN]_2_ (50 mol%) in the absence of HBpin (Entry 7). Changing the solvent to toluene, a non-coordinating solvent, also resulted in an excellent yield and diastereoselectivity of the aldol product 3a (89%, >95 : 5 d.r.). Performing the reaction at temperatures below 40 °C resulted in lower yields due to the incomplete reaction of starting materials. A two-step protocol (full conversion to the O–Bpin enolate over 20 hours followed by aldehyde addition) resulted in lower diastereoselectivity (92 : 8 d.r.), agreeing with the hypothesis that greater stereoselectivity is achieved when using O–BR_2_ enolates rather than O–Bpin enolates.

**Table 1:** Optimisation of reaction conditions – borane screen

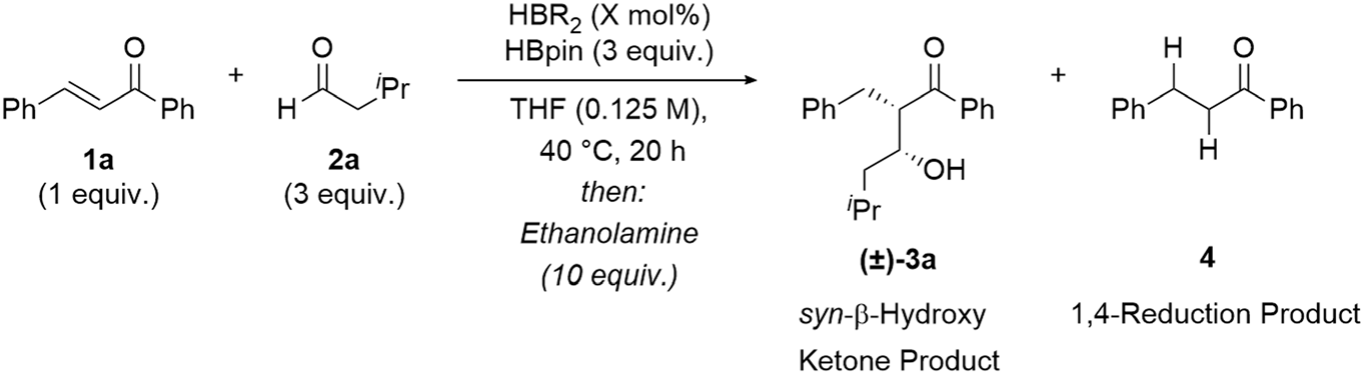						
Entry	HBR_2_	mol%	Conv. (%)	Ratio 3 : 4^a^	Yield 3a (%)^a^	d.r. (*syn* : *anti*)^a^
1	—	—	66	24 : 76	14	—
2	Me_2_S·BH_3_	20	>95	—	—	—
3	[HB(C_6_F_5_)_2_]_2_	5	93	44 : 56	34	95 : 5
4	THF HBSia_2_	10	93	59 : 41	41	92 : 8
5	[HBCy_2_]_2_	5	>95	77 : 23	79	>95 : 5
6	[H–*B*-9-BBN]_2_	4	>95	100 : 0	>95	>95 : 5
7^b^	[H–*B*-9-BBN]_2_	50	>95	100 : 0	>95	>95 : 5

a Determined by ^1^H NMR spectroscopic analysis of the crude reaction mixtures using 1,3,5-trimethoxybenzene as an internal standard.

b No HBpin. All optimisation reactions were carried out on a 0.50 mmol scale. See SI Table S1 for the full details of reaction optimisation.

Optimal catalysis conditions were established using [H–*B*-9-BBN]_2_, (4 mol%) with HBpin, (3 equiv.) in THF (0.125 m), at 40 °C for the coupling of model substrates chalcone 1a (1 equiv.) and isovaleraldehyde 2a (3 equiv.). These conditions resulted in full conversion to the β-hydroxy ketone 3a (>95% yield) with high *syn*-aldol diastereoselectivity (>95 : 5 d.r.), presumably through generation and coupling of the (*Z*)-boron enolate.^9,44,45^

**Fig. 2:**
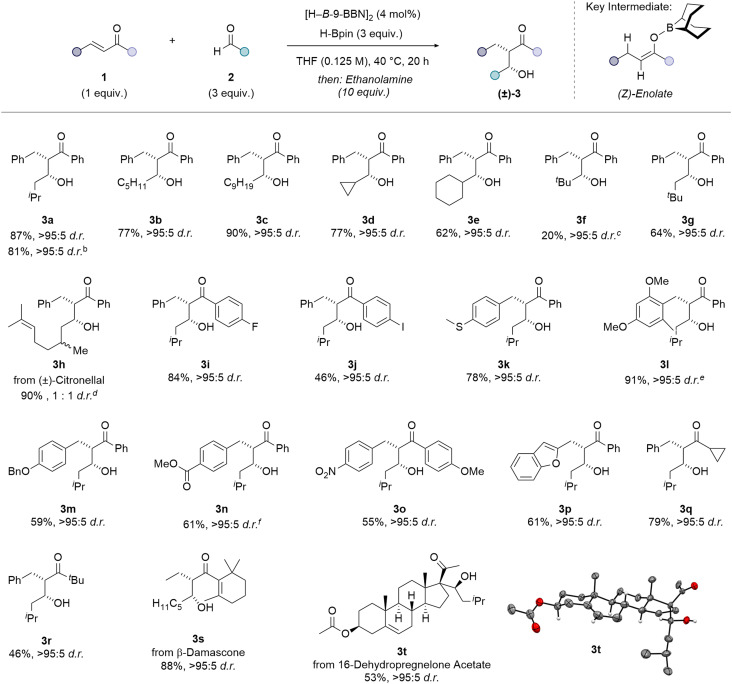
(A) Substrate scope for borane-catalysed enone-aldehyde coupling reaction. Conditions: enone 1 (0.50 mmol, 1 equiv.), aldehyde 2 (3 equiv.), [H–*B*-9-BBN]_2_ (4 mol%), HBpin (3 equiv.), THF (0.125 M), 20 h, 40 °C; then ethanolamine (10 equiv.). Diastereoselectivity was determined by ^1^H NMR spectroscopy of the isolated product. ^*b*^HBEpin (3 equiv.) instead of HBpin. ^*c*^5.5 mmol scale-up reaction. ^*d*^75 : 25 d.r. before purification. ^*e*^From (±)-citronellal. Isolated as an inseparable mixture of two diastereoisomers. >95 : 5 d.r. with respect to *syn* : *anti* of C2 and C3 in the product. ^*f*^90 : 10 d.r. before purification. ^*g*^94 : 6 d.r. before purification. Thermal ellipsoids for crystal structure of 3t shown at 50% probability level, red = oxygen, grey = carbon, white = hydrogen. (B) Proposed mechanism for the dialkylborane-catalysed reductive coupling of α,β-unsaturated ketones with aldehydes.

The scope and limitations of this enone-aldehyde coupling were then investigated (Fig. 2). In all cases ^1^H NMR spectroscopy was used to establish the diastereoselectivity of the reaction before and after purification. Chalcone 1a and isovaleraldehyde 2a were coupled with excellent diastereoselectivity (>95 : 5 d.r.) before purification, and the corresponding *syn*-β-hydroxy ketone 3a isolated as a single diastereoisomer in high yield (87%). Similar yield and diastereoselectivity were observed when 4,4,5,5-tetraethyl-1,3,2-dioxaborolane, HB(Epin), was used as an alternative turnover reagent, giving the aldol product 3a in 81% isolated yield and >95 : 5 d.r. A scale-up reaction (5.5 mmol) resulted in 70% isolated yield and >95 : 5 d.r. of aldol product 3a. The reaction of chalcone 1a with straight-chain, alkyl aldehydes, hexanal and decanal, gave high yields and excellent diastereoselectivity for the hexyl *syn*-aldol product 3b (77%, >95 : 5 d.r.) and decyl *syn*-aldol product 3c (90%, >95 : 5 d.r.). Good yields and excellent diastereoselectivity were observed using cyclopropane-carboxaldehyde 3d (77%, >95 : 5 d.r.) and cyclohexane-carboxaldehyde 3e (62%, >95 : 5 d.r). Pivaldehyde resulted in lower yield and poorer crude diastereoselectivity (75 : 25 d.r), nevertheless 3f was isolated as a single diastereoisomer (20%, >95 : 5 d.r.). Large amounts of enone 1,4-reduction product 4 was generated presumably due to the increased steric bulk of pivaldehyde slowing aldol coupling and leaving significant amount of unreacted boron-enolate on work-up. Using 3,3-dimethylbutanal, with a β-^*t*^Bu group, however resulted in an improved yield of the *syn*-aldol product 3g (64%, >95 : 5 d.r.). The enone-aldehyde coupling protocol was applied to (±)-citronellal, a monoterpene commonly used as an intermediate in the synthesis of several natural terpenoids.^46^ The corresponding *syn*-β-hydroxy ketone 3h was isolated in excellent yield and with excellent control of diastereoselectivity with respect to the *syn-versus anti*-aldol product (90%, >95 : 5 d.r.), but as a 1 : 1 epimeric mixture at the methyl group due to the use of the racemic aldehyde. Minimal product formation (<10%) was observed when aromatic aldehydes, such as benzaldehydes and heteroaromatic aldehydes, were used in this reaction. Substrates containing aryl halide substituents are often challenging when using transition-metal catalysts due to unwanted oxidative addition and protodehalogenation.^47^ The developed borane-catalysed reaction was successfully applied to chalcone derivatives bearing aryl halide substituents, such as 4′-fluorochalcone 3i (84%, >95 : 5 d.r.) and 4′-iodochalcone 3j (46%, >95 : 5 d.r.). The Lewis acidic catalyst, [H–*B*-9-BBN]_2_, was found to be tolerant of substrates bearing Lewis basic and electron-donating functionalities such as thioether 3k (78%, >95 : 5 d.r.), methoxy 3l (91%, >95 : 5 d.r.) and benzyloxy 3m (59%, >95 : 5 d.r.) groups. The reaction also tolerated the presence of reducible functional groups including ester- 3n (61%, >95 : 5 d.r.) and nitro-substituted substrates 3o (55%, >95 : 5 d.r.). The presence of benzofuran, as an alternative arene substituent, resulted in a moderate yield and high diastereoselectivity of 3p (61%, >95 : 5 d.r.). This reaction was also successful when one phenyl group of chalcone 1a was replaced with an alkyl substituent. A high yield and excellent diastereoselectivity of cyclopropyl substituted *syn*-β-hydroxy ketone 3q (79%, >95 : 5 d.r.) was observed. Increasing the steric bulk to a *tert*-butyl group resulted in a lower yield of the corresponding product 3r, but the excellent control of diastereoselectivity was maintained (46%, >95 : 5 d.r.). β-Damascone, which contributes to the fruity, floral aroma of rose oil and is widely used by the fragrance industry for the preparation of cosmetics and perfumes,^48–50^ was coupled with hexanal to give the corresponding *syn*-β-hydroxy ketone 3s in high yield and diastereoselectivity (88%, >95 : 5 d.r.). 16-Dehydropregnenolone acetate (16-DPA), a versatile building block and precursor for the production of various steroid drugs and hormones,^51,52^ was also successfully coupled with isovaleraldehyde 2a. Aldol product 3t was isolated in moderate yield (53%), but without reduction of the ester or alkene groups of the enone, and as a single diastereoisomer. The absolute stereochemical assignment of aldol product 3t was confirmed by single crystal X-ray analysis (Fig. 2). Minimal product formation (<10%) was observed when a β,β-disubstituted unsaturated ketone (1,3-diphenyl-2-buten-1-one) was used in this reaction. Minimal product formation (<10%) was observed with cyclohexenone, presumably due to the inability to orientate into the s-cis conformation required for 1,4-hydroboration.

A mechanism for the dialkylborane-catalysed reductive coupling of enones 1 with aldehydes 2 was proposed (Fig. 2B). 1,4-Hydroboration of the enone 1 with the [H–*B*-9-BBN]_2_ catalyst generates the O–*B*-9-BBN (*Z*)-enolate which undergoes C–C bond formation with the aldehyde 2. HBpin regenerates the [H–*B*-9-BBN]_2_ catalyst through B–O/B–H transborylation. Lastly, ethanolamine hydrolyses the O–Bpin bond to give the alcohol product 3a.

**Fig. 3:**
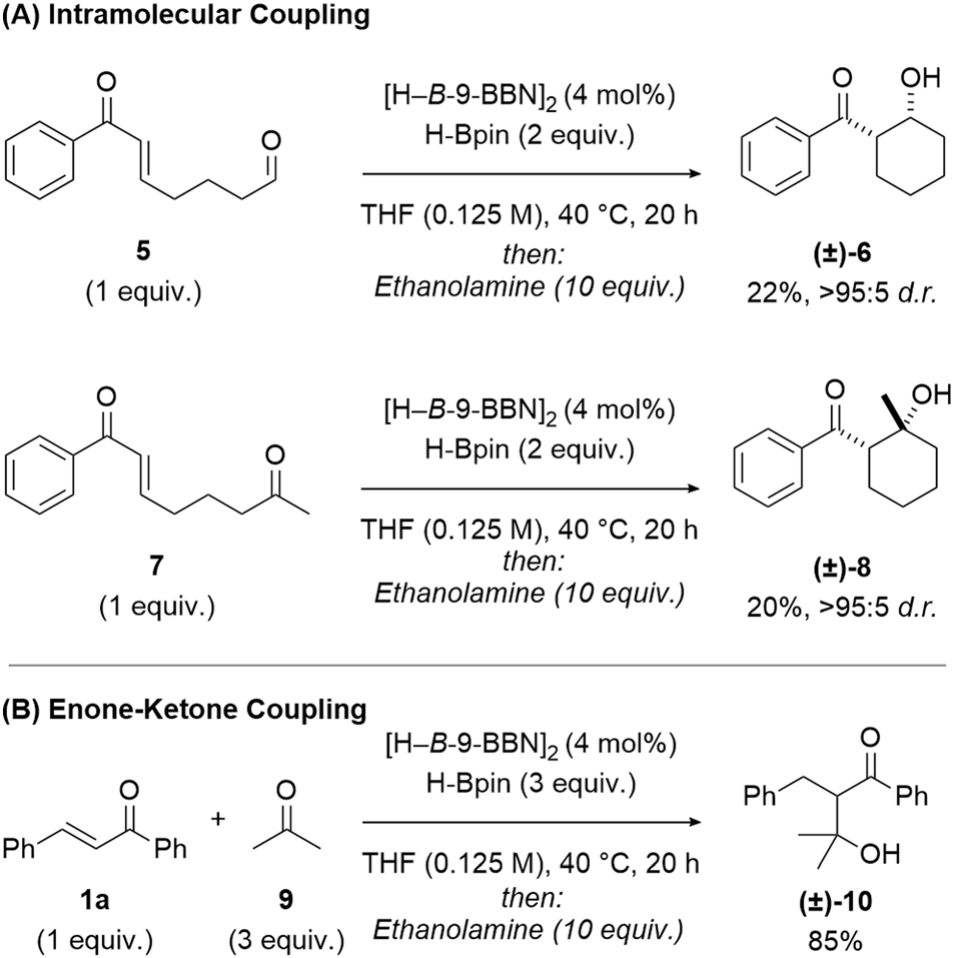
Additional reactivity. (A) Borane-catalysed intramolecular aldol coupling. Reaction conditions: substrate 5 or 7 (0.50 mmol, 1 equiv.), [H–*B*-9-BBN]_2_ (4 mol%), HBpin (2 equiv.), THF (0.125 M), 20 h, 40 °C; then ethanolamine (10 equiv.). Diastereoselectivity was determined by ^1^H NMR spectroscopy of both the crude reactions and isolated products – in all cases, the diastereoselectivity of the crude reaction was >95 : 5 d.r. (B) Borane-catalysed enone-ketone coupling of chalcone 1a and acetone 9. Reaction conditions: chalcone 1a (0.5 mmol, 1 equiv.), acetone 9 (3 equiv.), [H–*B*-9-BBN]_2_ (4 mol%), HBpin (3 equiv.), THF (0.125 M), 20 h, 40 °C; then ethanolamine (10 equiv.).

Intramolecular coupling of (*E*)-7-oxo-7-phenylhept-5-enal 5 and (2*E*)-1-phenyl-2-octene-1,7-dione 7 were investigated using the borane-catalysed protocol (Fig. 3A). Ring-closed *syn*-β-hydroxy ketones 6 (22%, >95 : 5 d.r.) and 8 (20%, >95 : 5 d.r.) were successfully synthesised with high diastereoselectivity, but in low yield, with competitive reduction of the aldehyde/ketone groups accounting for the reduced yield. To expand the synthetic application of this protocol, acetone 9 was successfully used as an alternative coupling partner (Fig. 3B) to give the β-hydroxy ketone 10 in good yield (85%), from coupling with chalcone 1a.

Trombini reported a two-step enantioselective coupling of enones and aldehydes mediated by stoichiometric diisopinocampheylborane [(−)-[HB(Ipc)_2_]_2_].^9,44,45^ Eight substrates were isolated in varying yields (30–90%) with moderate to high enantioselectivity (75 : 25 e.r.–95 : 5 e.r.).^9^ A high yield (83%) but poor enantiomeric excess (31%) was observed when dicaranylborane ([4-(Icr)_2_BH]_2_) was tested as an alternative borane reagent.^9^ Roush used stoichiometric [(−)-HB(Ipc)_2_]_2_ for the enantioselective aldol-type coupling reactions of α,β-unsaturated esters and amides.^40–42^ To render our catalytic method enantioselective various enantioenriched boranes were trialled as catalysts, including: dilongifolylborane ((+)-[HB(Lgf)_2_]_2_) synthesised from the parent terpene, longifolene; dicaranylborane ([4-(Icr)_2_BH]_2_), from 3-carene; ((−)-[HB(Ipc)_2_]_2_); and methoxy Soderquist borane ((*R*)-Ph-BBD-OMe) (see SI Table S4 for the full optimisation). Moderate yields and poor diastereo- and enantioselectivity were observed using (−)-[HB(Ipc)_2_]_2_ (57%, 90 : 10 d.r., 59 : 41 e.r.) and [4-(Icr)_2_BH]_2_ (56%, 85 : 15 d.r., 56 : 44 e.r.) as the catalysts (Table 2, Entries 1 and 2), reactions with the former were in contrast with those previously reported by Roush using α,β-unsaturated esters and amides.^40–42^ (*R*)-Ph-BBD-OMe resulted in a good yield and excellent diastereoselectivity (77%, >95 : 5 d.r.) however no control of enantioselectivity was observed (48 : 52 e.r.) (Entry 3). (+)-[HB(Lgf)_2_]_2_ gave a moderate yield (48%) and synthetically useful diastereo- and enantioselectivity (>95 : 5 d.r., 82 : 18 e.r.) (Entry 4). (+)-[HB(Lgf)_2_]_2_ was therefore taken forward for further reaction optimisation. Lowering the reaction temperature from 40 °C to room temperature (Entry 5) improved the yield and stereoselectivity of (−)-*syn*-β-hydroxy ketone 3a (67%, >95 : 5 d.r., 86 : 14 e.r.). Reduced enantioselectivity was observed when the solvent was changed to non-coordinating solvents such as toluene (88%, >95 : 5 d.r., 83 : 17 e.r.) (Entry 6) and hexane (64%, >95 : 5 d.r., 77 : 23 e.r.) (Entry 7). Changing the solvent from THF to methyl *tert*-butyl ether (MTBE) resulted in the highest yield and diastereo- and enantioselectivity of (−)-*syn*-β-hydroxy ketone 3a (95%, >95 : 5 d.r., 90 : 10 e.r.) (Entry 8). Competing racemic product formation, referred to as a ‘background’ reaction by an achiral borane, may erode the enantioselectivity of the targeted catalytic reaction. This can be quantified using enantiofidelity (e.f.), defined as the degree of enantioselectivity retained in the substoichiometric reaction in comparison to the stoichiometric reaction.^53^ The substoichiometric conditions (90 : 10 e.r.) achieved very similar enantioselectivity compared to the stoichiometric conditions (92 : 8 e.r.) (Entry 9) resulting in an enantiofidelity of 95%, and so negligible background (non-stereoselective) reactivity during catalysis. The absolute configuration of enantioenriched aldol product (2*S*,3*R*)-3a was confirmed by single crystal X-ray analysis, with the configuration within all other products assigned by analogy.

**Table 2:** Optimisation of the enantioselective borane-catalysed enone-aldehyde coupling^a^

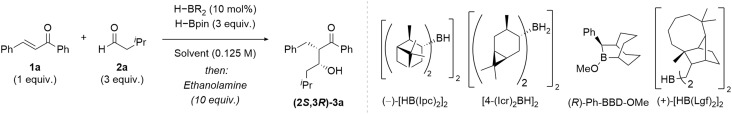							
Entry	HBR_2_	Temperature (°C)	Time (h)	Solvent	Yield (%)	d.r. (*syn* : anti)	e.r.
1	(-)-[HB(Ipc)_2_]_2_	40	24	THF	67 (57)	90 : 10	59 : 41
2	[4-(Icr)_2_BH]_2_	40	24	THF	67 (56)	85 : 15	56 : 44
3^b^	(*R*)-Ph-BBD-OMe	40	24	THF	92 (77)	>95 : 5	48 : 52
4	(+)-[HB(Lgf)_2_]_2_	40	24	THF	62 (48)	>95 : 5	82 : 18
5	(+)-[HB(Lgf)_2_]_2_	rt	72	THF	79 (67)	>95 : 5	86 : 14
6	(+)-[HB(Lgf)_2_]_2_	rt	72	Toluene	>95 (88)	>95 : 5	83 : 17
7	(+)-[HB(Lgf)_2_]_2_	rt	72	Hexane	70 (64)	>95 : 5	77 : 23
8	(+)-[HB(Lgf)_2_]_2_	rt	72	MTBE	>95 (95)	>95 : 5	90 : 10
9^c^	(+)-[HB(Lgf)_2_]_2_	rt	72	MTBE	84 (78)	>95 : 5	92 : 8

a Diastereoselectivity was determined by ^1^H NMR spectroscopy of the crude reaction mixture. Enantioselectivity was determined by HPLC analysis on a chiral stationary phase. The stereochemical assignment of the major enantiomer was confirmed by single crystal X-ray analysis.

b 5 equiv. of HBpin.

c Stoichiometric (+)-[HB(Lgf)_2_]_2_ used (1 equiv.), no HBpin.

**Fig. 4:**
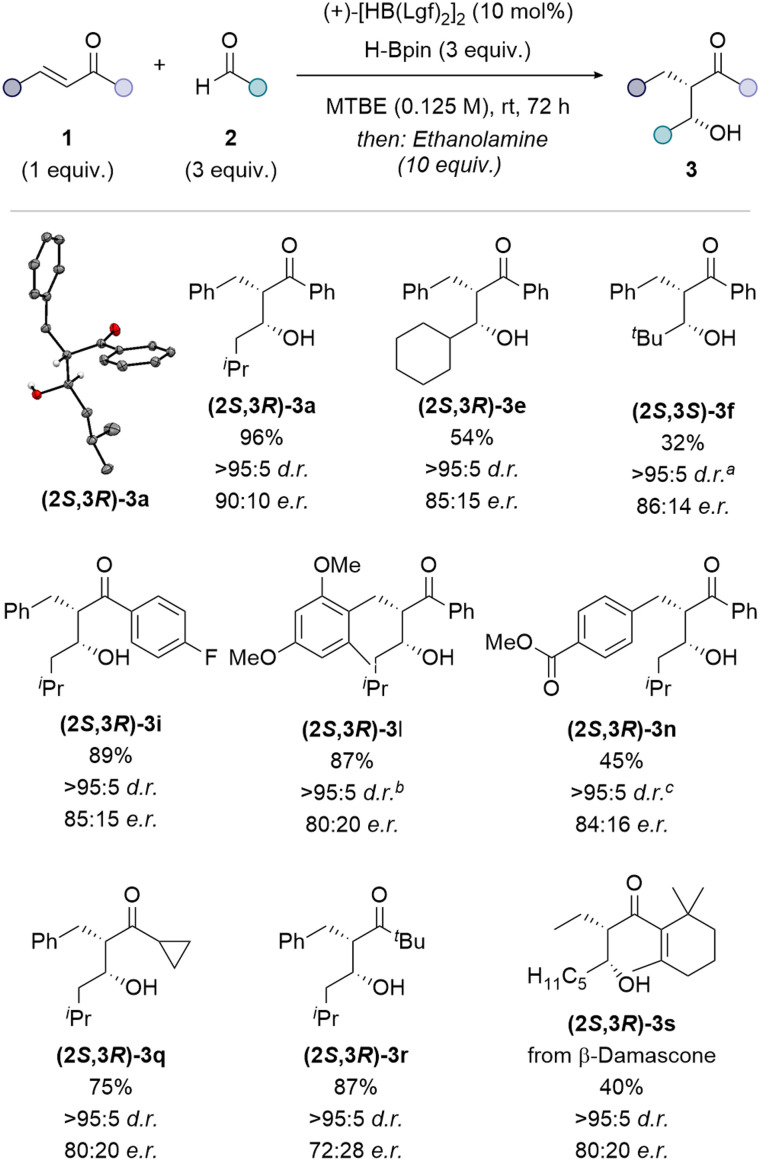
Substrate scope for the enantioselective, borane-catalysed enone-aldehyde coupling. Enone 1 (0.50 mmol, 1 equiv.), aldehyde 2 (3 equiv.), (+)-[HB(Lgf)_2_]_2_ (10 mol%), HBpin (3 equiv.), MTBE (0.125 M), 72 h, room temperature; then ethanolamine (10 equiv.). Diastereoselectivity was determined by ^1^H NMR spectroscopy of the isolated product. Enantioselectivity was determined by HPLC analysis of the isolated product on a chiral stationary phase. ^*a*^74 : 26 d.r. before purification. ^*b*^93 : 7 d.r. before purification. *^c^*93 : 7 d.r. before purification. Thermal ellipsoids for crystal structure of (2*S*,3*R*)-3a shown at 50% probability level, red = oxygen, grey = carbon, white = hydrogen.

The optimised reactions conditions were applied to representative substrates, covering a variety of functional groups, previously tested under the non-enantioselective reaction conditions. High diastereoselectivity was maintained but a reduction in yield and a slightly lower e.r. was observed when secondary and tertiary aldehydes were used including for the cyclohexyl aldehyde derived product (2*S*,3*R*)-3e (54%, >95 : 5 d.r., 85 : 15 e.r.) and *tert*-butyl aldehyde derived product (2*S*,3*S*)-3f (32%, >95 : 5 d.r., 86 : 14 e.r), respectively (Fig. 4). The presence of 4-fluoro substitution on the arene was tolerated, with an excellent yield and high diastereo- and enantioselectivity observed for aldol product (2*S*,3*R*)-3i (89%, >95 : 5 d.r., 85 : 15 e.r.). Lewis basic ether substituents on the aryl group resulted in a slightly lower enantioselectivity but excellent yield and diastereoselectivity for the methoxy substituted aldol product (2*S*,3*R*)-3l (87%, >95 : 5 d.r., 80 : 20 e.r.). The reaction tolerated the presence of a reducible ester group (2*S*,3*R*)-3n (45%, >95 : 5 d.r., 84 : 16 e.r.). Good yields but decreased enantioselectivity were observed when the aryl substituent adjacent to the ketone was changed to a cyclopropyl group (2*S*,3*R*)-3q (75%, >95 : 5 d.r., 80 : 20 e.r.) or a *tert*-butyl group (2*S*,3*R*)-3r (87%, >95 : 5 d.r., 72 : 28 e.r). A moderate yield and good stereoselectivity was observed when aliphatic enone β-damascone was coupled with hexanal to give the aldol product (2*S*,3*R*)-3s (40%, >95 : 5 d.r., 80 : 20 e.r.).

## Conclusions

In summary, we have demonstrated the application of B–O/B–H transborylation as a turnover strategy for the stereoselective dialkylborane-catalysed ([H–*B*-9-BBN]_2_ or (+)-[HB(Lgf)_2_]_2_) protocols for the reductive coupling of α,β-unsaturated ketones with aldehydes to give *syn*-aldol products. This has enabled previously stoichiometric organoborane reductants to be used as catalysts and provided a main-group alternative to the well-established transition-metal catalysed protocols. The one-pot [H–*B*-9-BBN]_2_-catalysed coupling of α,β-unsaturated ketones with aldehydes allowed for the synthesis of *syn*-β-hydroxycarbonyl products in excellent yields (up to 91%) and diastereoselectivity (>95 : 5 d.r.). This catalytic method was rendered asymmetric by using (+)-[HB(Lgf)_2_]_2_ as an enantioenriched dialkylborane catalyst which gave moderate to excellent yields (up to 96%) of the *syn*-β-hydroxycarbonyl products with excellent diastereo-selectivity and high enantioselectivity (up to >95 : 5 d.r., 90 : 10 e.r.). The catalytic methods were chemoselective for 1,4-hydroboration over 1,2-hydroboration (C=O and C=C) and reducible functional groups were tolerated.

## Author contributions

JM, AJN and SPT conceived and developed the method. JM and PD carried out all experimental work. JHPC and GSN conducted the X-ray crystallographic analysis. JM, AJN and SPT prepared the paper.

## Conflicts of interest

There are no conflicts to declare.

## Supplementary Material

SC-017-D6SC03052A-s001

SC-017-D6SC03052A-s002

## Data Availability

CCDC 2532648 3t and 2532647 (2*S*,3*R*)-3a contain the supplementary crystallographic data for this paper.^54*a*,*b*^ All experimental details, characterisation data, and optimisation are provided in the supplementary information (SI). All experimental and analytical data (processed and unprocessed) are openly available through Edinburgh Datastore. Supplementary information: synthesis details, experimental procedures and characterisation data for compounds including NMR spectra (PDF). See DOI: https://doi.org/10.1039/d6sc03052a.

## References

[cit1] Dias L. C., Lucca Jr E. C. d., Ferreira M. A. B., Polo E. C. (2012). J. Braz. Chem. Soc..

[cit2] Harada T. and Abiko A., in Comprehensive Organic Synthesis, ed. P. Knochel, Elsevier, Amsterdam, 2nd edn, 2014, pp. 451–511

[cit3] Garner S. A. , Han S. B. and Krische M. J., in Modern Reduction Methods, 2008, pp. 387–417

[cit4] Mase N. and Hayashi Y., in Comprehensive Organic Synthesis, ed. P. Knochel, Elsevier, Amsterdam, 2nd edn, 2014, pp. 273–339

[cit5] Braun M. , in Modern Aldol Reactions, 2004, pp. 1–61

[cit6] Mukaiyama T. and Matsuo J.-I., in Modern Aldol Reactions, 2004, pp. 127–160

[cit7] Mahrwald R. , Aldol reactions, Springer, Dordrecht, 2009

[cit8] Mekelburger H. B. and Wilcox C. S., in Comprehensive Organic Synthesis, ed. P. Knochel, Elsevier, Amsterdam, 2nd edn, 2014, pp. 243–272

[cit9] Boldrini G. P., Bortolotti M., Mancini F., Tagliavini E., Trombini C., Umani-Ronchi A. (1991). J. Org. Chem..

[cit10] Evans D. A., Nelson J. V., Vogel E., Taber T. R. (1981). J. Am. Chem. Soc..

[cit11] Brown H. C., Dhar R. K., Bakshi R. K., Pandiarajan P. K., Singaram B. (1989). J. Am. Chem. Soc..

[cit12] Gennari C. , Ceccarelli S. and Piarulli U., in Science of Synthesis, Georg Thieme Verlag KG, Stuttgart, 2005, vol. 6

[cit13] Brown H. C., Ganesan K., Dhar R. K. (1993). J. Org. Chem..

[cit14] Ganesan K., Brown H. C. (1993). J. Org. Chem..

[cit15] Han S. B., Hassan A., Krische M. J. (2008). Synthesis.

[cit16] Meyer C. C., Ortiz E., Krische M. J. (2020). Chem. Rev..

[cit17] Bee C., Han S. B., Hassan A., Iida H., Krische M. J. (2008). J. Am. Chem. Soc..

[cit18] Han S. B., Krische M. J. (2006). Org. Lett..

[cit19] Jang H.-Y., Huddleston R. R., Krische M. J. (2002). J. Am. Chem. Soc..

[cit20] Jung C.-K., Garner S. A., Krische M. J. (2006). Org. Lett..

[cit21] Jung C.-K., Krische M. J. (2006). J. Am. Chem. Soc..

[cit22] Marriner G. A., Garner S. A., Jang H.-Y., Krische M. J. (2004). J. Org. Chem..

[cit23] Matsuda I., Takahashi K., Sato S. (1990). Tetrahedron Lett..

[cit24] Kiyooka S.-i., Shimizu A., Torii S. (1998). Tetrahedron Lett..

[cit25] Lipshutz B. H., Papa P. (2002). Angew. Chem., Int. Ed..

[cit26] Miura K., Yamada Y., Tomita M., Hosomi A. (2004). Synlett.

[cit27] Shibata I., Kato H., Ishida T., Yasuda M., Baba A. (2004). Angew. Chem., Int. Ed..

[cit28] Osakama K., Sugiura M., Nakajima M., Kotani S. (2012). Tetrahedron Lett..

[cit29] Sugiura M., Sato N., Kotani S., Nakajima M. (2008). Chem. Commun..

[cit30] Sugiura M., Sato N., Sonoda Y., Kotani S., Nakajima M. (2010). Chem.–Asian J..

[cit31] Nicholson K., Langer T., Thomas S. P. (2021). Org. Lett..

[cit32] Benn K., Nicholson K., Langer T., Thomas S. P. (2021). Chem. Commun..

[cit33] Zhao G., Yang C., Guo L., Sun H., Lin R., Xia W. (2012). J. Org. Chem..

[cit34] Suwa T., Nishino K., Miyatake M., Shibata I., Baba A. (2000). Tetrahedron Lett..

[cit35] Ghobril C., Sabot C., Mioskowski C., Baati R. (2008). Eur. J. Org Chem..

[cit36] Huddleston R. R., Krische M. J. (2003). Org. Lett..

[cit37] Lipshutz B. H., Amorelli B., Unger J. B. (2008). J. Am. Chem. Soc..

[cit38] Huddleston R. R., Cauble D. F., Krische M. J. (2003). J. Org. Chem..

[cit39] Matsumoto Y., Hayashi T. (1991). Synlett.

[cit40] Allais C., Nuhant P., Roush W. R. (2013). Org. Lett..

[cit41] Nuhant P., Allais C., Roush W. R. (2013). Angew. Chem., Int. Ed..

[cit42] Allais C., Tsai A. S., Nuhant P., Roush W. R. (2013). Angew. Chem., Int. Ed..

[cit43] Moreno González A., Nicholson K., Llopis N., Nichol G. S., Langer T., Baeza A., Thomas S. P. (2022). Angew. Chem., Int. Ed..

[cit44] Boldrini G. P., Mancini F., Tagliavini E., Trombini C., Umani-Ronchi A. (1990). J. Chem. Soc., Chem. Commun..

[cit45] Boldrini G. P., Bortolotti M., Tagliavini E., Trombini C., Umani-Ronchi A. (1991). Tetrahedron Lett..

[cit46] Lenardão E. J., Botteselle G. V., de Azambuja F., Perin G., Jacob R. G. (2007). Tetrahedron.

[cit47] Alonso F., Beletskaya I. P., Yus M. (2002). Chem. Rev..

[cit48] Belsito D., Bickers D., Bruze M., Calow P., Greim H., Hanifin J. M., Rogers A. E., Saurat J. H., Sipes I. G., Tagami H. (2007). Food Chem. Toxicol..

[cit49] Lapczynski A., Lalko J., McGinty D., Bhatia S., Letizia C. S., Api A. M. (2007). Food Chem. Toxicol..

[cit50] Mannschreck A., von Angerer E. (2011). J. Chem. Educ..

[cit51] Kumar M., Rawat P., Khan M. F., Rawat A. K., Srivastava A. K., Maurya R. (2011). Bioorg. Med. Chem. Lett..

[cit52] Mancino V., Cerra B., Piccinno A., Gioiello A. (2018). Org. Process Res. Dev..

[cit53] Nicholson K., Dunne J., DaBell P., Garcia A. B., Bage A. D., Docherty J. H., Hunt T. A., Langer T., Thomas S. P. (2021). ACS Catal..

[cit54] (a) CCDC 2532648: Experimental Crystal Structure Determination, 2026, 10.5517/ccdc.csd.cc2r0fbk

