# Supplementary material for: Stereoselective, borane-catalysed synthesis of syn-β-hydroxyketones from α,β-unsaturated ketones
Source: Chem Sci. 2026 May 12;17(26):12917–24. doi: 10.1039/d6sc03052a (PMC13191370; doi:10.1039/d6sc03052a)
Supplement: SC-017-D6SC03052A-s001 [file SC-017-D6SC03052A-s001.pdf]

Supplementary Information (SI) for Chemical Science.
This journal is © The Royal Society of Chemistry 2026

Stereoselective, Borane-catalysed Synthesis of *syn*-β-Hydroxyketones from α,β-Unsaturated Ketones

Julie Macleod, Alastair J. Nimmo, Joseph H. P. Cockcroft, Paula Dominguez-Molano, Gary S. Nichol, and Stephen P. Thomas*

Supporting Information

1. General Experimental S2
2. General Procedures S4
3. Reaction Optimisations S6
4. Preparation of α,β-Unsaturated Ketones S13
5. Preparation of Boron Reagents S16
6. Racemic Substrate Scope S18
7. Unsuccessful Substrates S41
8. Enantioenriched Substrate Scope S42
9. Proposed Mechanism S51
10. References S52
Appendix I: ^{1}H, $^{19}F\{^{1}H\}$ and $^{13}C\{^{1}H\}$ NMR Spectra S54
Appendix II: HPLC Traces of Novel Compounds S135
Appendix III: Single Crystal X-ray Diffraction Data S145

1. General Experimental

Reaction Setup: All reactions were performed in oven (180 °C) dried glassware under an atmosphere of anhydrous nitrogen or argon, unless otherwise indicated. All air- and moisture sensitive reactions were carried out using standard vacuum line and Schlenk techniques, or in a glovebox with a purified argon atmosphere. Glassware was cleaned using base (KOH, ^{i}PrOH) and acid (HNO_3(aq)) baths. All reported reaction temperatures correspond to external heating block temperatures. Room temperature was approximately 16 - 20 °C. “Brine” refers to a saturated solution of sodium chloride in H_2O.

NMR Spectroscopy: ^{1}H, ^{11}B, ^{13}C{^{1}H}, and ^{19}F{^{1}H} NMR spectra were recorded on Bruker Avance III 400 and 500 MHz; Bruker AVI 400 MHz; Bruker Avance I 600 MHz spectrometers. Chemical shifts are reported in parts per million (ppm). ^{1}H NMR spectra were referenced to the residual proteo solvent peak ($CHCl_3$: 7.26 ppm; C_6H_6: 7.16 ppm). ^{13}C NMR spectra were referenced to the deuterated solvent peak ($CDCl_3$: 77.00 ppm; C_6D_6: 128.06). ^{11}B NMR spectra were referenced to $Et_2O{\cdot}BF_3$ and a background suppression function was applied. Multiplicities are indicated by br. (broad), s (singlet), d (doublet), t (triplet), q (quartet), quin. (quintet), sext. (sextet) and app. (apparent). Coupling constants, *J*, are reported in Hertz and rounded to the nearest 0.1 Hz. MestReNova processing software was used to analyse all NMR spectra.

Infrared Spectroscopy: Infrared (IR) spectra were recorded on a Shimadzu IR-Spirit spectrometer. Relevant peaks are reported in cm^{-1}.

Mass Spectrometry: Mass spectrometry (MS) was performed by the University of Edinburgh, School of Chemistry, Mass Spectrometry Laboratory. High-resolution mass spectra were recorded on a VG autospec, or Thermo/Finnigan MAT 900, mass spectrometer. Electrospray Ionization (ESI^+) spectra were performed using a time-of-flight (TOF) mass analyser. Data are reported in the form of m/z.

Chromatography: Analytical thin-layer chromatography was performed on aluminium-backed silica plates (Merck 60 F_{254}). Product spots were visualised by UV light at 254 nm. Flash column chromatography was carried out on a Teledyne ISCO CombiFlash NextGen 300+ using normal phase silica flash columns (Chromatography Direct; Modulus B Series; 12, 25 or 40 g; 40 – 63 µm; 60 Å).

HPLC Analyses: HPLC analyses were obtained on a Shimadzu LC-2050C 3D. Separation was achieved using Daicel CHIRALPAK IB, IC and IG columns using the method stated. HPLC traces of enantiomerically enriched compounds were compared with authentic racemic traces.

Optical Rotations ($[\alpha]_D$ values): Specific rotations were recorded using a Bellingham and Stanley Ltd. ADP 450 polarimeter operating at sodium D line with a Bellingham and Stanley Ltd. 0.5 mL cell (*l* = 0.25 dm). Concentrations (c) are reported in g/100 mL.

Melting Point: Melting points were determined using a Stuart Scientific SMP10 melting point apparatus and are uncorrected.

Solvents: All solvents for air- and moisture sensitive techniques were obtained from an anhydrous solvent system (Innovative Technology). Reaction solvent tetrahydrofuran (THF) (Fisher, HPLC grade) was dried by percolation through two columns packed with neutral alumina under a positive pressure of argon. Reaction solvent toluene (ACS grade) was dried by percolation through a column packed with neutral alumina and a column packed with Q5 reactant (supported copper catalyst for scavenging oxygen) under a positive pressure of argon. Reaction solvent methyl *tert*-butyl ether (MTBE) was purchased from Fisher Scientific UK (extra dry). Solvents for work-up, filtration, transfers, chromatography, and recrystallisation were chloroform ($CHCl_3$) (ACS grade, amylene stabilised), dichloromethane (CH_2Cl_2) (ACS grade, amylene stabilised), diethyl ether (Et_2O) (Fisher, BHT stabilised ACS grade), ethyl acetate (EtOAc) (Fisher, ACS grade), hexane (ACS grade), methanol (MeOH) (ACS grade), ethanol (EtOH) (ACS grade), pentane (ACS grade), and petroleum ether (40–60 °C, ACS grade).

Chemicals: All reagents were purchased from Sigma Aldrich, Alfa Aesar, Acros Organics, Fluorochem and Fisher Scientific UK and used as received, or synthesised within the laboratory.

Diastereoselectivity: For optimisation reactions, the diastereoselectivity was determined using ^{1}H NMR spectroscopy of the crude reaction mixture. For the substrate scope, the diastereoselectivity was determined using ^{1}H NMR spectroscopy of both the crude reaction mixture and isolated product (i.e. before and after purification).

2. General Procedures

General Procedure A: Substrate Synthesis

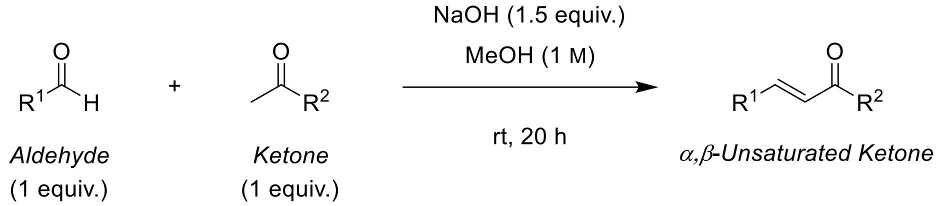

Following the procedure of Nicholson and Thomas,[1] the ketone (20 mmol, 1 equiv.) was added to a stirred solution of NaOH (30 mmol, 1.5 equiv.) in MeOH (20 mL, 1 M) and stirred at room temperature for 10 minutes. The aldehyde (20 mmol, 1 equiv.) was added dropwise and the solution was stirred for 20 hours at room temperature. Brine (20 mL) was added to the solution resulting in the precipitation of salts. The solution was extracted with dichloromethane (3 × 15 mL). The combined organic extracts were washed with brine (20 mL), water (20 mL), dried (Na_2SO_4), filtered and reduced *in vacou*. The resulting α,β-unsaturated ketones were purified by recrystallisation (*n*-hexane).

General Procedure B: Racemic Reductive-coupling Conditions

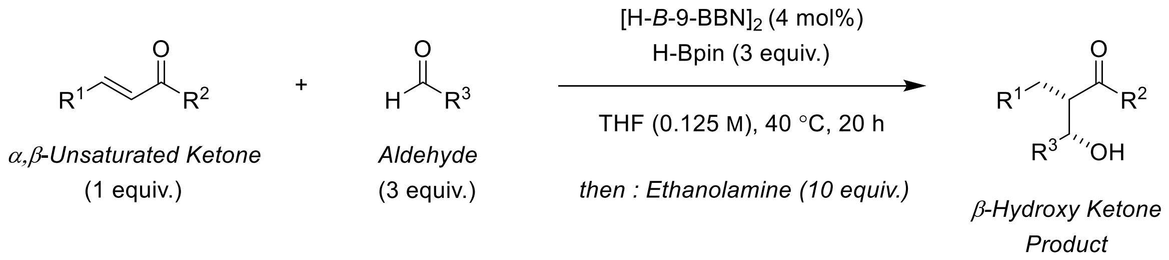

α,β-Unsaturated ketone (0.5 mmol, 1.0 equiv.), aldehyde (1.5 mmol, 3 equiv.) 9-borabicyclo(3.3.1)nonane ([H-*B*-9-BBN]$_2$) (0.02 mmol, 4 mol%) and 4,4,5,5-tetramethyl-1,3,2-dioxaborolane (HBpin) (1.5 mmol, 3 equiv.) were stirred in tetrahydrofuran (THF) (0.125 M) at 40 °C for 20 hours. The reaction was cooled to room temperature and ethanolamine (5.0 mmol, 10 equiv.) was added dropwise. The precipitate was removed by filtration, washing with Et_2O, then the crude product was concentrated *in vacuo.* The crude product was purified by flash column chromatography.

General Procedure C: Enantioenriched Reductive-coupling Conditions

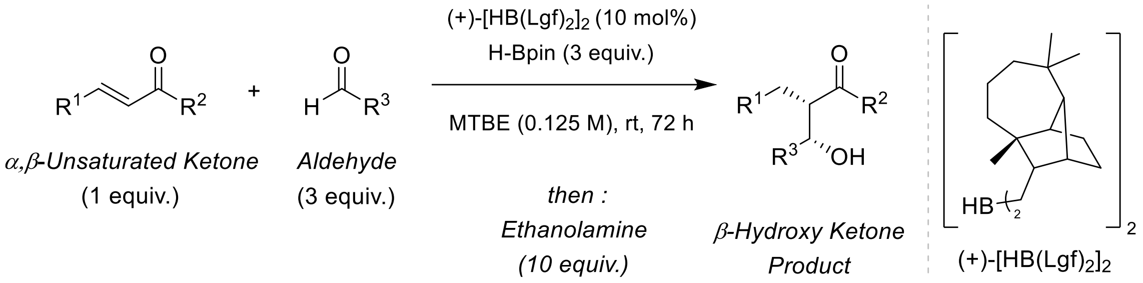

α,β-Unsaturated ketone (0.5 mmol, 1.0 equiv.), aldehyde (1.5 mmol, 3 equiv.) dilongifolylborane ((+)-[HB(Lgf)$_2$]$_2$) (0.05 mmol, 10 mol%) and 4,4,5,5-tetramethyl-1,3,2-dioxaborolane (HBpin) (1.5 mmol, 3 equiv.) were stirred in MTBE (0.125 M) at room temperature for 72 hours. Ethanolamine (5.0 mmol, 10 equiv.) was added dropwise. The precipitate was removed by filtration, washing with Et_2O, then the crude product was concentrated *in vacuo*. The crude product was purified by flash column chromatography.

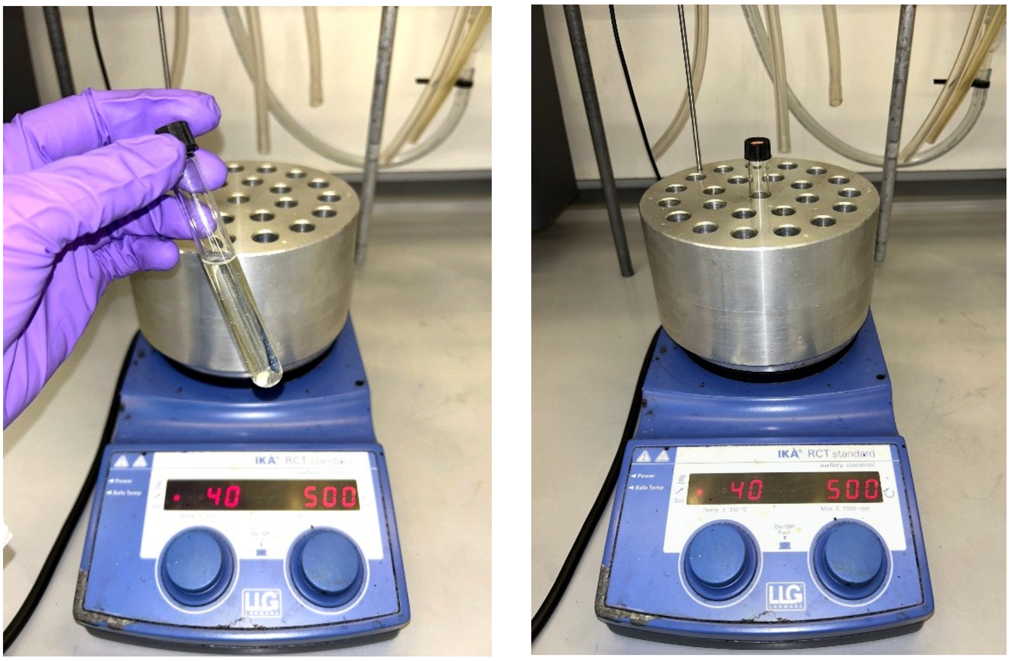

Reaction Set-up for General Procedures B and C

3. Reaction Optimisations

Table S1: Optimisation of Reaction Conditions

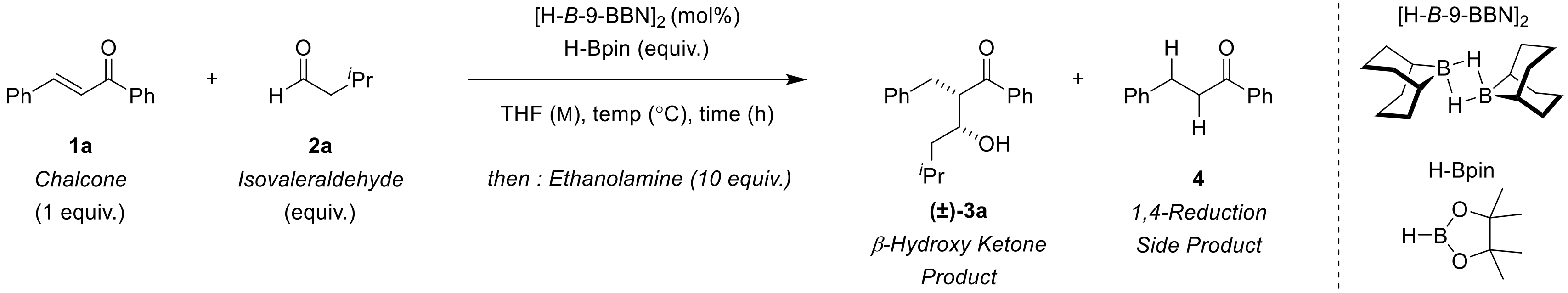

Entry	2a (equiv.)	H-Bpin (equiv.)	[H-*B*-9-BBN]$_2$ (mol %)	Concentration (M)	Time (h)	Temperature (°C)	Unreacted Chalcone (%)	Aldol Product (±)-3a Yield (%)	Side Product 4 Yield (%)	d.r. (*syn*:*anti*)
1	2.0	None	50	0.125	18	40	<5	**>95**	<5	>95:5
2	1.0	2.0	4	0.125	18	40	<5	**67**	23	>95:5
3	1.2	2.0	4	0.125	18	40	<5	**81**	15	>95:5
4	1.5	2.0	4	0.125	18	40	<5	**89**	<5	>95:5
**5**	**2.0**	**2.0**	**4**	**0.125**	**18**	**40**	**<5**	**>95**	**<5**	**>95:5**
**6**	**3.0**	**2.0**	**4**	**0.125**	**18**	**40**	**<5**	**>95**	**<5**	**>95:5**
7	4.0	2.0	4	0.125	18	40	<5	**91**	6	>95:5
8	6.0	2.0	4	0.125	18	40	<5	**86**	12	>95:5
9	1.5	2.0	4	0.125	4	40	6	**56**	40	>95:5
10	2.0	2.0	4	0.125	4	40	12	**66**	36	>95:5
11	3.0	2.0	4	0.125	4	40	8	**70**	26	>95:5

12	4.0	2.0	4	0.125	4	40	10	**75**	24	>95:5
13	6.0	2.0	4	0.125	4	40	21	**43**	58	>95:5
14	2.0	2.0	4	0.125	6	40	6	**76**	28	>95:5
15	3.0	2.0	4	0.125	6	40	5	**83**	21	>95:5
16	4.0	2.0	4	0.125	6	40	7	**84**	25	>95:5
17	1.5	2.0	4	0.125	7	40	11	**62**	24	>95:5
18	1.5	2.0	4	0.125	7	50	<5	**79**	8	>95:5
19	2.0	2.0	4	0.125	8	40	6	**82**	16	>95:5
20	3.0	2.0	4	0.125	8	40	8	**76**	29	>95:5
21	1.5	2.0	1	0.125	18	40	6	**54**	26	>95:5
22	1.5	2.0	2	0.125	18	40	<5	**62**	10	>95:5
23	1.5	2.0	3	0.125	18	40	<5	**87**	<5	>95:5
24	1.5	2.0	5	0.125	18	40	<5	**91**	<5	>95:5
25	1.5	2.0	6	0.125	18	40	<5	**87**	<5	>95:5
26	1.5	2.0	8	0.125	18	40	<5	**80**	<5	>95:5
27	1.5	2.0	4	0.125	18	30	<5	**81**	10	>95:5
28	1.5	2.0	4	0.125	18	RT	7	**63**	29	>95:5
29	1.5	1.0	4	0.125	18	40	7	**59**	33	>95:5
30	1.5	1.4	4	0.125	18	40	5	**81**	<5	>95:5
31	1.5	1.8	4	0.125	18	40	<5	**86**	<5	>95:5
**32**	**1.5**	**3.0**	**4**	**0.125**	**18**	**40**	**<5**	**>95**	**<5**	**>95:5**
33	1.2	2.0	4	0.100	18	40	<5	**74**	<5	>95:5
34	1.2	2.0	4	0.166	18	40	<5	**25**	32	>95:5

Table S2: Solvent Screening

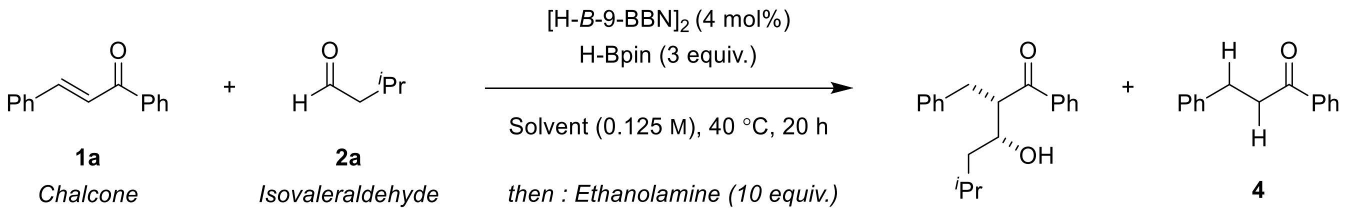

**1a** *Chalcone* (1 equiv.)	**2a** *Isovaleraldehyde* (2 equiv.)	**(±)-3a** *β-Hydroxy Ketone Product*	**4** *1,4-Reduction Side Product*

Entry	Solvent	Unreacted Chalcone (%)	Aldol Product 3a Yield (%)	Side Product 4 Yield (%)	d.r. (*syn*:anti)
**1**	**THF**	**<5**	**>95**	**<5**	**>95:5**
2	2,2,5,5-tetramethyl THF	34	21	11	>95:5
3	MTBE	<5	83	<5	>95:5
4	Toluene	<5	89	<5	>95:5
5	Acetonitrile	<5	90	<5	>95:5
6	Pentane	<5	40	<5	>95:5
7	Hexane	<5	43	<5	>95:5
8	Cyclohexane	<5	32	<5	>95:5

Table S3: Borane Screening

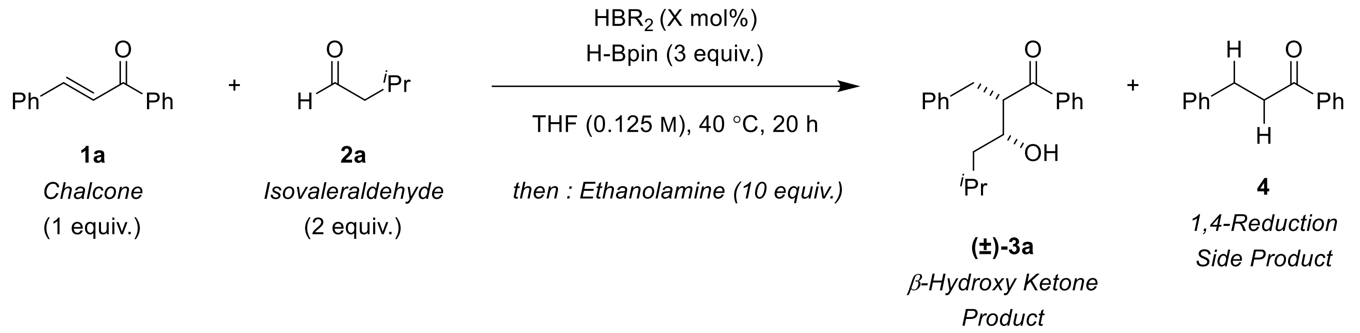

Entry	HBR_2	mol%	Unreacted Chalcone (%)	Aldol Product 3a Yield (%)	Side Product 4 Yield (%)	d.r. (*syn*:anti)
1	**-**	**-**	34	14	45	**-**
2	$Me_2S·BH_3$	20	<5	**-**	-	-
3	THF·$HBSia_2$	5	5	**32**	36	85:15
4		10	7	**41**	34	92:8
5	$[HB(C_6F_5)_2]_2$	5	7	**34**	43	95:5
6	$[HBCy_2]_2$	4	6	**68**	34	>95:5
7		5	<5	**79**	15	>95:5
8	**[H-*B*-9-BBN]$_2$**	**4**	**<5**	**>95**	**<5**	**>95:5**

Figure S1: Effect of a One-pot Versus Two-step Protocol on Product Diastereoselectivity

A) One-pot Protocol: Excellent Diastereoselectivity

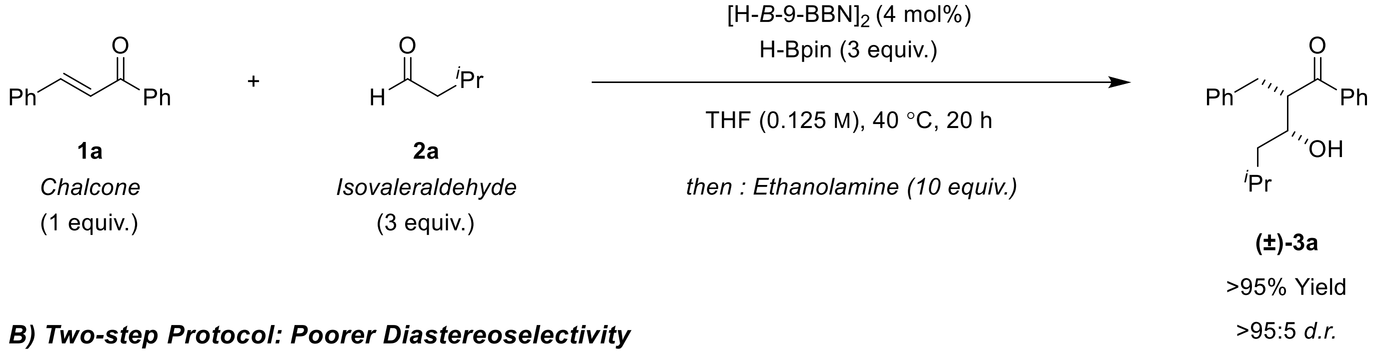

(±)-3a
>95% Yield
>95:5 *d.r.*

B) Two-step Protocol: Poorer Diastereoselectivity

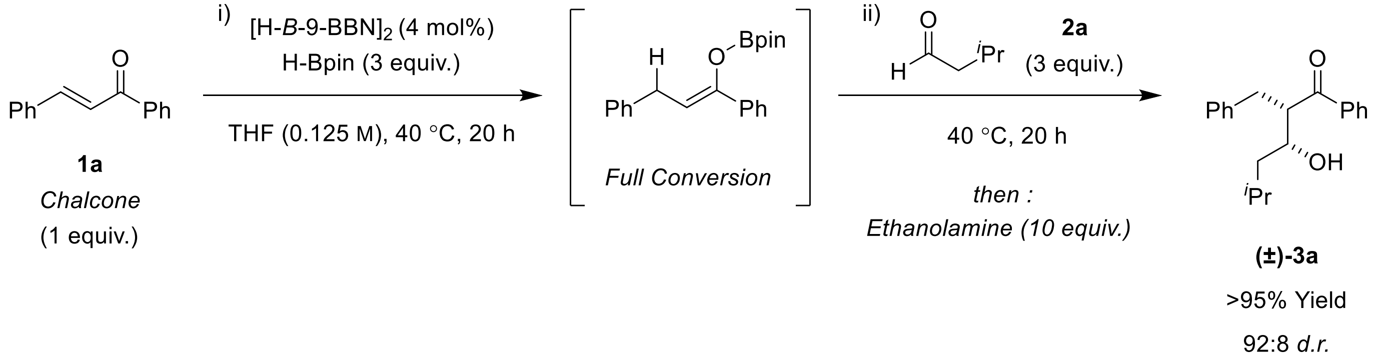

(±)-3a
>95% Yield
92:8 *d.r.*

Table S4: Enantioenriched Borane Screen

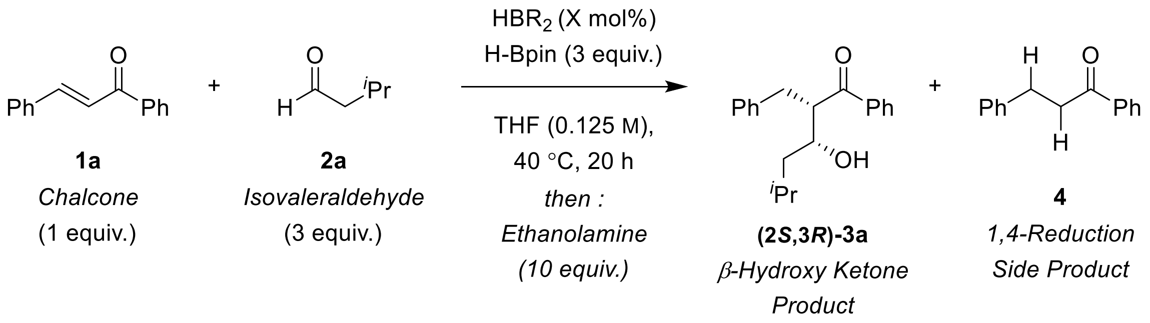

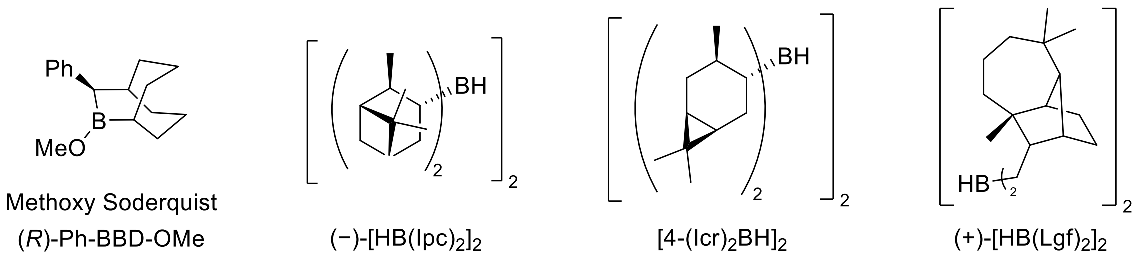

Entry	HBR$_2$	mol%	Unreacted Chalcone (%)	(2S,3R)-3a Yield (%)	Side Product 4 Yield (%)	d.r. (*syn*:anti)	e.r.
1	(*R*)-Ph-BBD-OMe + 5 equiv. HBpin	10	<5	92 (77)	<5	>95:5	48:52
2	(−)-[HB(Ipc)$_2$]$_2$	10	<5	67 (57)	18	90:10	59:41
3		50 (no HBpin)	<5	69 (54)	12	90:10	69:31
4	[4-(Icr)$_2$BH]$_2$	10	15	67 (56)	6	85:15	56:44
5		50	15	24 (16)	55	-	68:32
6	(+)-[HB(Lgf)$_2$]$_2$	10	<5	79 (67)	24	>95:5	86:14
7		50	<5	87 (80)	19	>95:5	87:13

Table S5: Enantioenriched Reaction Optimisation with Dilongifolylborane

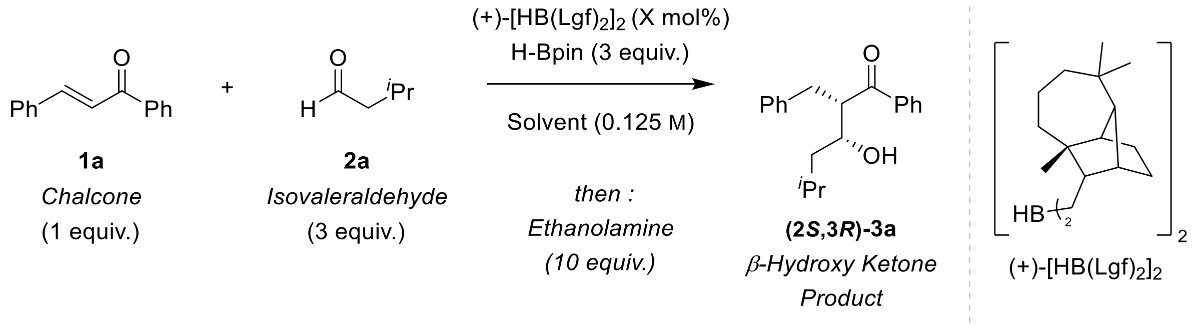

Entry	Mol%	Solvent	Temperature (°C)	Time (h)	Yield (%)	d.r. (*syn*:anti)	e.r.
1	10	THF	40	24	62 (48)	>95:5	82:18
2	Stoichiometric	THF	40	24	74 (64)	>95:5	82:18
3	10	THF	rt	72	79 (67)	>95:5	86:14
4	Stoichiometric	THF	rt	72	87 (80)	>95:5	87:13
5	10	MTBE	rt	72	91 (80)	>95:5	90:10
6	Stoichiometric	MTBE	rt	72	84 (78)	>95:5	92:8
7	10	MTBE	5	168	>95 (95)	>95:5	86:16
8	10	DME	rt	72	92 (79)	>95:5	85:15
9	10	Dioxane	rt	72	84 (76)	>95:5	89:11
10	10	Cyclopentyl Methyl Ether	rt	72	>95 (90)	>95:5	87:13
11	10	EtOAc	rt	72	>95 (77)	>95:5	88:12
12	Stoichiometric	EtOAc	rt	72	>95 (70)	>95:5	90:10
13	10	Ethyl Isovalerate	rt	72	>95 (90)	>95:5	86:14
14	10	*t*-Butyl acetate	rt	72	>95 (86)	>95:5	86:14
15	10	Dimethyl Carbonate	rt	72	>95 (93)	>95:5	86:14
16	10	Hexane	rt	72	70 (64)	>95:5	77:23
17	10	Toluene	rt	72	>95 (88)	>95:5	83:17
18	Stoichiometric	Toluene	rt	72	93 (90)	>95:5	89:11

4. Preparation of α,β-Unsaturated Ketones

The following α,β-unsaturated ketones were available in the laboratory, previously prepared in accordance with literature: **S1**,[1] **S2**,[1] **S3**,[1] **S4**,[1] **S5**,[1] **S6**,[1] **S7**,[1] **S8**[1] and **7**[2].

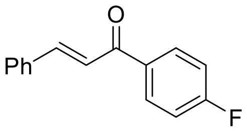

S1

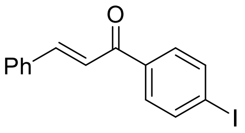

S2

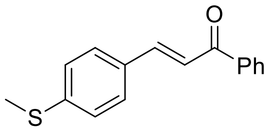

S3

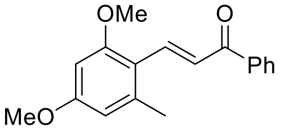

S4

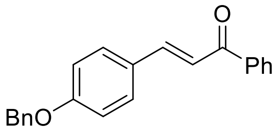

S5

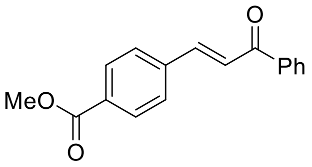

S6

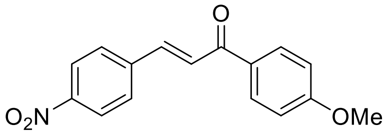

S7

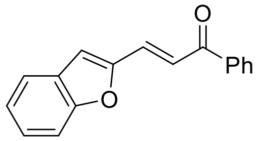

S8

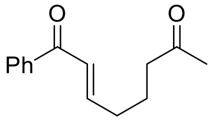

7

(2*E*)-1-Cyclopropyl-3-phenyl-2-propen-1-one (S9)

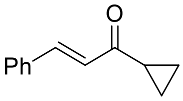

S9

Following General Procedure A, cyclopropyl methyl ketone (2.0 mL, 20 mmol, 1 equiv.), benzaldehyde (2.5 mL, 20 mmol, 1 equiv.), sodium hydroxide (1.2 g, 30 mmol, 1.5 equiv.) and methanol (20 mL, 1 M) gave crude product that was purified by trituration with *n*-hexane to give the α,β-unsaturated ketone **S9** as an amorphous white solid (1.1 g, 6.4 mmol, 33%).

^{1}H NMR: (600 MHz, $CDCl_3$)

0.96 – 1.00 (m, 2H), 1.16 – 1.18 (m, 2H), 2.25 (tt, *J* = 7.9, 4.6 Hz, 1H), 6.88 (d, *J* = 16.2 Hz, 1H), 7.39 – 7.40 (m, 3H), 7.56 – 7.58 (m, 2H), 7.62 (d, *J* = 16.1 Hz, 1H).

^{13}C{^{1}H} NMR: (126 MHz, $CDCl_3$)

11.5, 19.8, 126.6, 128.4, 129.1, 130.5, 134.9, 142.1, 200.2.

Spectroscopic data were in accordance with those previously reported.[3]

(1*E*)-4,4-Dimethyl-1-phenyl-1-penten-3-one (S10)

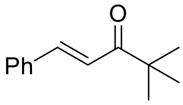

S10

Following General Procedure A, 3,3-dimethyl-2-butanone (2.0 mL, 20 mmol, 1 equiv.), benzaldehyde (2.5 mL, 20 mmol, 1 equiv.), sodium hydroxide (1.2 g, 30 mmol, 1.5 equiv.) and methanol (20 mL, 1 M) gave crude product that was purified by trituration with *n*-hexane to give the α,β-unsaturated ketone **S10** as an amorphous white solid (0.31 g, 1.6 mmol, 8%).

^{1}H NMR: (500 MHz, $CDCl_3$)

1.24 (s, 9H), 7.13 (d, *J* = 15.6 Hz, 1H), 7.37 – 7.40 (m, 3H), 7.57 – 7.58 (m, 2H), 7.68 (d, *J* = 15.6 Hz, 1H).

^{13}C{^{1}H} NMR: (126 MHz, $CDCl_3$)

26.5, 43.4, 120.9, 128.4, 129.0, 130.3, 135.1, 143.0, 204.4.

Spectroscopic data were in accordance with those previously reported.[4]

(*E*)-7-Oxo-7-phenylhept-5-enal (5)

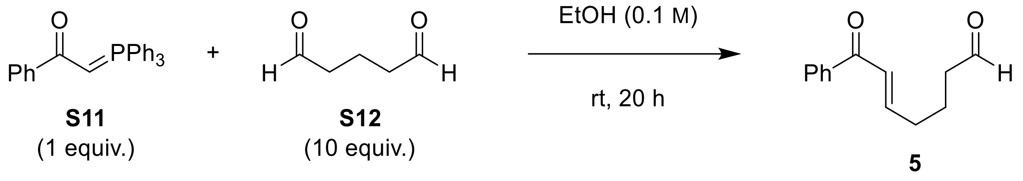

By modification of procedure reported by Benaglia and co-workers,[5] 1-phenyl-2-(triphenylphosphaneylidene)ethan-1-one **S11** (1.00 g, 2.5 mmol, 1 equiv.) was dissolved in EtOH (30 mL) at room temperature, then glutaric aldehyde **S12** (4.7 mL, 50 mmol, 10 equiv.) was added. The reaction was stirred at room temperature for 20 hours. Water (50 mL) was added and the reaction mixture was extracted with Et_2O (3 × 50 mL). The combined organic extracts were washed with HCl (0.2 M, 3 × 50 mL), brine (3 × 50 mL), dried (Na_2SO_4), filtered and reduced *in vacou*. The crude compound was purified by flash silica gel chromatography (80:20 *n*-hexane/EtOAc) to give the product as a yellow oil (325 mg, 1.6 mmol, 64%).

^{1}H NMR: (500 MHz, $CDCl_3$)

1.87 (quin., *J* = 7.2 Hz, 2H), 2.31 – 2.40 (m, 2H), 2.51 (dt, *J* = 7.3, 1.4 Hz, 2H), 6.90 (dt, *J* = 15.4, 1.4 Hz, 1H), 7.00 (dt, *J* = 15.4, 6.8 Hz, 1H), 7.42 – 7.50 (m, 2H), 7.51 – 7.58 (m, 1H), 7.91 (dd, *J* = 8.4, 1.3 Hz, 2H), 9.78 (s, 1H).

^{13}C{^{1}H} NMR: (126 MHz, $CDCl_3$)

20.6, 32.0, 43.1, 126.7, 128.6, 128.7, 132.9, 137.9, 148.1, 190.6, 201.7.

Spectroscopic data were in accordance with those previously reported.[6]

5. Preparation of Boron Reagents

The following boranes were available in the laboratory, previously prepared in accordance with literature: **S13**[7], **S14**[8], **S15**[9].

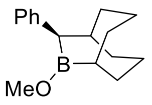

Methoxy Soderquist
(*R*)-Ph-BBD-OMe
S13

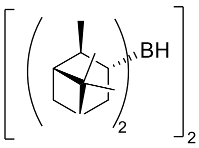

(−)-$[HB(Ipc)_2]_2$
S14

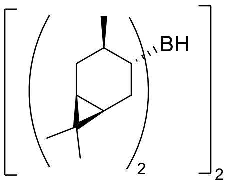

$[4\text{-}(Icr)_2BH]_2$
S15

4,4,5,5-Tetraethyl-1,3,2-dioxaborolane (HB(Epin)) (S16)

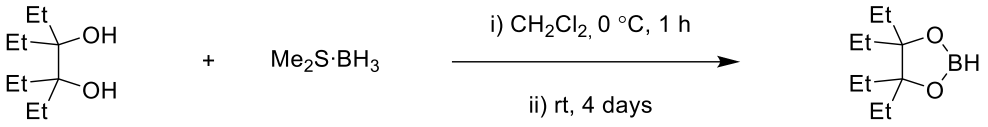

(1.1 equiv.) (1.0 equiv.) **S16**

Procedure adapted from Kochi and co-workers.[10] A solution of 3,4-diethylhexane-3,4-diol (4.8 g, 28 mmol, 1.1 equiv.) in CH_2Cl_2 (10 mL, 2.8 M) was added dropwise over 1 hour to a solution of $Me_2S{\cdot}BH_3$ (2.5 mL, 26 mmol, 1 equiv.) in CH_2Cl_2 (10 mL, 2.6 M) at 0 °C. The solution was warmed to room temperature and stirred for 4 days. The crude mixture was purified using a method reported by Thomas and co-workers to obtain the resulting dioxaborolane **S16** as a colourless liquid (2.6 g, 14 mmol, 54%).[11]

^{1}H NMR: (500 MHz, $CHCl_3$)

0.78 (t, *J* = 7.5 Hz, 12H), 1.43 (sext., *J* = 7.5 Hz, 4H), 1.55 (sext., *J* = 7.5 Hz, 4H), 4.20 (br q, *J* = 147.8 Hz, 1H)

^{13}C{^{1}H} NMR: (126 MHz, $CDCl_3$)

8.9, 26.4, 88.6

^{11}B NMR: (128 MHz, C_6D_6)

27.7 (d)

Spectroscopic data were in accordance with those previously reported.[10, 11]

Dilongifolylborane, (+)-[HB(Lgf)$_2$]$_2$, (S17)

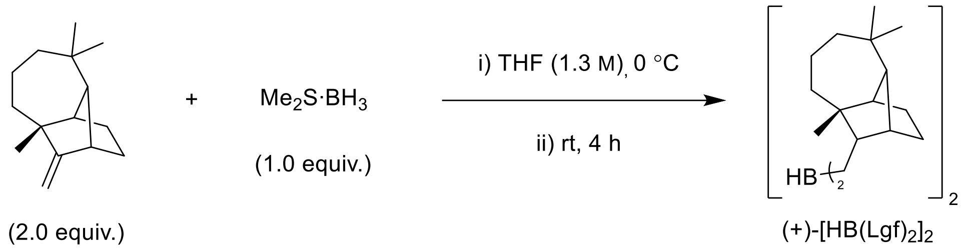

S17

Procedure adapted from Brown and co-workers.[9, 12] (+)-Longifolene (4.4 mL, 20 mmol, 2.0 equiv.) was added dropwise to a solution of $Me_2S·BH_3$ (0.95 mL,10 mmol, 1.0 equiv.) in THF (8.0 mL, 1.3 M) at 0 °C. The solution was warmed to room temperature and stirred overnight. Precipitation of an amorphous white solid was observed. The solvent (THF) and SMe_2 were removed by cannula filtration and the amorphous white solid was washed with diethyl ether (3 × 10 mL). Residual solvent was removed *in vacou*. The resulting dialkylborane **S17** was obtained as an amorphous white solid (1.6 g, 3.8 mmol, 38%).

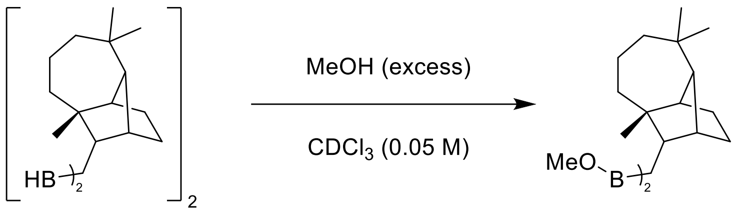

S17 (1 equiv.) **S18**

Dilongifolylborane **S17** is insoluble in all common solvents. Therefore, to analyse the sample, dilongifolylborane **S17** was methanolysed in an NMR tube with excess MeOH to form soluble *B*-methoxy-dilongifolylborane **S18.**

^{1}H NMR: (500 MHz, $CHCl_3$)

0.82 (s, 6H), 0.86 (s, 6H), 0.88 (s, 6H), 0.89 – 0.92 (m, 2H), 1.04 – 1.09 (m, 2H), 1.14 – 1.18 (m, 4H), 1.25 (s, 2H), 1.26 – 1.59 (m, 18H), 1.87 (d, *J* = 4.2 Hz, 2H), 3.62 (s, 3H)

$^{13}C\{^1H\}$ NMR: (126 MHz, $CDCl_3$)

21.4, 22.7 (br s), 25.0, 31.0, 31.5, 32.2, 32.9, 34.3, 37.3, 39.3, 44.8, 45.7, 45.7, 51.5, 53.2, 64.7

^{11}B NMR: (160 MHz, THF)

54.6 (br s)

Spectroscopic data were in accordance with those previously reported.[9, 12]

6. Racemic Substrate Scope

2-Benzyl-3-hydroxy-5-methyl-1-phenylhexan-1-one (3a)

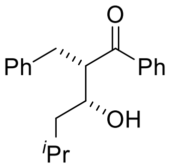

3a

Following General Procedure B, (*E*)-chalcone (100 mg, 0.50 mmol, 1.0 equiv.), isovaleraldehyde (0.16 mL, 1.5 mmol, 3 equiv.), [H-*B*-9-BBN]$_2$ (5 mg, 0.02 mmol, 4 mol%), HBpin (0.22 mL, 1.5 mmol, 3 equiv.) and THF (4 mL, 0.125 M) were reacted to give the crude aldol product (>95:5 *d.r.*) that was purified by flash column chromatography (hexane:ethyl acetate, 99:1 to 90:10) to give the β-hydroxy ketone product **3a** as an amorphous white solid (130 mg, 0.44 mmol, 87%, >95:5 *d.r.*).

^{1}H NMR: (500 MHz, $CDCl_3$)

0.91 (d, *J* = 6.6 Hz, 3H), 0.93 (d, *J* = 6.7 Hz, 3H) 1.25 – 1.30 (m, 1H), 1.57 – 1.63 (m, 1H), 1.79 – 1.89 (m, 1H), 2.63 (d, *J* = 3.3 Hz, 1H), 3.09 – 3.18 (m, 2H), 3.75 – 3.78 (m, 1H), 4.00 – 4.04 (m, 1H), 7.06 – 7.10 (m, 1H), 7.12 – 7.17 (m, 4H), 7.32 -7.35 (m, 2H), 7.46 – 7.49 (m, 1H), 7.67 – 7.69 (m, 2H).

^{13}C{^{1}H} NMR: (126 MHz, $CDCl_3$)

22.0, 23.6, 25.0, 33.7, 44.1, 53.7, 70.3, 126.3, 128.5, 128.6, 128.6, 129.1, 133.3, 137.6, 139.8, 205.2.

Spectroscopic data were in accordance with those previously reported.[13]

2-Benzyl-3-hydroxy-1-phenyloctan-1-one (3b)

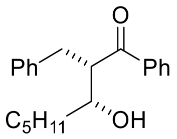

3b

Following General Procedure B, (*E*)-chalcone (100 mg, 0.50 mmol, 1.0 equiv.), hexanal (0.18 mL, 1.5 mmol, 3 equiv.), [H-*B*-9-BBN]$_2$ (5 mg, 0.02 mmol, 4 mol%), HBpin (0.22 mL, 1.5 mmol, 3 equiv.) and THF (4 mL, 0.125 M) gave crude product (>95:5 *d.r.*) that was purified by flash column chromatography (hexane:ethyl acetate, 99:1 to 90:10) to give the β-hydroxy ketone product **3b** as a colourless oil (120 mg, 0.39 mmol, 77%, >95:5 *d.r.*).

^{1}H NMR: (500 MHz, $CDCl_3$)

0.88 (t, *J* = 7.0 Hz, 3H), 1.26 – 1.39 (m, 5H), 1.50 – 1.53 (m, 2H), 1.58 – 1.64 (m, 1H), 2.76 (s, 1H), 3.11 – 3.19 (m, 2H), 3.80 – 3.83 (m, 1H), 3.92 – 3.96 (m, 1H), 7.06 – 7.10 (m, 1H), 7.12 – 7.17 (m, 4H), 7.31 – 7.35 (m, 2H), 7.46 – 7.49 (m, 1H), 7.68 – 7.69 (m, 2H).

^{13}C{^{1}H} NMR: (126 MHz, $CDCl_3$)

14.1, 22.7, 25.9, 31.8, 33.7, 35.0, 53.5, 72.3, 126.3, 128.4, 128.5, 128.6, 129.1, 133.3, 137.6, 139.8, 205.1.

IR ν_{max}: (neat)

3439 (w, br), 2956 (m), 2930 (m), 2858 (m), 1668 (s).

HRMS: (*m/z,* ESI$^+$)

Requires: 311.2006 ($C_{21}H_{27}O_2$, $[M + H]^+$), Found: 311.2006

2-Benzyl-3-hydroxy-1-phenyldodecan-1-one (3c)

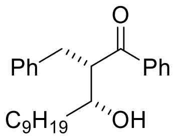

3c

Following General Procedure B, (*E*)-chalcone (100 mg, 0.50 mmol, 1.0 equiv.), decanal (0.28 mL, 1.5 mmol, 3 equiv.), [H-*B*-9-BBN]$_2$ (5 mg, 0.02 mmol, 4 mol%), HBpin (0.22 mL, 1.5 mmol, 3 equiv.) and THF (4 mL, 0.125 M) gave crude product (>95:5 *d.r.*) that was purified by flash column chromatography (hexane:ethyl acetate, 99:1 to 90:10) to give the β-hydroxy ketone product **3c** as a colourless oil (160 mg, 0.45 mmol, 90%, >95:5 *d.r.*).

^{1}H NMR: (500 MHz, $CDCl_3$)

0.87 – 0.90 (m, 3H), 1.26 – 1.30 (m, 12H), 1.35 – 1.39 (m, 1H), 1.46 – 1.56 (m, 2H), 1.58 – 1.66 (m, 1H), 2.70 – 2.71 (m, 1H), 3.10 – 3.19 (m, 2H), 3.81 (dt, *J* = 9.4, 4.4 Hz, 1H), 3.91 – 3.95 (m, 1H), 7.06 – 7.10 (m, 1H), 7.12 – 7.17 (m, 4H), 7.32 – 7.35 (m, 2H), 7.46 – 7.49 (m, 1H), 7.67 – 7.69 (m, 2H).

^{13}C{^{1}H} NMR: (126 MHz, $CDCl_3$)

14.2, 22.8, 26.2, 29.4, 29.6, 29.6, 29.7, 32.0, 33.7, 35.0, 53.4, 72.3, 126.3, 128.4, 128.5, 128.6, 129.1, 133.3, 137.6, 139.8, 205.1.

IR v_{max}: (neat)

3426 (w, br), 2924 (m), 2854 (m), 1672 (s).

HRMS: (*m/z,* ESI$^+$)

Requires: 367.2632 ($C_{25}H_{35}O_2$, $[M + H]^+$), Found: 367.2634 (+0.65 ppm)

2-Benzyl-3-cyclopropyl-3-hydroxy-1-phenylpropan-1-one (3d)

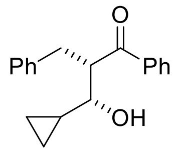

3d

Following General Procedure B, (*E*)-chalcone (100 mg, 0.50 mmol, 1.0 equiv), cyclopropanecarboxaldehyde (0.10 mL, 1.5 mmol, 3 equiv.), [H-*B*-9-BBN]$_2$ (5 mg, 0.02 mmol, 4 mol%), HBpin (0.22 mL, 1.5 mmol, 3 equiv.) and THF (4 mL, 0.125 M) gave crude product (>95:5 *d.r.*) that was purified by flash column chromatography (hexane:ethyl acetate, 99:1 to 90:10) to give the β-hydroxy ketone product **3d** as an amorphous white solid (110 mg, 0.39 mmol, 77%, >95:5 *d.r.*).

^{1}H NMR: (500 MHz, $CDCl_3$)

0.20 – 0.25 (m, 1H), 0.34 – 0.43 (m, 2H), 0.51 – 0.56 (m, 1H), 0.96 – 1.02 (m, 1H), 2.47 (d, *J* = 2.7 Hz, 1H), 3.16 – 3.19 (m, 1H), 3.21 – 3.28 (m, 2H), 4.02 (dt, *J* = 9.9, 4.8 Hz, 1H), 7.07 – 7.11 (m, 1H), 7.15 – 7.17 (m, 4H), 7.31 – 7.34 (m, 2H), 7.45 – 7.48 (m, 1H), 7.70 – 7.72 (m, 2H).

^{13}C{^{1}H} NMR: (126 MHz, $CDCl_3$)

3.6, 3.8, 16.3, 34.7, 54.4, 77.5, 126.3, 128.4, 128.5, 128.5, 129.1, 133.1, 138.1, 139.9, 204.2.

Spectroscopic data were in accordance with those previously reported.[13]

2-Benzyl-3-cyclohexyl-3-hydroxy-1-phenylpropan-1-one (3e)

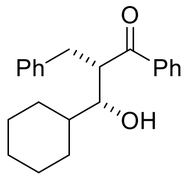

3e

Following General Procedure B, (*E*)-chalcone (100 mg, 0.50 mmol, 1.0 equiv.), cyclohexanecarboxaldehyde (0.18 mL, 1.5 mmol, 3 equiv.), $[H\text{-}B\text{-}9\text{-}BBN]_2$ (5 mg, 0.02 mmol, 4 mol%), HBpin (0.22 mL, 1.5 mmol, 3 equiv.) and THF (4 mL, 0.125 M) gave crude product (>95:5 *d.r.*) that was purified by flash column chromatography (hexane:ethyl acetate, 99:1 to 90:10) to give the β-hydroxy ketone product **3e** as an amorphous white solid (100 mg, 0.31 mmol, 62%, >95:5 *d.r.*).

^{1}H NMR: (500 MHz, $CDCl_3$)

0.95 – 1.03 (m, 1H), 1.09 – 1.19 (m, 3H), 1.21 – 1.31 (m, 1H), 1.55 – 1.61 (m, 1H), 1.66 – 1.74 (m, 2H), 1.78 – 1.83 (m, 2H), 2.02 – 2.05 (m, 1H), 2.76 (d, *J* = 2.9 Hz, 1H), 3.05 (dd, *J* = 13.7, 3.7 Hz, 1H), 3.21 (dd, *J* = 13.7, 10.4 Hz, 1H), 3.61 (dt, *J* = 7.8, 3.1 Hz, 1H), 4.01 (dt, *J* = 10.5, 3.6 Hz, 1H), 7.06 – 7.10 (m, 1H), 7.13 – 7.18 (m, 4H), 7.32 – 7.35 (m, 2H), 7.46 – 7.49 (m, 1H), 7.66 – 7.68 (m, 2H).

^{13}C{^{1}H} NMR: (126 MHz, $CDCl_3$)

26.0, 26.2, 26.5, 29.3, 29.6, 32.7, 40.8, 50.1, 76.2, 126.3, 128.4, 128.5, 128.7, 129.1, 133.3, 137.3, 140.1, 205.3.

IR ν_{max}: (neat)

3498 (w, br), 2924 (s), 2854 (m), 1663 (s).

HRMS: (*m/z,* ESI$^+$)

Requires: 323.2006 ($C_{22}H_{27}O_2$, $[M + H]^+$), Found: 323.2006

2-Benzyl-3-hydroxy-4,4-dimethyl-1-phenylpentan-1-one (3f)

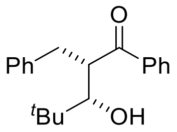

3f

Following General Procedure B, (*E*)-chalcone (100 mg, 0.50 mmol, 1.0 equiv.), trimethylacetaldehyde (0.16 mL, 1.5 mmol, 3 equiv.), [H-*B*-9-BBN]$_2$ (5 mg, 0.02 mmol, 4 mol%), HBpin (0.22 mL, 1.5 mmol, 3 equiv.) and THF (4 mL, 0.125 M) gave crude product (75:25 *d.r.*) that was purified by flash column chromatography (hexane:ethyl acetate, 99:1 to 90:10) to give the β-hydroxy ketone product **3f** as an amorphous white solid (30 mg, 0.10 mmol, 20%, >95:5 *d.r.*).

^{1}H NMR: (500 MHz, CDCl$_3$)

1.02 (s, 9H), 2.46 (d, *J* = 3.8 Hz, 1H), 3.14 (dd, *J* = 13.7, 10.6 Hz, 1H), 3.27 (dd, *J* = 13.7, 3.7 Hz, 1H), 3.63 (app. t, *J* = 3.7 Hz, 1H), 4.03 (app. dt, *J* = 10.6, 3.7 Hz, 1H), 7.03 – 7.06 (m, 1H), 7.09 – 7.13 (m, 4H), 7.29 – 7.32 (m, 2H), 7.43 – 7.46 (m, 1H), 7.63 – 7.65 (m, 2H).

^{13}C{^{1}H} NMR: (126 MHz, CDCl$_3$)

27.1, 34.8, 36.4, 49.5, 78.6, 126.3, 128.4, 128.5, 128.6, 129.1, 133.1, 137.3, 140.0, 205.2.

IR ν_{max}: (neat)

3458 (w, br), 2956 (m), 2928 (w), 2869 (w), 1667 (s).

HRMS: (*m/z,* ESI$^+$)

Requires: 297.1849 ($C_{20}H_{25}O_2$, [M + H]$^+$), Found: 297.1835 (−4.71 ppm)

2-Benzyl-3-hydroxy-5,5-dimethyl-1-phenylhexan-1-one (3g)

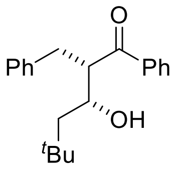

3g

Following General Procedure B, (*E*)-chalcone (100 mg, 0.50 mmol, 1.0 equiv.), 3,3-dimethylbutanal (0.19 mL, 1.5 mmol, 3 equiv.), [H-*B*-9-BBN]$_2$ (5 mg, 0.02 mmol, 4 mol%), HBpin (0.22 mL, 1.5 mmol, 3 equiv.) and THF (4 mL, 0.125 M) gave crude product (>95:5 *d.r.*) that was purified by flash column chromatography (hexane:ethyl acetate, 99:1 to 90:10) to give the β-hydroxy ketone product **3g** as an amorphous white solid (100 mg, 0.32 mmol, 64%, >95:5 *d.r.*).

^{1}H NMR: (500 MHz, $CDCl_3$)

0.97 (s, 9H), 1.41 (d, *J* = 14.5 Hz, 1H), 1.58 (dd, *J* = 14.5, 8.6 Hz, 1H), 2.58 (d, *J* = 2.2 Hz, 1H), 3.08 – 3.20 (m, 2H), 3.73 (dt, *J* = 10.0, 4.1 Hz, 1H), 4.08 – 4.11 (m, 1H), 7.06 – 7.09 (m, 1H), 7.12 – 7.17 (m, 4H), 7.31 – 7.34 (m, 2H), 7.46 – 7.49 (m, 1H), 7.66 – 7.68 (m, 2H).

^{13}C{^{1}H} NMR: (126 MHz, $CDCl_3$)

30.2, 30.6, 33.8, 49.2, 55.1, 69.8, 126.3, 128.4, 128.5, 128.6, 129.1, 133.3, 137.6, 139.8, 205.2.

Spectroscopic data were in accordance with those previously reported.[13]

2-Benzyl-3-hydroxy-5,9-dimethyl-1-phenyldec-8-en-1-one (3h)

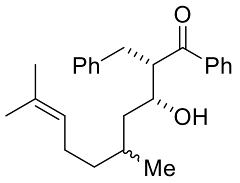

3h

Following General Procedure B, (*E*)-chalcone (100 mg, 0.50 mmol, 1.0 equiv.), (±)-citronellal (0.27 mL, 1.5 mmol, 3 equiv.), [H-*B*-9-BBN]$_2$ (5 mg, 0.02 mmol, 4 mol%), HBpin (0.22 mL, 1.5 mmol, 3 equiv.) and THF (4 mL, 0.125 M) gave crude product (1:1 *d.r.*) that was purified by flash column chromatography (hexane:ethyl acetate, 99:1 to 90:10) to give the β-hydroxy ketone product **3h** as a colourless oil (160 mg, 0.45 mmol, 90%, 1:1 *d.r.*). The product was a mixture of inseparable diastereomers.

^{1}H NMR: (500 MHz, $CDCl_3$)

0.90 (d, *J* = 6.0 Hz, 3H), 0.92 (d, *J* = 6.0 Hz, 3H), 1.13 – 1.22 (m, 3H), 1.26 – 1.34 (m, 1H), 1.37 – 1.44 (m, 1H), 1.48 – 1.51 (m, 2H), 1.59 (d, *J* = 8.5 Hz, 6H), 1.64 – 1.72 (m, 9H), 1.95 – 2.05 (m, 4H), 2.56 (br s, 1H), 2.71 (br s, 1H), 3.07 – 3.18 (m, 4H), 3.74 – 3.79 (m, 2H), 4.04 – 4.05 (m, 2H), 5.06 – 5.12 (m, 2H), 7.06 – 7.09 (m, 2H), 7.11 – 7.17 (m, 8H), 7.32 – 7.35 (m, 4H), 7.46 – 7.49 (m, 2H), 7.67 – 7.69 (m, 4H).

^{13}C{^{1}H} NMR: (126 MHz, $CDCl_3$)

17.8, 17.8, 19.1, 20.4, 25.5, 25.6, 25.9, 25.9, 25.9, 29.2, 29.6, 33.4, 33.9, 36.7, 37.9, 42.2, 42.4, 53.2, 60.0, 70.0, 70.2, 124.8, 126.3, 128.5, 128.5, 128.6, 128.6, 128.6, 129.1, 131.5, 131.6, 133.3, 133.3, 137.5, 137.6, 139.8, 139.9, 205.1, 205.3.

IR v_{max}: (neat)

3675 (w, br), 2989 (m),I 2971 (m), 2911 (m), 1901 (m), 1671 (s).

HRMS: (*m/z,* ESI$^+$)

Requires: 365.2475 ($C_{25}H_{33}O_2$, $[M + H]^+$), Found: 365.2472 (−0.85 ppm)

2-Benzyl-1-(4-fluorophenyl)-3-hydroxy-5-methylhexan-1-one (3i)

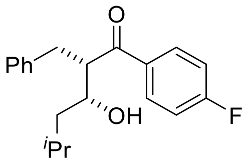

3i

Following General Procedure B, (*E*)-1-(4-fluorophenyl)-3-phenylprop-2-en-1-one (110 mg, 0.50 mmol, 1.0 equiv.), isovaleraldehyde (0.16 mL, 1.5 mmol, 3 equiv.), [H-*B*-9-BBN]$_2$ (5 mg, 0.02 mmol, 4 mol%), HBpin (0.22 mL, 1.5 mmol, 3 equiv.) and THF (4 mL, 0.125 M) gave crude product (>95:5 *d.r.*) that was purified by flash column chromatography (hexane:ethyl acetate, 99:1 to 90:10) to give the β-hydroxy ketone product **3i** as a colourless oil (130 mg, 0.42 mmol, 84%, >95:5 *d.r.*).

^{1}H NMR: (500 MHz, $CDCl_3$)

0.90 – 0.94 (m, 6H), 1.23 – 1.29 (m, 1H), 1.56 – 1.62 (m, 1H), 1.82 – 1.89 (m, 1H), 2.71 (br s, 1H), 3.11 – 3.13 (m, 2H), 3.69 – 3.72 (m, 1H), 4.00 – 4.04 (m, 1H), 6.96 – 6.99 (m, 2H), 7.07 – 7.11 (m, 3H), 7.14 – 7.17 (m, 2H), 7.67 – 7.70 (m, 2H).

^{13}C{^{1}H} NMR: (126 MHz, $CDCl_3$)

21.9, 23.6, 24.9, 34.1, 44.1, 53.9, 70.3, 115.7 (d, *J* = 21.4 Hz), 126.4, 128.6, 129.1, 131.2 (d, *J* = 10.8 Hz), 134.1 (d, *J* = 2.5 Hz), 139.6, 165.9 (d, *J* = 252.0 Hz), 203.7.

^{19}F{^{1}H} NMR: (470 MHz, $CDCl_3$)

−104.7.

IR ν_{max}: (neat)

3450 (w, br), 2957 (w), 2931 (w), 2870 (w), 1668 (s), 1596 (s).

HRMS: (*m/z,* ESI^{+})

Requires: 315.1755 ($C_{20}H_{24}FO_2$, $[M + H]^+$), Found: 315.1756 (0.36 ppm)

2-Benzyl-3-hydroxy-1-(4-iodophenyl)-5-methylhexan-1-one (3j)

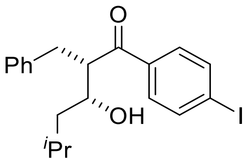

3j

Following General Procedure B, (*E*)-1-(4-iodophenyl)-3-phenylprop-2-en-1-one (170 mg, 0.50 mmol, 1.0 equiv.), isovaleraldehyde (0.16 mL, 1.5 mmol, 3 equiv.), [H-*B*-9-BBN]$_2$ (5 mg, 0.02 mmol, 4 mol%), HBpin (0.22 mL, 1.5 mmol, 3 equiv.) and THF (4 mL, 0.125 M) gave crude product (>95:5 *d.r.*) that was purified by flash column chromatography (hexane:ethyl acetate, 99:1 to 90:10) to give the β-hydroxy ketone product **3j** as an amorphous white solid (100 mg, 0.23 mmol, 46%, >95:5 *d.r.*).

^{1}H NMR: (500 MHz, $CDCl_3$)

0.91 (d, *J* = 6.6 Hz, 3H), 0.93 (d, *J* = 6.7 Hz, 3H), 1.23 – 1.27 (m, 1H), 1.54- 1.59 (m, 1H), 1.80 – 1.84 (m, 1H), 2.57 (d, *J* = 3.7 Hz, 1H), 3.11 (d, *J* = 7.2 Hz, 2H), 3.68 (td, *J* = 7.2, 4.1 Hz, 1H), 3.98 – 4.02 (m, 1H), 7.08 – 7.11 (m, 3H), 7.15 – 7.18 (m, 2H), 7.34 – 7.37 (m, 2H), 7.67 – 7.69 (m, 2H).

^{13}C{^{1}H} NMR: (126 MHz, $CDCl_3$)

21.9, 23.7, 24.9, 34.0, 44.1, 54.0, 70.4, 101.5, 126.5, 128.7, 129.1, 129.8, 136.9, 137.9, 139.6, 204.5.

IR ν_{max}: (neat)

3466 (w, br), 2956 (w), 2931 (w), 2870 (w), 1668 (m), 1579 (m).

HRMS: (*m/z,* ESI$^+$)

Requires: 423.0816 ($C_{20}H_{24}IO_2$, [M + H]$^+$), Found: 423.0826 (+2.48 ppm)

3-Hydroxy-5-methyl-2-(4-(methylthio)benzyl)-1-phenylhexan-1-one (3k)

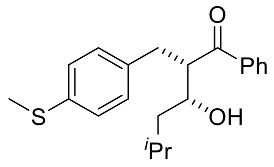

3k

Following General Procedure B, (*E*)-3-(4-(methylthio)phenyl)-1-phenylprop-2-en-1-one (130 mg, 0.50 mmol, 1.0 equiv), isovaleraldehyde (0.16 mL, 1.5 mmol, 3 equiv.), [H-*B*-9-BBN]$_2$ (5 mg, 0.02 mmol, 4 mol%), HBpin (0.22 mL, 1.5 mmol, 3 equiv.) and THF (4 mL, 0.125 M) gave crude product (>95:5 *d.r.*) that was purified by flash column chromatography (hexane:ethyl acetate, 99:1 to 90:10) to give the β-hydroxy ketone product **3k** as an amorphous white solid (130 mg, 0.39 mmol, 78%, >95:5 *d.r.*).

^{1}H NMR: (500 MHz, $CDCl_3$)

0.91 (d, *J* = 6.6 Hz, 3H), 0.93 (d, *J* = 6.7 Hz, 3H), 1.24 – 1.29 (m, 1H), 1.56 – 1.61 (m, 1H), 1.79 – 1.84 (m, 1H), 2.39 (s, 3H), 2.52 (d, *J* = 4.5 Hz, 1H), 3.10 – 3.15 (m, 2H), 3.72 – 3.76 (m, 1H), 3.99 – 4.03 (m, 1H), 7.03 – 7.08 (m, 4H), 7.34 – 7.37 (m, 2H), 7.48 – 7.51 (m, 1H), 7.69 – 7.71 (m, 2H).

^{13}C{^{1}H} NMR: (126 MHz, $CDCl_3$)

16.4, 22.0, 23.6, 25.0, 33.1, 44.1, 53.7, 70.3, 127.3, 128.5, 128.7, 129.7, 133.4, 136.0, 136.9, 137.4, 205.0.

IR ν_{max}: (neat)

3513 (s), 3395 (w, br), 2962 (m), 2929 (m), 2902 (m), 2868 (w), 1657 (s).

HRMS: (*m/z,* ESI$^+$)

Requires: 343.1726 ($C_{21}H_{27}O_2S$, $[M + H]^+$), Found: 343.1730 (+1.08 ppm)

2-(2,4-Dimethoxy-6-methylbenzyl)-3-hydroxy-5-methyl-1-phenylhexan-1-one (3l)

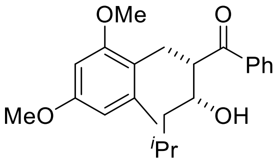

3l

Following General Procedure B, (*E*)-3-(2,4-dimethoxy-6-methylphenyl)-1-phenylprop-2-en-1-one (140 mg, 0.50 mmol, 1.0 equiv.), isovaleraldehyde (0.16 mL, 1.5 mmol, 3 equiv.), $[H\text{-}B\text{-}9\text{-}BBN]_2$ (5 mg, 0.02 mmol, 4 mol%), HBpin (0.22 mL, 1.5 mmol, 3 equiv.) and THF (4 mL, 0.125 M) gave crude product (90:10 *d.r.*) that was purified by flash column chromatography (hexane:ethyl acetate, 99:1 to 90:10) to give the β-hydroxy ketone product **3l** as an amorphous white solid (170 mg, 0.46 mmol, 91%, >95:5 *d.r.*).

^{1}H NMR: (500 MHz, $CDCl_3$)

0.92 (d, *J* = 6.7 Hz, 6H), 1.25 – 1.30 (m, 1H), 1.60 – 1.65 (m, 1H), 1.85 – 1.87 (m, 1H), 2.21 (s, 3H), 3.05 – 3.11 (m, 2H), 3.36 (d, *J* = 3.0 Hz, 1H), 3.62 (s, 3H), 3.70 (s, 3H), 3.73 – 3.76 (m, 1H), 4.04 – 4.08 (m, 1H), 6.11 (d, *J* = 2.5 Hz, 1H), 6.17 (d, *J* = 2.5 Hz, 1H), 7.27 – 7.30 (m, 2H), 7.42 – 7.46 (m, 1H), 7.61 – 7.63 (m, 2H).

$^{13}C\{^1H\}$ NMR: (126 MHz, $CDCl_3$)

20.4, 22.2, 23.6, 24.3, 24.9, 43.8, 49.9, 55.1, 55.4, 70.6, 96.0, 106.7, 119.0, 128.3, 128.3, 133.1, 137.5, 138.3, 158.6, 158.8, 207.1.

IR ν_{max}: (neat)

3482 (w, br), 2956 (w), 2870 (w), 2838 (w), 1661 (m), 1595 (m).

HRMS: (*m/z,* ESI$^+$)

Requires: 371.2217 ($C_{23}H_{31}O_4$, $[M + H]^+$), Found: 371.2207 (−2.66 ppm)

3-Hydroxy-5-methyl-2-(4-phenoxybenzyl)-1-phenylhexan-1-one (3m)

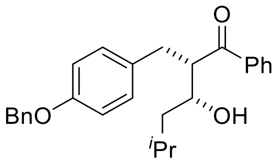

3m

Following General Procedure B, (*E*)-3-(4-phenoxyphenyl)-1-phenylprop-2-en-1-one (150 mg, 0.50 mmol, 1.0 equiv.), isovaleraldehyde (0.16 mL, 1.5 mmol, 3 equiv.), $[H\text{-}B\text{-}9\text{-}BBN]_2$ (5 mg, 0.02 mmol, 4 mol%), HBpin (0.22 mL, 1.5 mmol, 3 equiv.) and THF (4 mL, 0.125 M) gave crude product (>95:5 *d.r.*) that was purified by flash column chromatography (hexane:ethyl acetate, 99:1 to 90:10) to give β-hydroxy ketone product **3m** as an amorphous white solid (120 mg, 0.30 mmol, 59%, >95:5 *d.r.*).

^{1}H NMR: (500 MHz, $CDCl_3$)

0.91 (d, *J* = 6.6 Hz, 3H), 0.93 (d, *J* = 6.7 Hz, 3H), 1.24 – 1.29 (m, 1H), 1.56 – 1.62 (m, 1H), 1.78 – 1.86 (m, 1H), 2.63 (br s, 1H), 3.06 – 3.15 (m, 2H), 3.72 – 3.75 (m, 1H), 4.01 – 4.02 (m, 1H), 4.90 – 4.96 (m, 2H), 6.69 – 6.75 (m, 3H), 7.05 – 7.08 (m, 1H), 7.29 – 7.37 (m, 7H), 7.47 – 7.51 (m, 1H), 7.67 – 7.69 (m, 2H).

^{13}C{^{1}H} NMR: (126 MHz, $CDCl_3$)

22.0, 23.6, 24.9, 33.8, 44.1, 53.5, 70.0, 70.2, 112.9, 115.7, 121.8, 127.5, 128.0, 128,7, 129.6, 133.3, 137.2, 137.6, 141.5, 158.9, 205.2.

IR ν_{max}: (neat)

3484 (m), 3032 (w), 2956 (m), 2943 (m), 2962 (m), 2901 (m), 2867 (w), 1658 (s).

HRMS: (*m/z,* ESI$^+$)

Requires: 403.2268 ($C_{27}H_{31}O_3$, $[M + H]^+$), Found: 403.2270 (+0.57 ppm)

Methyl 4-(2-benzoyl-3-hydroxy-5-methylhexyl)benzoate (3n)

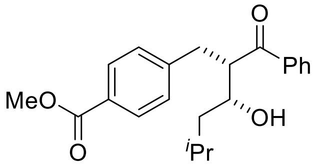

3n

Following General Procedure B, methyl (*E*)-4-(3-oxo-3-phenylprop-1-en-1-yl)benzoate (130 mg, 0.50 mmol, 1.0 equiv.), isovaleraldehyde (0.16 mL, 1.5 mmol, 3 equiv.), $[H\text{-}B\text{-}9\text{-}BBN]_2$ (5 mg, 0.02 mmol, 4 mol%), HBpin (0.22 mL, 1.5 mmol, 3 equiv.) and THF (4 mL, 0.125 M) gave crude product (94:6 *d.r.*) that was purified by flash column chromatography (hexane:ethyl acetate, 99:1 to 90:10) to give the β-hydroxy ketone product **3n** as an amorphous white solid (110 mg, 0.31 mmol, 61%, >95:5 *d.r.*).

^{1}H NMR: (600 MHz, $CDCl_3$)

0.91 (d, *J* = 6.6 Hz, 3H), 0.93 (d, *J* = 6.7 Hz, 3H), 1.24 – 1.29 (m, 1H), 1.57 – 1.61 (m, 1H), 1.79 – 1.86 (m, 1H), 2.54 (d, *J* = 4.3 Hz, 1H), 3.13 – 3.24 (m, 2H), 3.76 – 3.79 (m, 1H), 3.85 (s, 3H), 4.01 – 4.04 (m, 1H), 7.19 – 7.20 (m, 2H), 7.33 – 7.35 (m, 2H), 7.47 – 7.50 (m, 1H), 7.67 – 7.69 (m, 2H), 7.82 – 7.84 (m, 2H).

$^{13}C\{^1H\}$ NMR: (126 MHz, $CDCl_3$)

21.9, 23.6, 25.0, 33.7, 44.2, 52.1, 53.5, 70.3, 128.3, 128.4, 128.8, 129.2, 129.9, 133.6, 137.3, 145.5, 167.1, 204.5.

IR v_{max}: (neat)

3454 (m, br), 2955 (w), 2935 (w), 2899 (w), 1697 (s), 1668 (s), 1432 (m), 1282 (s).

HRMS: (*m/z,* ESI^+)

Requires: 355.1904 ($C_{22}H_{27}O_4$, $[M + H]^+$), Found: 355.1892 (−3.35 ppm)

3-hydroxy-1-(4-methoxyphenyl)-5-methyl-2-(4-nitrobenzyl)hexan-1-one (3o)

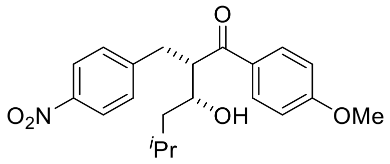

3o

Following General Procedure B, (*E*)-1-(4-methoxyphenyl)-3-(4-nitrophenyl)prop-2-en-1-one (140 mg, 0.50 mmol, 1.0 equiv.), isovaleraldehyde (0.16 mL, 1.5 mmol, 3 equiv.), $[H\text{-}B\text{-}9\text{-}BBN]_2$ (5 mg, 0.02 mmol, 4 mol%), HBpin (0.22 mL, 1.5 mmol, 3 equiv.) and THF (4 mL, 0.125 M) gave crude product (>95:5 *d.r.*) that was purified by flash column chromatography (hexane:ethyl acetate, 99:1 to 90:10) to give the β-hydroxy ketone product **3o** as a colourless oil (100 mg, 0.28 mmol, 55%, >95:5 *d.r.*).

^{1}H NMR: (500 MHz, $CDCl_3$)

0.91 (d, *J* = 6.6 Hz, 3H), 0.93 (d, *J* = 6.7 Hz, 3H), 1.24 – 1.28 (m, 1H), 1.57 – 1.62 (m, 1H), 1.79 – 1.87 (m, 1H), 2.57 (d, *J* = 2.5 Hz, 1H), 3.17 (dd, *J* = 13.5, 4.0 Hz, 1H), 3.28 (dd, *J* = 13.6, 10.4 Hz, 1H), 3.70 – 3.73 (m, 1H), 3.82 (s, 3H), 4.00 – 4.04 (m, 1H), 6.81 – 6.84 (m, 2H), 7.27 – 7.29 (m, 2H), 7.68 – 7.71 (m, 2H), 8.00 – 8.02 (m, 2H).

$^{13}C\{^1H\}$ NMR: (126 MHz, $CDCl_3$)

21.9, 23.7, 25.0, 33.6, 44.3, 52.8, 55.7, 70.3, 114.1, 123.7, 130.0, 130.1, 130.8, 146.6, 148.1, 164.2, 202.0.

IR ν_{max}: (neat)

3463 (w, br), 2956 (m), 2870 (w), 2841 (w), 1656 (m), 1596 (s), 1343 (s), 1168 (s).

HRMS: (*m/z,* ESI^+)

Requires: 372.1805 ($C_{21}H_{26}N_1O_5$, $[M + H]^+$), Found: 372.1804 (−0.40 ppm)

2-(Benzofuran-2-ylmethyl)-3-hydroxy-5-methyl-1-phenylhexan-1-one (3p)

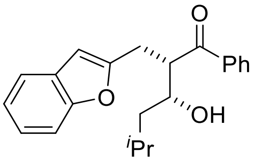

3p

Following General Procedure B, (*E*)-3-(benzofuran-2-yl)-1-phenylprop-2-en-1-one (120 mg, 0.50 mmol, 1.0 equiv.), isovaleraldehyde (0.16 mL, 1.5 mmol, 3 equiv.), [H-*B*-9-BBN]$_2$ (5 mg, 0.02 mmol, 4 mol%), HBpin (0.22 mL, 1.5 mmol, 3 equiv.) and THF (4 mL, 0.125 M) gave crude product (>95:5 *d.r.*) that was purified by flash column chromatography (hexane:ethyl acetate, 99:1 to 90:10) to give the β-hydroxy ketone product **3p** as an amorphous white solid (110 mg, 0.31 mmol, 61%, >95:5 *d.r.*).

^{1}H NMR: (500 MHz, $CDCl_3$)

0.91 (d, *J* = 6.6 Hz, 3H), 0.93 (d, *J* = 6.7 Hz, 3H), 1.28 – 1.33 (m, 1H), 1.57 – 1.63 (m, 1H), 1.80 – 1.89 (m, 1H), 2.66 (d, *J* = 3.3 Hz, 1H), 3.24 – 3.36 (m, 2H), 4.01 – 4.04 (m, 1H), 4.08 – 4.12 (m, 1H), 6.33 (d, *J* = 0.8 Hz, 1H), 7.10 – 7.18 (m, 2H), 7.31 – 7.33 (m, 1H), 7.36 – 7.39 (m, 3H), 7.48 – 7.51 (m, 1H), 7.87 – 7.89 (m, 2H).

^{13}C{^{1}H} NMR: (126 MHz, $CDCl_3$)

22.0, 23.6, 24.9, 26.2, 44.0, 50.0, 69.9, 103.8, 110.8, 120.5, 122.6, 123.5, 128.6, 128.8, 128.8, 133.6, 136.9, 154.8, 156.6, 204.1.

IR v_{max}: (neat)

3440 (w, br), 2956 (m), 2931 (w), 2870 (w), 1671 (s), 1596 (m), 1252 (s).

HRMS: (*m/z,* ESI$^+$)

Requires: 337.1798 ($C_{22}H_{25}O_3$, $[M + H]^+$), Found: 337.1797 (+0.36 ppm)

2-Benzyl-1-cyclopropyl-3-hydroxy-5-methylhexan-1-one (3q)

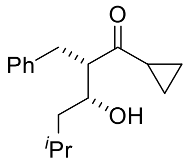

3q

Following General Procedure B, (*E*)-1-cyclopropyl-3-phenylprop-2-en-1-one (90 mg, 0.50 mmol, 1.0 equiv.), isovaleraldehyde (0.16 mL, 1.5 mmol, 3 equiv.), [H-*B*-9-BBN]$_2$ (5 mg, 0.02 mmol, 4 mol%), HBpin (0.22 mL, 1.5 mmol, 3 equiv.) and THF (4 mL, 0.125 M) gave crude product (>95:5 *d.r.*) that was purified by flash column chromatography (hexane:ethyl acetate, 99:1 to 90:10) to give the β-hydroxy ketone product **3q** as an amorphous white solid (100 mg, 0.40 mmol, 79%, >95:5 *d.r.*).

^{1}H NMR: (500 MHz, $CDCl_3$)

0.67 – 0.72 (m, 1H), 0.83 – 0.91 (m, 2H), 1.00 (d, *J* = 6.6 Hz, 3H), 1.02 – 1.06 (m, 4H), 1.29 – 1.34 (m, 1H), 1.61 – 1.65 (m, 1H), 1.69 – 1.73 (m, 1H), 1.86 – 1.94 (m, 1H), 2.73 (d, *J* = 3.3 Hz, 1H), 3.01 – 3.07 (m, 2H), 3.09 – 3.14 (m, 1H), 4.04 – 4.08 (m, 1H), 7.24 – 7.28 (m, 3H), 7.33 – 7.36 (m, 2H).

^{13}C{^{1}H} NMR: (126 MHz, $CDCl_3$)

11.7, 11.8, 22.0, 22.4, 23.7, 24.9, 33.1, 43.7, 60.0, 69.8, 126.4, 128.6, 129.1, 140.2, 215.5.

IR ν_{max}: (neat)

3418 (w, br), 2989 (m), 2961 (m), 2912 (m), 1869 (m), 1685 (s).

HRMS: (*m/z,* ESI$^+$)

Requires: 261.1849 ($C_{17}H_{25}O_2$, [M + H]$^+$), Found: 261.1846 (−1.19 ppm)

4-Benzyl-5-hydroxy-2,2,7-trimethyloctan-3-one (3r)

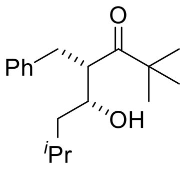

3r

Following General Procedure B, (1*E*)-4,4-dimethyl-1-phenyl-1-penten-3-one (95 mg, 0.50 mmol, 1.0 equiv.), isovaleraldehyde (0.16 mL, 1.5 mmol, 3 equiv.), [H-*B*-9-BBN]$_2$ (5 mg, 0.02 mmol, 4 mol%), HBpin (0.22 mL, 1.5 mmol, 3 equiv.) and THF (4 mL, 0.125 M) gave crude product (>95:5 *d.r.*) that was purified by flash column chromatography (hexane:ethyl acetate, 99:1 to 90:10) to give the β-hydroxy ketone product **3r** as a colourless oil (63 mg, 0.23 mmol, 46%, >95:5 *d.r.*).

^{1}H NMR: (500 MHz, $CDCl_3$)

0.79 (s, 9H), 0.93 (d, *J* = 6.6 Hz, 3H), 0.96 (d, *J* = 6.6 Hz, 3H), 1.23 – 1.28 (m, 1H), 1.63 – 1.69 (m, 1H), 1.76 – 1.86 (m, 1H), 2.87 (dd, *J* = 13.4, 3.9 Hz, 1H), 2.92 (br. s, 1H), 2.99 (dd, *J* = 13.4, 10.7 Hz, 1H), 3.27 (ddd, *J* = 10.7, 4.0, 1.8 Hz, 1H), 3.79 (ddd, *J* = 8.7, 4.4, 1.9 Hz, 1H), 7.09 – 7.10 (m, 2H), 7.14 – 7.17 (m, 1H), 7.21 – 7.24 (m, 2H).

^{13}C{^{1}H} NMR: (126 MHz, $CDCl_3$)

22.3, 23.5, 25.0, 25.7, 32.5, 43.8, 52.8, 69.5, 126.5, 128.5, 129.5, 140.1, 220.9.

IR v_{max}: (neat)

3532 (w, br), 3956 (m), 2933 (m), 2869 (w), 1684 (s).

HRMS: (*m/z,* ESI$^+$)

Requires: 277.2162 ($C_{18}H_{29}O_2$, $[M + H]^+$), Found: 277.2161 (−0.40 ppm)

2-Ethyl-3-hydroxy-1-(2,6,6-trimethylcyclohex-1-en-1-yl)octan-1-one (3s)

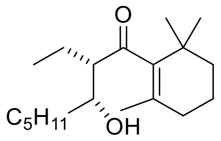

3s

Following General Procedure B, (*E*)-β-damascone (0.10 mL, 0.50 mmol, 1.0 equiv.), hexanal (0.18 mL, 1.5 mmol, 3 equiv.), [H-*B*-9-BBN]$_2$ (5 mg, 0.02 mmol, 4 mol%), HBpin (0.22 mL, 1.5 mmol, 3 equiv.) and THF (4 mL, 0.125 M) gave crude product (>95:5 *d.r.*) that was purified by flash column chromatography (hexane:ethyl acetate, 99:1 to 90:10) to give the β-hydroxy ketone product **3s** as a colourless oil (130 mg, 0.44 mmol, 88%, >95:5 *d.r.*).

^{1}H NMR: (500 MHz, $CDCl_3$)

0.88 – 0.91 (m, 3H), 1.01 (t, *J* = 7.5 Hz, 3H), 1.07 (s, 3H), 1.16 (s, 3H), 1.29 – 1.35 (m, 5H), 1.43 – 1.45 (m, 3H), 1.49 – 1.53 (m, 1H), 1.61 (s, 3H), 1.63 – 1.73 (m, 4H), 1.74 – 1.82 (m, 1H), 2.02 – 2.04 (m, 2H), 2.67 (td, *J* = 6.1, 2.3 Hz, 1H), 2.83 (d, *J* = 4.6 Hz, 1H), 3.97 (dtd, *J* = 9.1, 4.7, 2.3 Hz, 1H)

^{13}C{^{1}H} NMR: (126 MHz, $CDCl_3$)

13.7, 14.2, 18.1, 18.7, 21.7, 22.8, 26.3, 29.2, 29.3, 31.9, 32.0, 33.2, 34.5, 40.1, 58.1, 71.2, 134.0, 142.9, 215.5.

IR v_{max}: (neat)

3488 (w, br), 2956 (m), 2932 (m), 2872 (w), 1664 (m), 1459 (m).

HRMS: (*m/z,* ESI$^+$)

Requires: 295.2632 ($C_{19}H_{35}O_2$, [M + H]$^+$), Found: 295.2624 (−2.57 ppm)

(3S,8R,9S,10R,13S,14S,17S)-17-Acetyl-17-((S)-1-hydroxy-3-methylbutyl)-10,13-dimethyl-2,3,4,7,8,9,10,11,12,13,14,15,16,17-tetradecahydro-1H-cyclopenta[a]-phenanthren-3-yl acetate (3t)

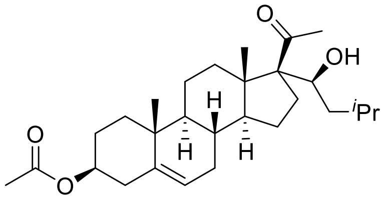

3t

Following General Procedure B, 16-dehydropregnenolone acetate (180 mg, 0.50 mmol, 1.0 equiv.), isovaleraldehyde (0.16 mL, 1.5 mmol, 3 equiv.), [H-*B*-9-BBN]$_2$ (5 mg, 0.02 mmol, 4 mol%), HBpin (0.22 mL, 1.5 mmol, 3 equiv.) and THF (4 mL, 0.125 M) gave crude product that was purified by flash column chromatography (hexane:ethyl acetate, 99:1 to 90:10) to give the title compound **3t** as an amorphous white solid (120 mg, 0.27 mmol, 53%). The product was isolated as a single diastereomer. The product was crystalised in minimal Et_2O to obtain colourless, block-shaped crystals for X-ray analysis. The absolute configuration of product **3t** was supported by single crystal X-ray crystallography.

^{1}H NMR: (500 MHz, $CDCl_3$)

0.70 (s, 3H), 0.92 (m, 6H), 0.97 – 1.02 (m, 4H), 1.05 – 1.15 (m, 2H), 1.24 – 1.36 (m, 2H), 1.46 – 1.54 (m, 4H), 1.55 – 1.62 (m, 3H), 1.68 – 1.74 (m, 1H), 1.83 – 1.86 (m, 3H), 1.92 – 1.99 (m, 2H), 2.00 – 2.06 (m, 4H), 2.24 (s, 3H), 2.28 – 2.35 (m, 2H), 2.38 – 2.44 (m, 1H), 2.67 (d, *J* = 8.4 Hz, 1H), 3.82 – 3.86 (m, 1H), 4.58 – 4.62 (m, 1H), 5.36 – 5.37 (m, 1H).

^{13}C{^{1}H} NMR: (126 MHz, $CDCl_3$)

16.7, 19.4, 20.9, 21.5, 21.6, 24.2, 25.1, 25.2, 27.8, 28.2, 32.2, 32.4, 32.5, 34.6, 36.7, 37.1, 38.2, 44.1, 47.6, 49.5, 52.0, 68.8, 72.8, 74.0, 122.3, 139.9, 170.6, 216.0.

IR v_{max}: (neat)

3411 (w, br), 2956 (m), 2903 (m), 2825 (w), 1732 (s), 1675 (s), 1238 (s), 1031 (s).

HRMS: (*m/z,* ESI$^+$)

Requires: 445.3312 ($C_{28}H_{45}O_4$, $[M + H]^+$), Found: 445.3305 (−1.66 ppm)

$[\alpha]_D^{22}$: (*c* 1.00, $CHCl_3$)

−93.0

mp: (Et_2O)

139 – 140 °C

(2-Hydroxycyclohexyl)(phenyl)methanone (6)

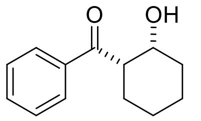

6

Following General Procedure B, (*E*)-7-oxo-7-phenylhept-5-enal (100 mg, 0.50 mmol, 1.0 equiv.), $[H\text{-}B\text{-}9\text{-}BBN]_2$ (5 mg, 0.02 mmol, 4 mol%), HBpin (0.22 mL, 1.5 mmol, 3 equiv.) and THF (4 mL, 0.125 M) gave crude product (>95:5 *d.r.*) that was purified by flash column chromatography (hexane:ethyl acetate, 99:1 to 90:10) to give the β-hydroxy ketone product **6** as a colourless oil (22 mg, 0.11 mmol, 22%, >95:5 *d.r.*).

^{1}H NMR: (500 MHz, $CDCl_3$)

1.39 - 1.48 (m, 2H), 1.72 – 2.02 (m, 6H), 3.36 (ddd, *J* = 12.1, 3.6, 2.0 Hz, 1H), 3.91 – 3.92 (m, 1H), 4.29 (br s, 1H), 7.47 – 7.50 (m, 2H), 7.57 – 7.61 (m, 1H), 7.92 – 7.94 (m, 2H).

^{13}C{^{1}H} NMR: (126 MHz, $CDCl_3$)

19.8, 24.8, 25.8, 32.1, 48.4, 66.6, 128.6, 128.9, 133.6, 136.0, 206.2.

Spectroscopic data were in accordance with those previously reported.[14]

(2-Hydroxy-2-methylcyclohexyl)(phenyl)methanone (8)

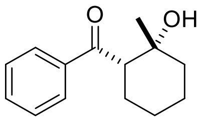

8

Following General Procedure B, (2*E*)-1-phenyl-2-octene-1,7-dione (110 mg, 0.50 mmol, 1.0 equiv.), $[H\text{-}B\text{-}9\text{-}BBN]_2$ (5 mg, 0.02 mmol, 4 mol%), HBpin (0.22 mL, 1.5 mmol, 3 equiv.) and THF (4 mL, 0.125 M) gave crude product (>95:5 *d.r.*) that was purified by flash column chromatography (hexane:ethyl acetate, 99:1 to 90:10) to give the β-hydroxy ketone product **8** as a colourless oil (22 mg, 0.10 mmol, 20%, >95:5 *d.r.*).

^{1}H NMR: (500 MHz, $CDCl_3$)

1.19 (s, 3H), 1.31 – 1.35 (m, 2H), 1.51 – 1.57 (m, 1H), 1.69 – 1.86 (m, 5H), 3.31 (dd, *J* = 12.2, 3.6 Hz, 1H), 4.46 (s, 1H), 7.47 – 7.51 (m, 2H), 7.58 – 7.62 (m, 1H), 7.94 – 7.96 (m, 2H).

^{13}C{^{1}H} NMR: (126 MHz, $CDCl_3$)

21.4, 25.7, 27.2, 29.8, 39.1, 51.6, 70.3, 128.4, 129.0, 133.8, 136.8, 207.3.

Spectroscopic data were in accordance with those previously reported.[15]

2-Benzyl-3-hydroxy-3-methyl-1-phenylbutan-1-one (10)

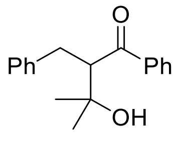

10

Following General Procedure B, (*E*)-chalcone (100 mg, 0.50 mmol, 1.0 equiv.), acetone (0.11 mL, 1.5 mmol, 3 equiv.), $[H\text{-}B\text{-}9\text{-}BBN]_2$ (5 mg, 0.02 mmol, 4 mol%), HBpin (0.22 mL, 1.5 mmol, 3 equiv.) and THF (4 mL, 0.125 M) gave crude product that was purified by flash column chromatography (hexane:ethyl acetate, 99:1 to 90:10) to give the β-hydroxy ketone product **10** as an amorphous white solid (110 mg, 0.43 mmol, 85%).

^{1}H NMR: (500 MHz, $CDCl_3$)

1.23 (s, 3H), 1.38 (s, 3H), 3.14 – 3.20 (m, 2H), 3.36 (s, 1H), 3.79 (dd, *J* = 8.9, 5.9 Hz, 1H), 7.02 – 7.05 (m, 1H), 7.08 – 7.13 (m, 4H), 7.26 – 7.29 (m, 2H), 7.41 – 7.45 (m, 1H), 7.57 – 7.59 (m, 2H).

^{13}C{^{1}H} NMR: (126 MHz, $CDCl_3$)

27.9, 29.8, 35.5, 57.0, 72.5, 126.3, 128.3, 128.5, 128.6, 129.1, 133.2, 138.9, 139.6, 207.7.

IR ν_{max}: (neat)

3501 (m, br), 2988 (m), 2968 (m), 2901 (m), 1655 (s).

HRMS: (*m/z,* ESI^+)

Requires: 269.1536 ($C_{18}H_{21}O_2$, $[M + H]^+$), Found: 269.1531 (−1.89 ppm)

7. Unsuccessful Substrates

α,β-Unsaturated Ketones

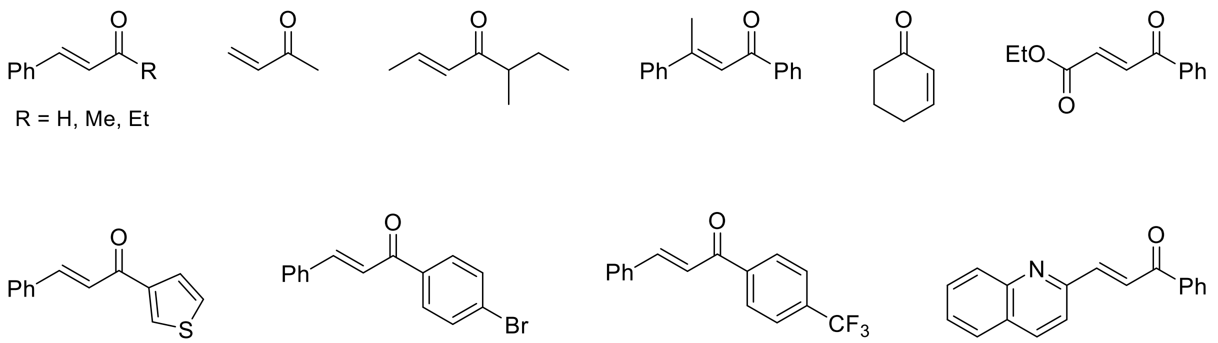

Aldehydes

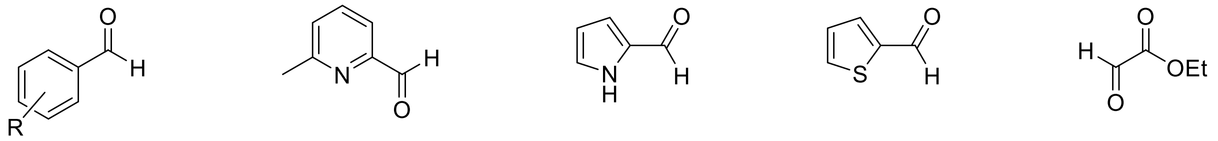

8. Enantioenriched Substrate Scope

(2*S*,3*R*)-2-Benzyl-3-hydroxy-5-methyl-1-phenylhexan-1-one (3a)

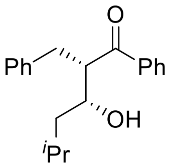

(2*S*,3*R*)-3a

Following General Procedure C, (*E*)-chalcone (100 mg, 0.50 mmol, 1.0 equiv.), isovaleraldehyde (0.16 mL, 1.5 mmol, 3 equiv.), (+)-[HB(Lgf)$_2$]$_2$ (42 mg, 0.050 mmol, 10 mol%), HBpin (0.22 mL, 1.5 mmol, 3 equiv.) and THF (4 mL, 0.125 M) gave crude product (>95:5 *d.r.*) that was purified by flash column chromatography (hexane:ethyl acetate, 99:1 to 90:10) to give the β-hydroxy ketone product **(2*S*,3*R*)-3a** as an amorphous white solid (140 mg, 0.48 mmol, 96%, >95:5 *d.r.*, 90:10 *e.r.*). The product was crystalised in minimal Et$_2$O to obtain colourless, block-shaped crystals for X-ray analysis. The absolute configuration of **(2*S*,3*R*)-3a** was supported by single crystal X-ray crystallography. Since the crystal used to obtain the X-ray structure was grown from a mixture of enantiomers, the crystal used to obtain the X-ray structure was analysed by HPLC to confirm it was the major enantiomer.

Chiral HPLC: (CHIRALPAK IB, 95:5 *n*-hexane:*iso*propanol, flow rate 1.0 mL/min, 244 nm, 30 °C)

t_R **(2*S*,3*R*)-3a** 6.8 min, t_R **(2*R*,3*S*)-3a** 9.8 min, 90:10 *e.r.*

t_R of single crystal **(2*S*,3*R*)-3a** 6.7 min

$[\alpha]_D^{23}$: (*c* 1.05, $CHCl_3$)

−41.9

mp: (Et_2O)

93 - 95 °C

Spectroscopic data were in accordance with those reported for product **3a**.

(2*S*,3*R*)-2-Benzyl-3-cyclohexyl-3-hydroxy-1-phenylpropan-1-one (3e)

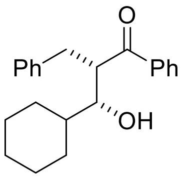

(2*S*,3*R*)-3e

Following General Procedure C, (*E*)-chalcone (100 mg, 0.50 mmol, 1.0 equiv.), cyclohexanecarboxaldehyde (0.18 mL, 1.5 mmol, 3 equiv.), (+)-$[HB(Lgf)_2]_2$ (42 mg, 0.050 mmol, 10 mol%), HBpin (0.22 mL, 1.5 mmol, 3 equiv.) and THF (4 mL, 0.125 M) gave crude product (>95:5 *d.r.*) that was purified by flash column chromatography (hexane:ethyl acetate, 99:1 to 90:10) to give the β-hydroxy ketone product **(2*S*,3*R*)-3e** as an amorphous white solid (90 mg, 0.27 mmol, 54%, >95:5 *d.r.*, 85:15 *e.r.*).

Chiral HPLC: (CHIRALPAK IC**,** 95:5 *n*-hexane:*iso*propanol, flow rate 1.0 mL/min, 244 nm, 30 °C)

t_R **(2*R*,3*S*)-3e** 10.8 min, t_R **(2*S*,3*R*)**-3e 12.7 min, 85:15 *e.r.*

$[\alpha]_D^{23}$**:** (*c* 1.03, $CHCl_3$)

−10.1

Spectroscopic data were in accordance with those reported for product **3e.**

(2*S*,3*S*)-2-Benzyl-3-hydroxy-4,4-dimethyl-1-phenylpentan-1-one (3f)

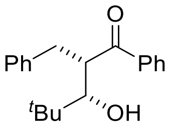

(2*S*,3*S*)-3f

Following General Procedure C, (*E*)-chalcone (100 mg, 0.50 mmol, 1.0 equiv.), trimethylacetaldehyde (0.16 mL, 1.5 mmol, 3 equiv.), (+)-$[HB(Lgf)_2]_2$ (42 mg, 0.050 mmol, 10 mol%), HBpin (0.22 mL, 1.5 mmol, 3 equiv.) and THF (4 mL, 0.125 M) gave crude product (74:26 *d.r.*) that was purified by flash column chromatography (hexane:ethyl acetate, 99:1 to 90:10) to give the β-hydroxy ketone product **(2*S*,3*S*)-3f** as an amorphous white solid (48 mg, 0.16 mmol, 32%, >95:5 *d.r.,* 86:14 *e.r.*).

Chiral HPLC: (CHIRALPAK IC, 95:5 *n*-hexane:isopropanol, flow rate 1.0 mL/min, 244 nm, 30 °C)

t_R **(2*R*,3*R*)-3f** 6.7 min, t_R **(2*S*,3*S*)-3f** 8.6 min, 86:14 *e.r.*

$[\alpha]_D^{23}$**:** (*c* 0.40, $CHCl_3$)

−44.0

Spectroscopic data were in accordance with those reported for product **3f.**

(2*S*,3*R*)-2-Benzyl-1-(4-fluorophenyl)-3-hydroxy-5-methylhexan-1-one (3i)

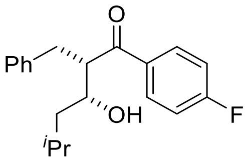

(2*S*,3*R*)-3i

Following General Procedure C, (*E*)-1-(4-fluorophenyl)-3-phenylprop-2-en-1-one (110 mg, 0.50 mmol, 1.0 equiv.), isovaleraldehyde (0.16 mL, 1.5 mmol, 3 equiv.), (+)-[HB(Lgf)$_2$]$_2$ (42 mg, 0.050 mmol, 10 mol%), HBpin (0.22 mL, 1.5 mmol, 3 equiv.) and THF (4 mL, 0.125 M) gave crude product (>95:5 *d.r.*) that was purified by flash column chromatography (hexane:ethyl acetate, 99:1 to 90:10) to give the β-hydroxy ketone product **(2*S*,3*R*)-3i** as an amorphous white solid (140 mg, 0.45 mmol, 89%, >95:5 *d.r.*, 85:15 *e.r.*).

Chiral HPLC: (CHIRALPAK IB**,** 95:5 *n*-hexane:isopropanol, flow rate 1.0 mL/min, 244 nm, 30 °C)

t_R **(2*S*,3*R*)-3i** 6.9 min, t_R **(2*R*,3*S*)-3i** 10.7 min, 85:15 *e.r.*

$[\alpha]_D^{23}$**:** (*c* 1.15, $CHCl_3$)

−41.0

Spectroscopic data were in accordance with those reported for product **3i.**

(2*S*,3*R*)-2-(2,4-Dimethoxy-6-methylbenzyl)-3-hydroxy-5-methyl-1-phenylhexan-1-one (3l)

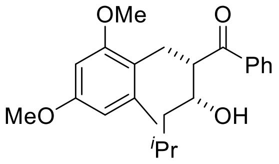

(2*S*,3*R*)-3l

Following General Procedure C, (*E*)-3-(2,4-dimethoxy-6-methylphenyl)-1-phenylprop-2-en-1-one (140 mg, 0.50 mmol, 1.0 equiv.), isovaleraldehyde (0.16 mL, 1.5 mmol, 3 equiv.), (+)-[HB(Lgf)$_2$]$_2$ (42 mg, 0.050 mmol, 10 mol%),HBpin (0.22 mL, 1.5 mmol, 3 equiv.) and THF (4 mL, 0.125 M) gave crude product (93:7 *d.r.*) that was purified by flash column chromatography (hexane:ethyl acetate, 99:1 to 90:10) to give the β-hydroxy ketone product **(2*S*,3*R*)-3l** as an amorphous white solid (161 mg, 0.44 mmol, 87%, >95:5 *d.r.*, 80:20 *e.r.*).

Chiral HPLC: (CHIRALPAK IC**,** 95:5 *n*-hexane:isopropanol, flow rate 1.0 mL/min, 244 nm, 30 °C)

t_R **(2*R*,3*S*)-3l** 12.0 min, t_R **(2*S*,3*R*)-3l** 19.3 min, 80:20 *e.r.*

$[\alpha]_D^{23}$ **:** (*c* 1.00, $CHCl_3$)

−75.2

Spectroscopic data were in accordance with those reported for product **3l.**

(2*S*,3*R*)-Methyl-4-(2-benzoyl-3-hydroxy-5-methylhexyl)benzoate (3n)

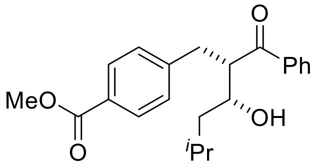

(2*S*,3*R*)-3n

Following General Procedure C, methyl-(*E*)-4-(3-oxo-3-phenylprop-1-en-1-yl)benzoate (130 mg, 0.50 mmol, 1.0 equiv.), isovaleraldehyde (0.16 mL, 1.5 mmol, 3 equiv.), (+)-$[HB(Lgf)_2]_2$ (42 mg, 0.050 mmol, 10 mol%),HBpin (0.22 mL, 1.5 mmol, 3 equiv.) and THF (4 mL, 0.125 M) gave crude product (93:7 *d.r.*) that was purified by flash column chromatography (hexane:ethyl acetate, 99:1 to 90:10) to give the β-hydroxy ketone product **(2*S*,3*R*)-3n** as a yellow oil (80 mg, 0.23 mmol, 45%, >95:5 *d.r.*, 84:16 *e.r.*).

Chiral HPLC: (CHIRALPAK IC**,** 95:5 *n*-hexane:isopropanol, flow rate 1.0 mL/min, 244 nm, 30 °C)

t_R **(2*R*,3*S*)-3n** 14.4 min, t_R **(2*S*,3*R*)-3n** 18.3 min, 84:16 *e.r.*

$[\alpha]_D^{23}$ **:** (*c* 0.45, $CHCl_3$)

+7.1

Spectroscopic data were in accordance with those reported for product **3n.**

(2*S*,3*R*)-2-Benzyl-1-cyclopropyl-3-hydroxy-5-methylhexan-1-one (3q)

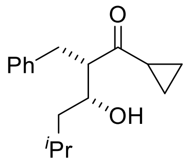

(2*S*,3*R*)-3q

Following General Procedure C, (*E*)-1-cyclopropyl-3-phenylprop-2-en-1-one (90 mg, 0.50 mmol, 1.0 equiv.), isovaleraldehyde (0.16 mL, 1.5 mmol, 3 equiv.), (+)-[HB(Lgf)$_2$]$_2$ (42 mg, 0.050 mmol, 10 mol%), HBpin (0.22 mL, 1.5 mmol, 3 equiv.) and THF (4 mL, 0.125 M) gave crude product (>95:5 *d.r.*) that was purified by flash column chromatography (hexane:ethyl acetate, 99:1 to 90:10) to give the β-hydroxy ketone product **(2*S*,3*R*)-3q** as an amorphous white solid (98 mg, 0.38 mmol, 75%, >95:5 *d.r.*, 80:20 *e.r.*).

Chiral HPLC: (CHIRALPAK IC**,** 95:5 *n*-hexane:isopropanol, flow rate 1.0 mL/min, 210 nm, 30 °C)

t_R **(2*R*,3*S*)-3q** 7.4 min, t_R **(2*S*,3*R*)-3q** 9.0 min, 80:20 *e.r.*

$[\alpha]_D^{23}$**:** (*c* 1.10, $CHCl_3$)

−40.7

Spectroscopic data were in accordance with those reported for product **3q.**

(2*S*,3*R*)-4-Benzyl-5-hydroxy-2,2,7-trimethyloctan-3-one (3r)

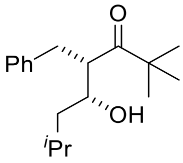

(2*S*,3*R*)-3r

Following General Procedure C, (1*E*)-4,4-dimethyl-1-phenyl-1-penten-3-one (95 mg, 0.50 mmol, 1.0 equiv.), isovaleraldehyde (0.16 mL, 1.5 mmol, 3 equiv.), (+)-[HB(Lgf)$_2$]$_2$ (42 mg, 0.050 mmol, 10 mol%), HBpin (0.22 mL, 1.5 mmol, 3 equiv.) and THF (4 mL, 0.125 M) gave crude product (>95:5 *d.r.*) that was purified by flash column chromatography (hexane:ethyl acetate, 99:1 to 90:10) to give the β-hydroxy ketone product **(2*S*,3*R*)-3r** as a colourless oil (96 mg, 0.44 mmol, 87%, >95:5 *d.r.*, 72:28 *e.r.*).

Chiral HPLC: (CHIRALPAK IB**,** 95:5 *n*-hexane:isopropanol, flow rate 1.0 mL/min, 251 nm, 30 °C)

t_R **(2*S*,3*R*)-3r** 8.3 min, t_R **(2*R*,3*S*)-3r** 10.7 min, 72:28 *e.r.*

$[\alpha]_D^{23}$ **:** (*c* 0.97, $CHCl_3$)

+13.2

Spectroscopic data were in accordance with those reported for product **3r.**

(2*R*,3*R*)-2-Ethyl-3-hydroxy-1-(2,6,6-trimethylcyclohex-1-en-1-yl)octan-1-one (3s)

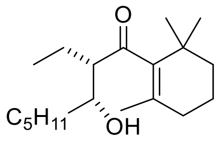

(2*R*,3*R*)-3s

Following General Procedure C, (*E*)-β-damascone (0.10 mL, 0.50 mmol, 1.0 equiv.), hexanal (0.18 mL, 1.5 mmol, 3 equiv.), (+)-$[HB(Lgf)_2]_2$ (42 mg, 0.050 mmol, 10 mol%), HBpin (0.22 mL, 1.5 mmol, 3 equiv.) and THF (4 mL, 0.125 M) gave crude product (>95:5 *d.r.*) that was purified by flash column chromatography (hexane:ethyl acetate, 99:1 to 90:10) to give the β-hydroxy ketone product **(2*R*,3*R*)-3s** as a colourless oil (58 mg, 0.20 mmol, 40%, >95:5 *d.r.*, 80:20 *e.r.*).

Chiral HPLC: (CHIRALPAK IG, 99:1 *n*-hexane:isopropanol, flow rate 1.0 mL/min, 244 nm, 30 °C)

t_R **(2*S*,3*S*)-3s** 17.2 min, t_R **(2*R*,3*R*)-3s** 18.5 min, 80:20 *e.r.*

$[\alpha]_D^{23}$**:** (*c* 1.08, $CHCl_3$)

−20.7

Spectroscopic data were in accordance with those reported for product **3s.**

9. Proposed Mechanism

The proposed mechanism is based on previous work in the Thomas group.[1, 13] The mechanism for the hydrolysis of the *O*-Bpin bond was reported by Brown.[16]

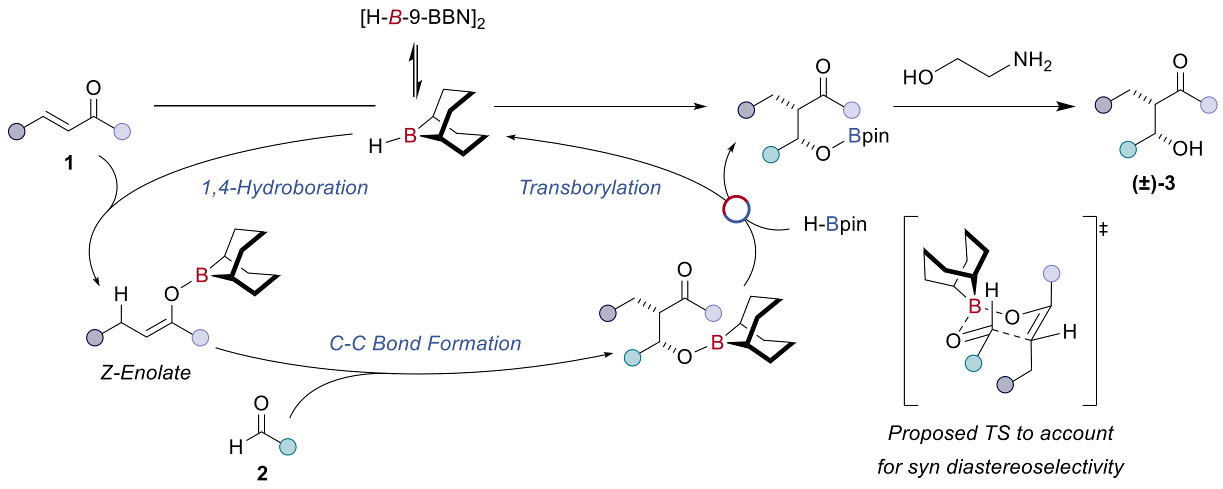

Proposed TS to account for syn diastereoselectivity

10. References

(1) Nicholson, K.; Langer, T.; Thomas, S. P. Borane-Catalyzed, Chemoselective Reduction and Hydrofunctionalization of Enones Enabled by B–O Transborylation. *Org. Lett.* **2021**, *23* (7), 2498-2504.

(2) Eastman, H.; Ryan, J.; Maciá, B.; Caprio, V.; O'Reilly, E. Alcohol Dehydrogenase-Triggered Oxa-Michael Reaction for the Asymmetric Synthesis of Disubstituted Tetrahydropyrans and Tetrahydrofurans. *ChemCatChem* **2019**, *11* (16), 3760-3762.

(3) Wang, D.; Zhang, Y.; Harris, A.; Gautam, L. N. S.; Chen, Y.; Shi, X. Triazole-Gold-Promoted, Effective Synthesis of Enones from Propargylic Esters and Alcohols: A Catalyst Offering Chemoselectivity, Acidity and Ligand Economy. *Adv. Synth. Catal.* **2011**, *353* (14-15), 2584-2588.

(4) Thiot, C.; Mioskowski, C.; Wagner, A. Sequential Hiyama Coupling/Narasaka Acylation Reaction of (E)-1,2-Disilylethene: Rapid Assembly of α,β-Unsaturated Carbonyl Motifs. *Eur. J. Org. Chem.* **2009**, *2009* (19), 3219-3227.

(5) Medici, F.; Resta, S.; Presenti, P.; Caruso, L.; Puglisi, A.; Raimondi, L.; Rossi, S.; Benaglia, M. Stereoselective Visible-Light Catalyzed Cyclization of Bis(enones): A Viable Approach to the Synthesis of Enantiomerically Enriched Cyclopentane Rings. *Eur. J. Org. Chem.* **2021**, *2021* (32), 4521-4524.

(6) Richards, E. L.; Murphy, P. J.; Dinon, F.; Fratucello, S.; Brown, P. M.; Gelbrich, T.; Hursthouse, M. B. Assessing the scope of the tandem Michael/intramolecular aldol reaction mediated by secondary amines, thiols and phosphines. *Tetrahedron* **2001**, *57* (36), 7771-7784.

(7) Gonzalez, A. Z.; Román, J. G.; Gonzalez, E.; Martinez, J.; Medina, J. R.; Matos, K.; Soderquist, J. A. 9-Borabicyclo[3.3.2]decanes and the Asymmetric Hydroboration of 1,1-Disubstituted Alkenes. *ACS Catal.* **2008**, *130* (29), 9218-9219. Nicholson, K.; Peng, Y.; Llopis, N.; Willcox, D. R.; Nichol, G. S.; Langer, T.; Baeza, A.; Thomas, S. P. Boron-Catalyzed, Diastereo- and Enantioselective Allylation of Ketones with Allenes. *ACS Catalysis* **2022**, *12* (17), 10887-10893.

(8) Brown, H. C.; Desai, M. C.; Jadhav, P. K. Hydroboration. 61. Diisopinocampheylborane of high optical purity. Improved preparation and asymmetric hydroboration of representative cis-disubstituted alkenes. *J. Org. Chem,* **1982**, *47* (26), 5065-5069.

(9) Jadhav, P. K.; Prasad, J. V. N. V.; Brown, H. C. A convenient method for upgrading the enantiomeric purities of (+)-longifolene and (+)-3-carene to materials approaching 100% ee. *J. Org. Chem,* **1985**, *50* (17), 3203-3206.

(10) Kanno, S.; Kakiuchi, F.; Kochi, T. Palladium-Catalyzed Hydroboration/Cyclization of 1,n-Dienes. *J. Org. Chem,* **2023**, *88* (4), 2621-2630.

(11) Macleod, J.; Thomas, S. P. Colorimetric indication of hidden catalysis. *Nat. Chem.* **2026**, *18* (1), 173-179.

(12) Jadhav, P. K.; Brown, H. C. Dilongifolylborane: a new effective chiral hydroborating agent with intermediate steric requirements. *J. Org. Chem.* **1981**, *46* (14), 2988-2990.

(13) Moreno González, A.; Nicholson, K.; Llopis, N.; Nichol, G. S.; Langer, T.; Baeza, A.; Thomas, S. P. Diastereoselective, Catalytic Access to Cross-Aldol Products Directly from Esters and Lactones. *Angew. Chem. Int. Ed.* **2022**, *61* (39), e202209584.

(14) Baik, T.-G.; Luis, A. L.; Wang, L.-C.; Krische, M. J. Diastereoselective Cobalt-Catalyzed Aldol and Michael Cycloreductions. *J. Am. Chem. Soc.* **2001**, *123* (21), 5112-5113.

(15) Huddleston, R. R.; Cauble, D. F.; Krische, M. J. Borane-Mediated Aldol Cycloreduction of Monoenone Monoketones: Diastereoselective Formation of Quaternary Centers. *J. Org. Chem.* **2003**, *68* (1), 11-14.

(16) Krishnamurthy, S.; Brown, H. C. Selective reductions. 22. Facile reduction of .alpha.,.beta.-unsaturated aldehydes and ketones with 9-borabicyclo[3.3.1]nonane. A remarkably convenient procedure for the selective conversion of conjugated aldehydes and ketones to the corresponding allylic alcohols in the presence of other functional groups. *J. Org. Chem,* **1977**, *42* (7), 1197-1201.

Appendix I: ^{1}H, $^{19}F\{^{1}H\}$ and $^{13}C\{^{1}H\}$ NMR Spectra

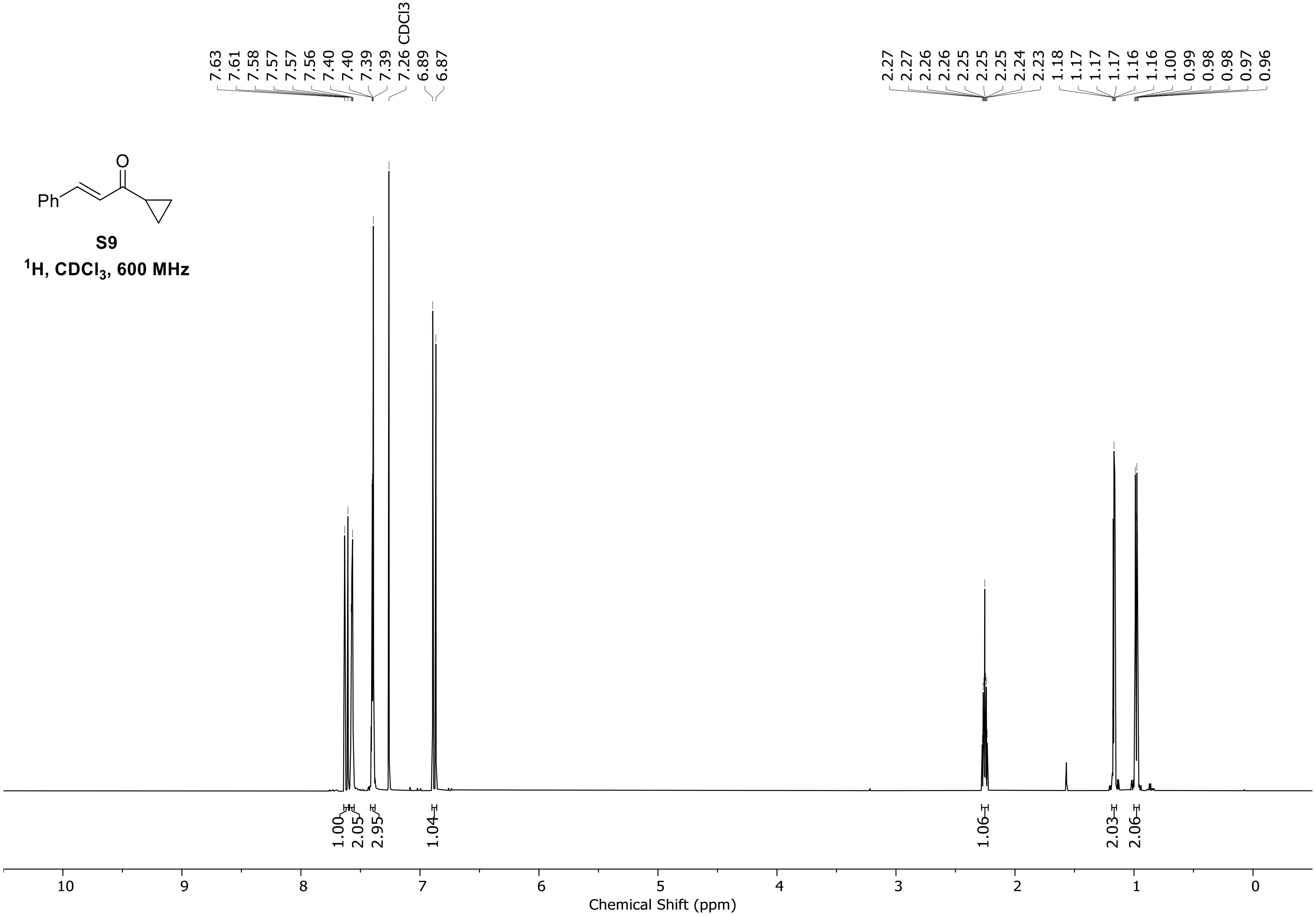
7.63 7.61 7.58 7.57 7.57 7.56 7.40 7.40 7.39 7.39 7.26 CDCl3 6.89 6.87
2.27 2.27 2.26 2.26 2.25 2.25 2.25 2.24 2.23 1.18 1.17 1.17 1.17 1.16 1.16 1.00 0.99 0.98 0.98 0.97 0.96
O
Ph
S9
1H, CDCl3, 600 MHz
1.00 2.05 2.95 1.04 1.06 2.03 2.06
10 9 8 7 6 5 4 3 2 1 0
Chemical Shift (ppm)

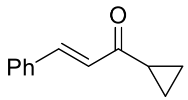

S9

$^{13}C\{^1H\}$, $CDCl_3$, 126 MHz

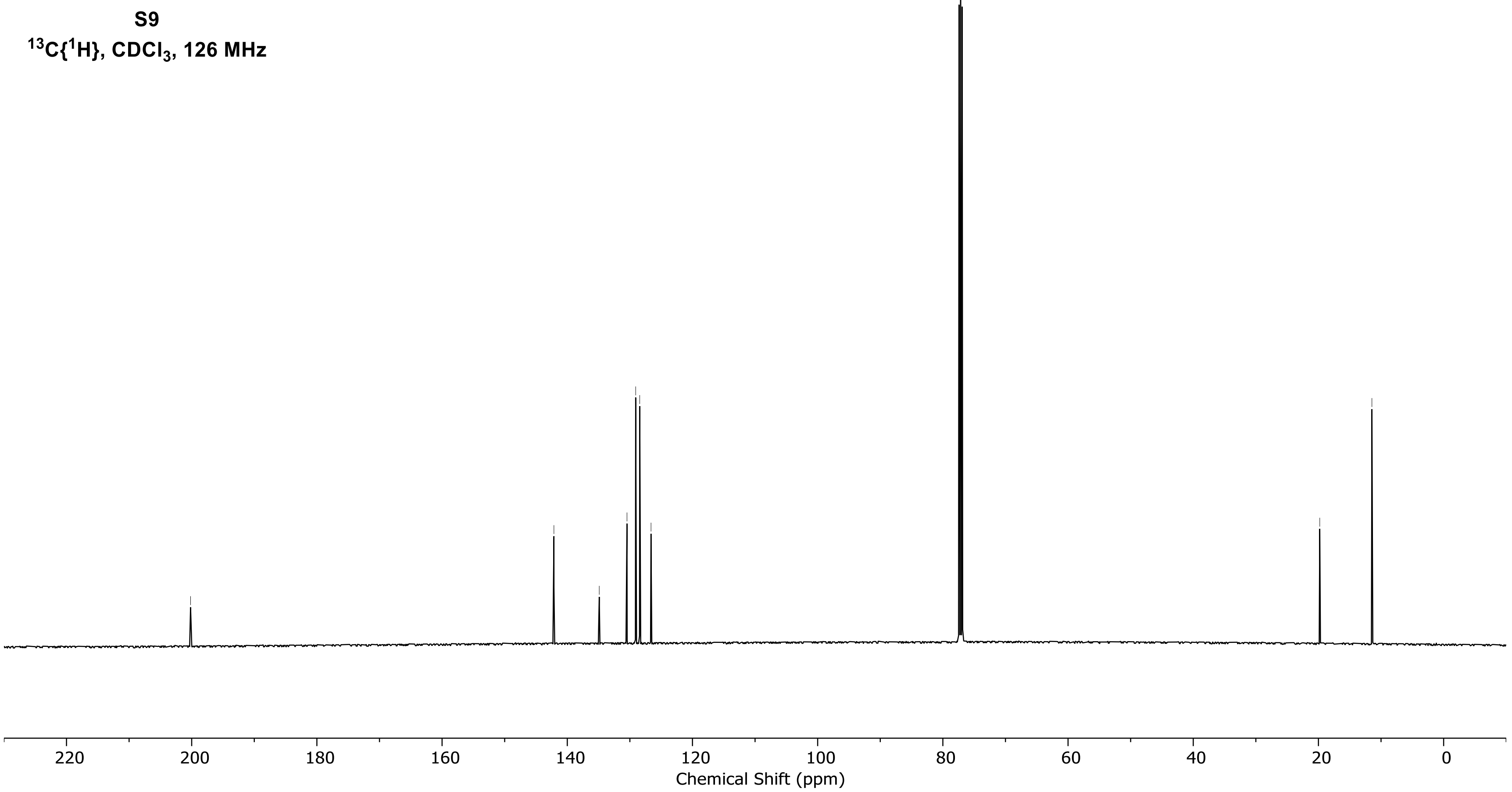

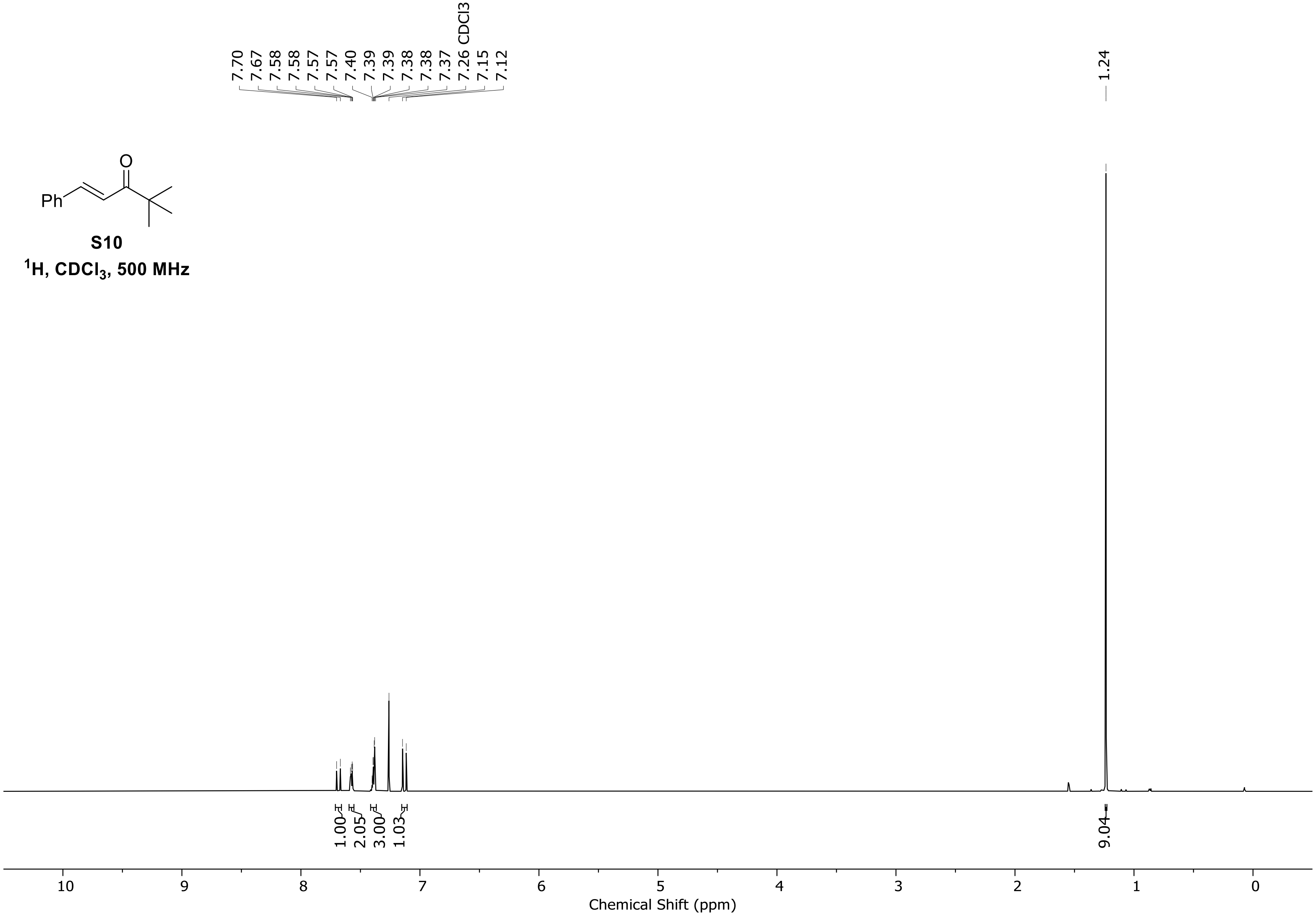

S10

^{1}H, $CDCl_3$, 500 MHz

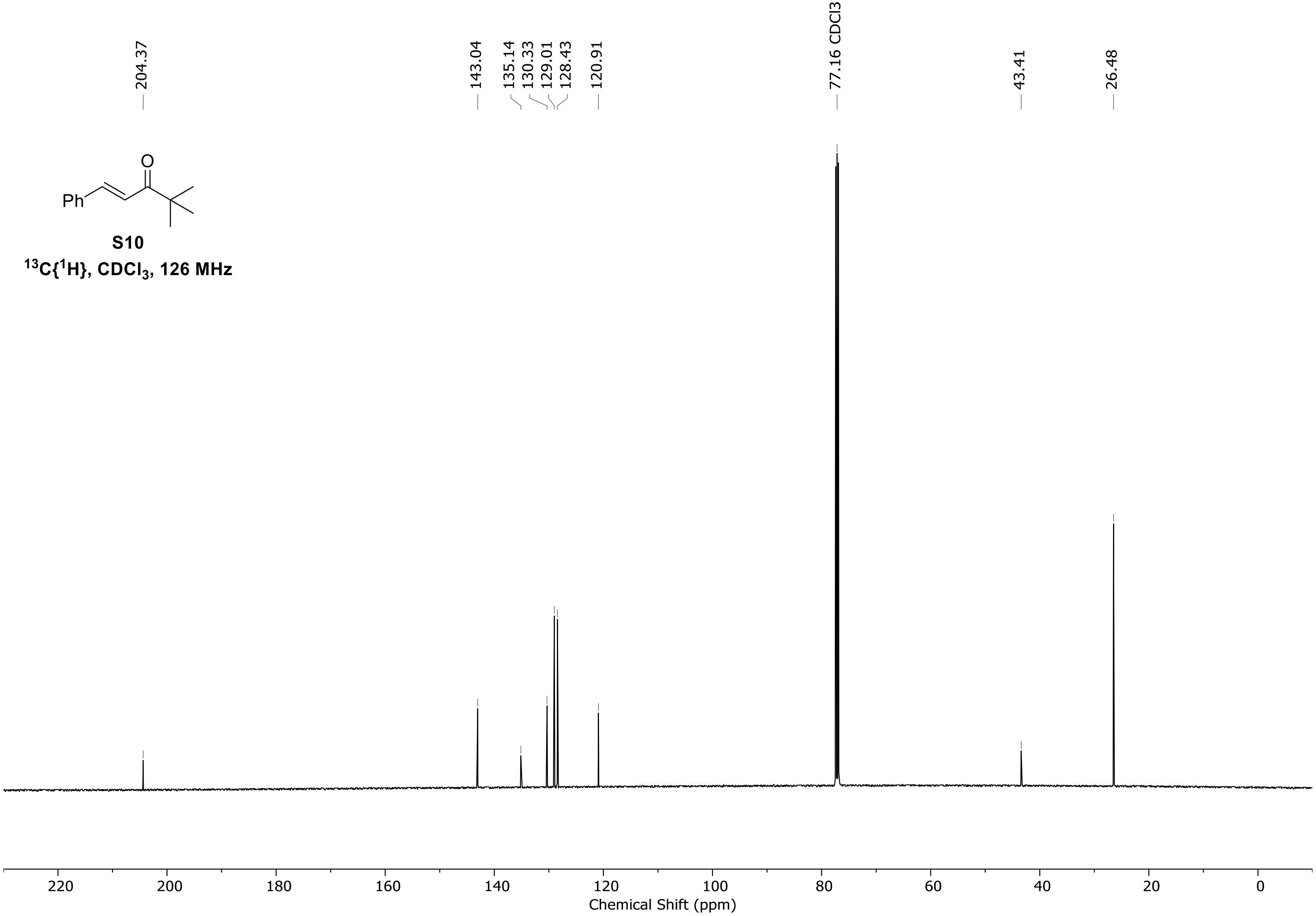

S10

$^{13}C\{^{1}H\}$, $CDCl_3$, 126 MHz

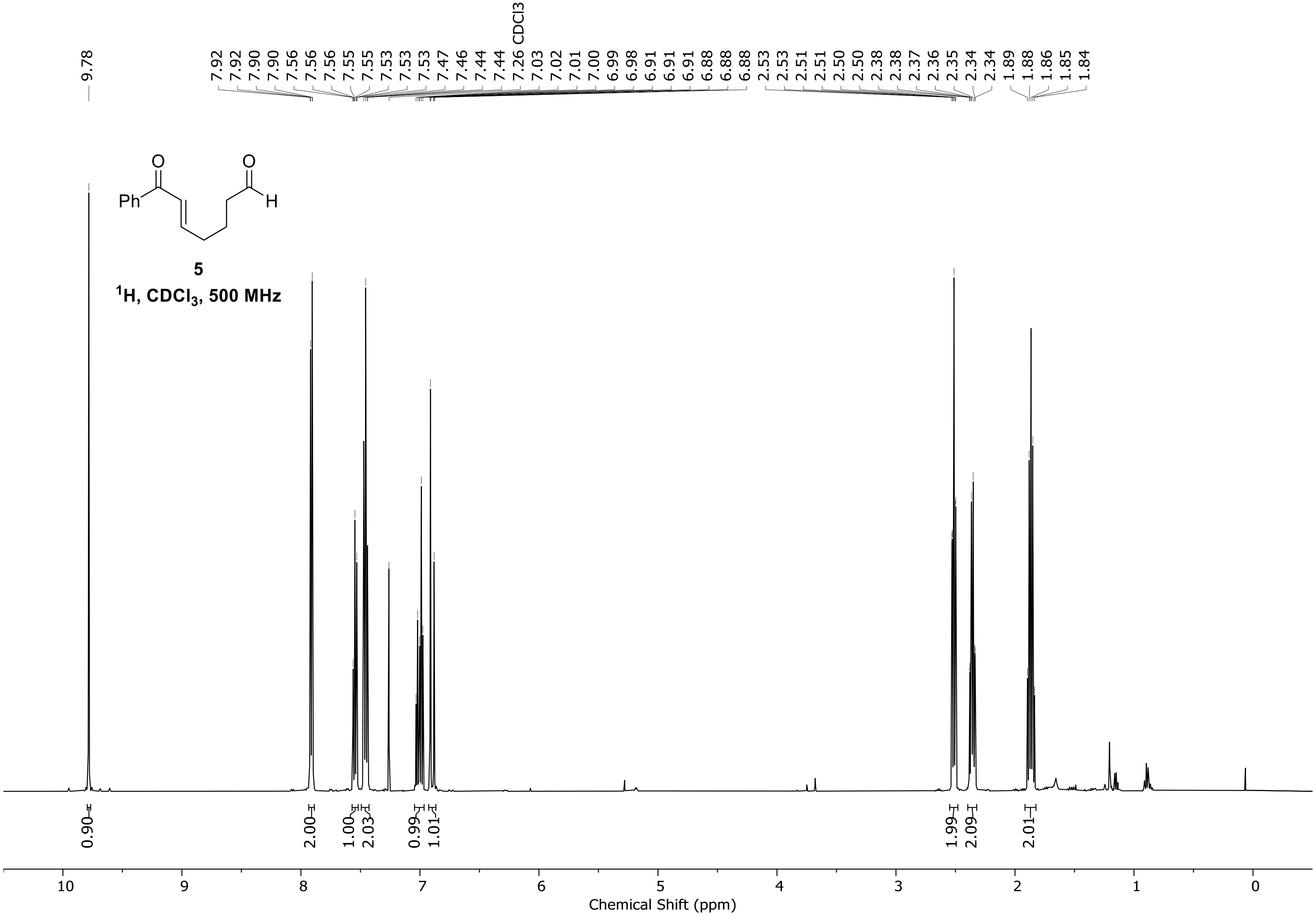
9.78
7.92 7.92 7.90 7.90 7.56 7.56 7.56 7.55 7.55 7.53 7.53 7.53 7.47 7.46 7.44 7.44 7.26 CDCl3 7.03 7.02 7.01 7.00 6.99 6.98 6.91 6.91 6.91 6.88 6.88 6.88
2.53 2.53 2.51 2.51 2.50 2.50 2.38 2.38 2.37 2.36 2.35 2.34 2.34 1.89 1.88 1.86 1.85 1.84
O
O
Ph
H
5
1H, CDCl3, 500 MHz
0.90
2.00
1.00
2.03
0.99
1.01
1.99
2.09
2.01
10
9
8
7
6
5
4
3
2
1
0
Chemical Shift (ppm)

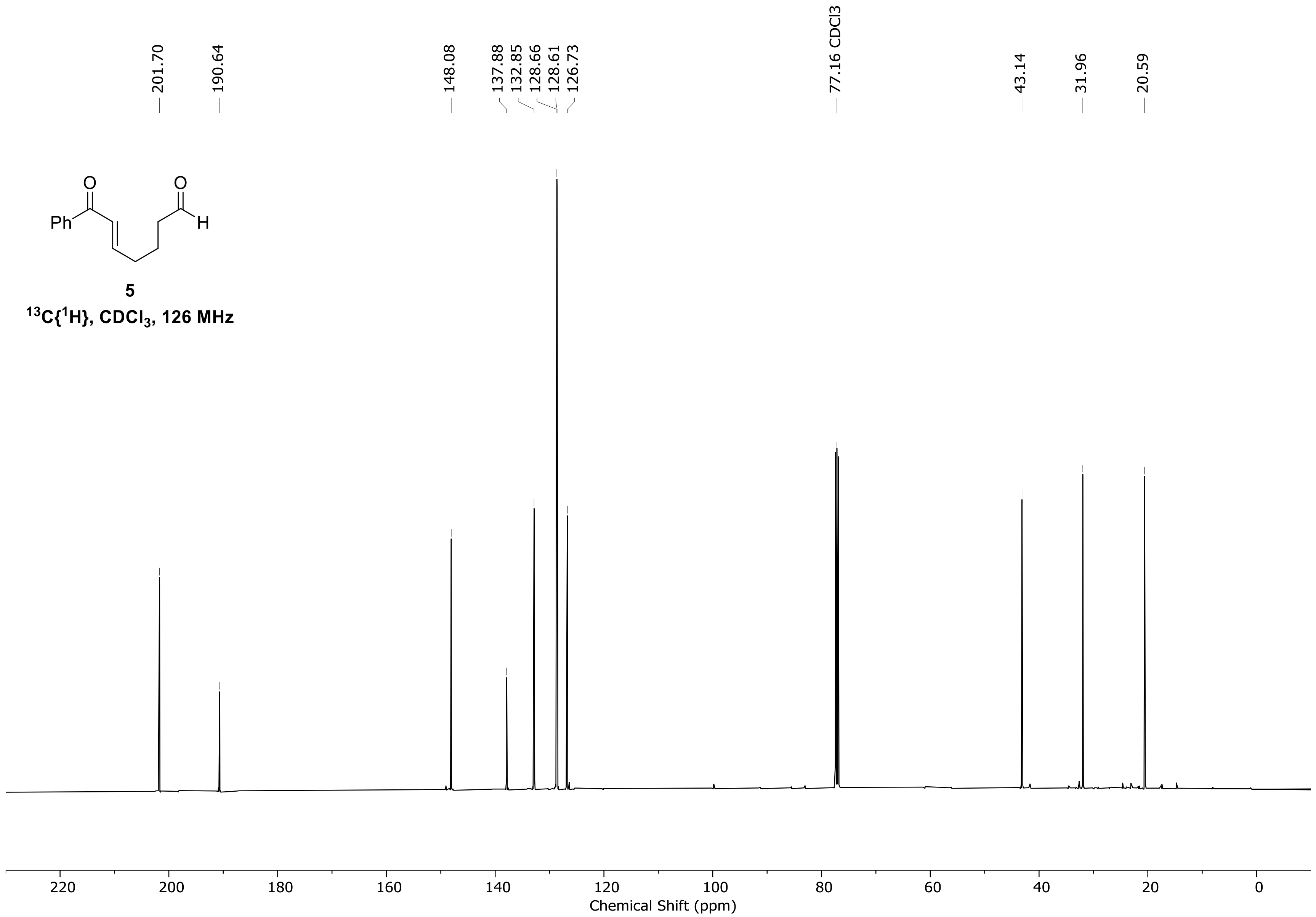

201.70
190.64
148.08
137.88
132.85
128.66
128.61
126.73
77.16 CDCl3
43.14
31.96
20.59
Ph
O
O
H
5
13C{1H}, CDCl3, 126 MHz
220
200
180
160
140
120
100
80
60
40
20
0
Chemical Shift (ppm)

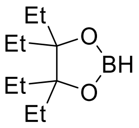

S16

^{1}H, $CDCl_3$, 500 MHz

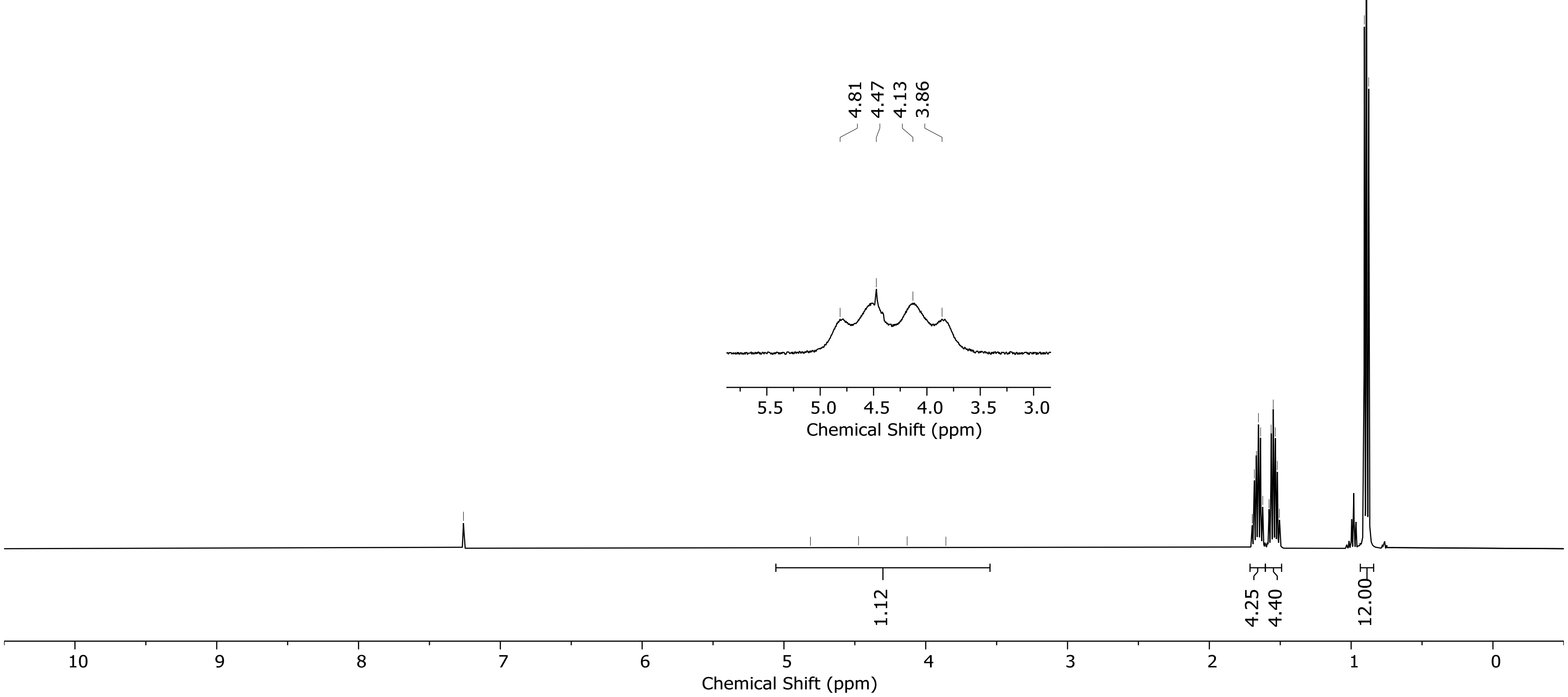

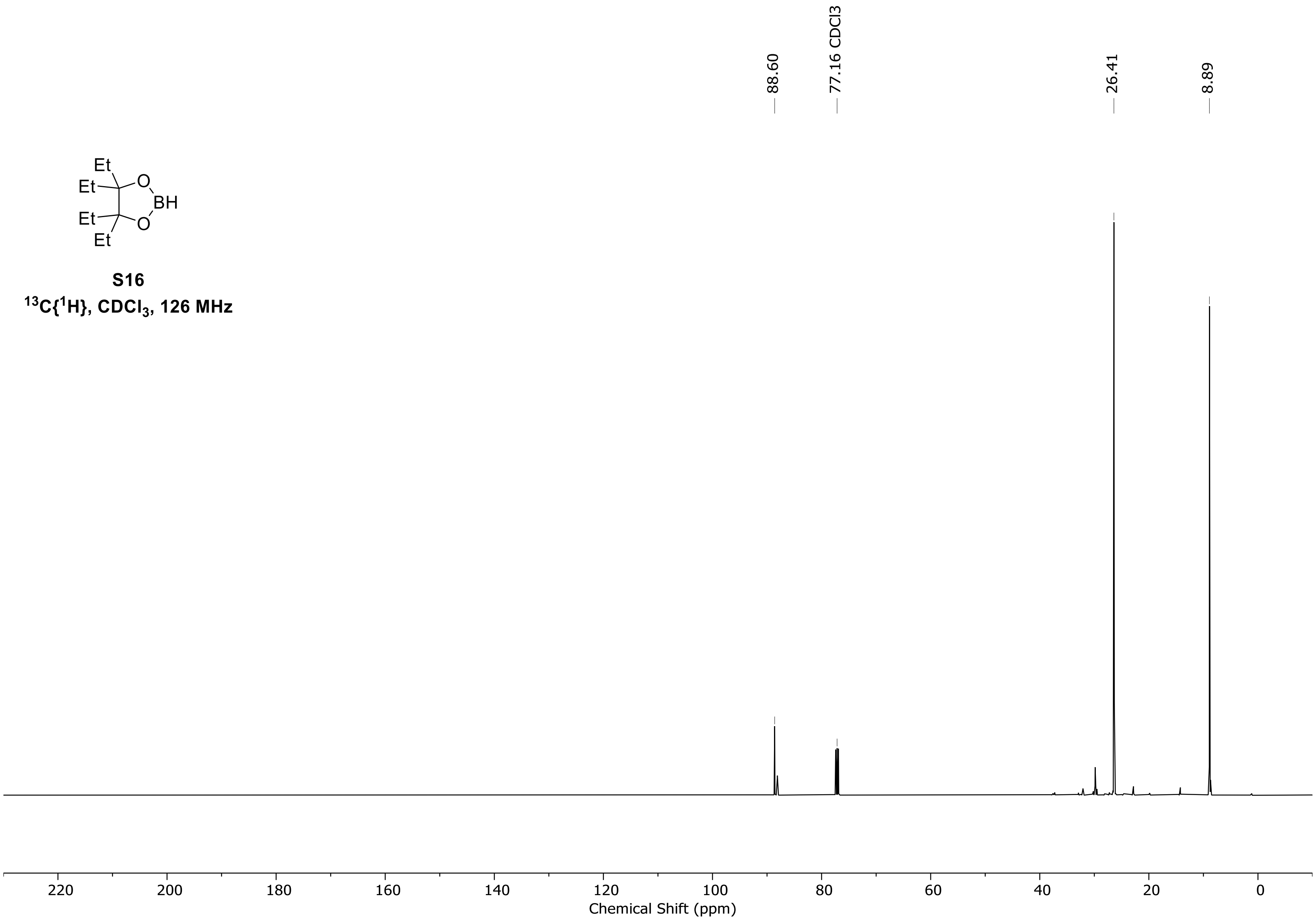

S16
$^{13}C\{^1H\}$, $CDCl_3$, 126 MHz

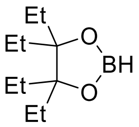

S16

^{11}B, C_6D_6, 128 MHz

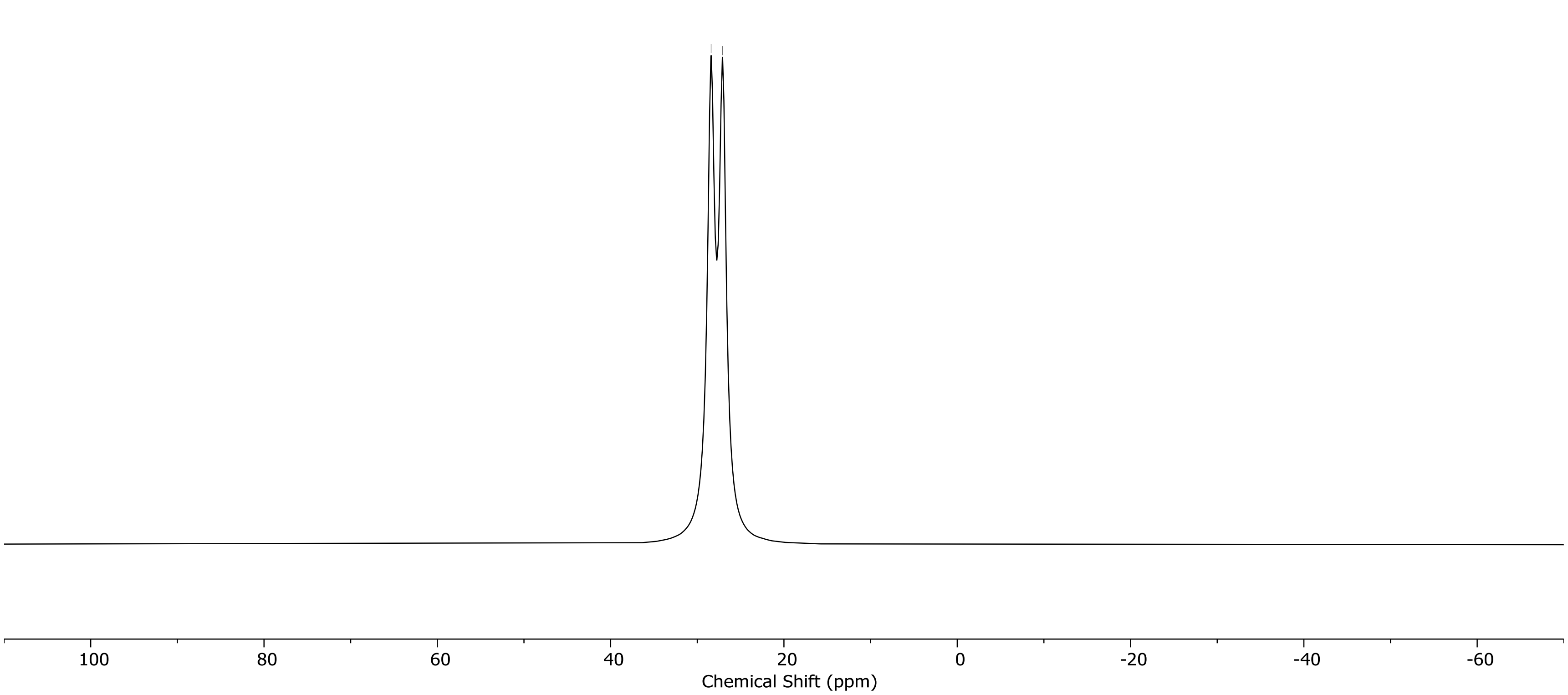

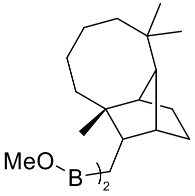

S18

^{1}H, CDCl$_3$, 500 MHz

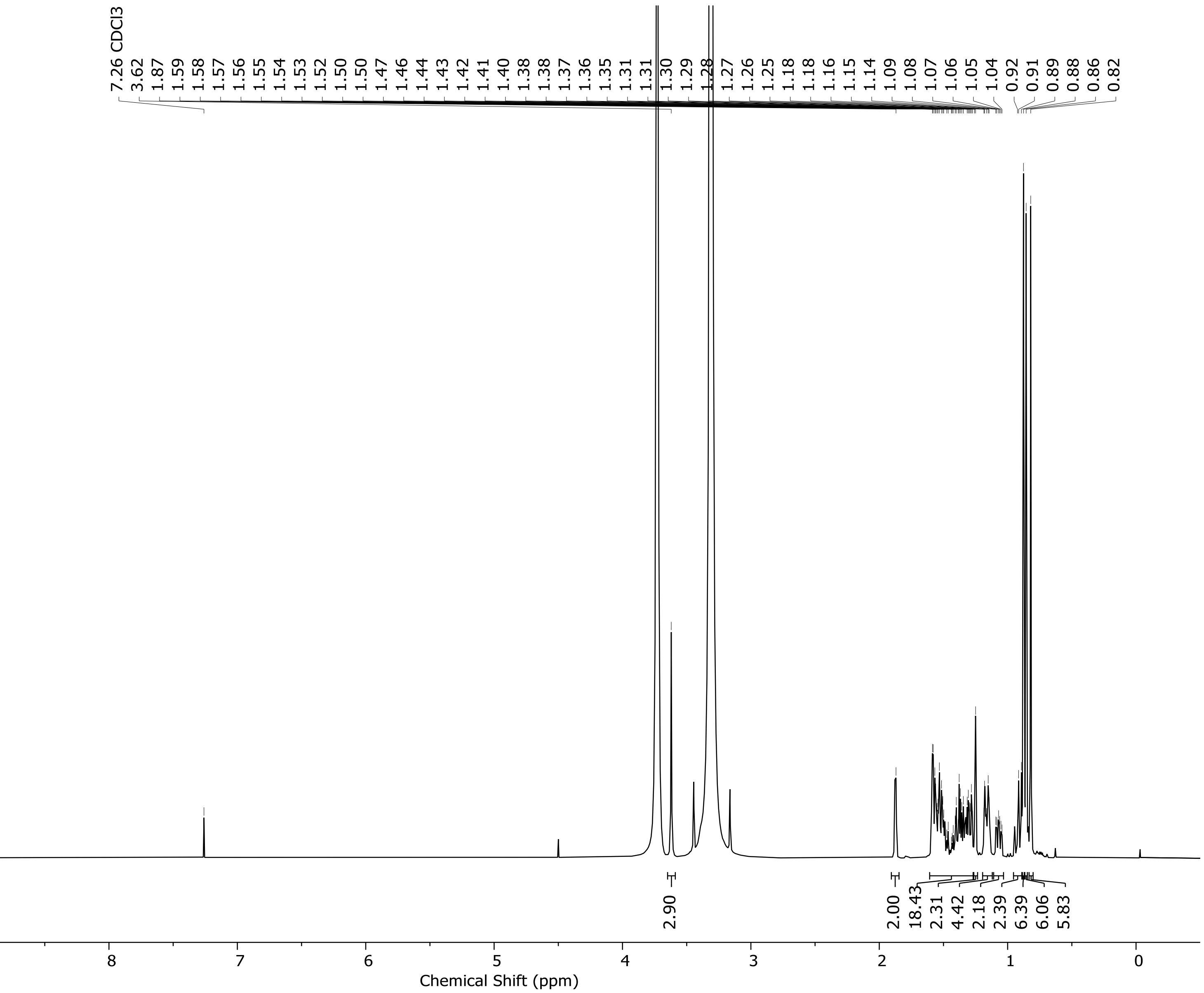

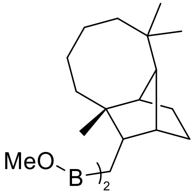

S18

$^{13}C\{^{1}H\}$, CDCl$_3$, 126 MHz

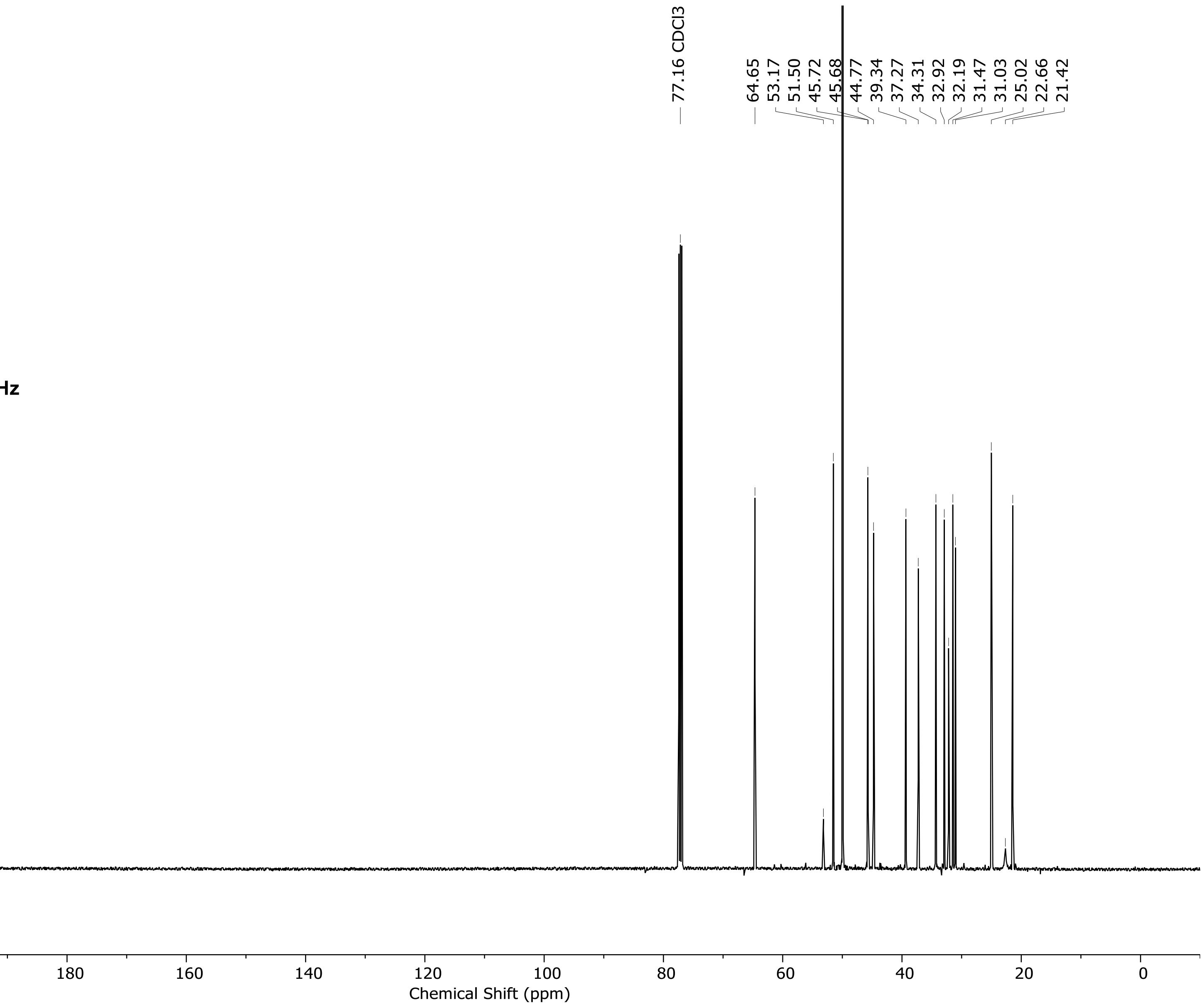

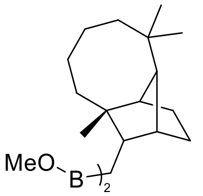

S18

^{11}B, THF, 160 MHz

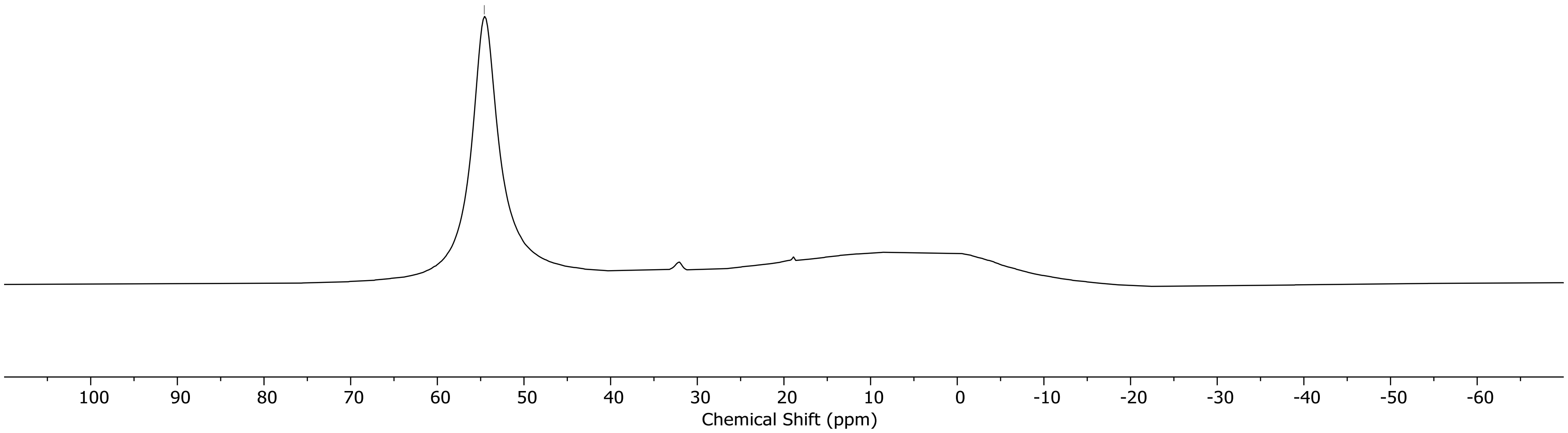

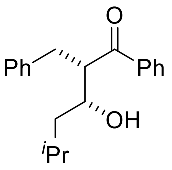

3a
Crude Reaction
^{1}H, $CDCl_3$, 600 MHz

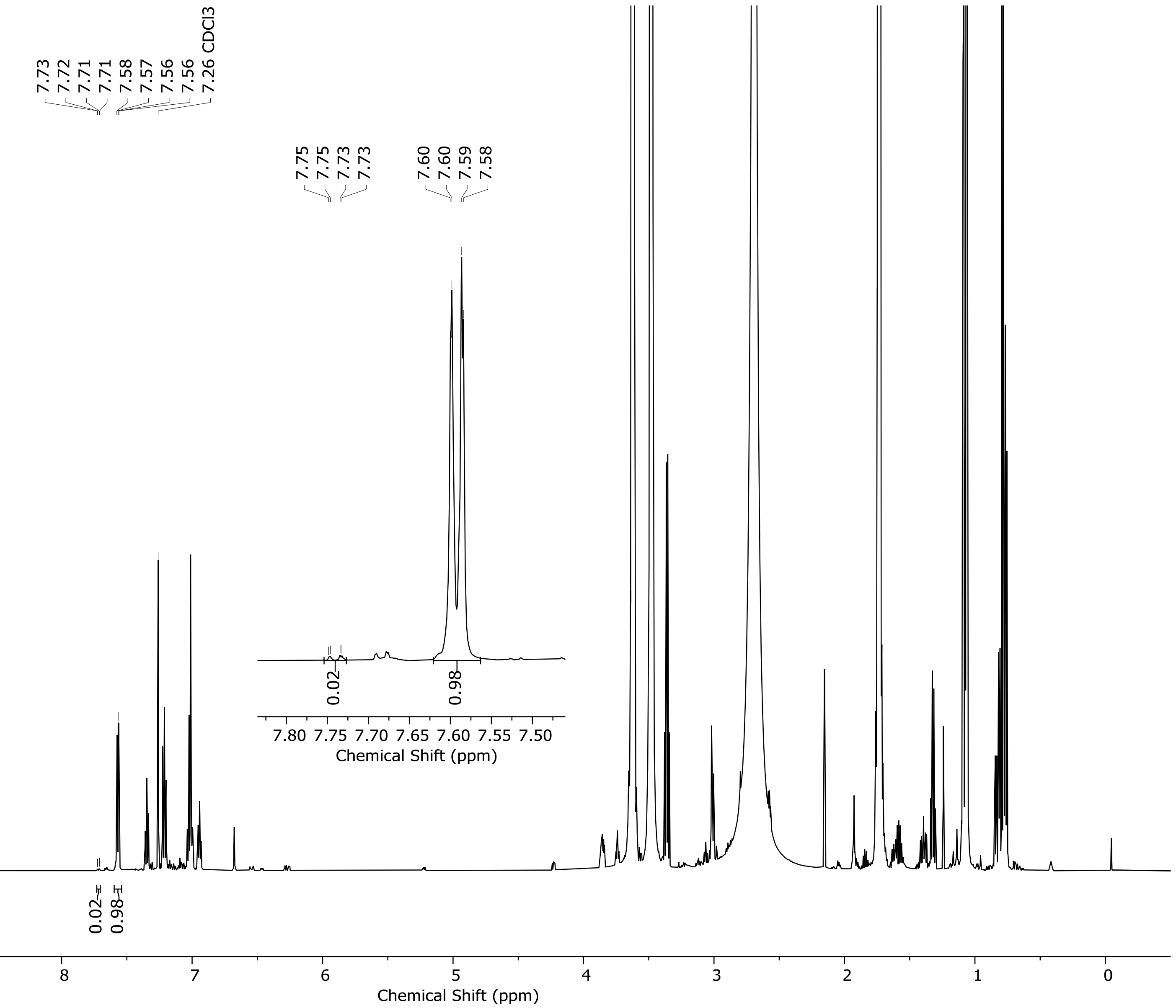

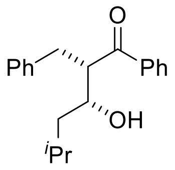

3a

^{1}H, $CDCl_3$, 500 MHz

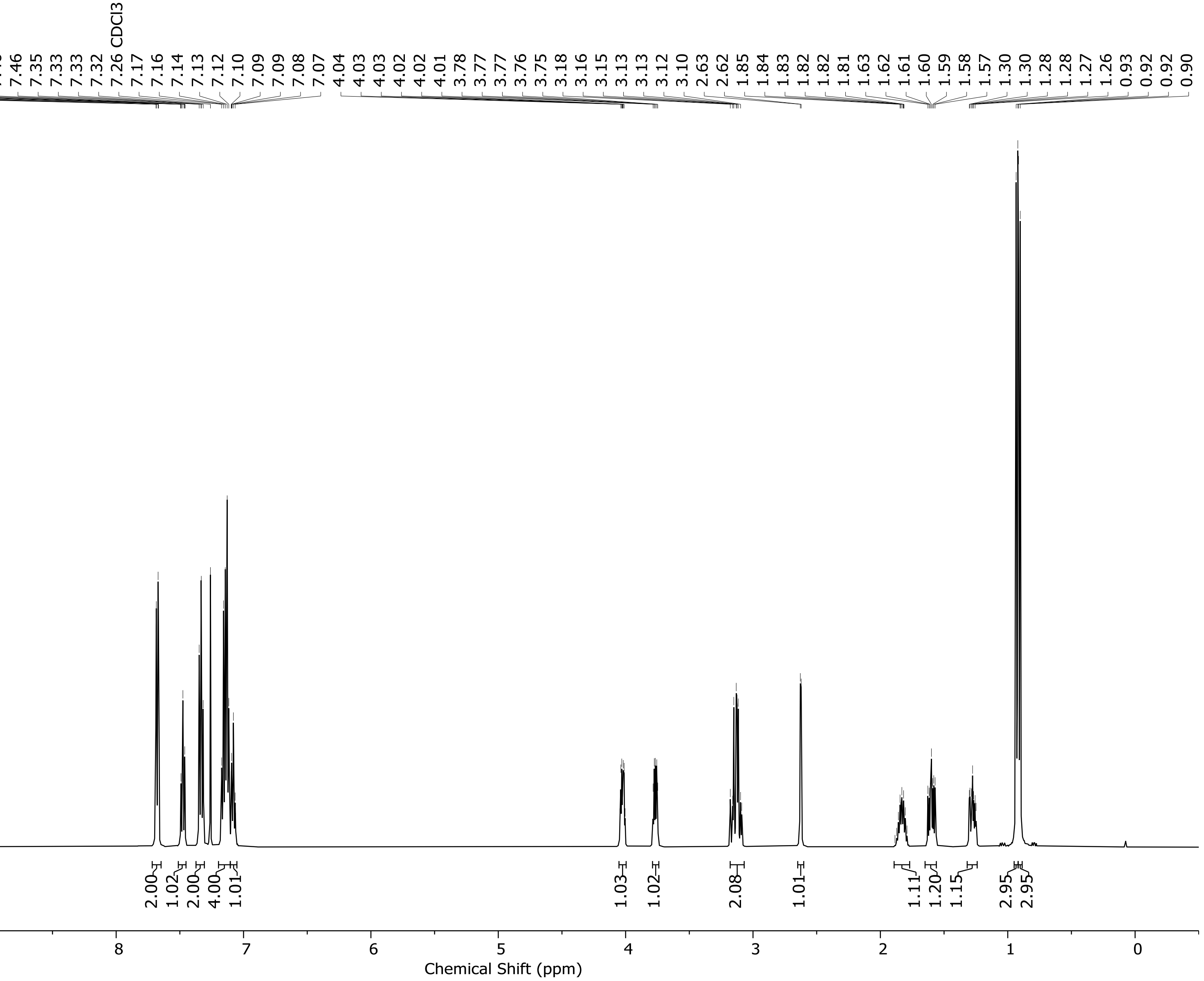

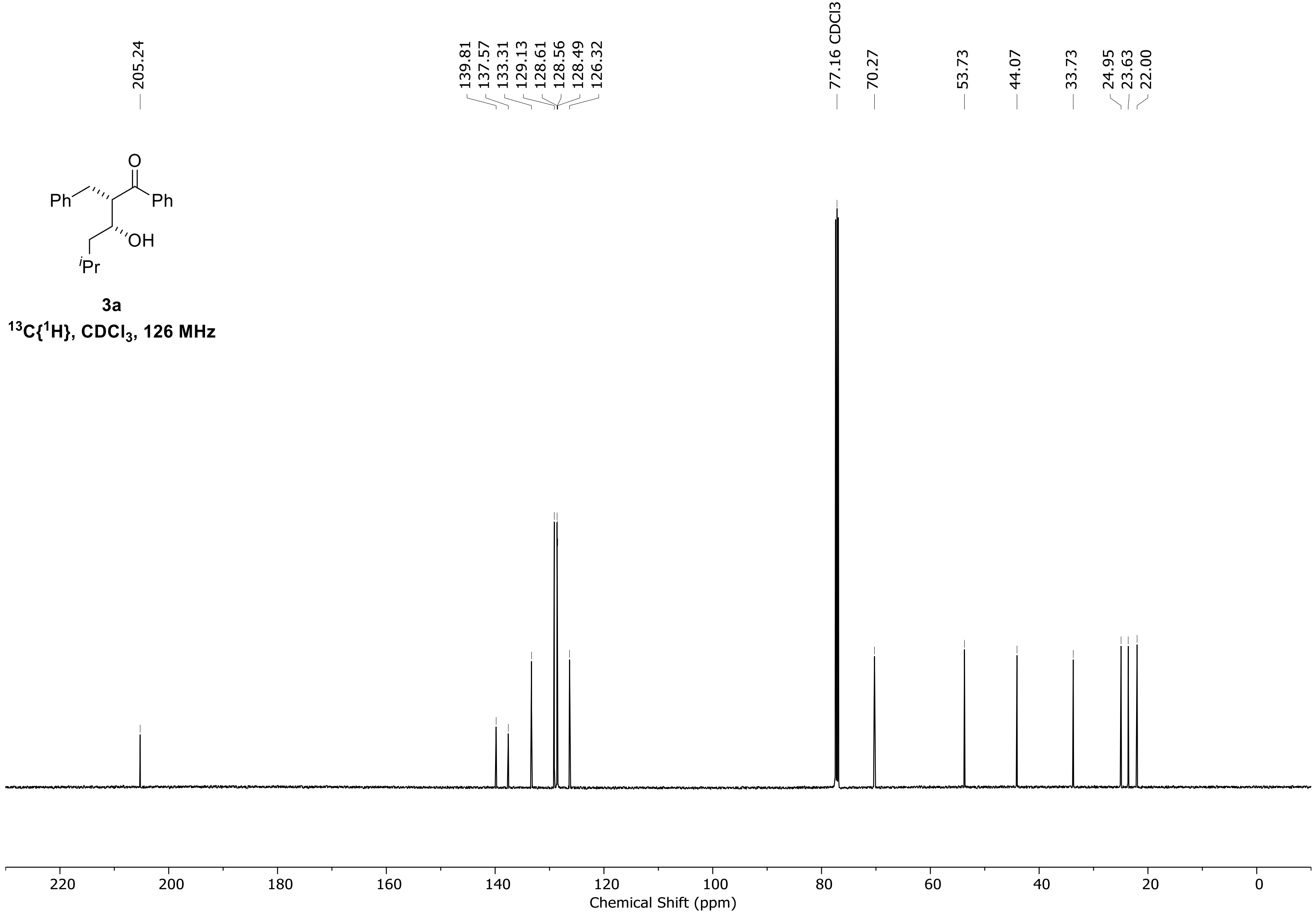

3a

$^{13}C\{^1H\}$, $CDCl_3$, 126 MHz

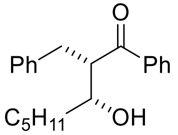

3b
Crude Reaction
^{1}H, $CDCl_3$, 400 MHz

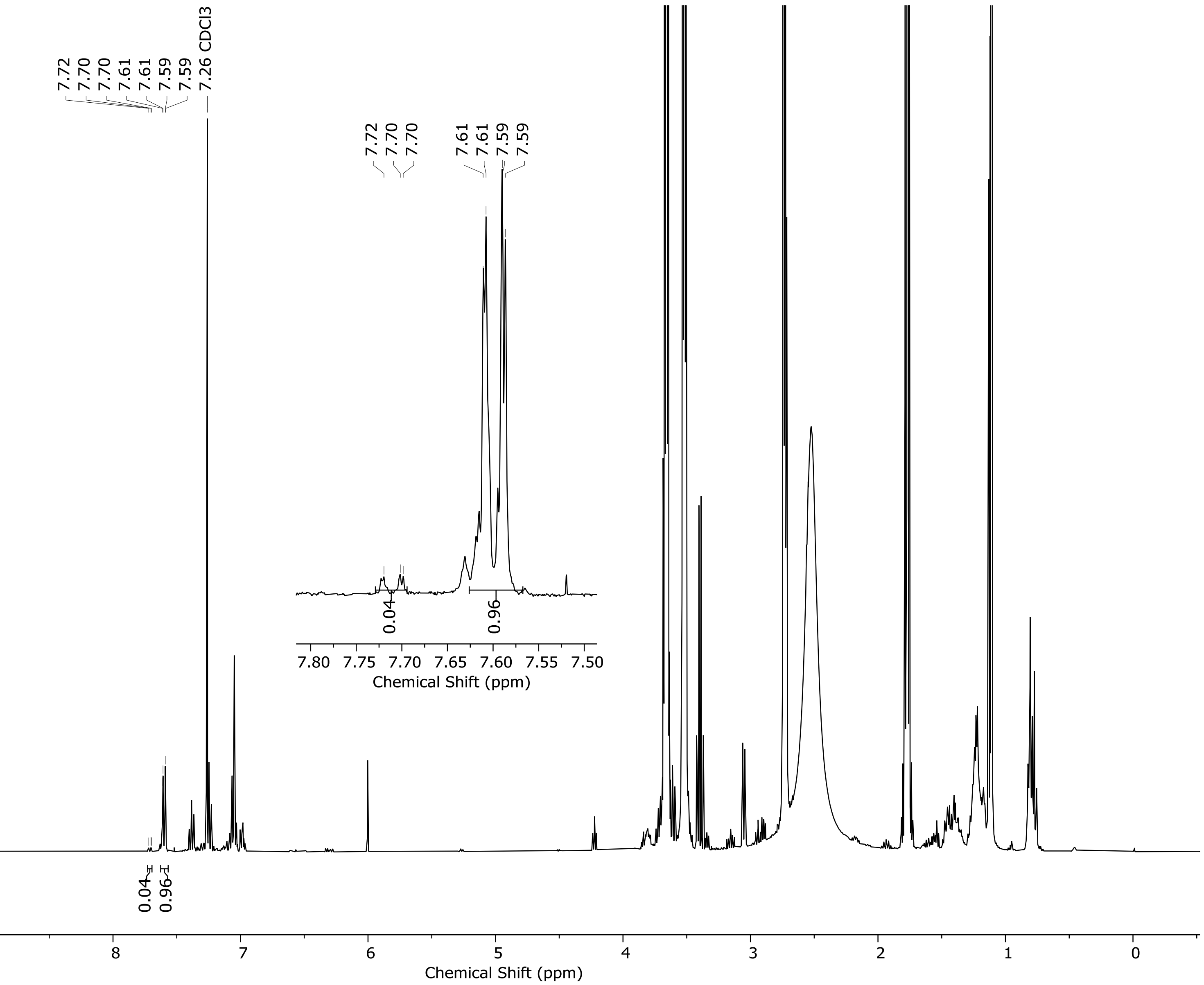

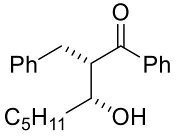

3b

^{1}H, CDCl$_3$, 500 MHz

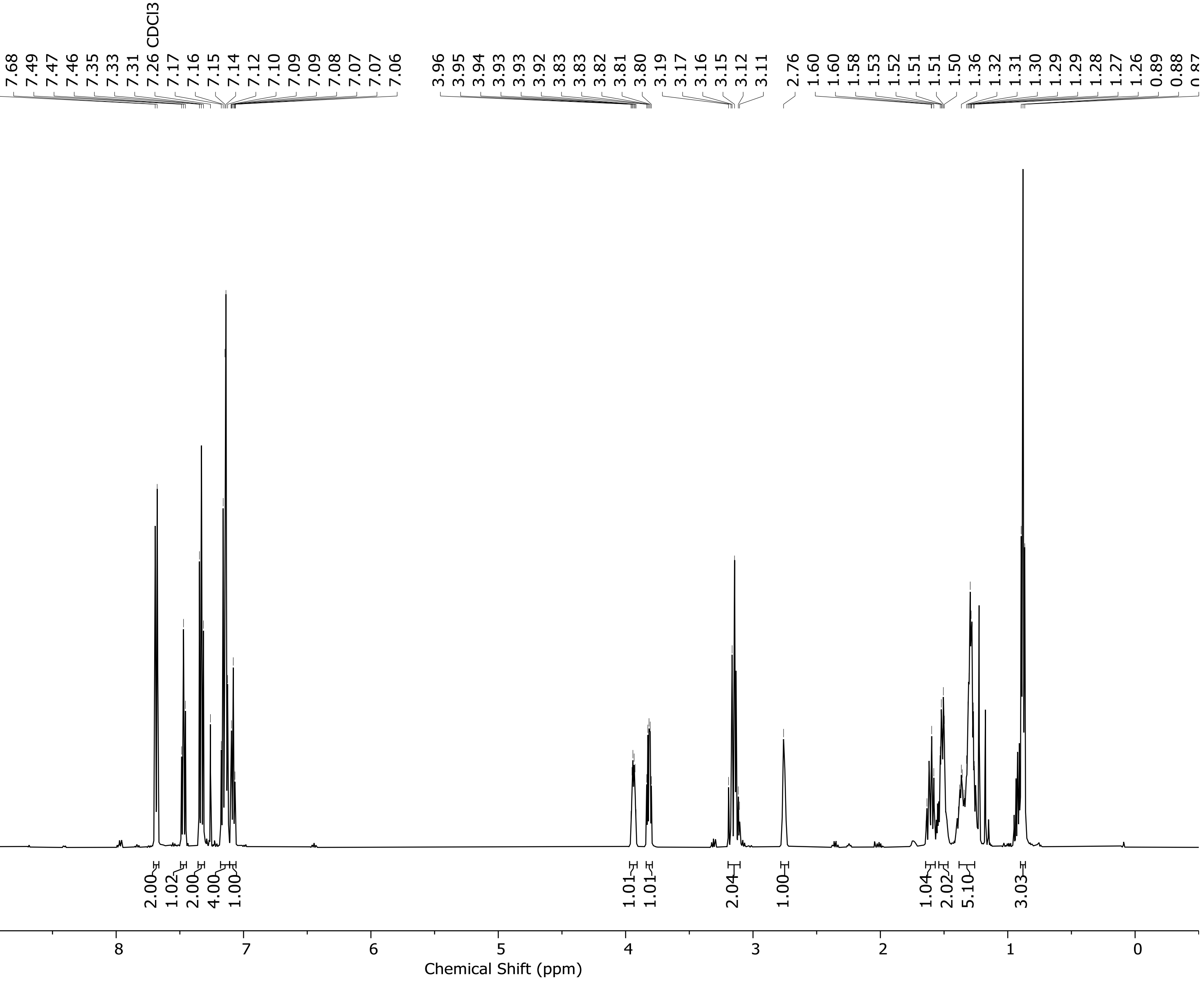

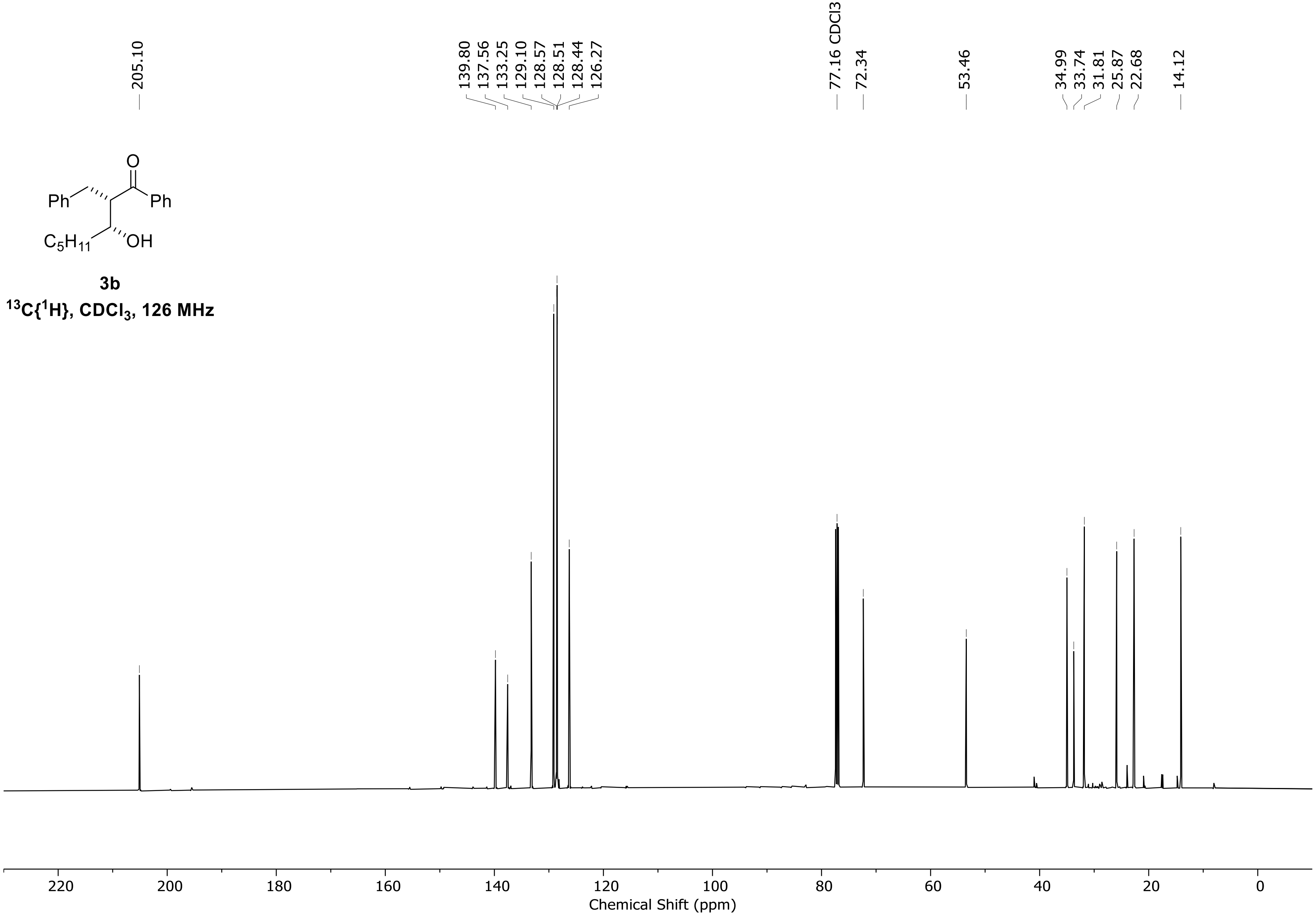

Ph
Ph
O
C5H11
OH
3b
13C{1H}, CDCl3, 126 MHz
205.10
139.80
137.56
133.25
129.10
128.57
128.51
128.44
126.27
77.16 CDCl3
72.34
53.46
34.99
33.74
31.81
25.87
22.68
14.12
220
200
180
160
140
120
100
80
60
40
20
0
Chemical Shift (ppm)

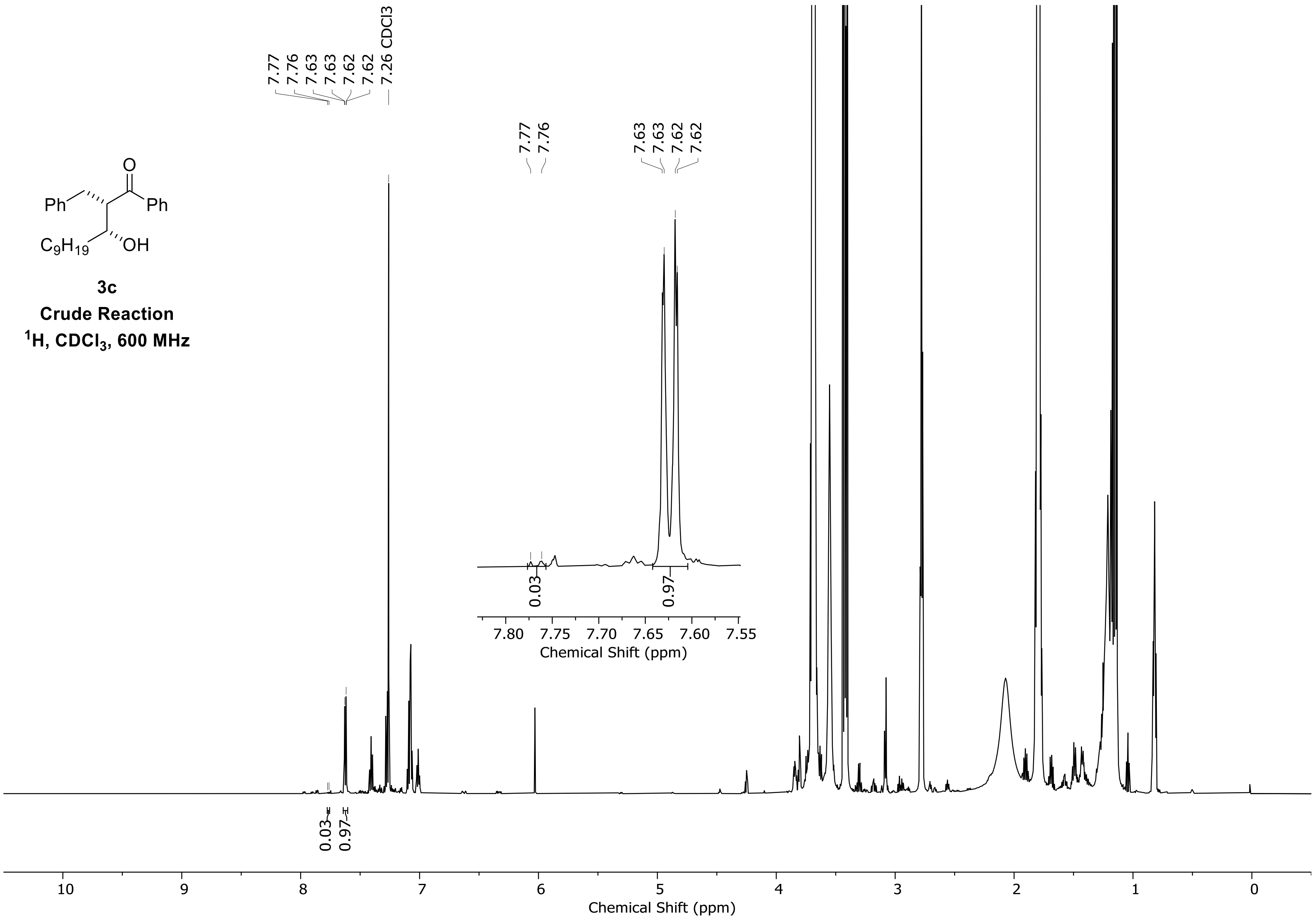

3c
Crude Reaction
^{1}H, CDCl$_3$, 600 MHz

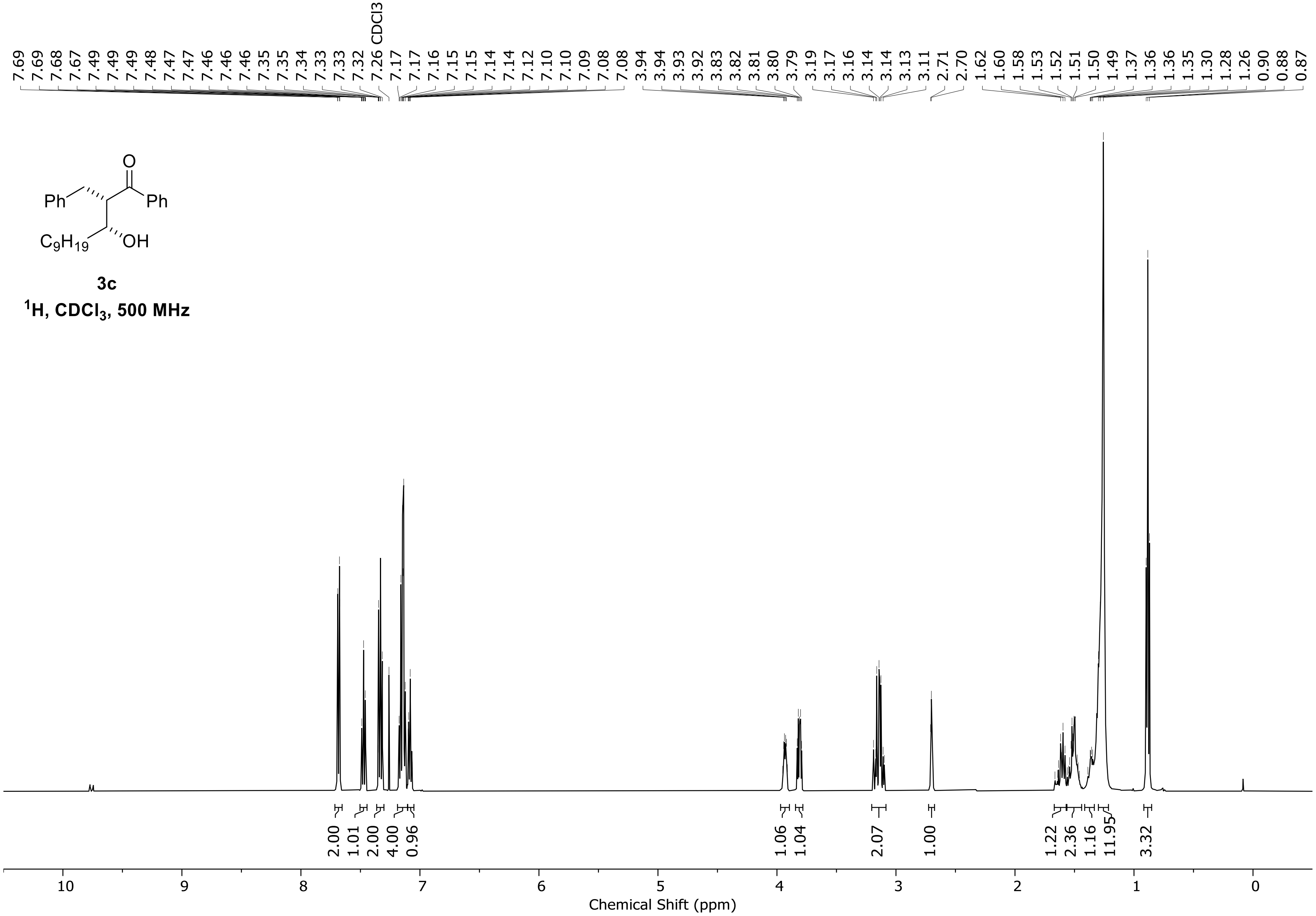
7.69 7.69 7.68 7.67 7.49 7.49 7.49 7.48 7.47 7.47 7.46 7.46 7.46 7.35 7.35 7.34 7.33 7.33 7.32 7.26 CDCl3 7.17 7.17 7.16 7.15 7.15 7.14 7.14 7.12 7.10 7.10 7.09 7.08 7.08 3.94 3.94 3.93 3.92 3.83 3.82 3.81 3.80 3.79 3.19 3.17 3.16 3.14 3.14 3.13 3.11 2.71 2.70 1.62 1.60 1.58 1.53 1.52 1.51 1.50 1.49 1.37 1.36 1.36 1.35 1.30 1.28 1.26 0.90 0.88 0.87
O
Ph
Ph
C_9H_{19}
OH
3c
^1H, $CDCl_3$, 500 MHz
2.00 1.01 2.00 4.00 0.96 1.06 1.04 2.07 1.00 1.22 2.36 1.16 11.95 3.32
10 9 8 7 6 5 4 3 2 1 0
Chemical Shift (ppm)

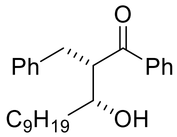

3c

$^{13}C\{^1H\}$, $CDCl_3$, 126 MHz

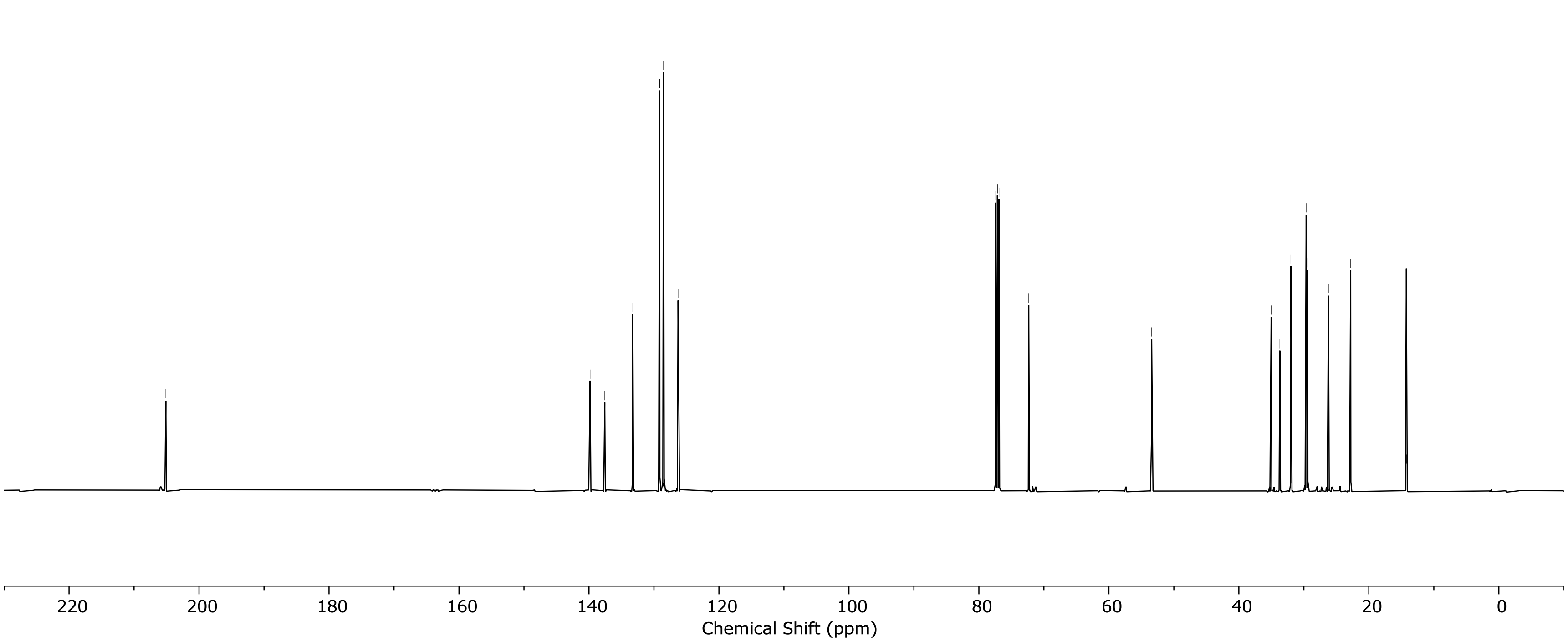

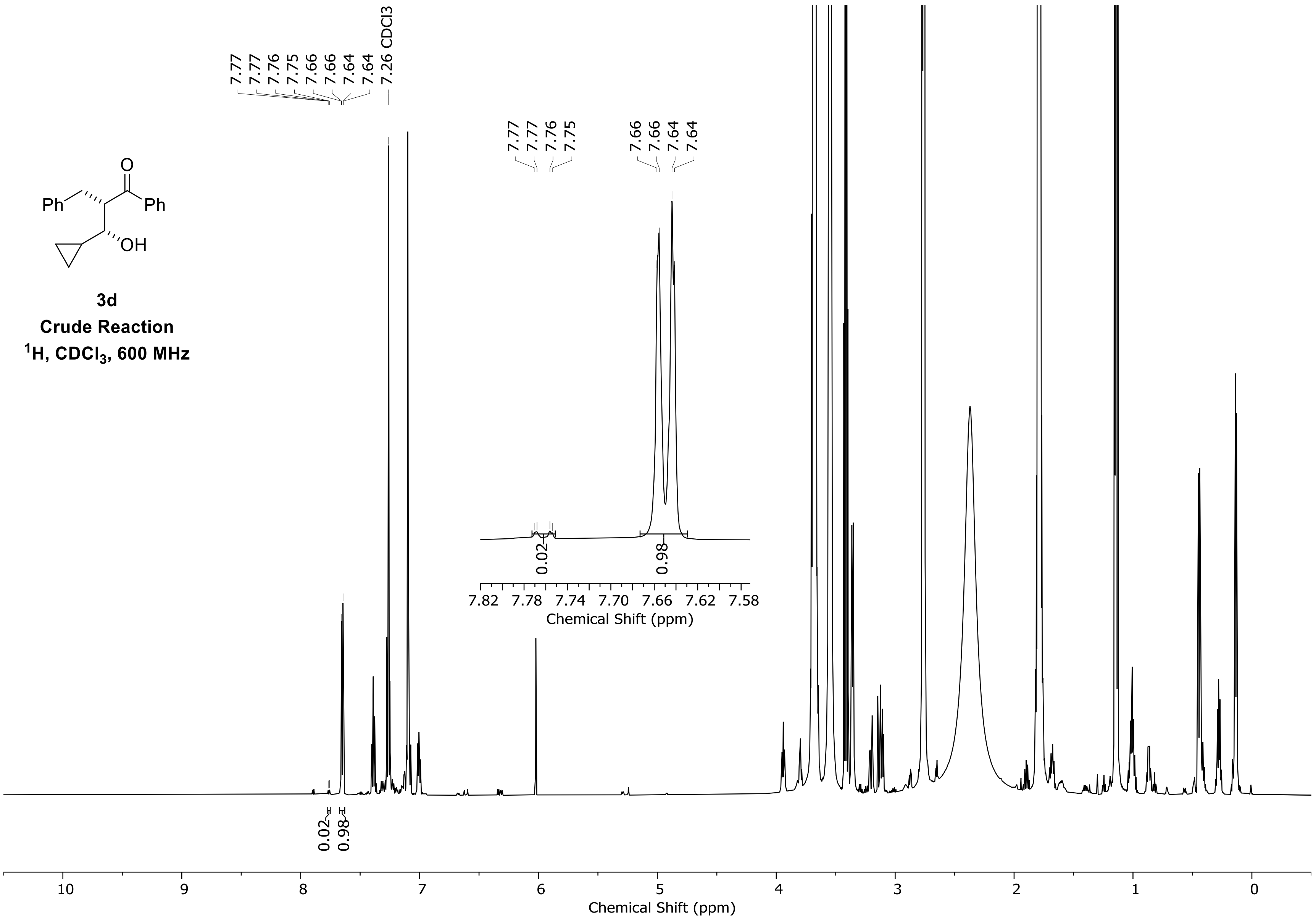
7.77
7.77
7.76
7.75
7.66
7.66
7.64
7.64
7.26 CDCl3
O
Ph
Ph
OH
3d
Crude Reaction
1H, CDCl3, 600 MHz
0.02
0.98
7.82 7.78 7.74 7.70 7.66 7.62 7.58
Chemical Shift (ppm)
10 9 8 7 6 5 4 3 2 1 0
Chemical Shift (ppm)

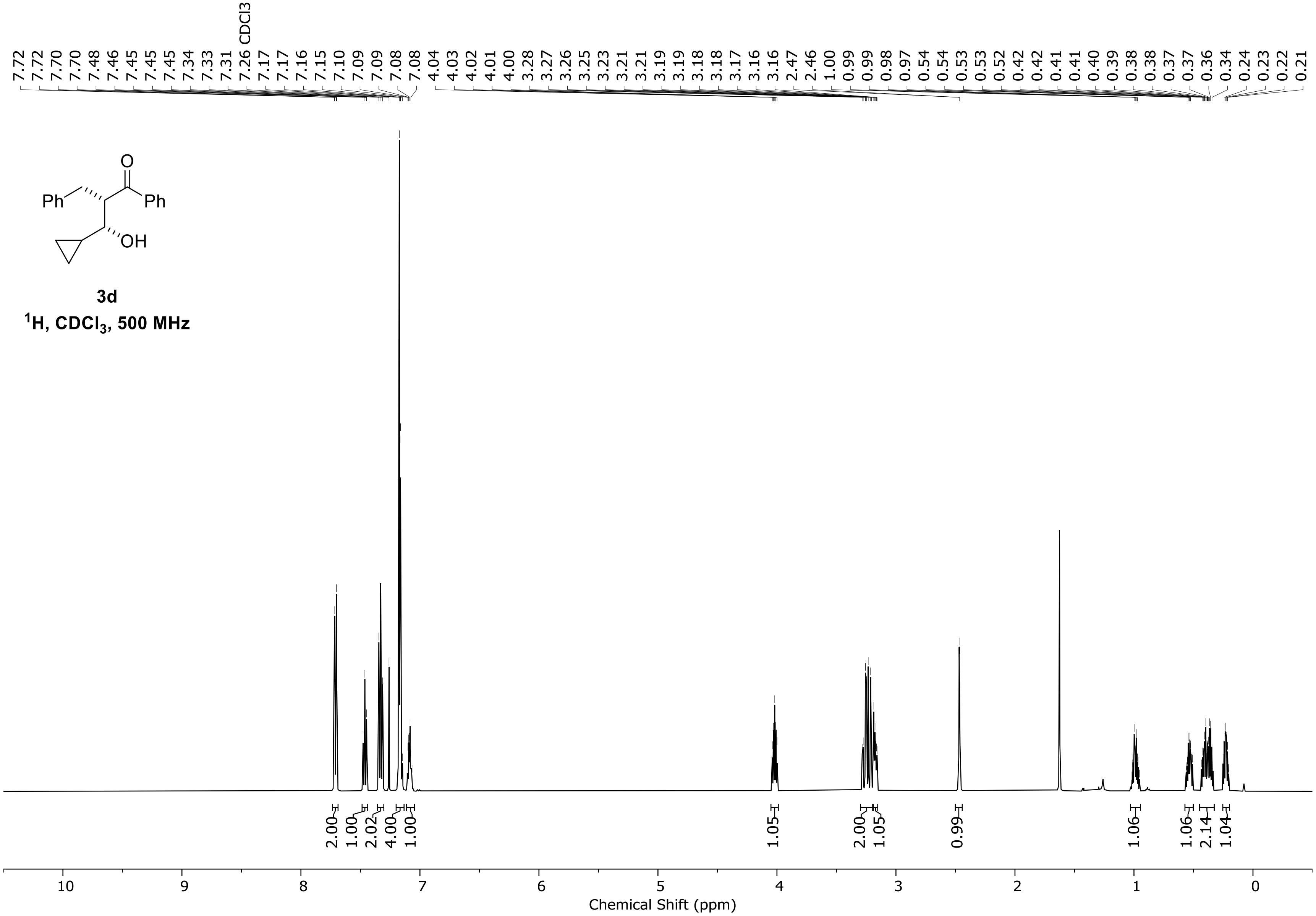
3d
1H, CDCl3, 500 MHz
Ph
Ph
O
OH
CDCl3
Chemical Shift (ppm)

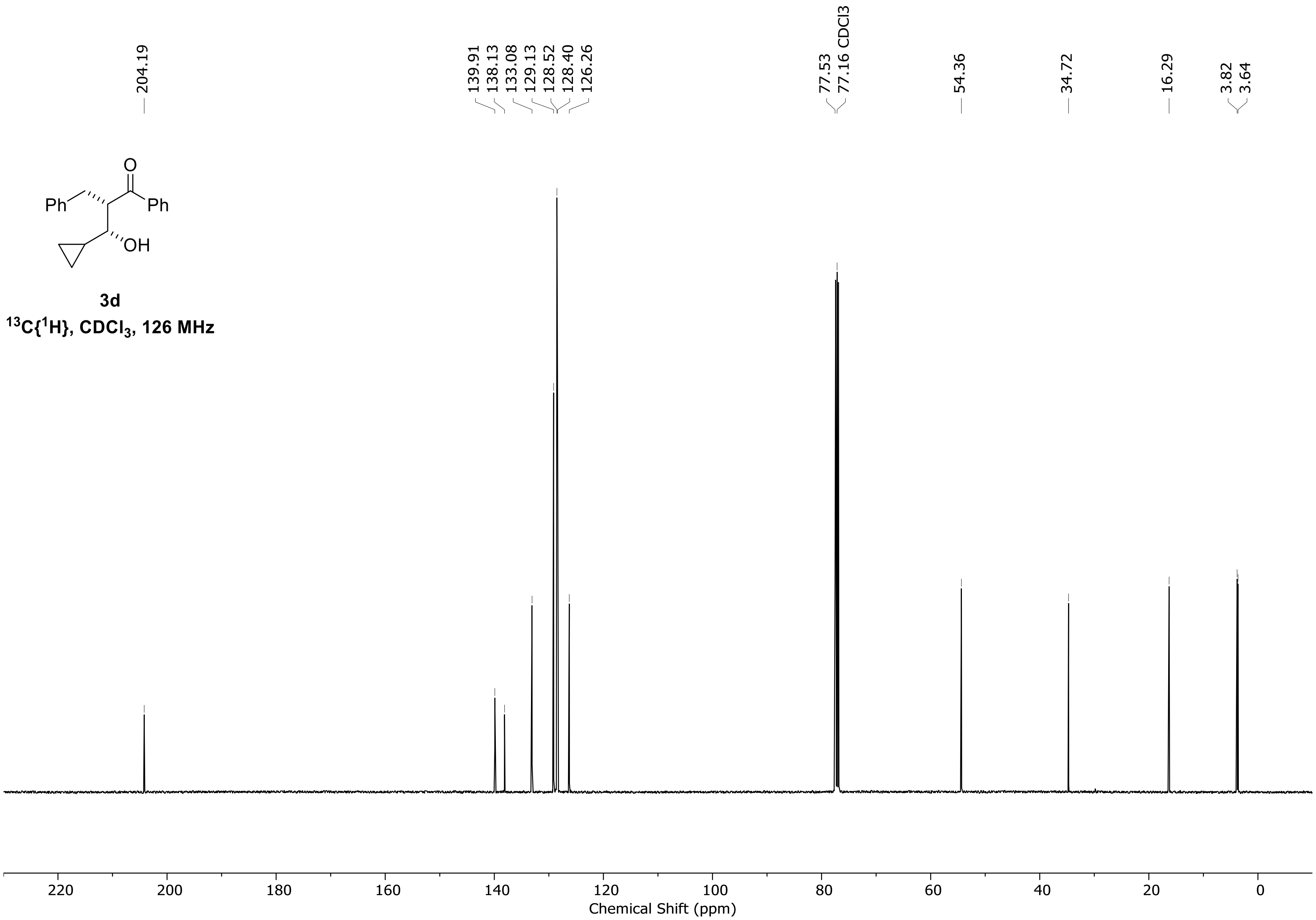

3d

$^{13}C\{^{1}H\}$, $CDCl_3$, 126 MHz

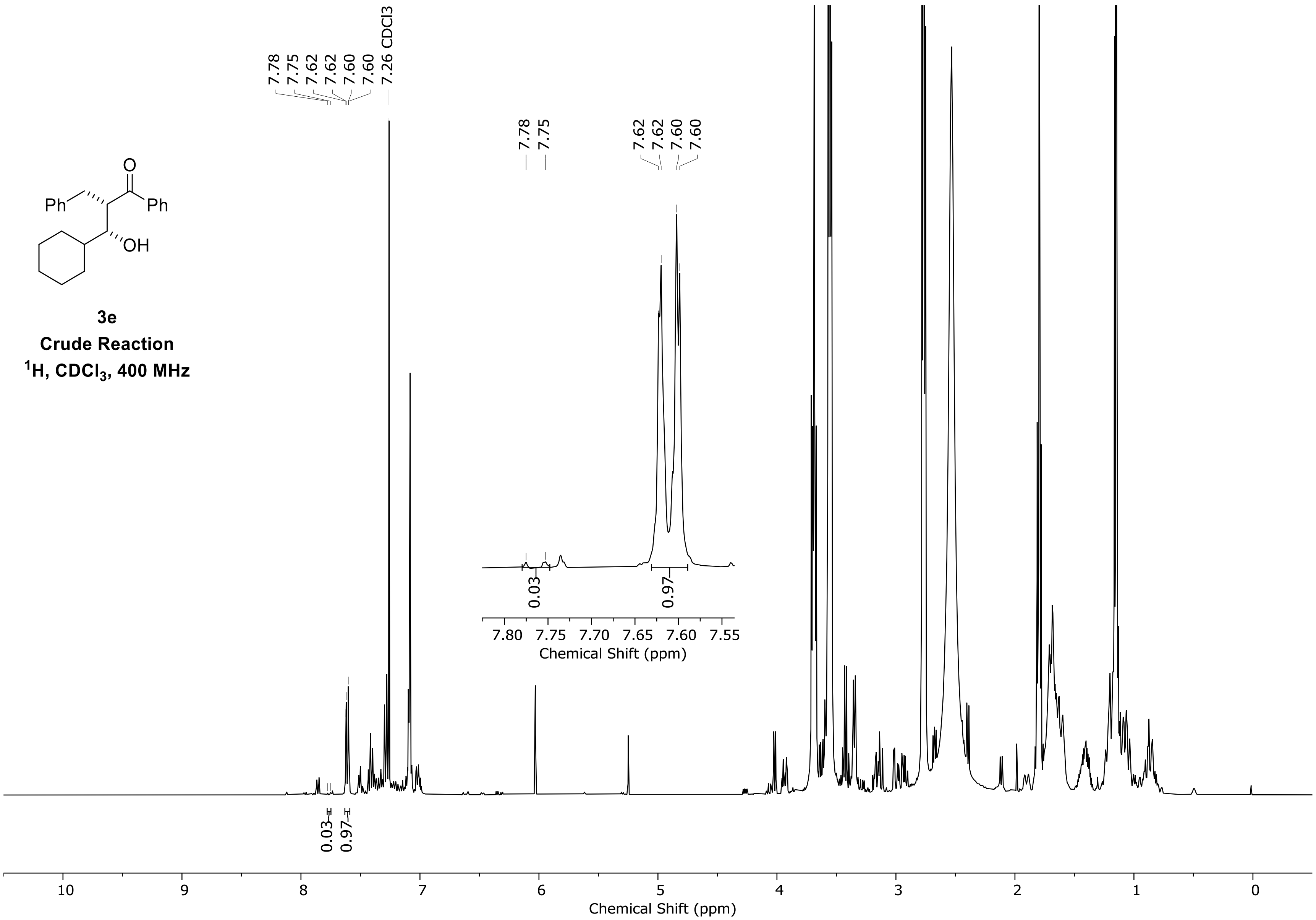
Ph
O
Ph
OH
3e
Crude Reaction
1H, CDCl3, 400 MHz
7.78
7.75
7.62
7.62
7.60
7.60
7.26 CDCl3
0.03
0.97
7.80 7.75 7.70 7.65 7.60 7.55
Chemical Shift (ppm)
10 9 8 7 6 5 4 3 2 1 0
Chemical Shift (ppm)

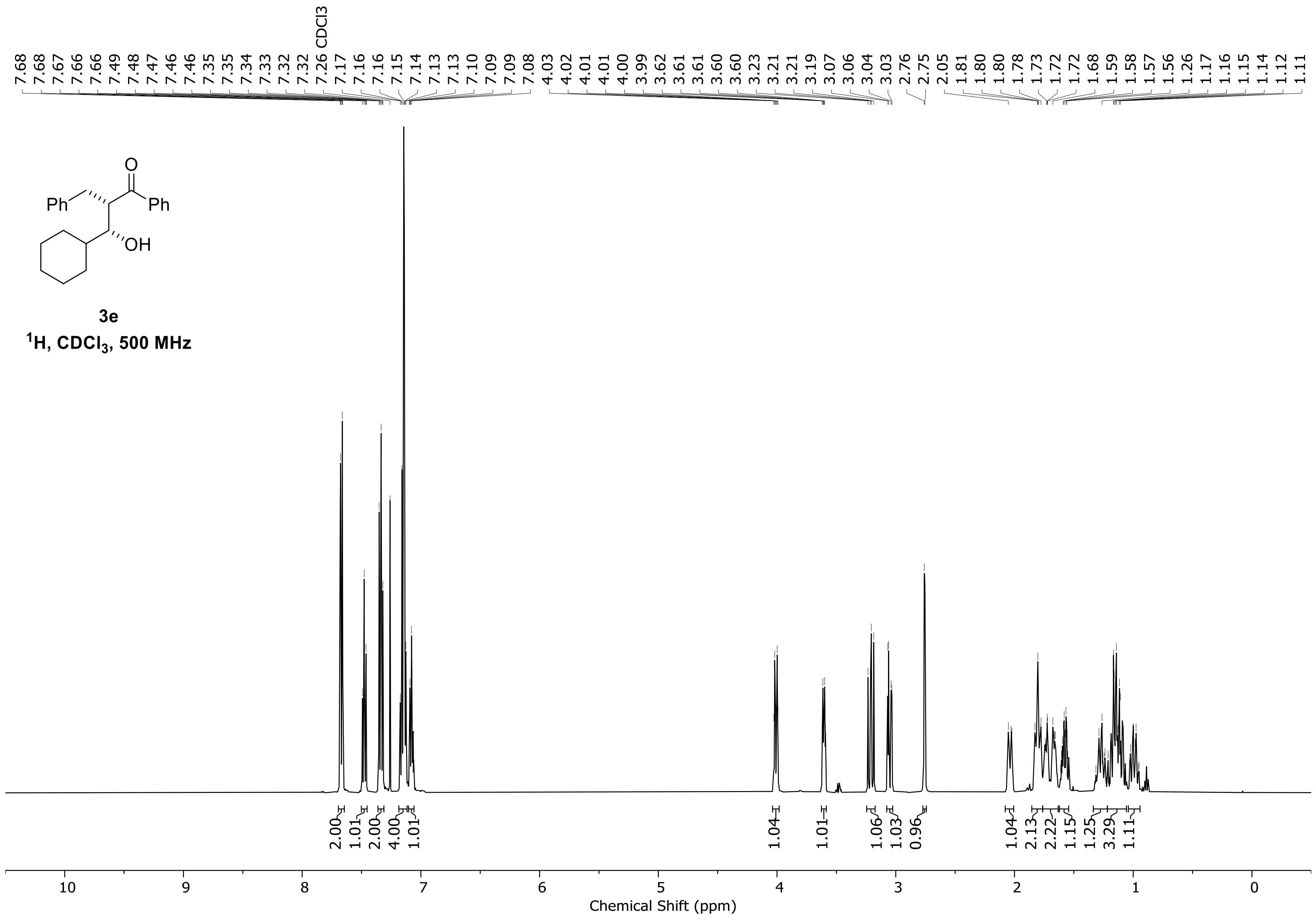

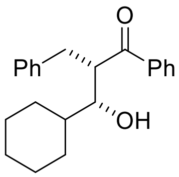

3e

$^{13}C\{^{1}H\}$, $CDCl_3$, 126 MHz

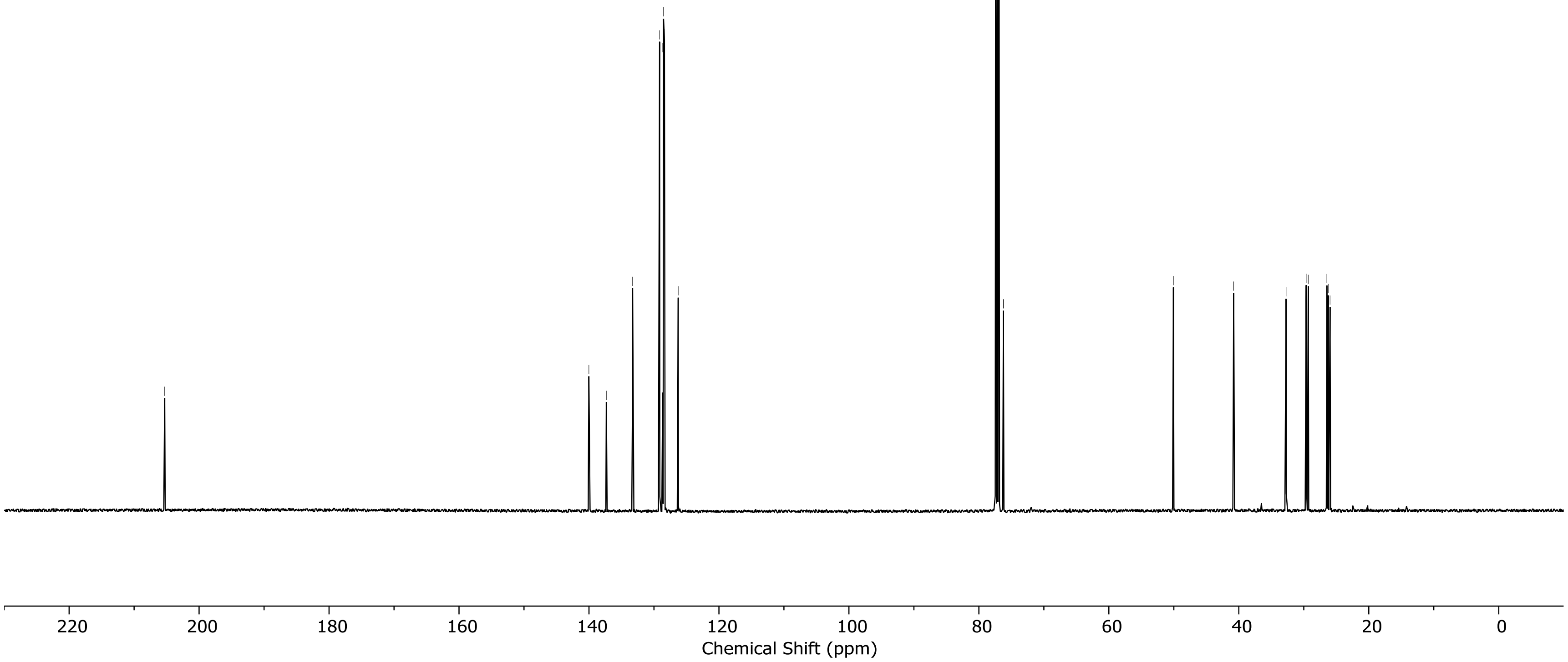

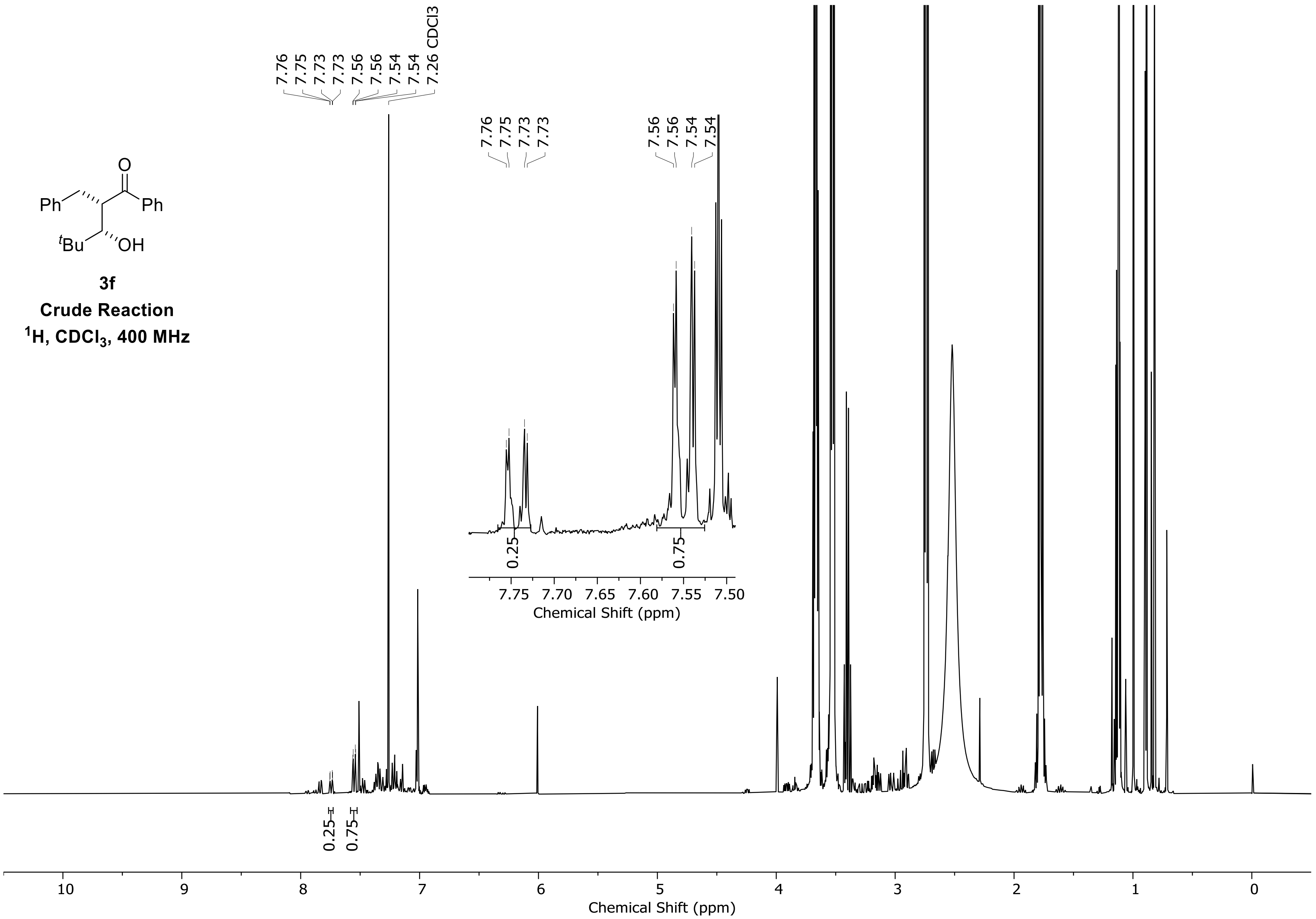

Ph
Ph
O
^{t}Bu
OH
3f
Crude Reaction
^{1}H, CDCl$_3$, 400 MHz
7.76
7.75
7.73
7.73
7.56
7.56
7.54
7.54
7.26 CDCl3
0.25
0.75
Chemical Shift (ppm)

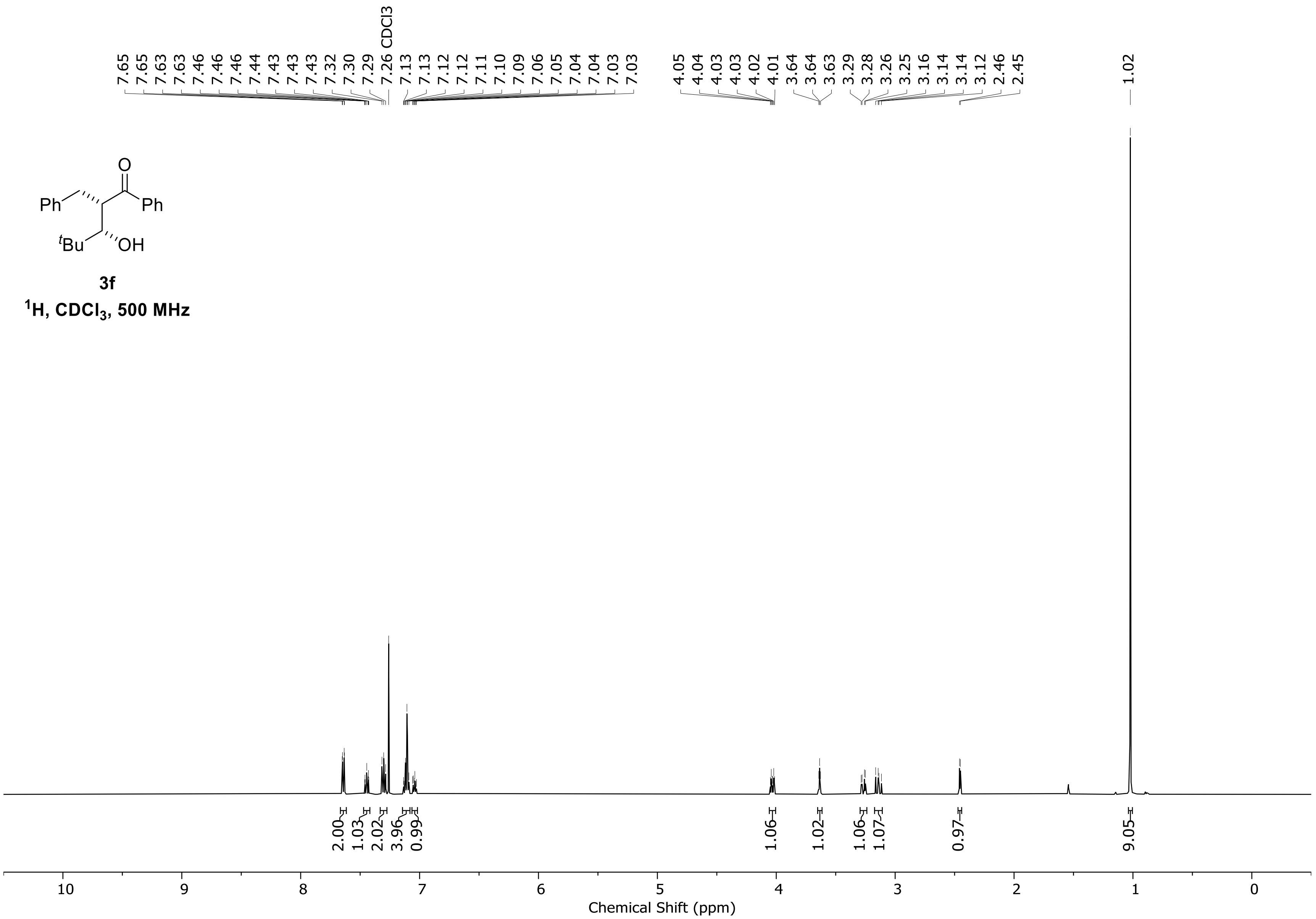

3f

^{1}H, CDCl$_3$, 500 MHz

3f

$^{13}C\{^1H\}$, $CDCl_3$, 126 MHz

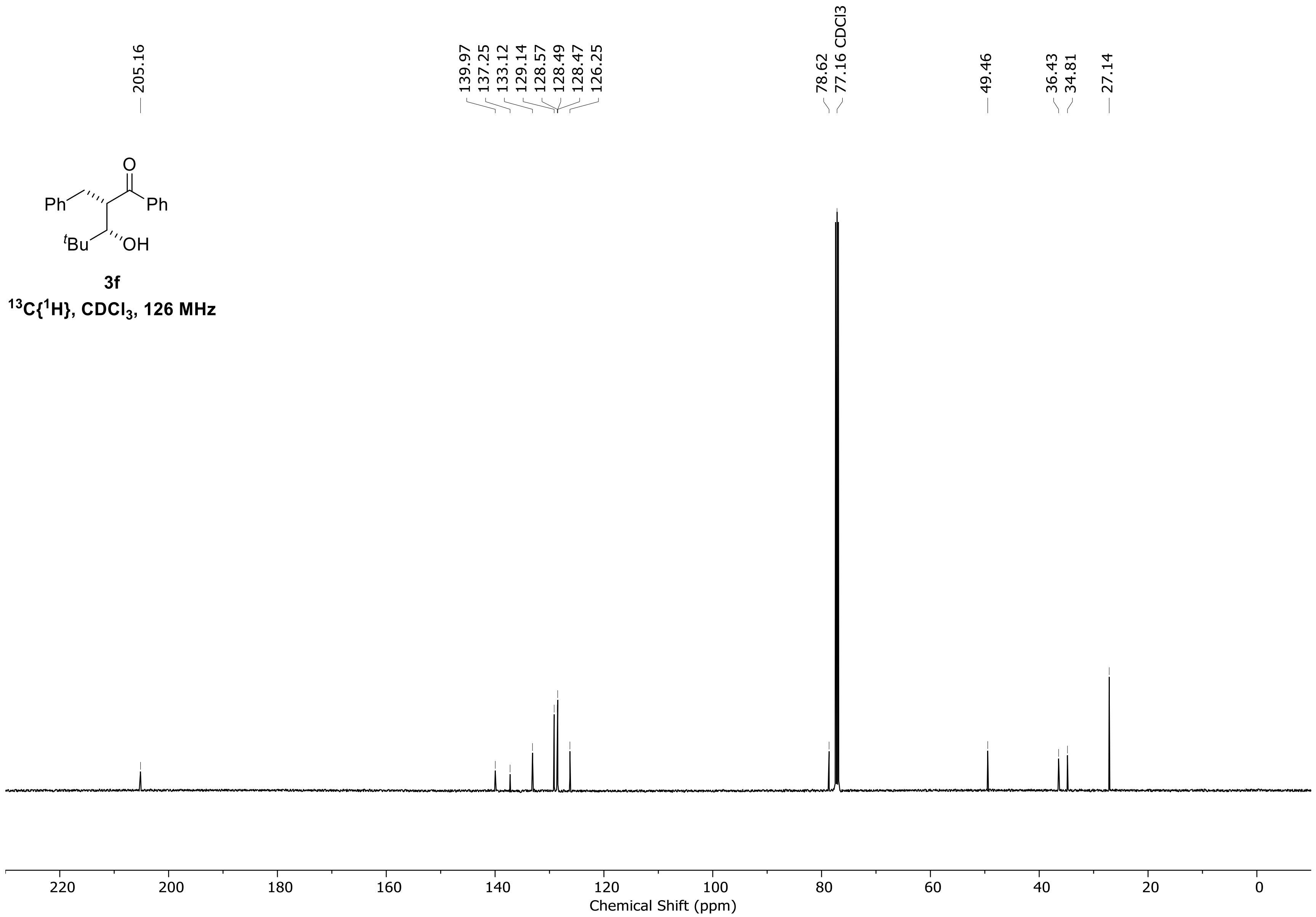

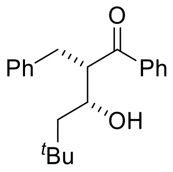

3g
Crude Reaction
^{1}H, $CDCl_3$, 400 MHz

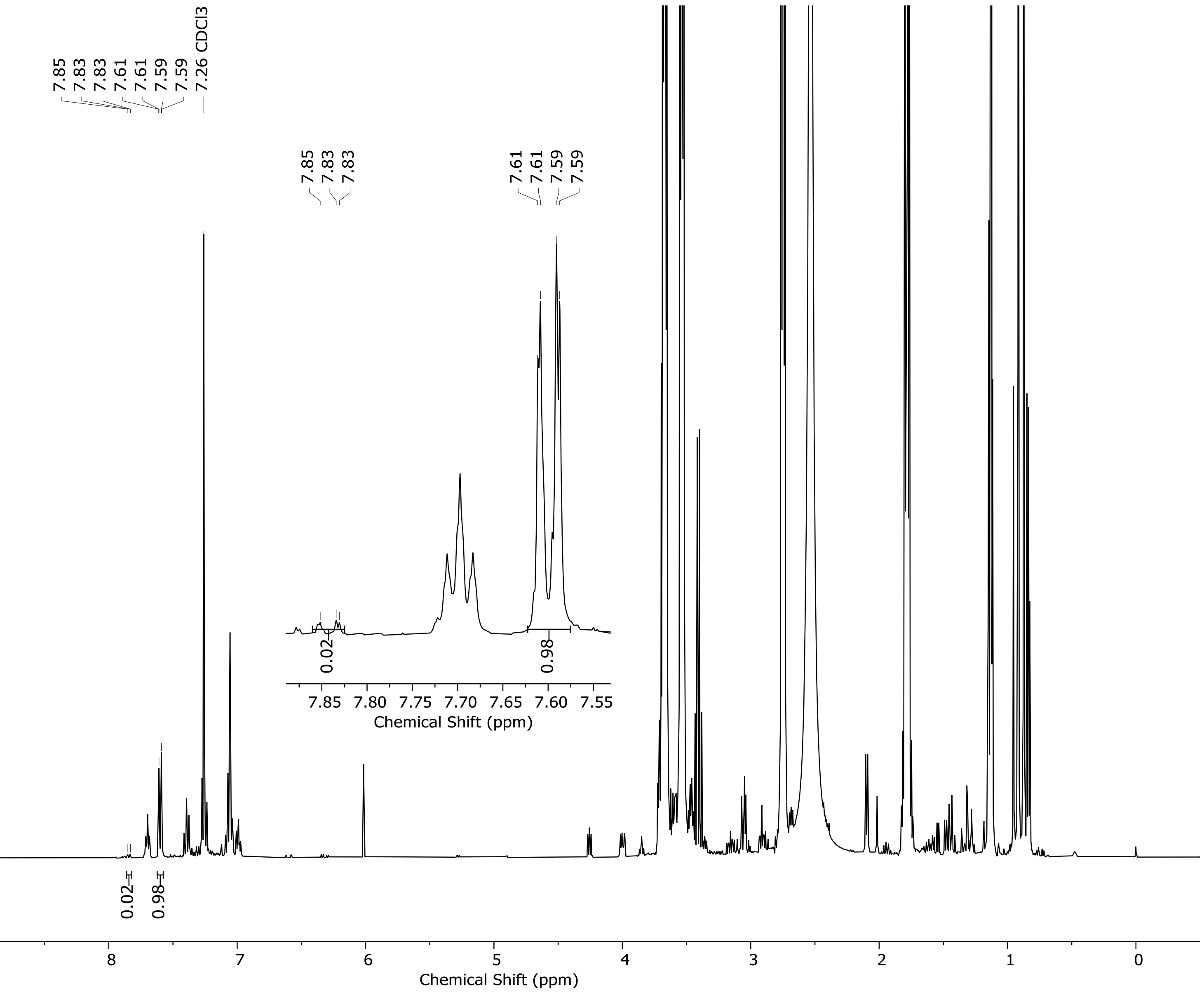

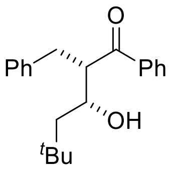

3g
^{1}H, $CDCl_3$, 500 MHz

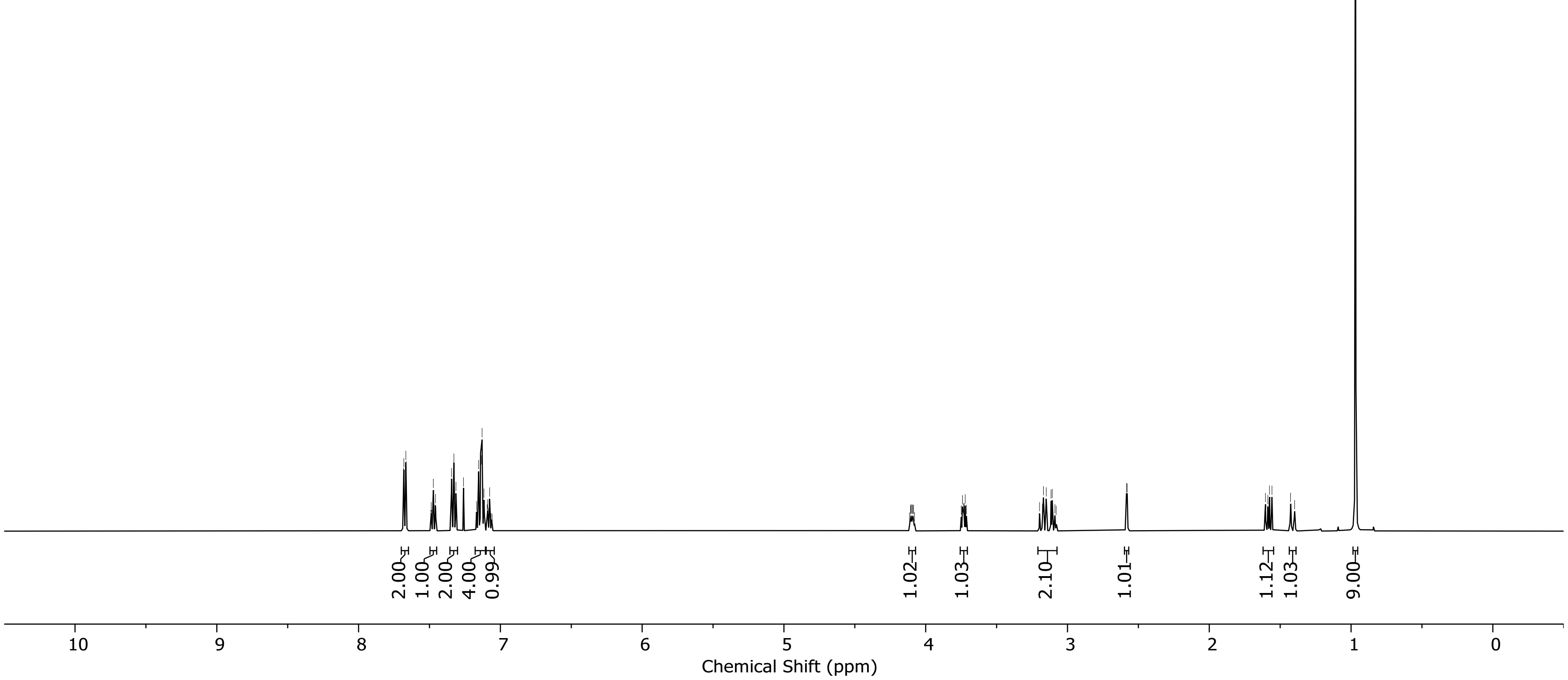

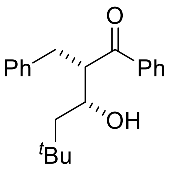

3g

$^{13}C\{^{1}H\}$, $CDCl_3$, 126 MHz

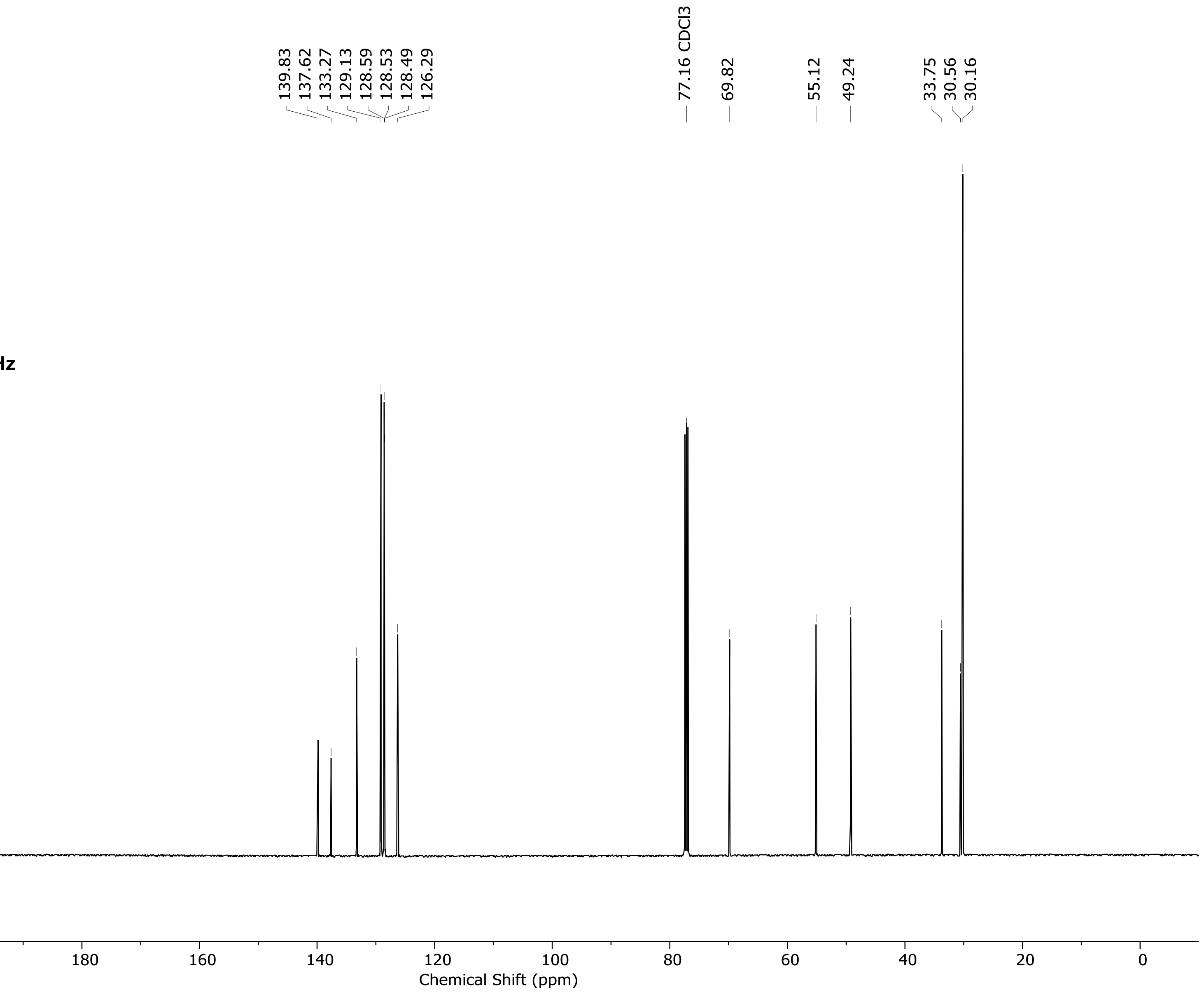

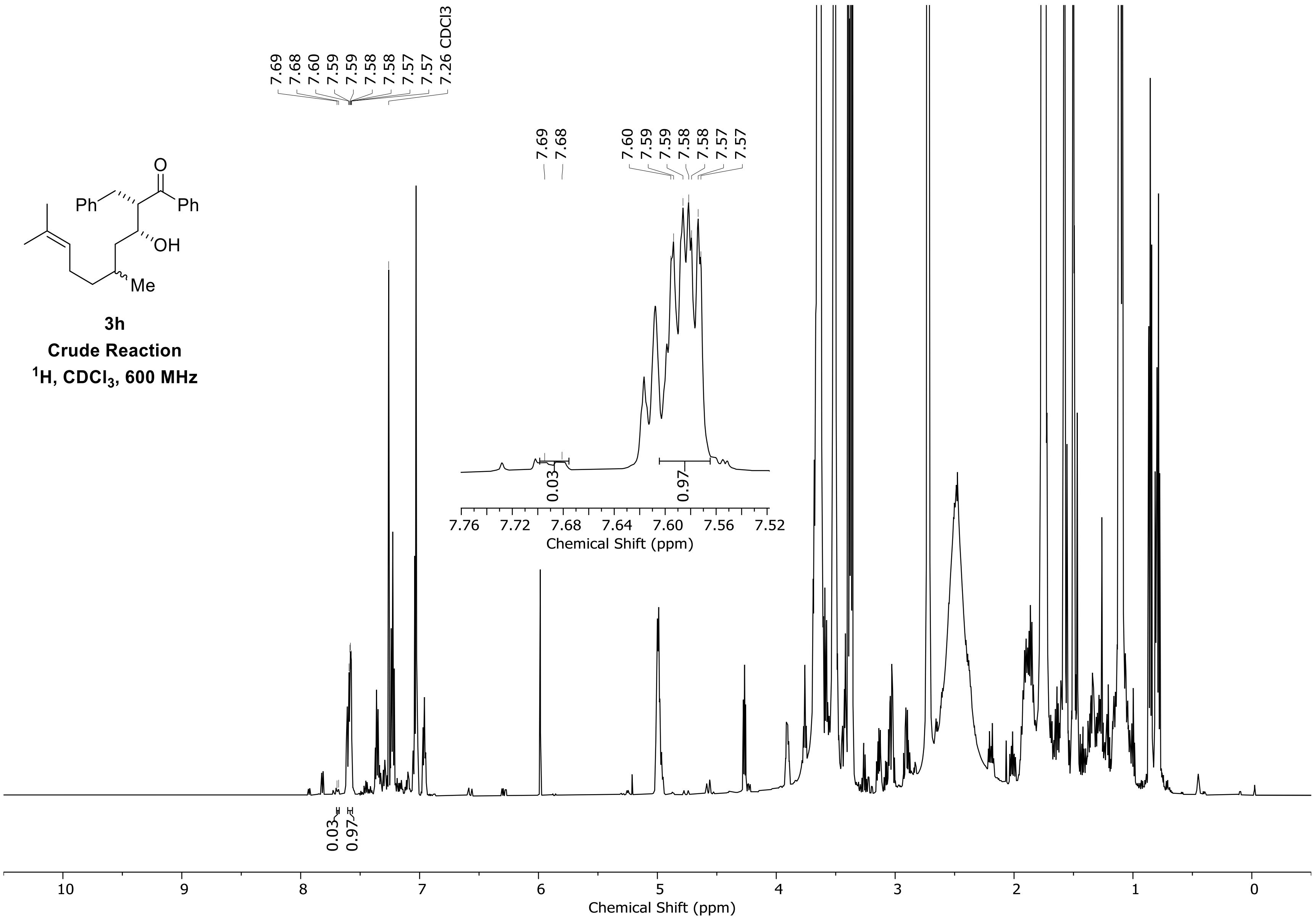

Ph
O
Ph
OH
Me
3h
Crude Reaction
^{1}H, CDCl$_3$, 600 MHz
7.69
7.68
7.60
7.59
7.59
7.58
7.58
7.57
7.57
7.26 CDCl3
0.03
0.97
Chemical Shift (ppm)

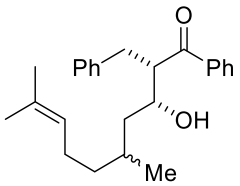

3h

^{1}H, CDCl$_3$, 500 MHz

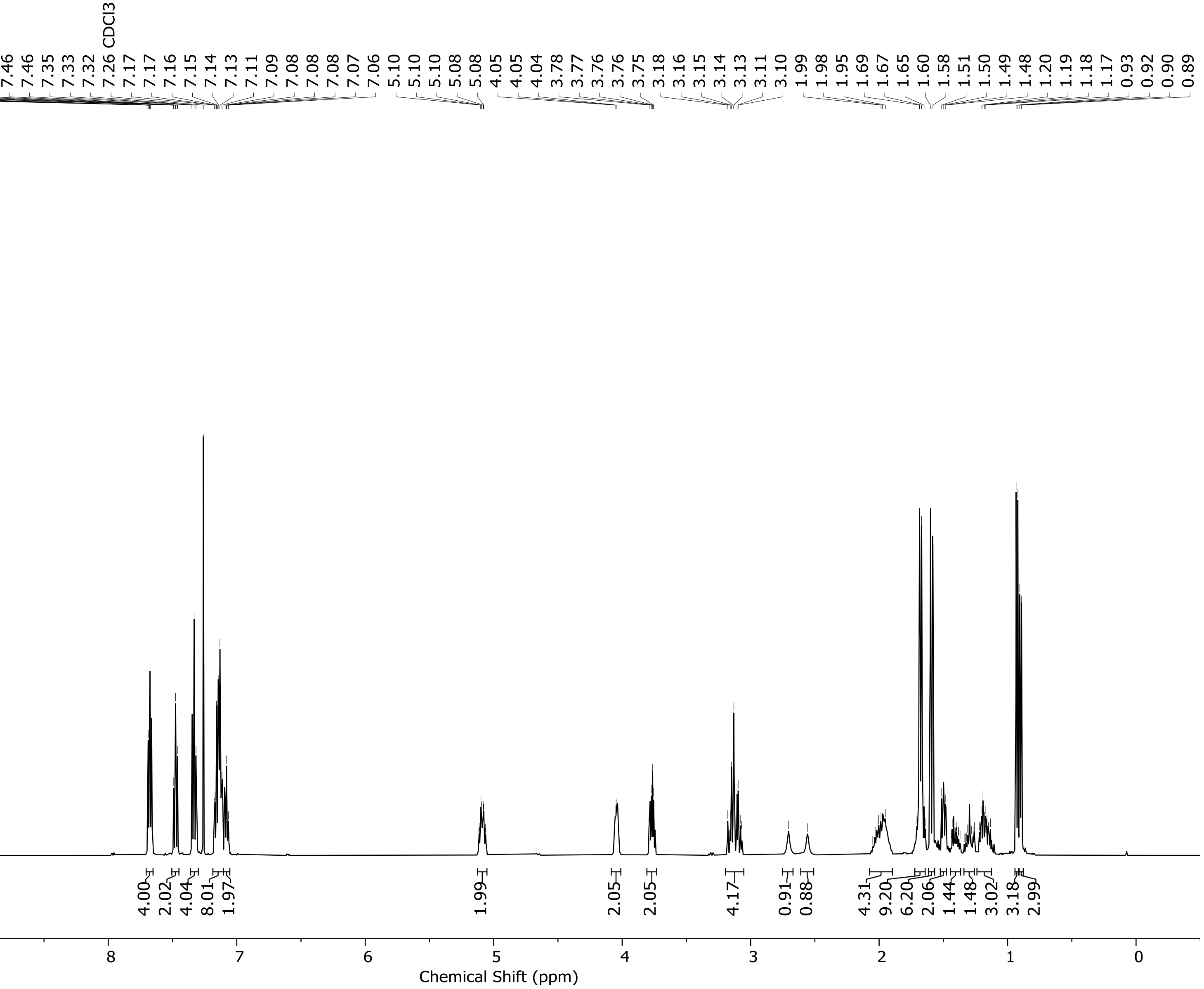

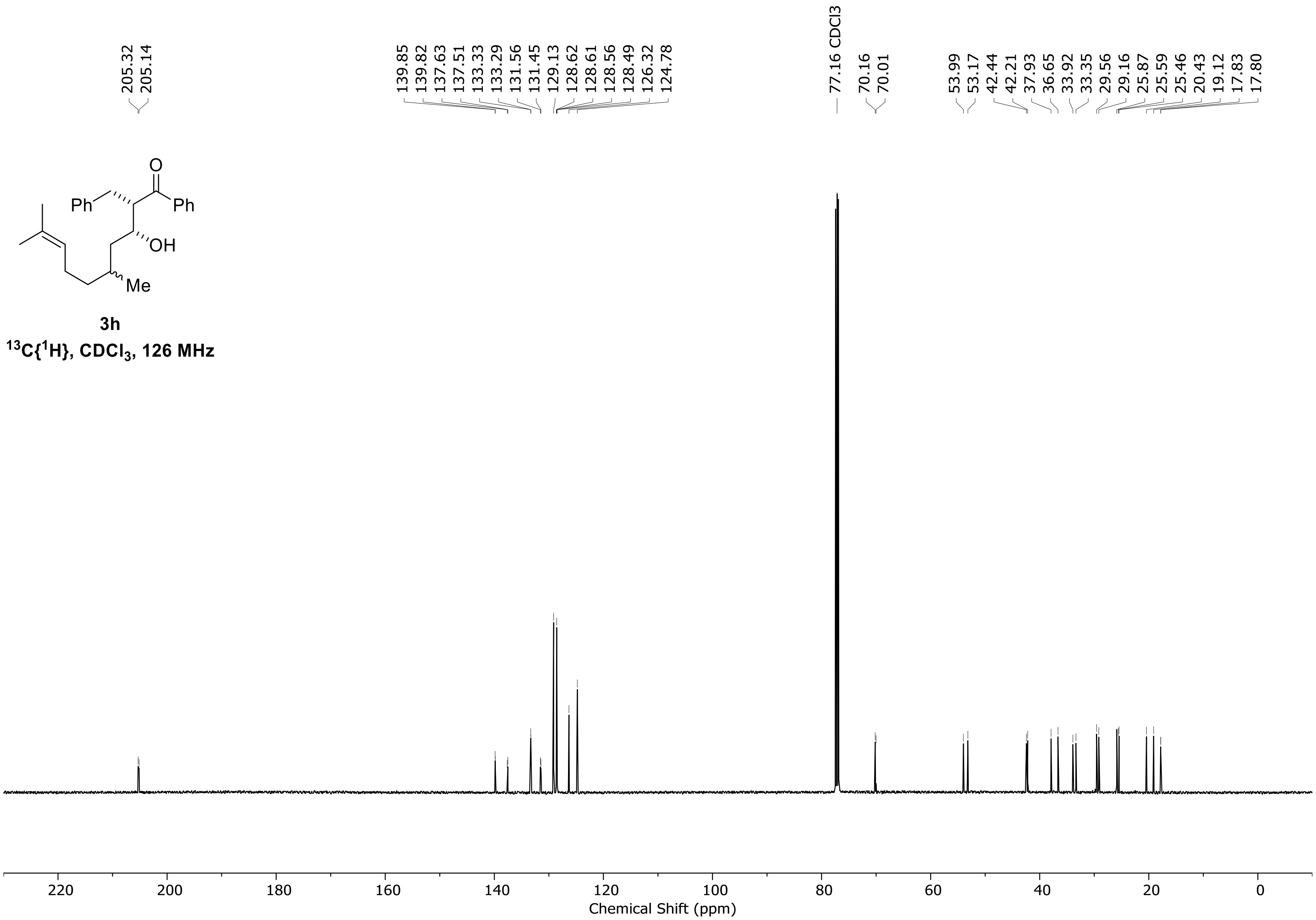

3h

$^{13}C\{^{1}H\}$, $CDCl_3$, 126 MHz

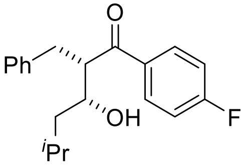

3i
Crude Reaction
^{1}H, $CDCl_3$, 600 MHz

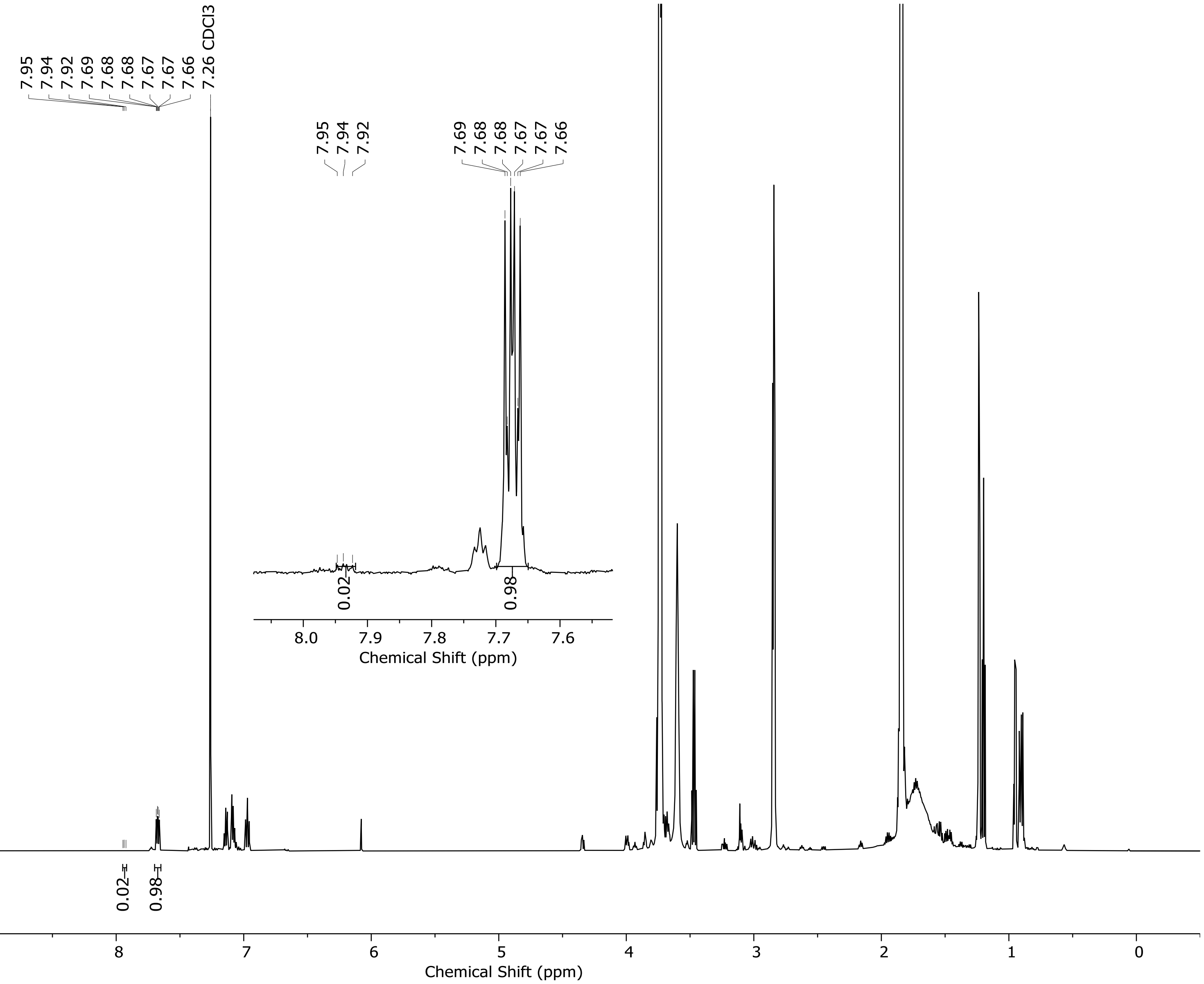

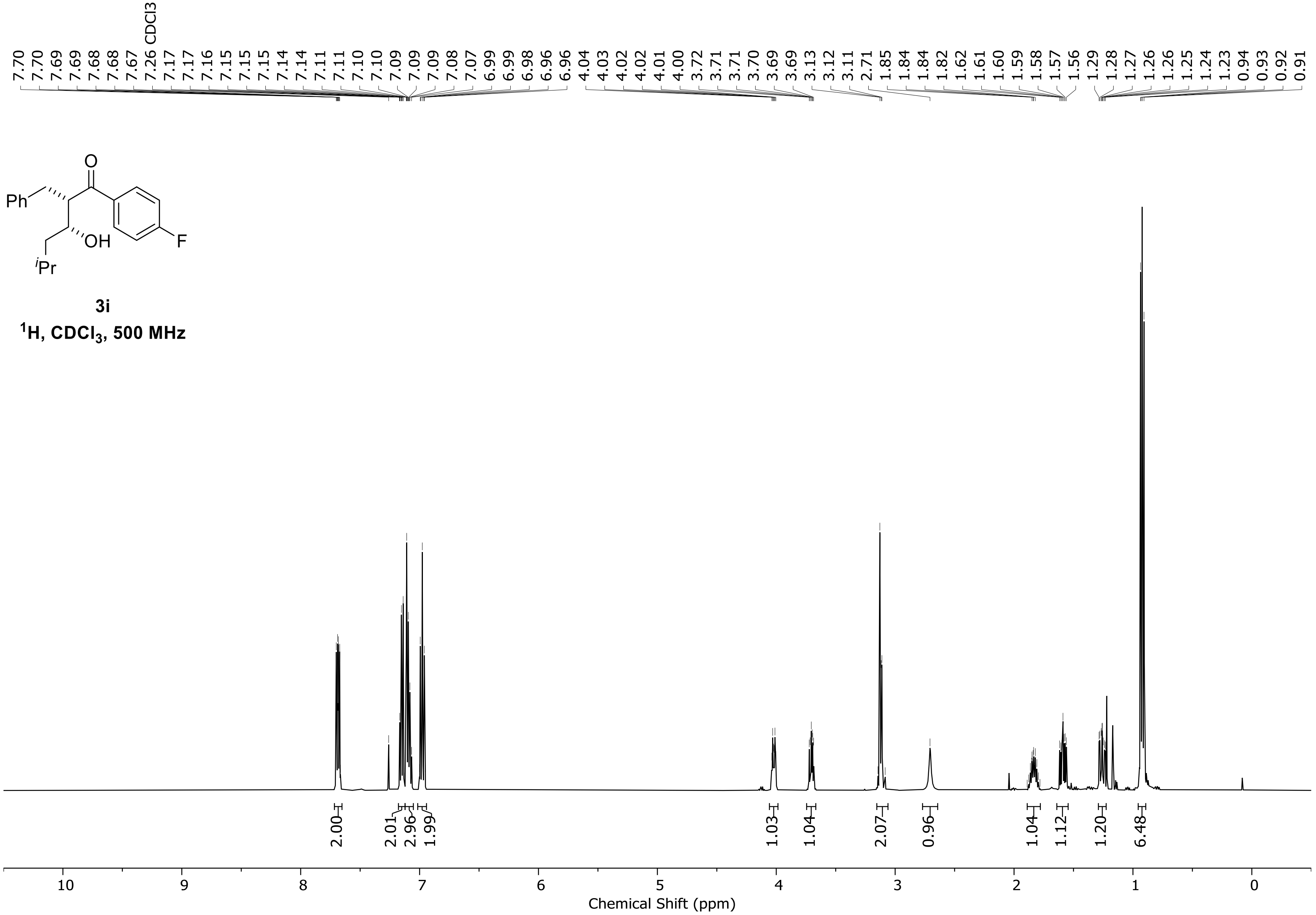

3i

^{1}H, CDCl$_3$, 500 MHz

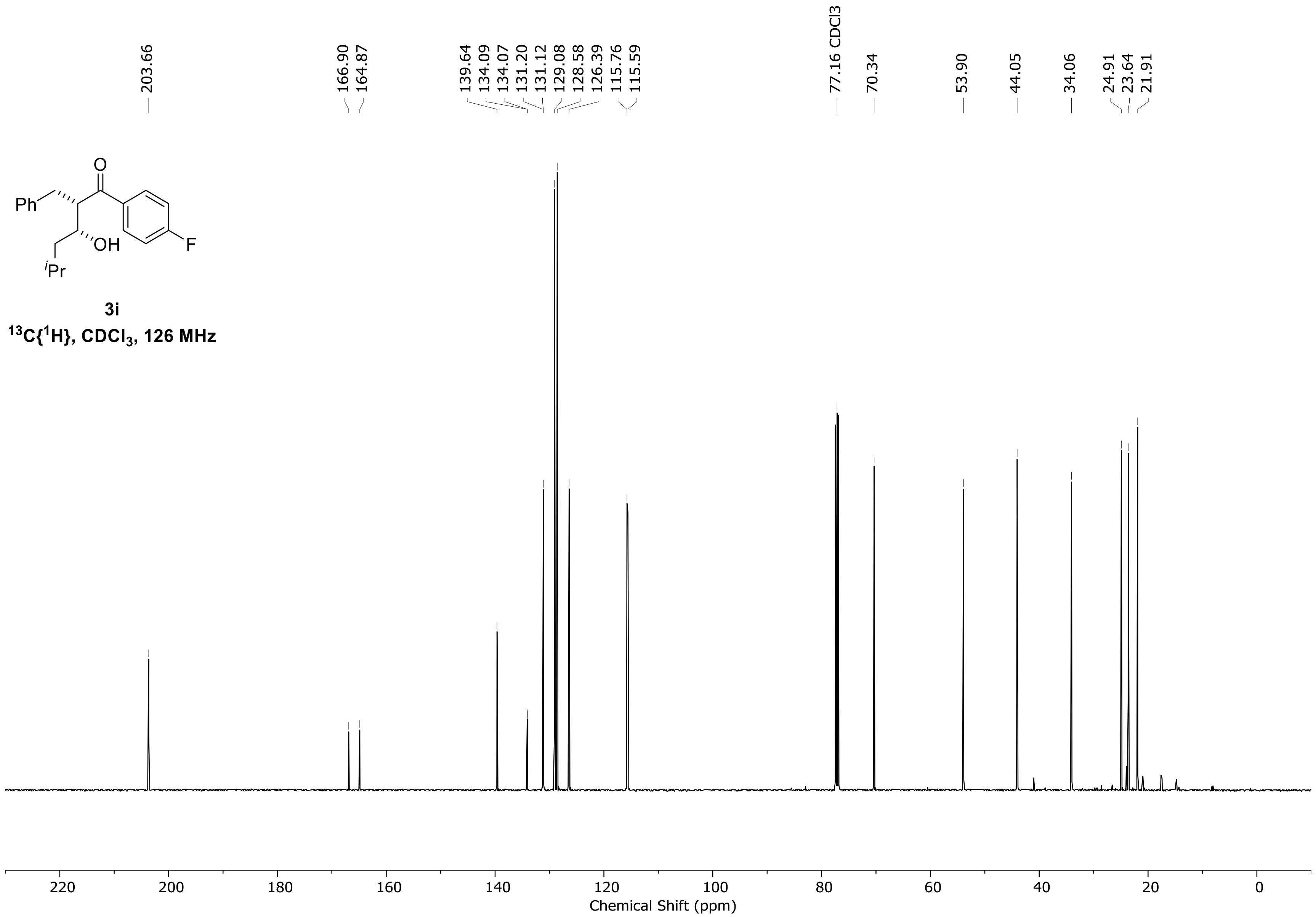

3i
$^{13}C\{^1H\}$, $CDCl_3$, 126 MHz

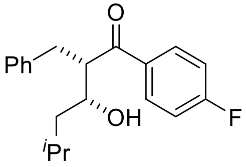

3i
$^{19}F\{^{1}H\}$, $CDCl_3$, 470 MHz

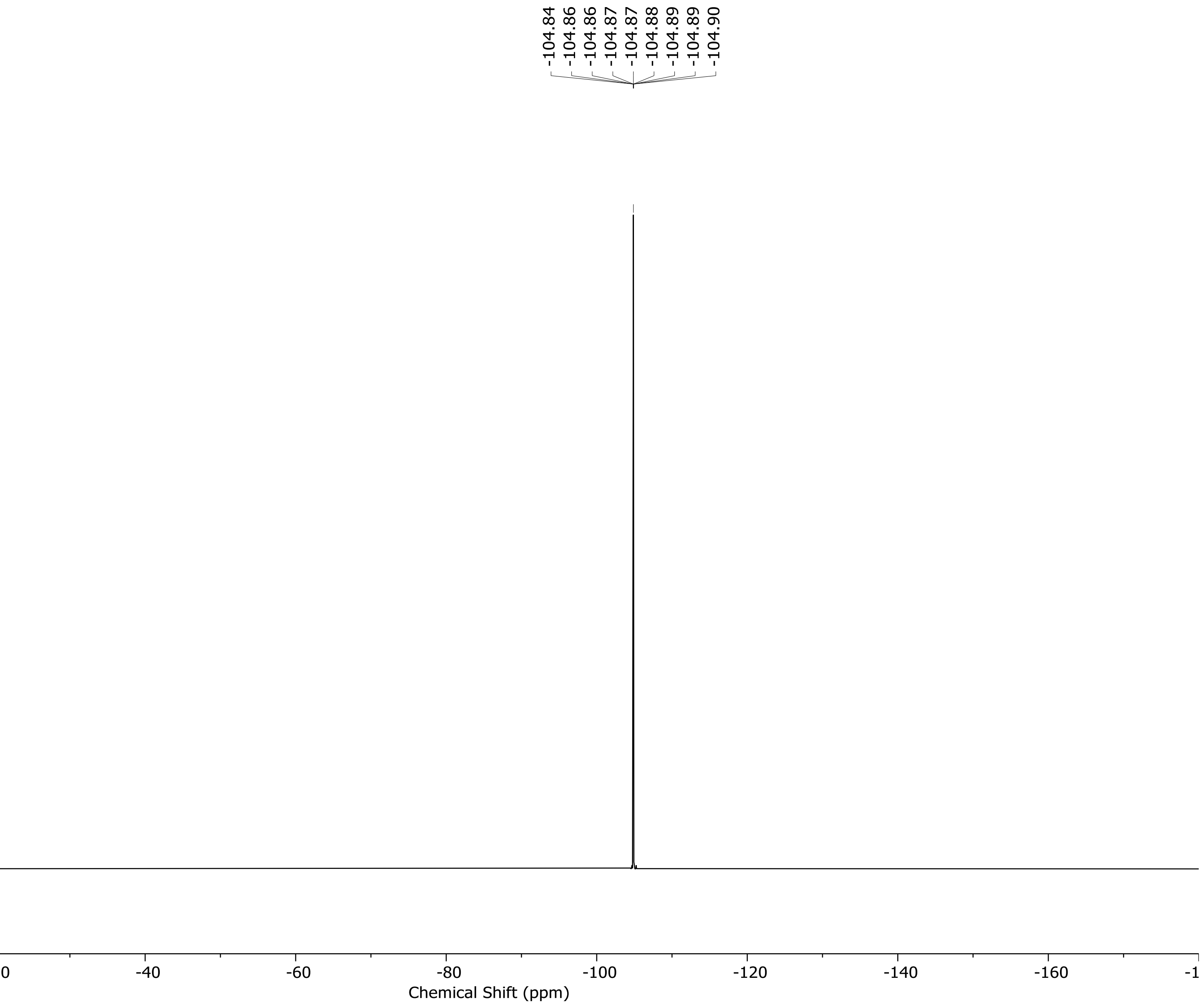

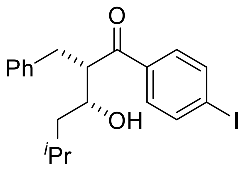

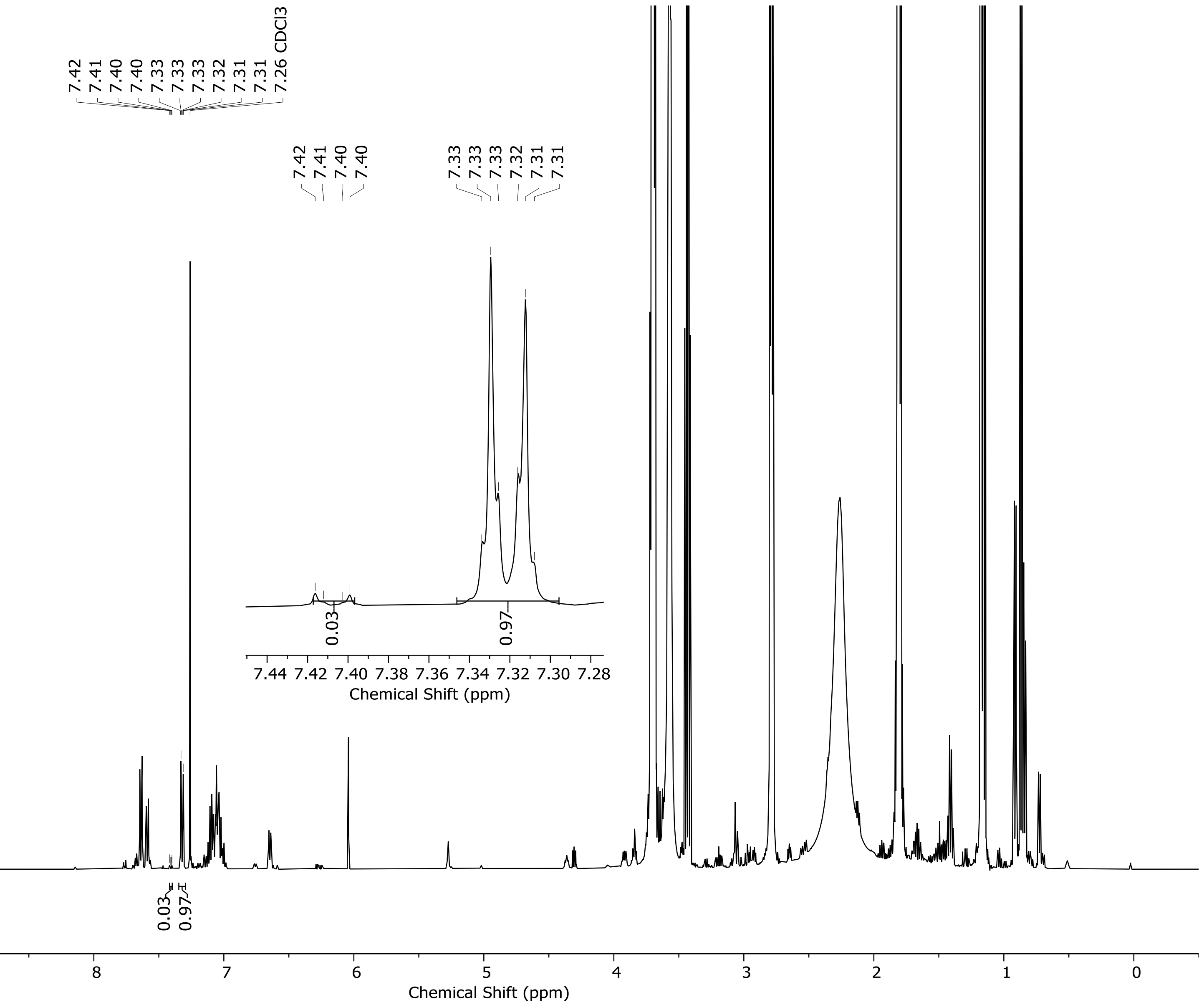

3j

Crude Reaction

^{1}H, $CDCl_3$, 500 MHz

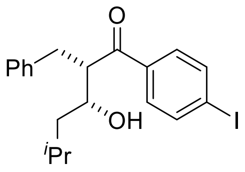

3j

^{1}H, CDCl$_3$, 500 MHz

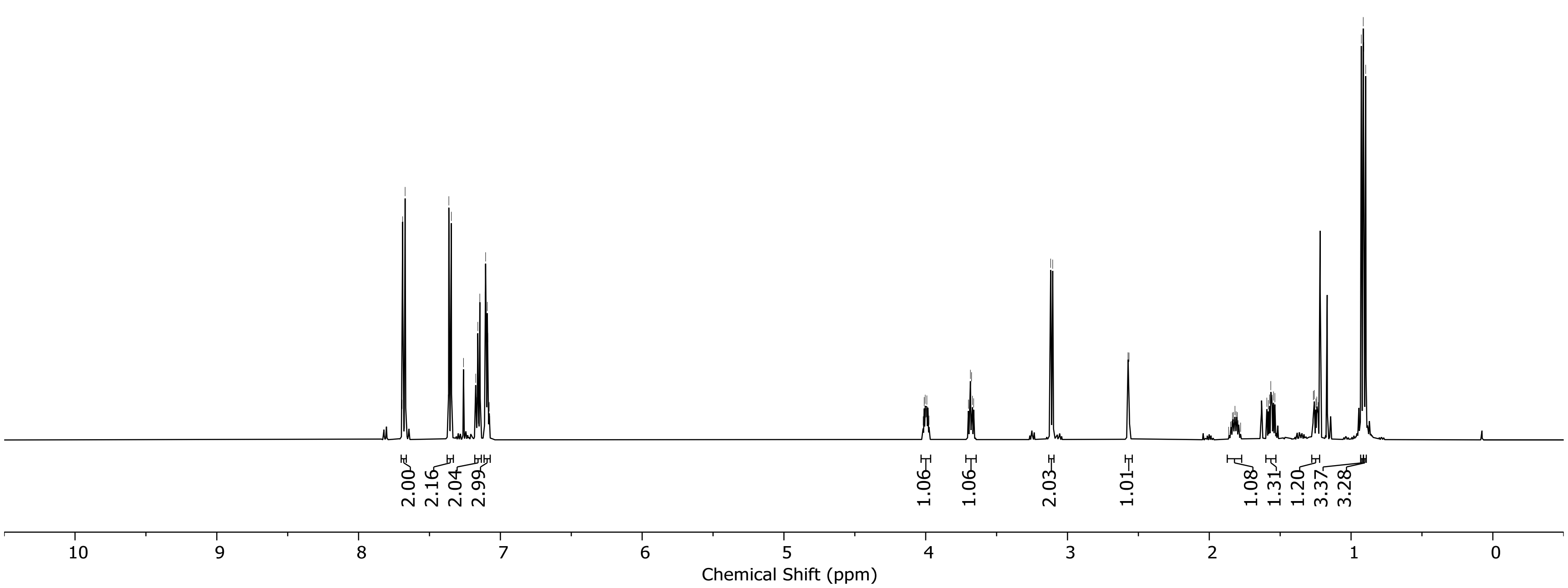

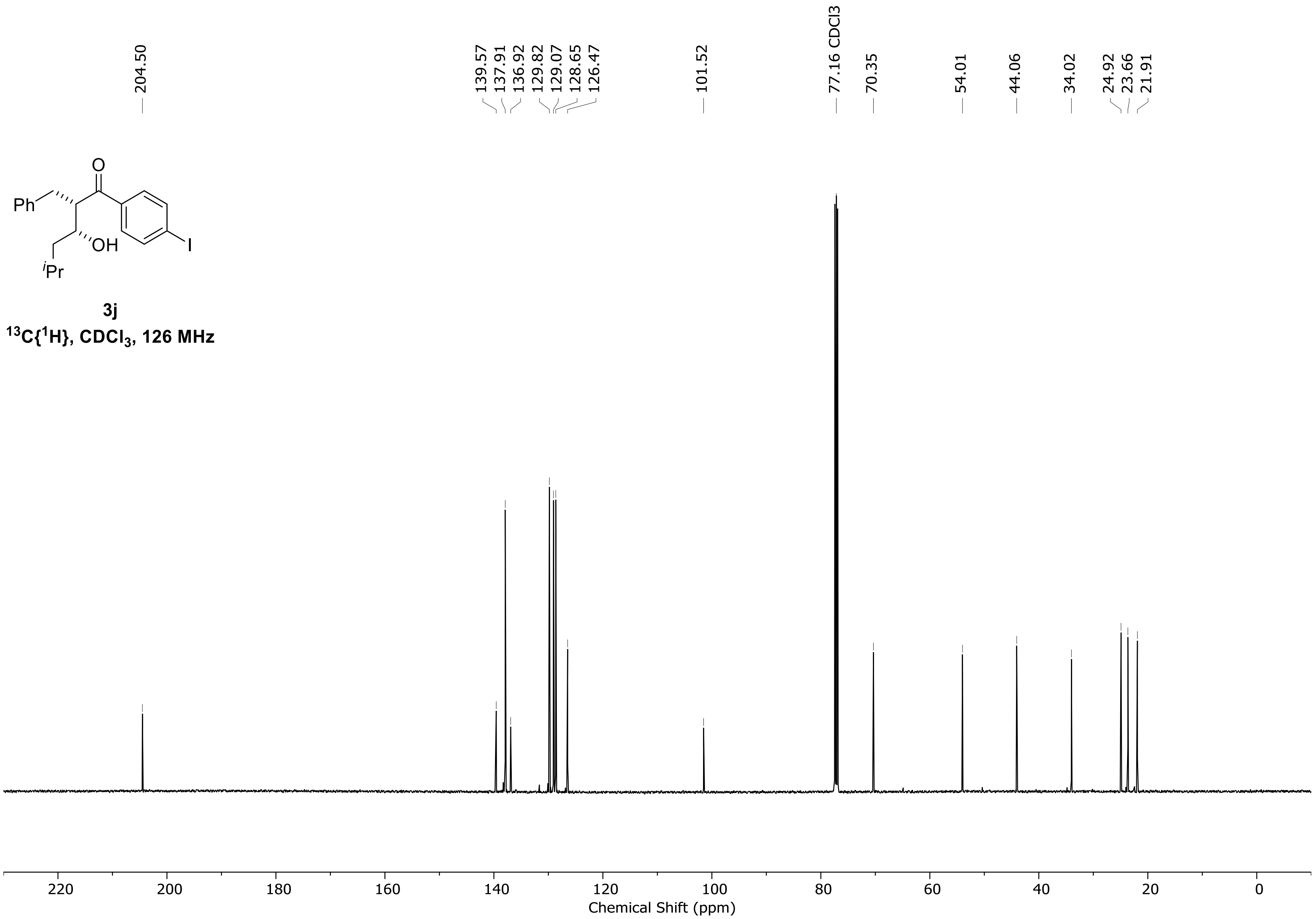

3j

$^{13}C\{^1H\}$, $CDCl_3$, 126 MHz

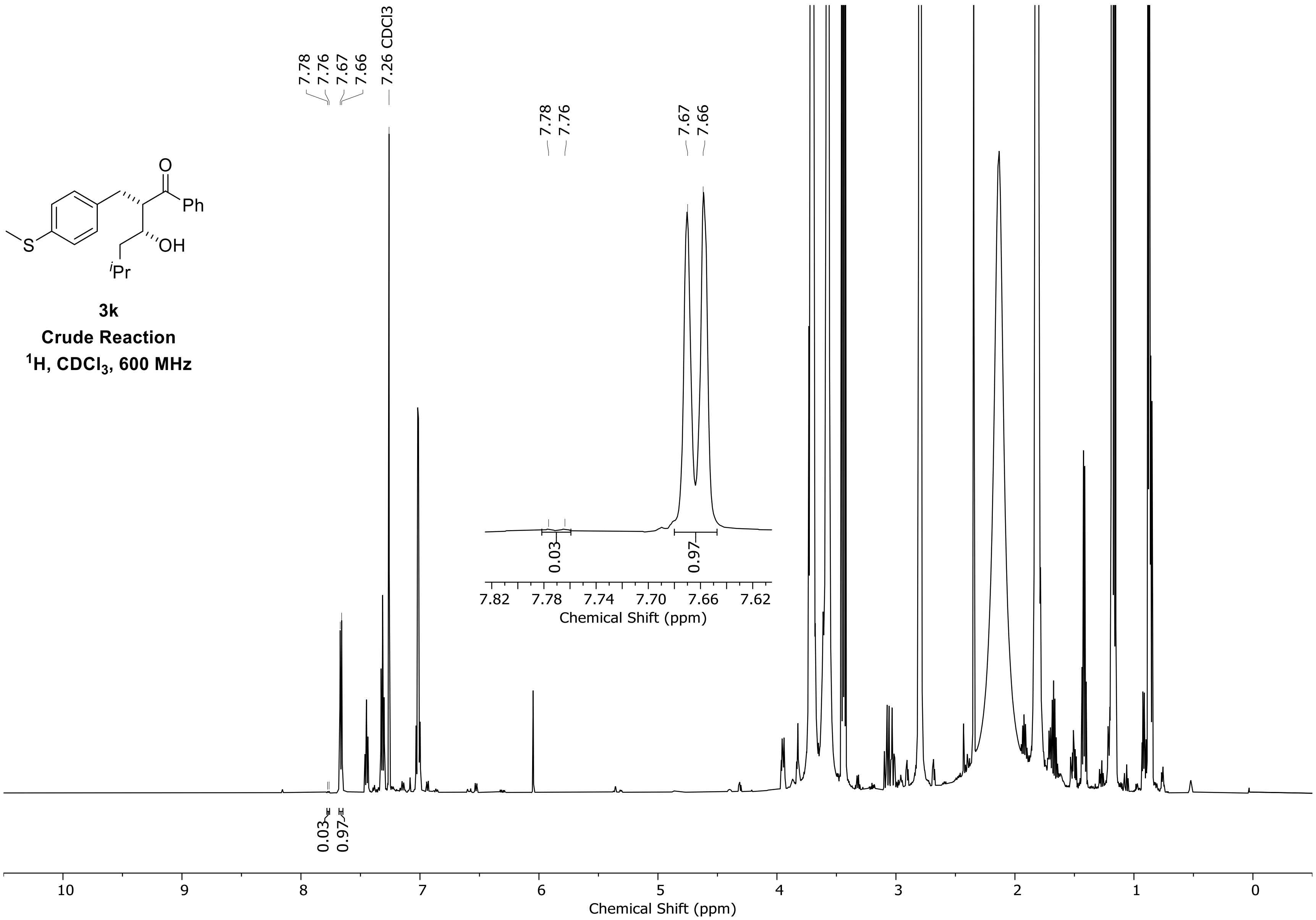
O
Ph
S
OH
iPr
3k
Crude Reaction
1H, CDCl3, 600 MHz
7.78
7.76
7.67
7.66
7.26 CDCl3
0.03
0.97
7.82
7.78
7.74
7.70
7.66
7.62
Chemical Shift (ppm)
10
9
8
7
6
5
4
3
2
1
0
Chemical Shift (ppm)

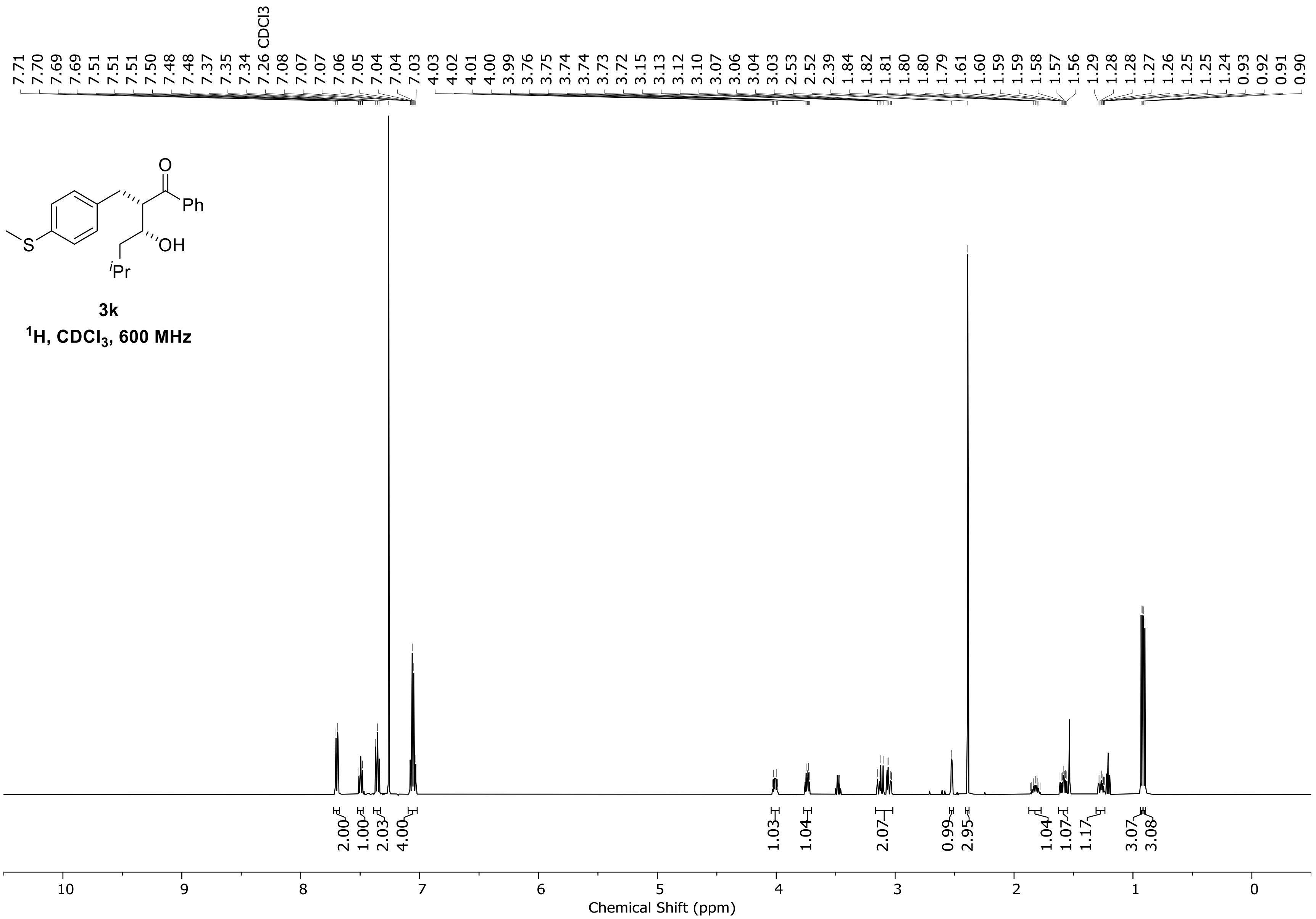
7.71 7.70 7.69 7.69 7.51 7.51 7.51 7.50 7.48 7.48 7.37 7.35 7.34 7.26 CDCl3 7.08 7.07 7.07 7.06 7.05 7.04 7.04 7.03 4.03 4.02 4.01 4.00 3.99 3.76 3.75 3.74 3.74 3.73 3.72 3.15 3.13 3.12 3.10 3.07 3.06 3.04 3.03 2.53 2.52 2.39 1.84 1.82 1.81 1.80 1.80 1.79 1.61 1.60 1.59 1.59 1.58 1.57 1.56 1.29 1.28 1.28 1.27 1.26 1.25 1.25 1.24 0.93 0.92 0.91 0.90
O
Ph
S
OH
^{i}Pr
3k
^{1}H, $CDCl_3$, 600 MHz
2.00 1.00 2.03 4.00 1.03 1.04 2.07 0.99 2.95 1.04 1.07 1.17 3.07 3.08
10 9 8 7 6 5 4 3 2 1 0
Chemical Shift (ppm)

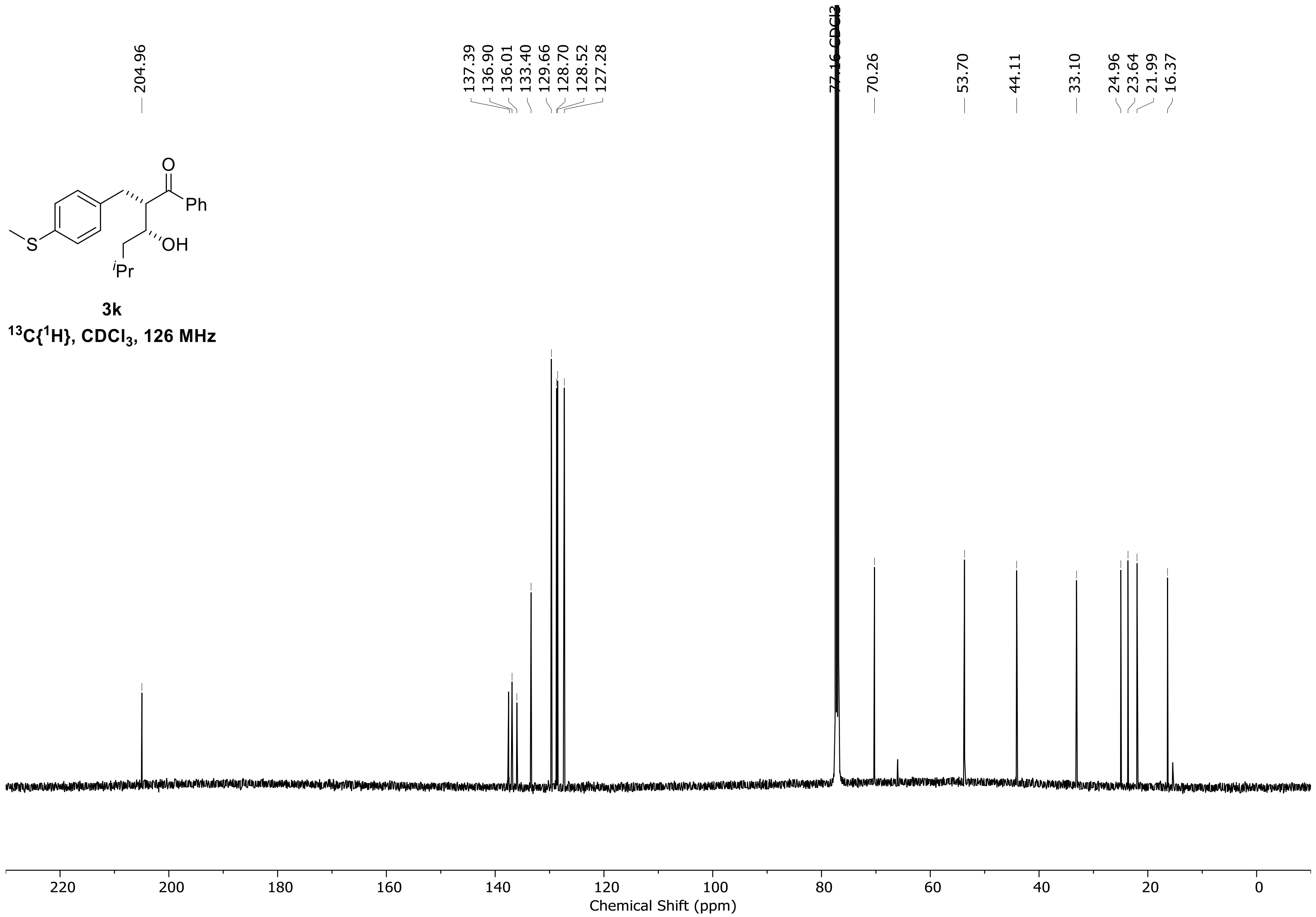

3k

$^{13}C\{^{1}H\}$, $CDCl_3$, 126 MHz

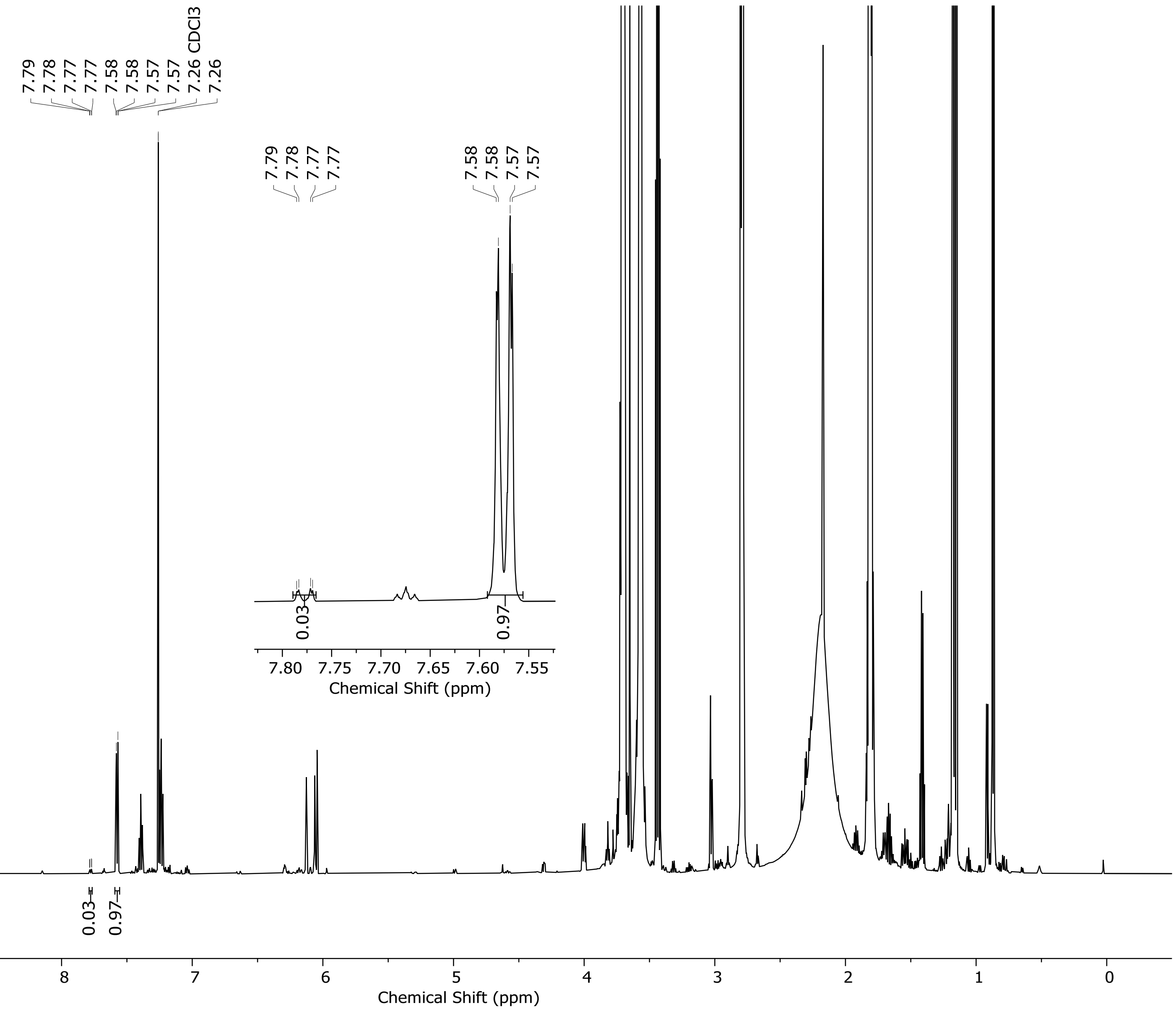

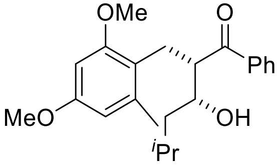

3l
Crude Reaction
^{1}H, $CDCl_3$, 600 MHz

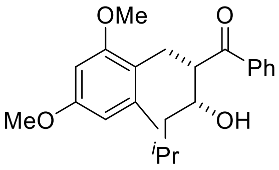

3l
^{1}H, $CDCl_3$, 500 MHz

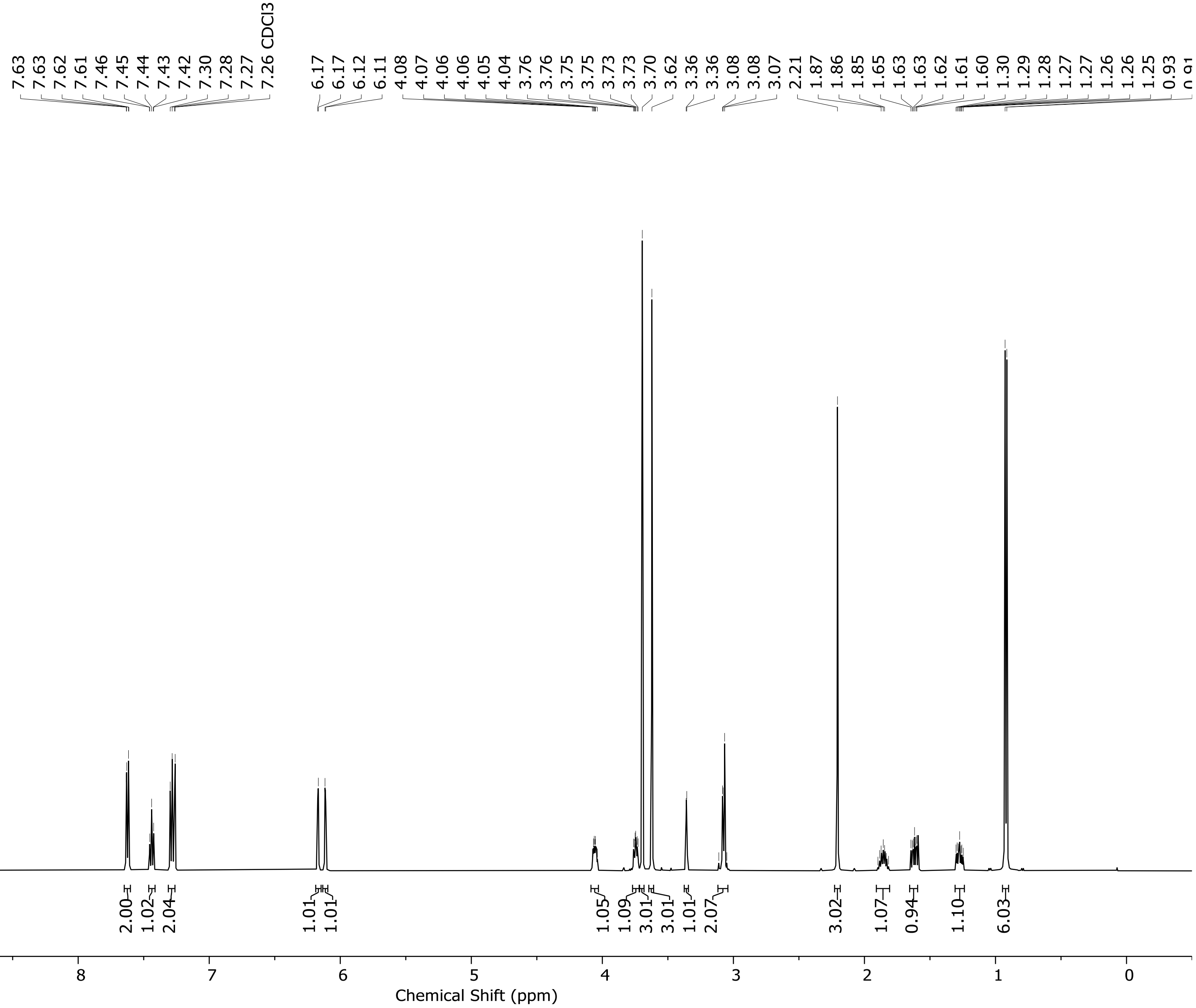

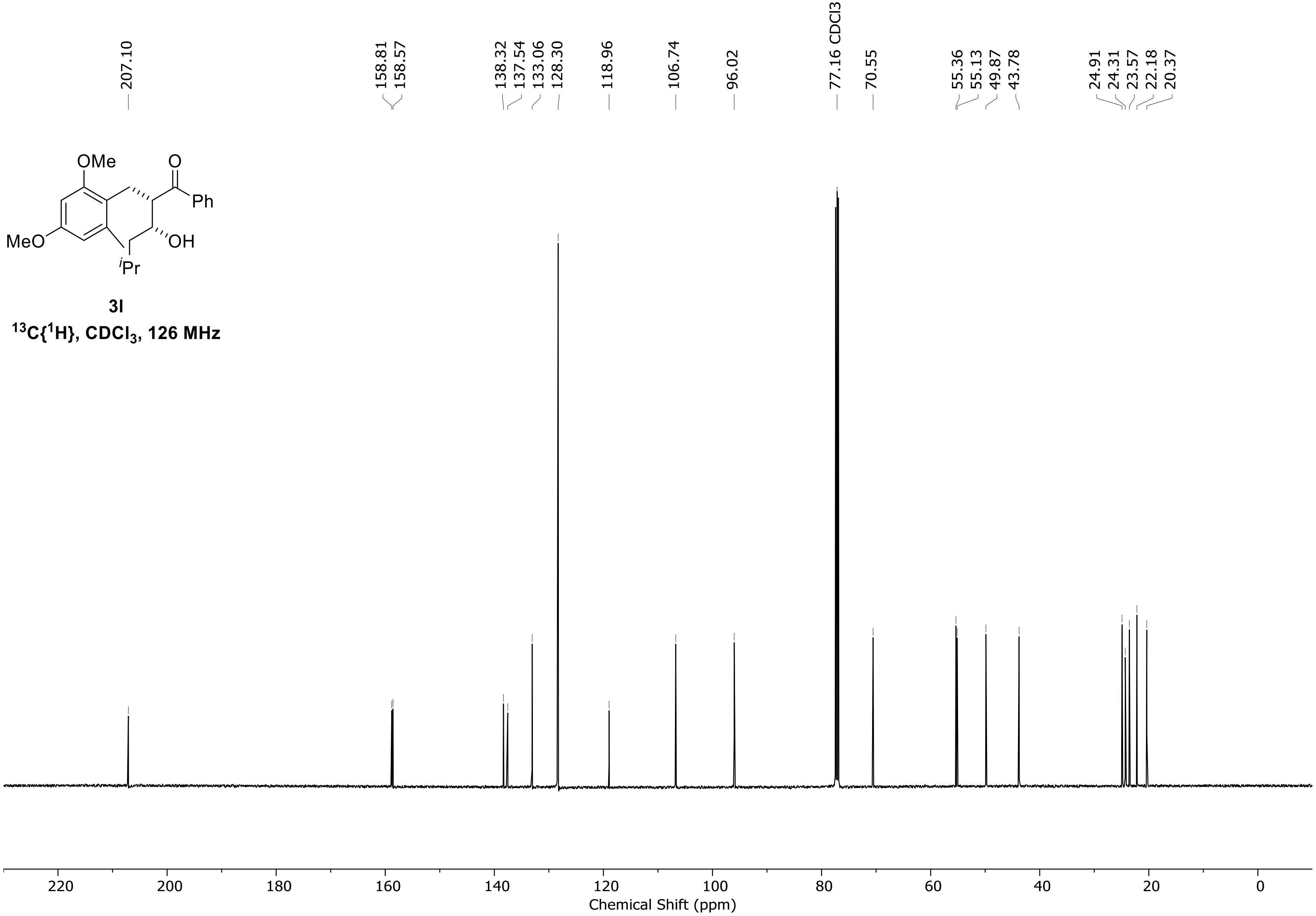
207.10
158.81
158.57
138.32
137.54
133.06
128.30
118.96
106.74
96.02
77.16 CDCl3
70.55
55.36
55.13
49.87
43.78
24.91
24.31
23.57
22.18
20.37
OMe
O
Ph
MeO
OH
iPr
3l
13C{1H}, CDCl3, 126 MHz
220
200
180
160
140
120
100
80
60
40
20
0
Chemical Shift (ppm)

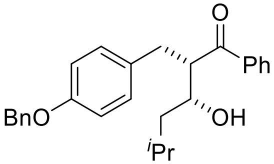

3m
Crude Reaction
^{1}H, $CDCl_3$, 600 MHz

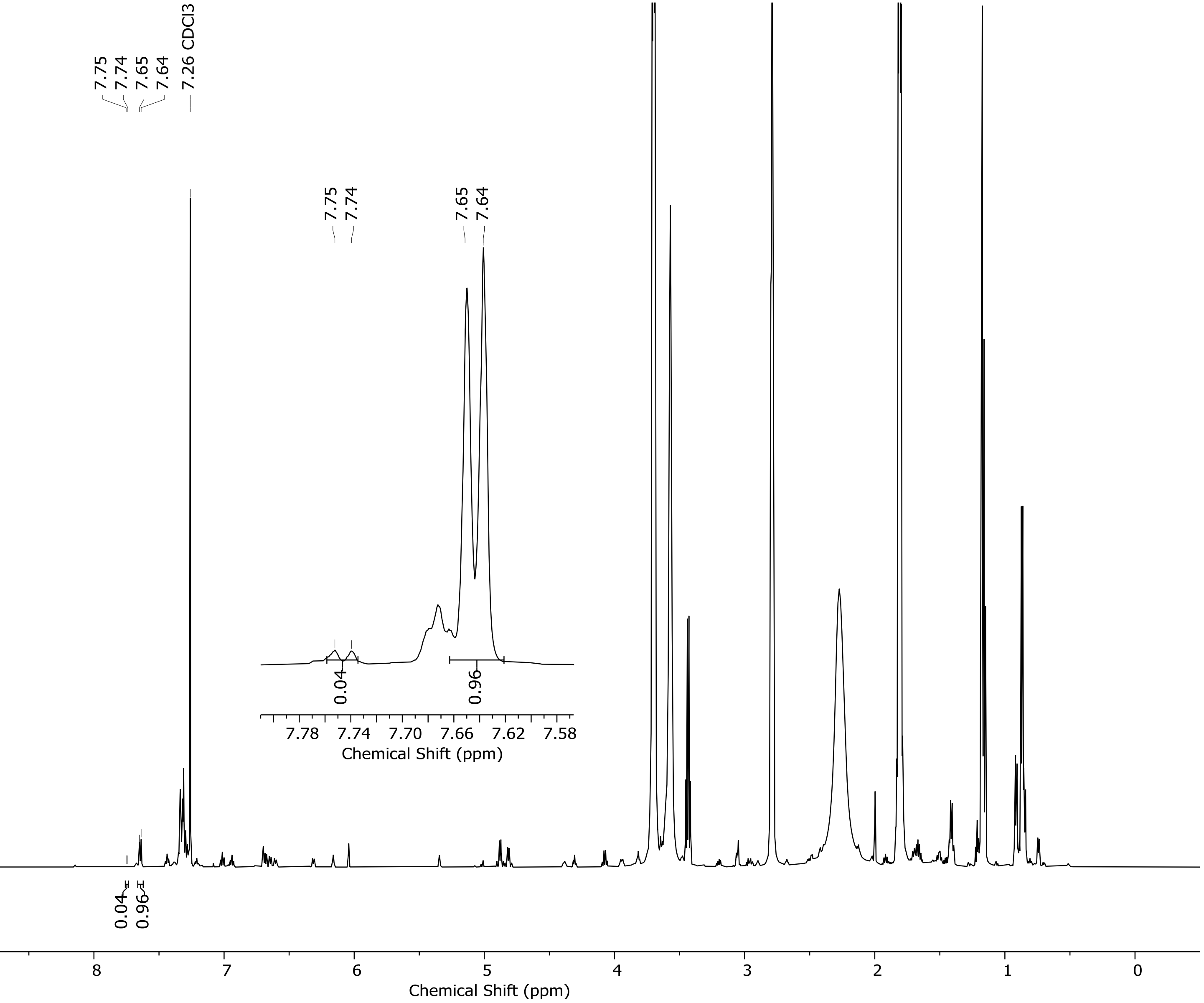

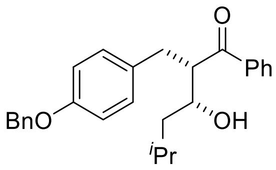

3m

^{1}H, CDCl$_3$, 500 MHz

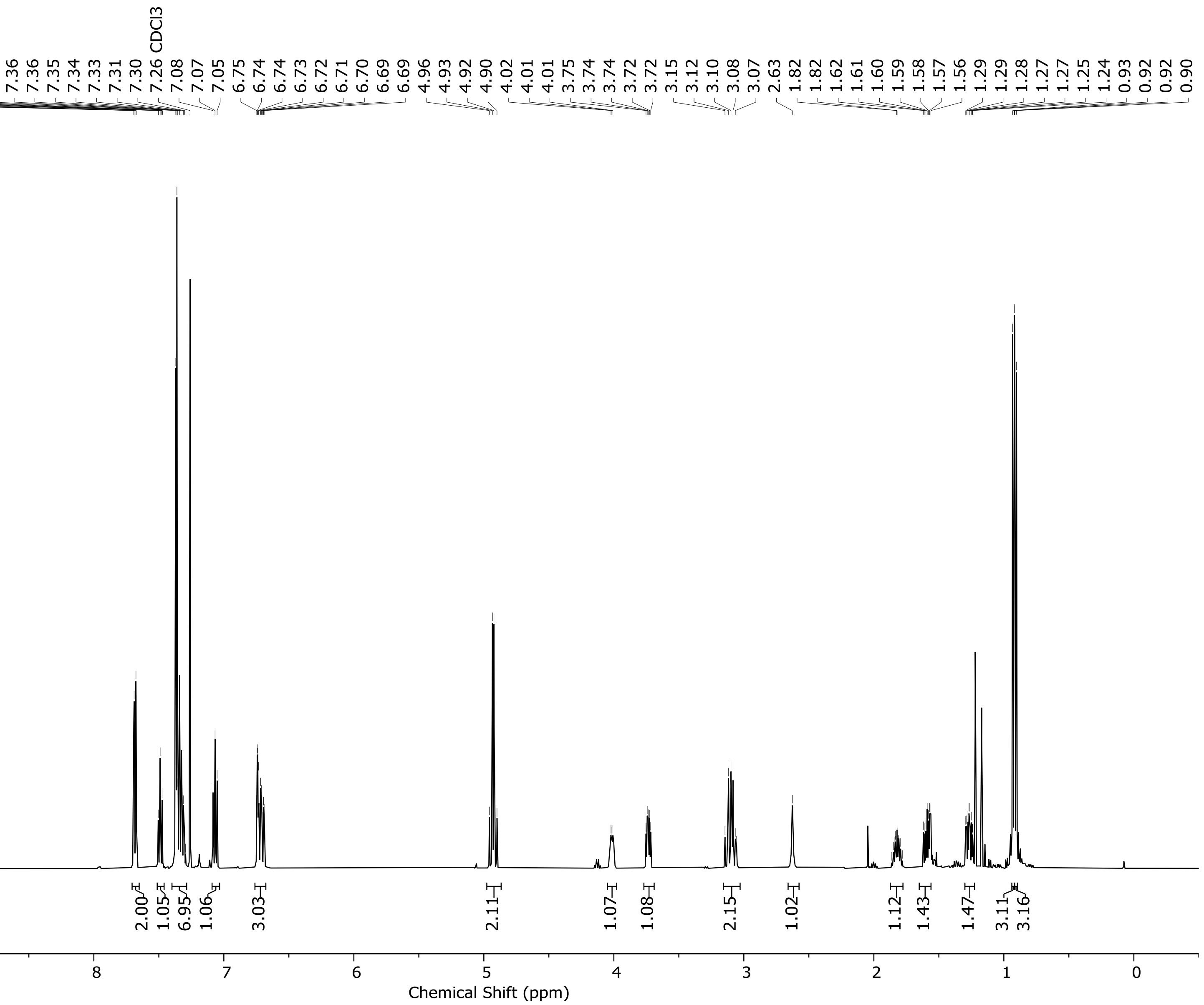

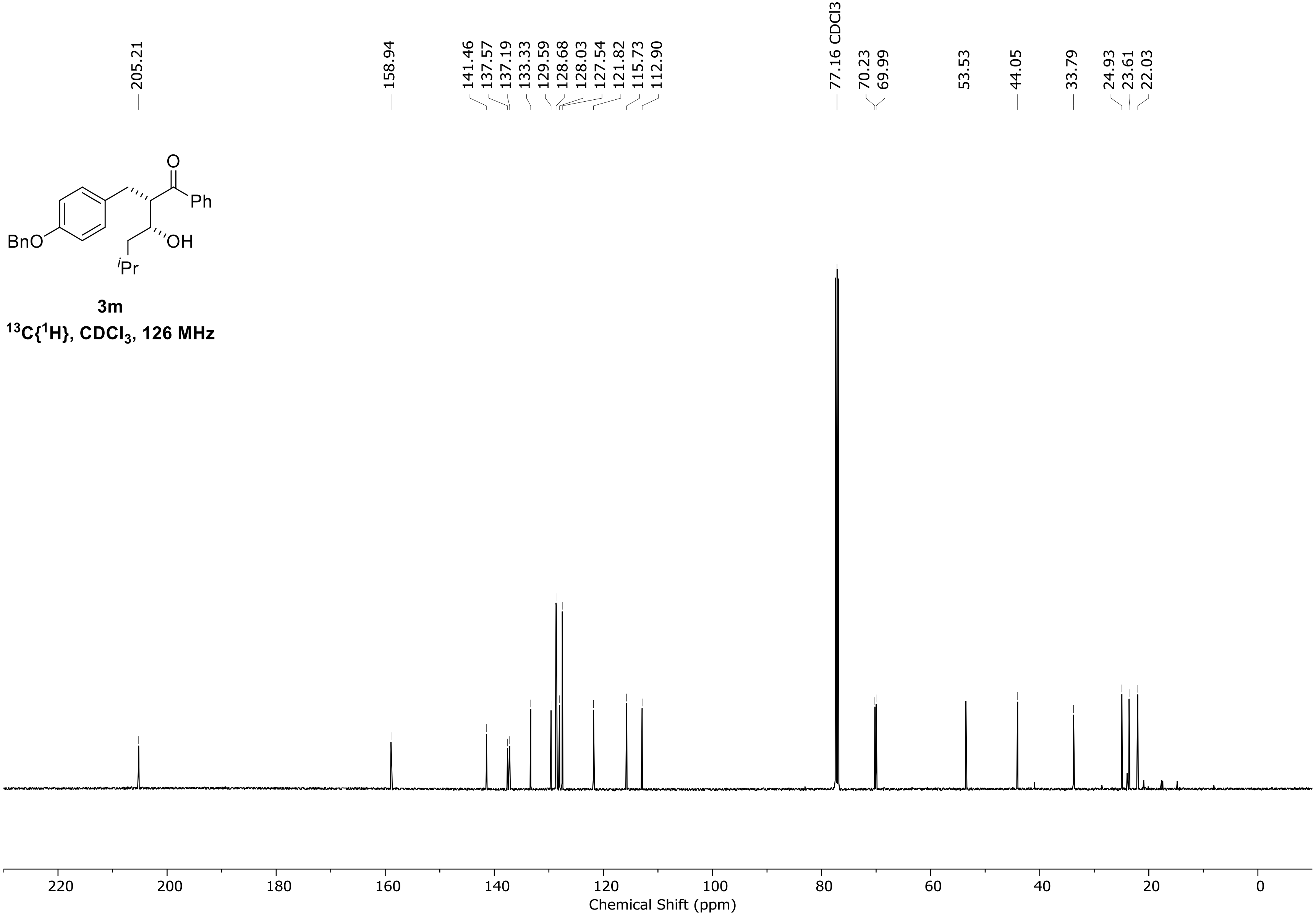

3m

$^{13}C\{^1H\}$, $CDCl_3$, 126 MHz

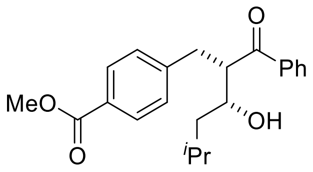

3n
Crude Reaction
^{1}H, CDCl$_3$, 400 MHz

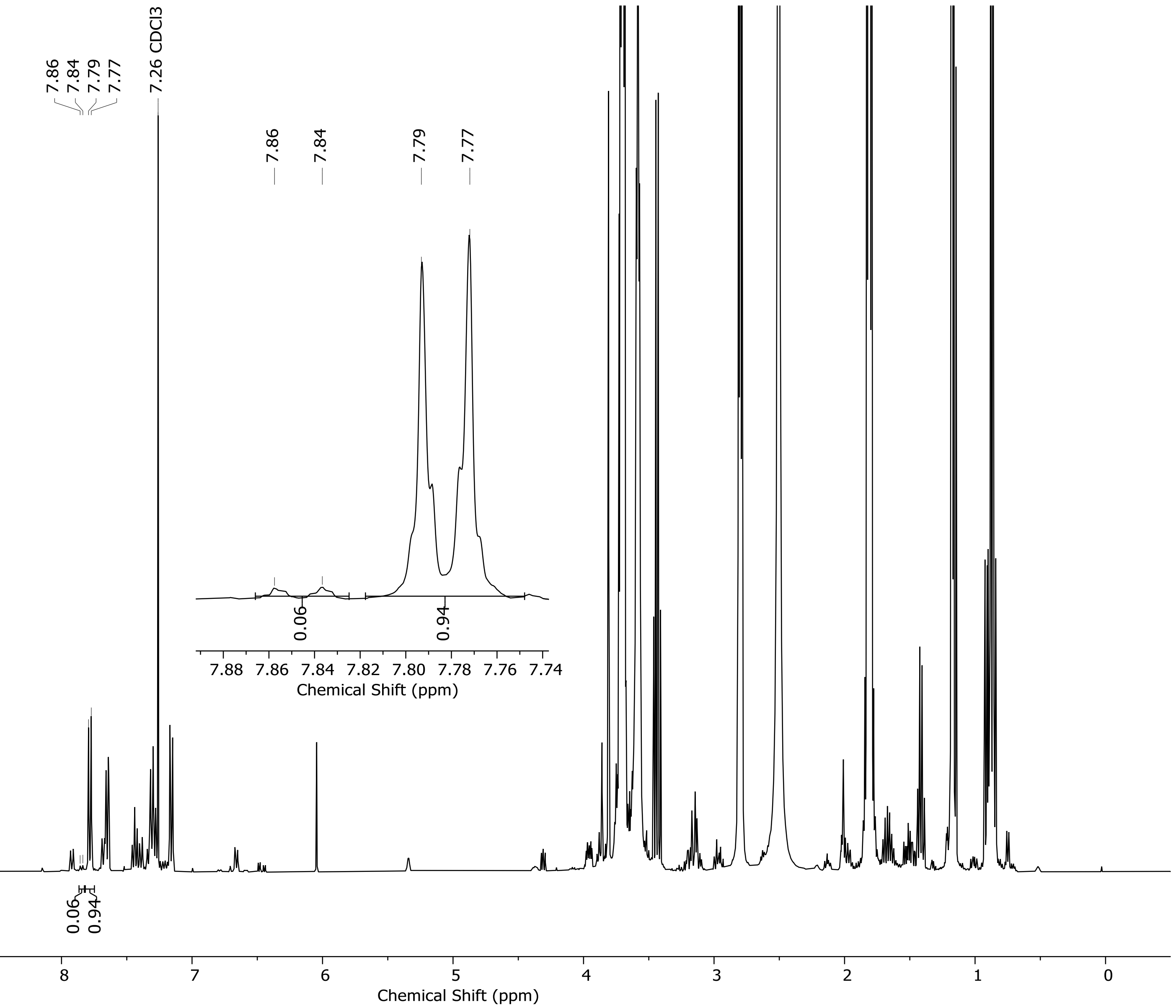

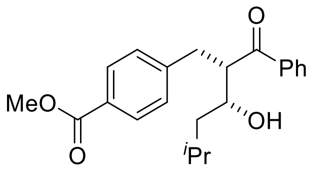

3n
^{1}H, CDCl$_3$, 600 MHz

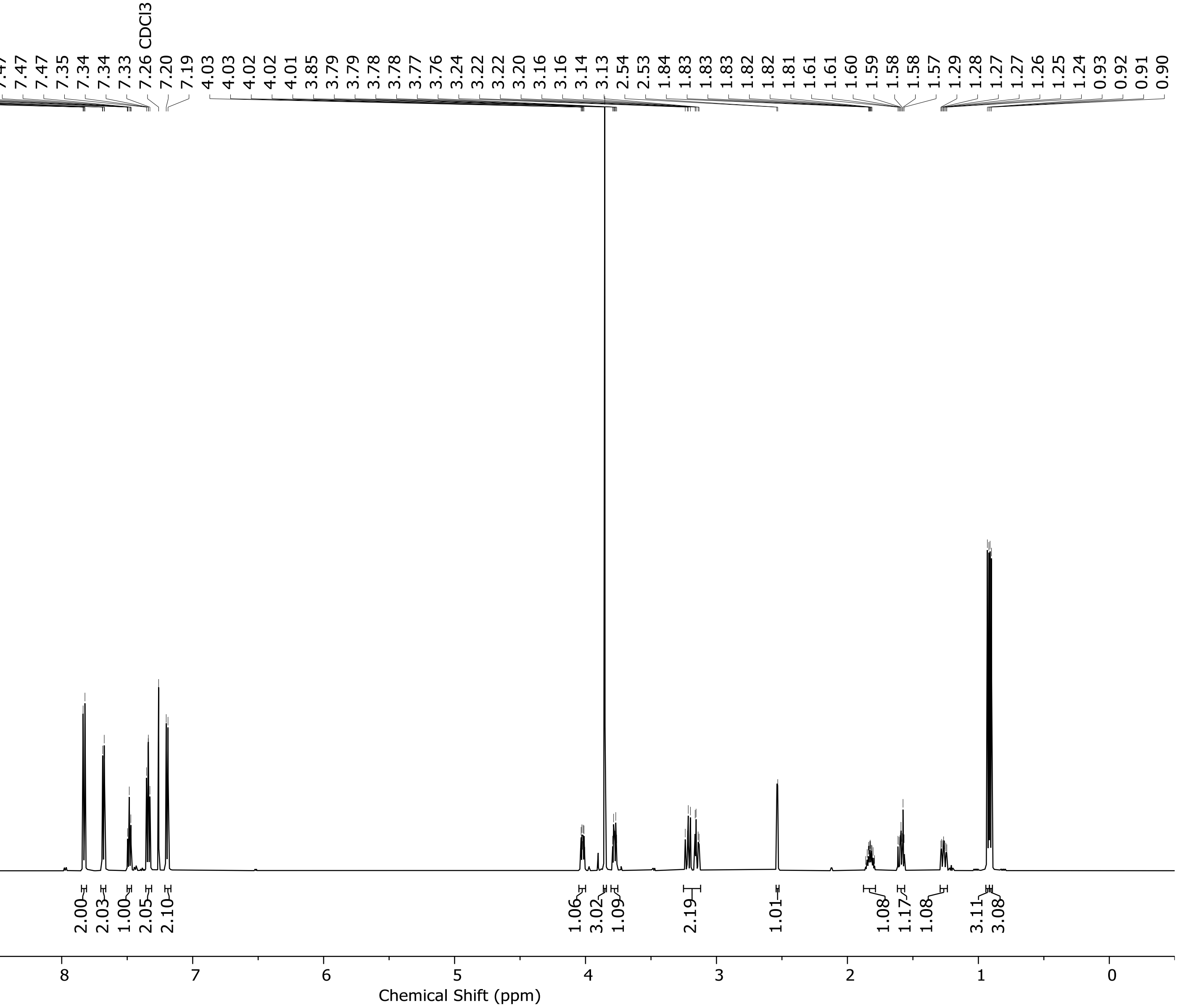

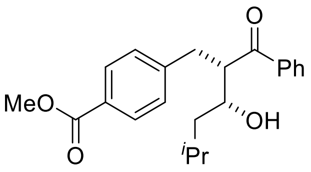

3n

$^{13}C\{^1H\}$, $CDCl_3$, 126 MHz

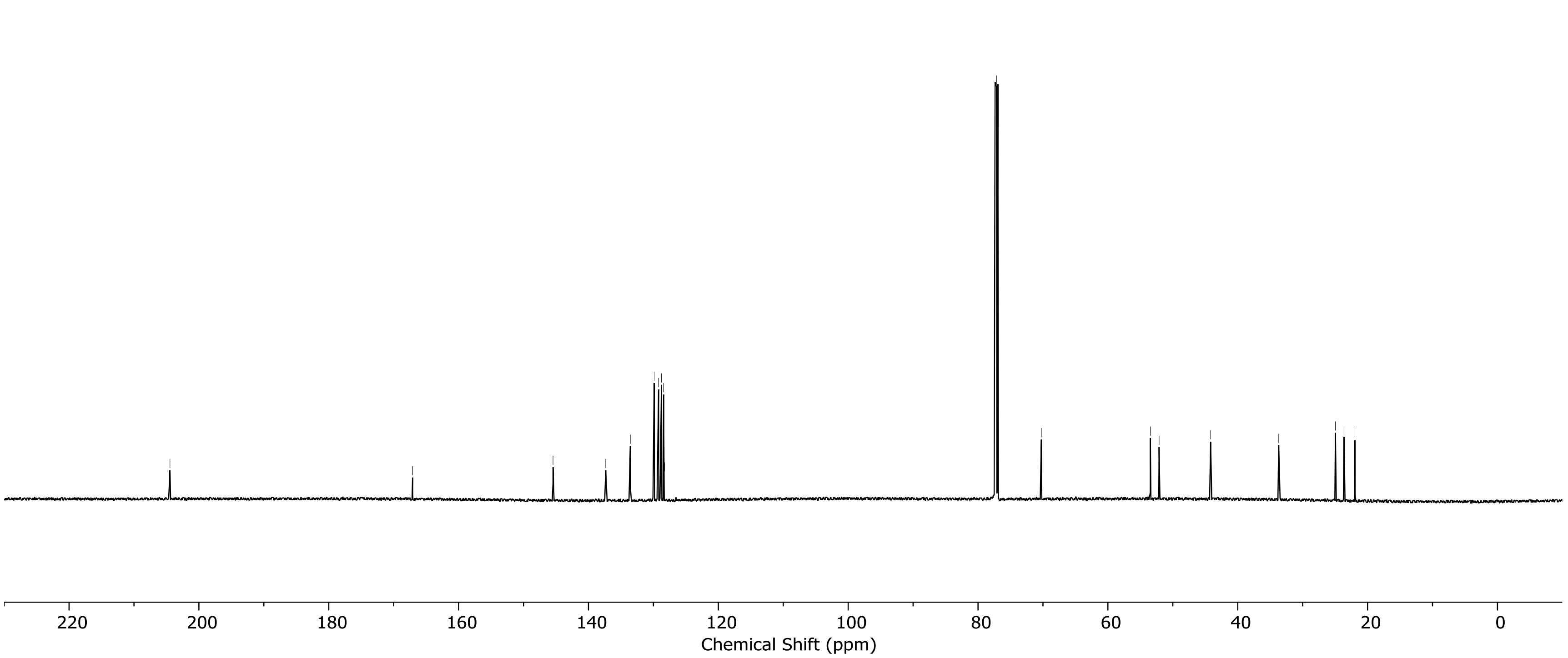

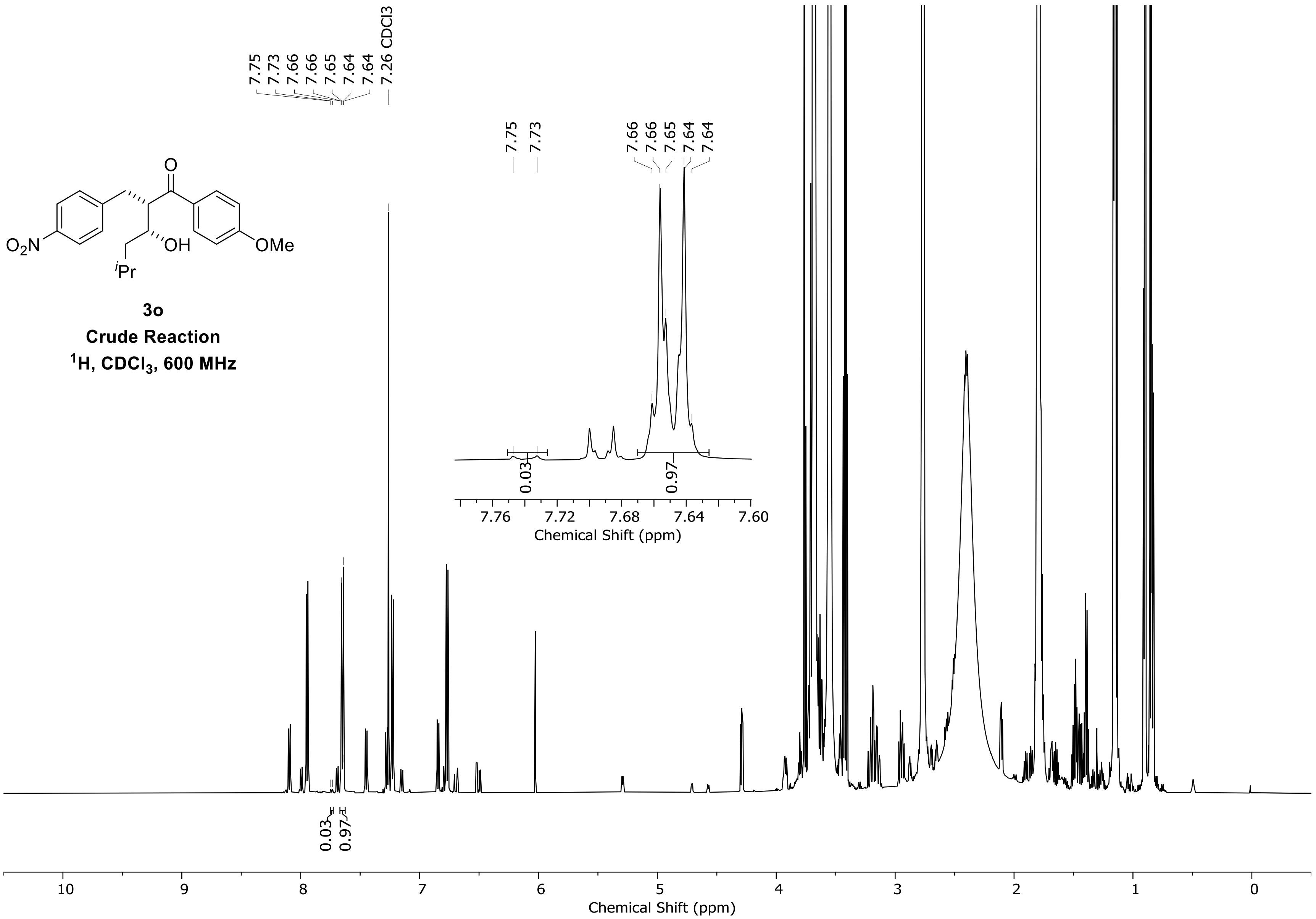

3o
Crude Reaction
^{1}H, $CDCl_3$, 600 MHz

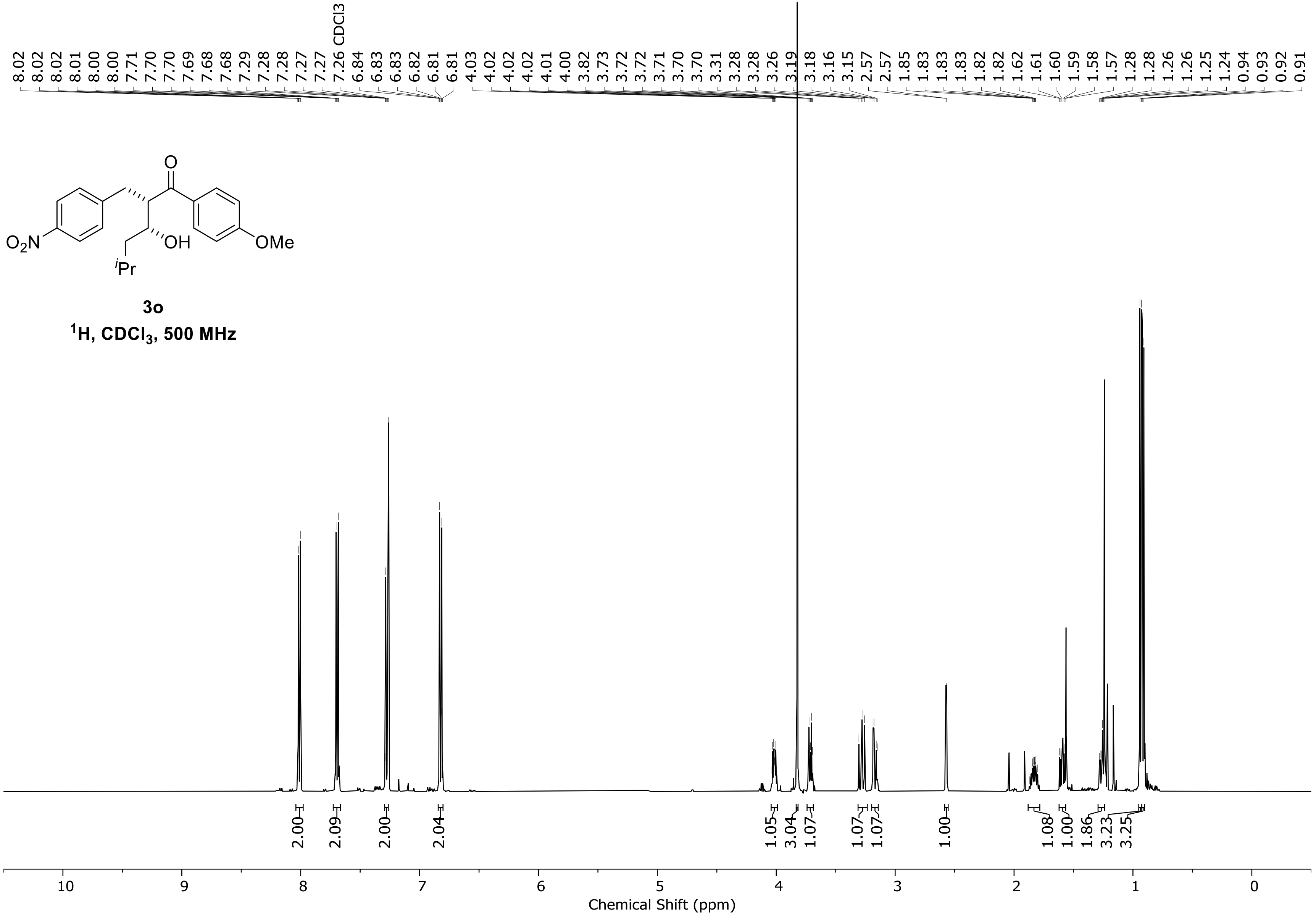

3o

^{1}H, $CDCl_3$, 500 MHz

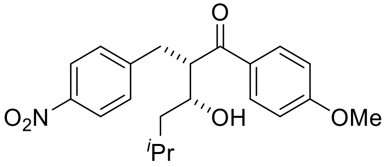

3o
$^{13}C\{^1H\}$, $CDCl_3$, 126 MHz

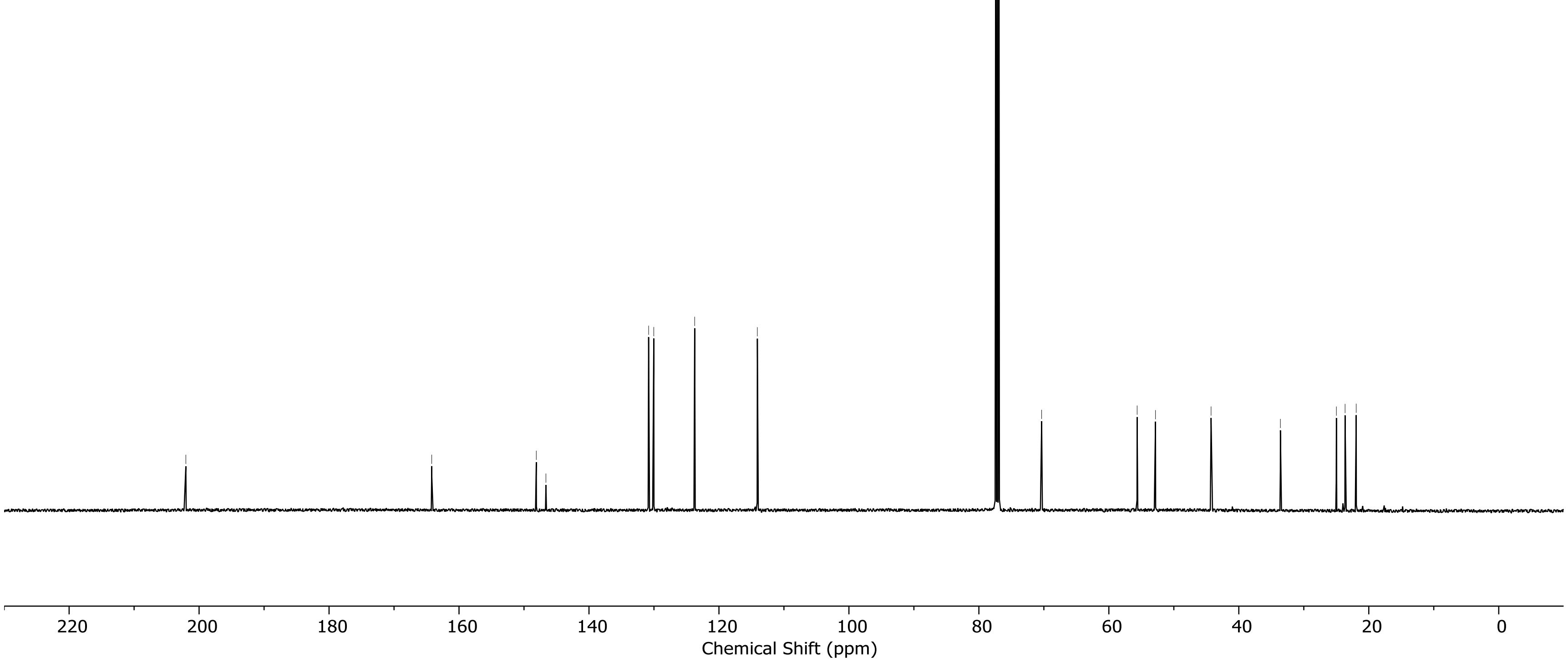

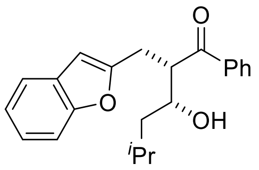

3p
Crude Reaction
^{1}H, $CDCl_3$, 400 MHz

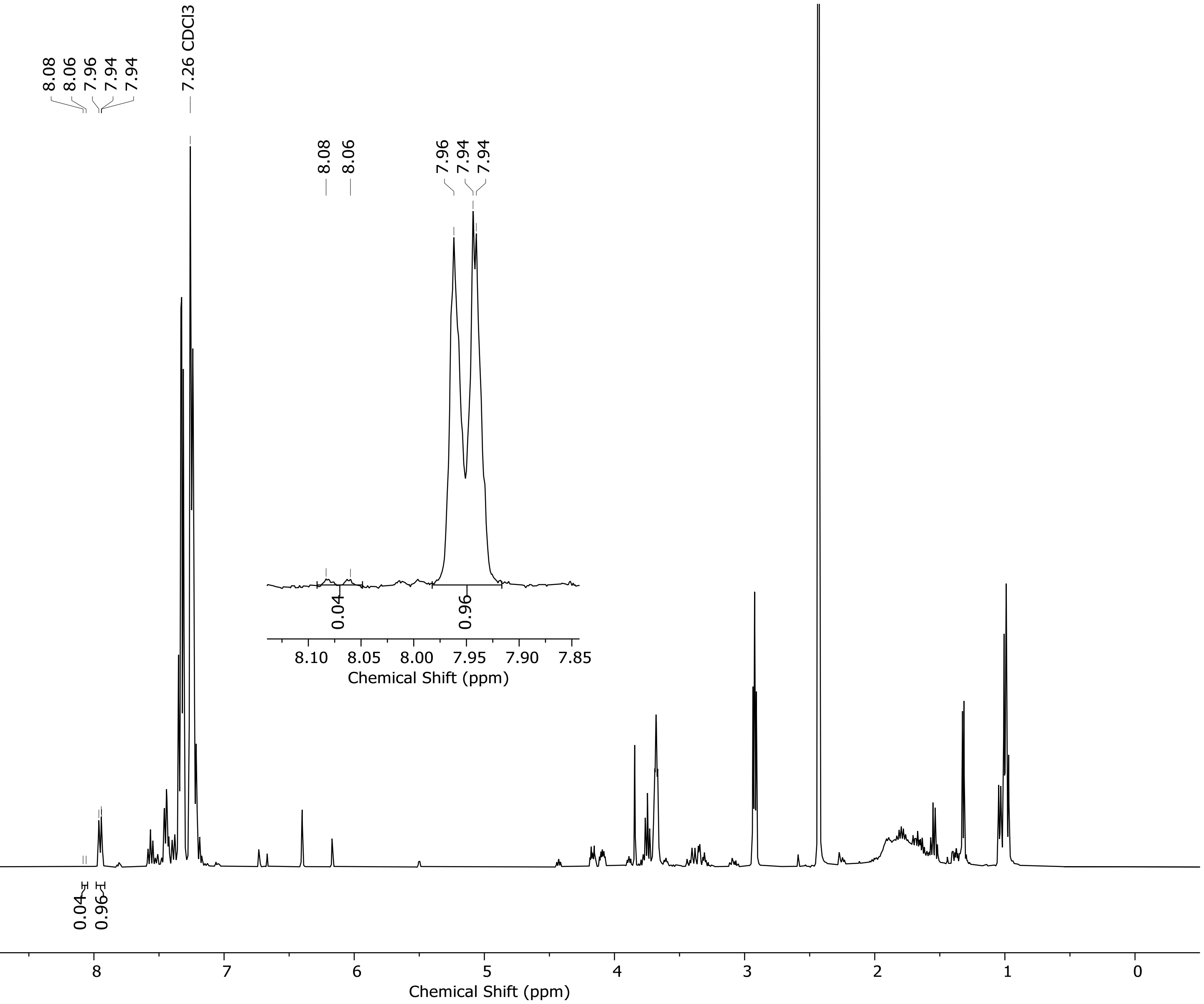

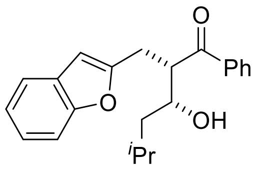

3p

^{1}H, CDCl$_3$, 500 MHz

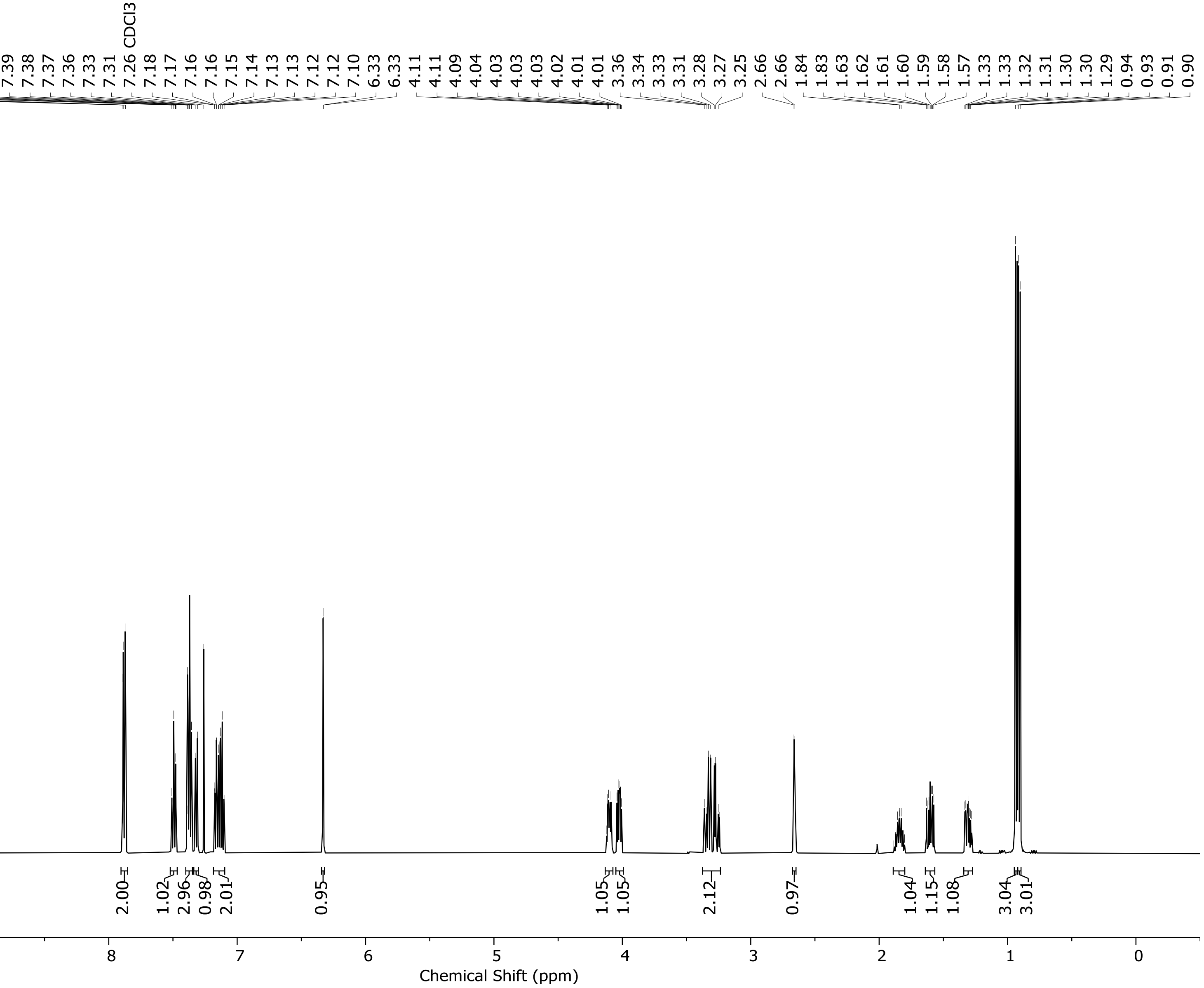

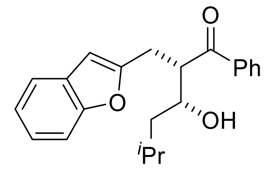

3p

$^{13}C\{^{1}H\}$, $CDCl_3$, 126 MHz

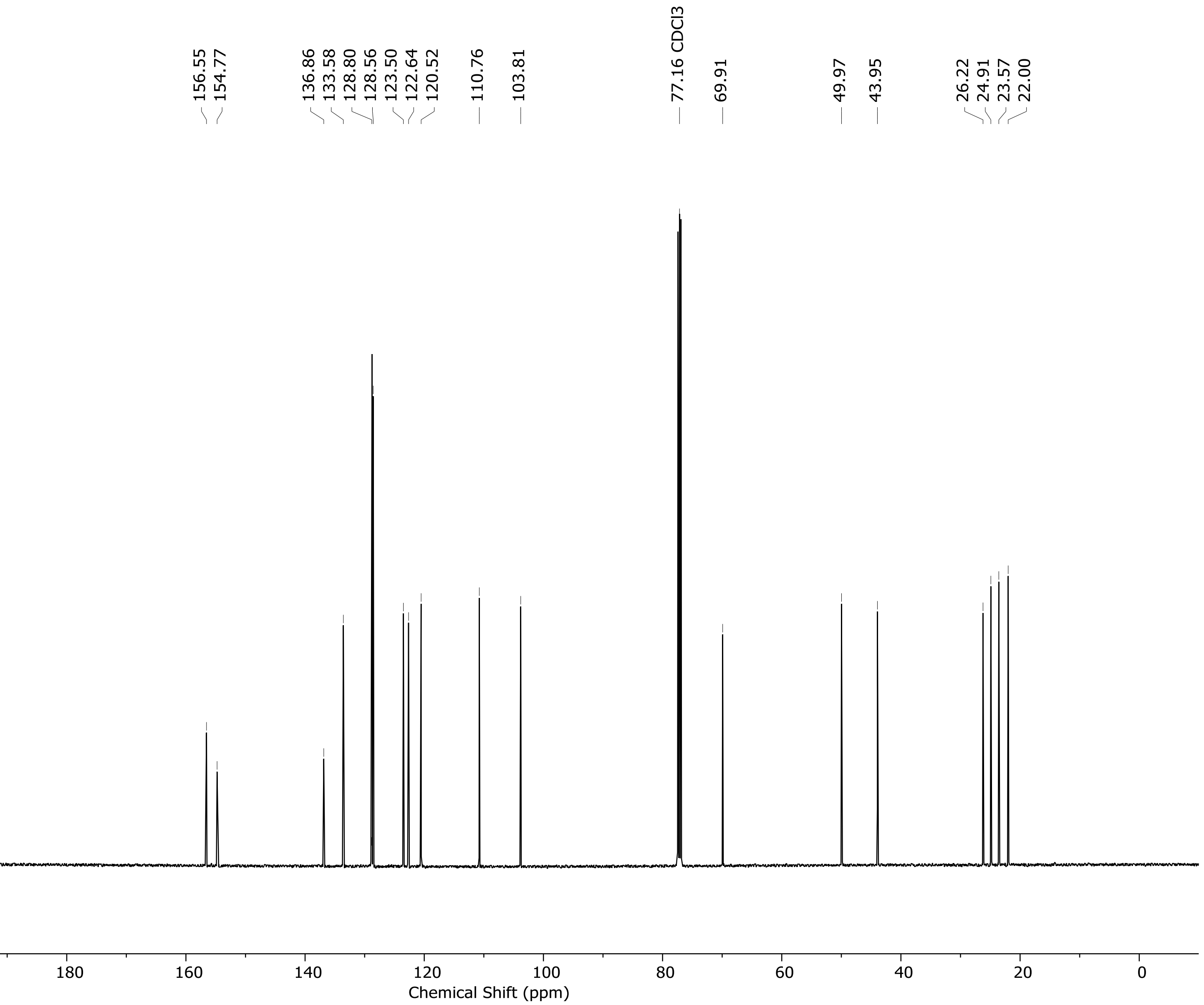

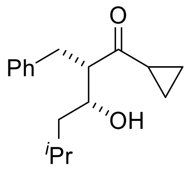

3q
Crude Reaction
^{1}H, $CDCl_3$, 400 MHz

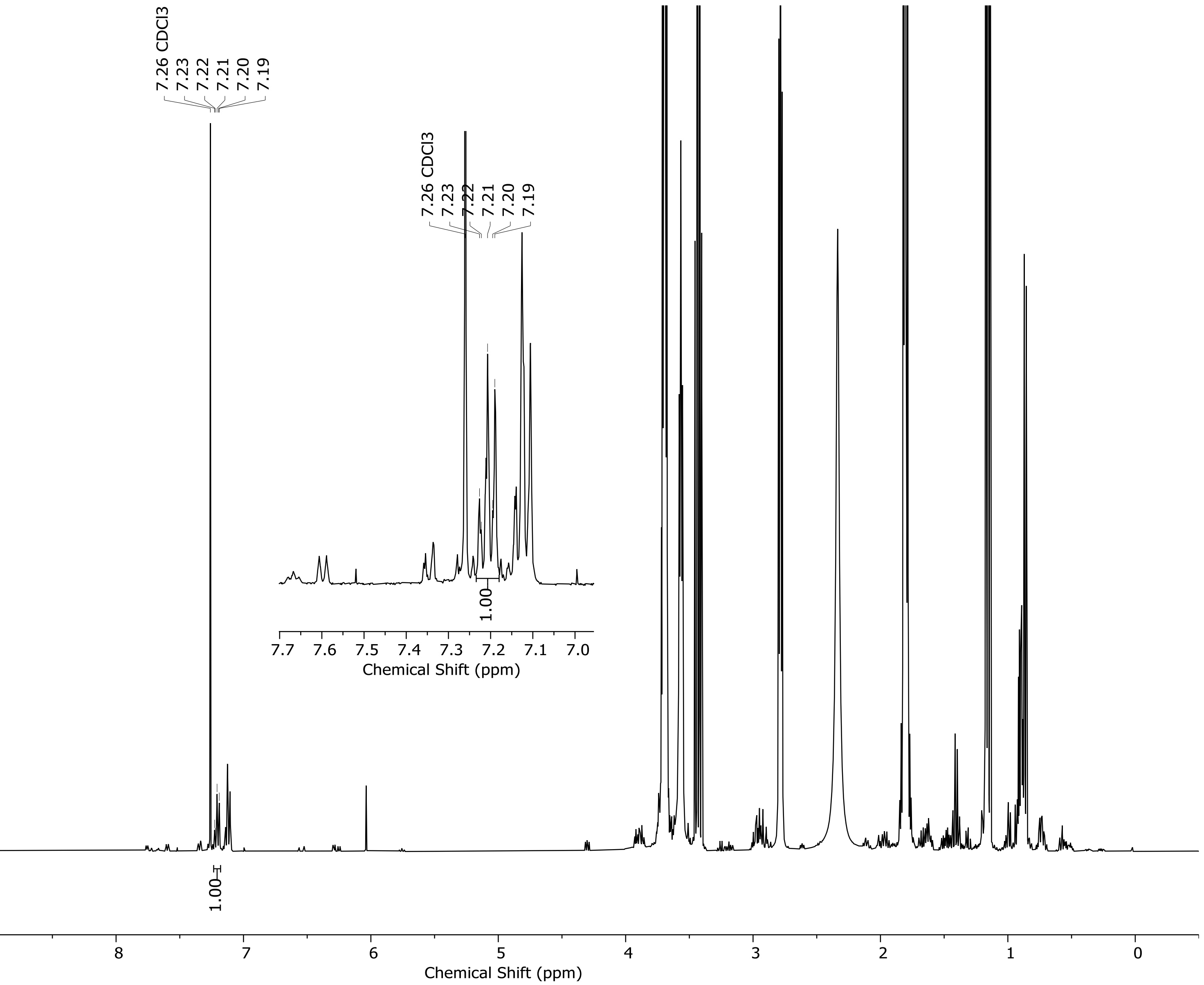

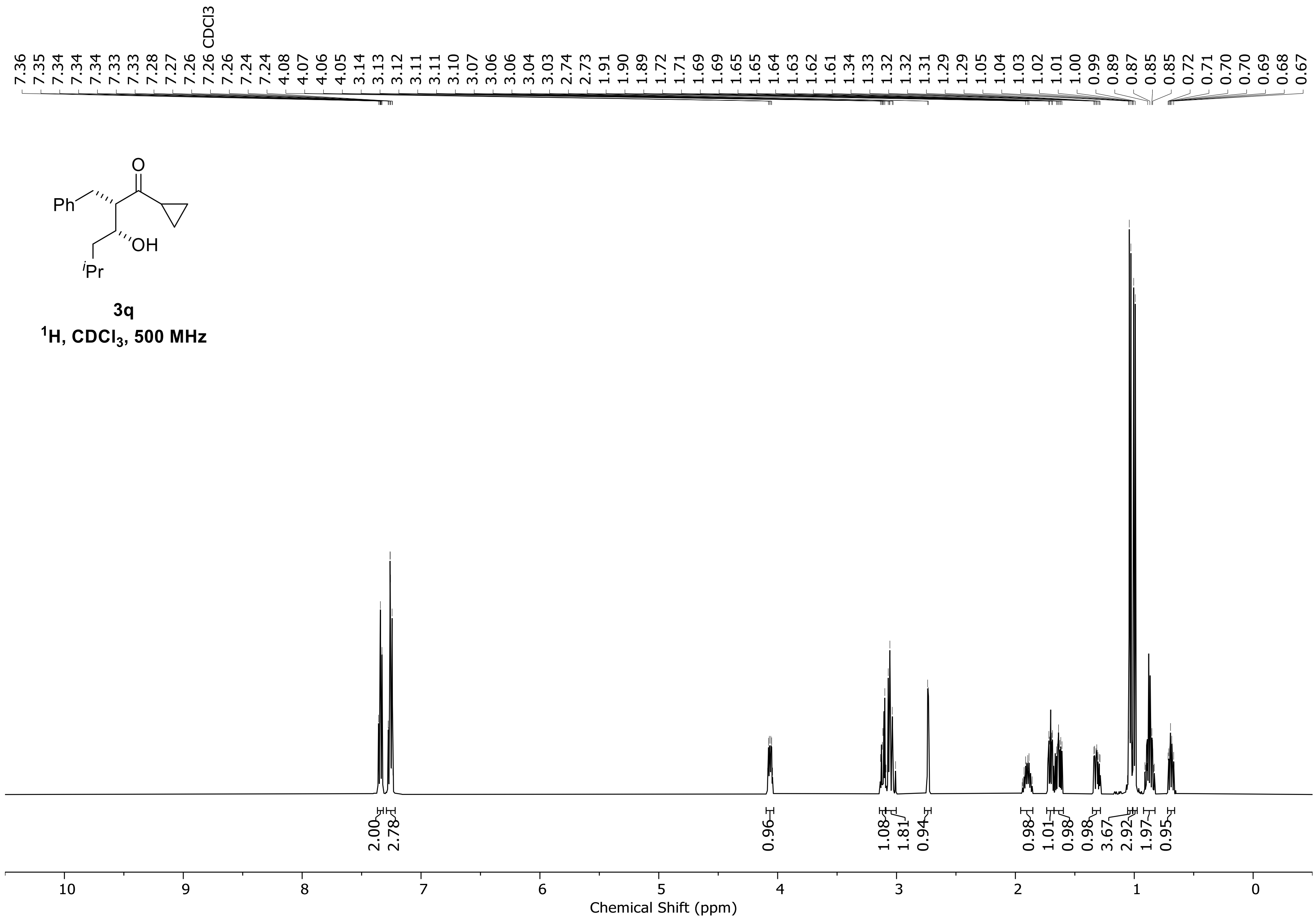

3q

^{1}H, CDCl$_3$, 500 MHz

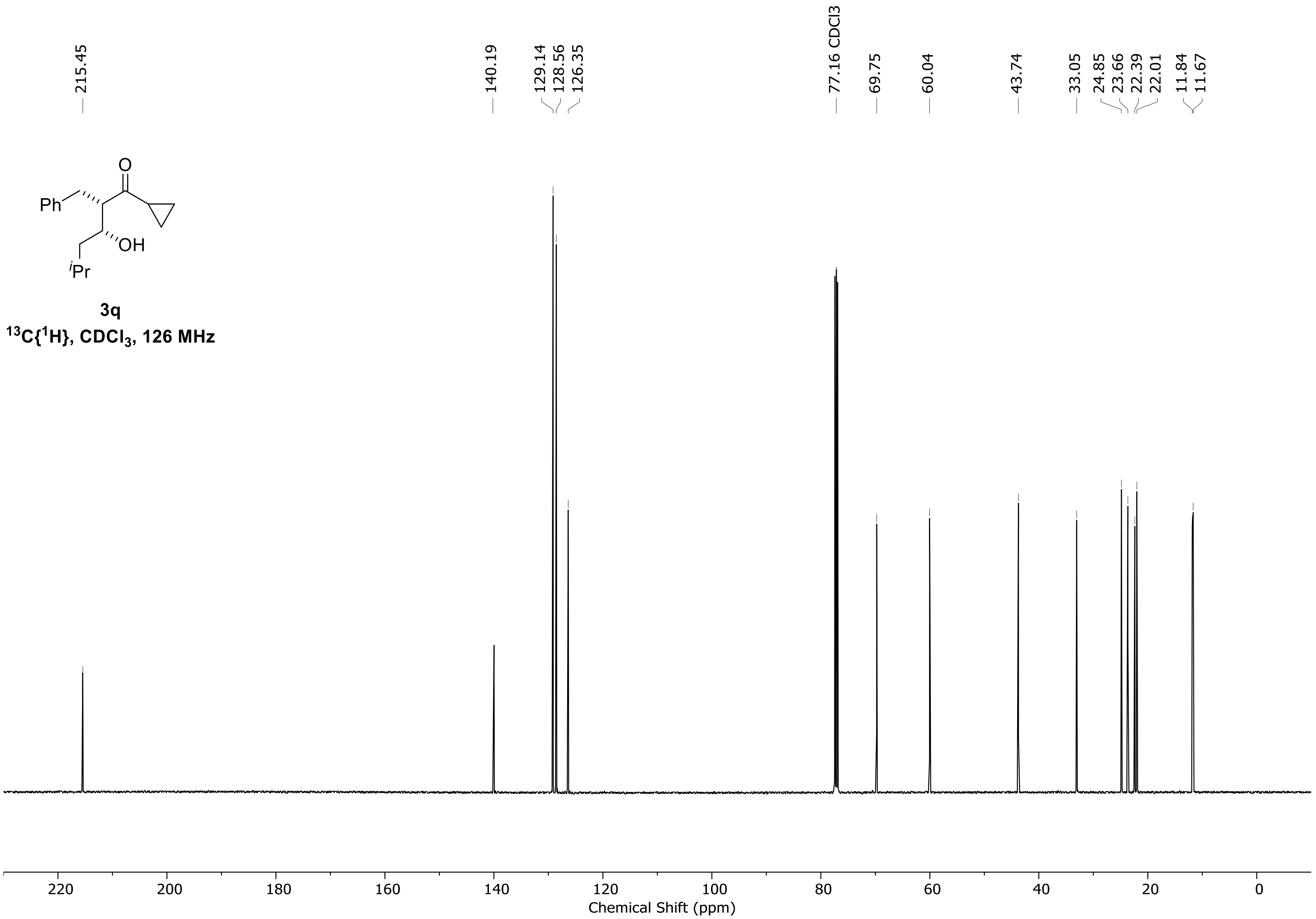

3q

$^{13}C\{^{1}H\}$, $CDCl_3$, 126 MHz

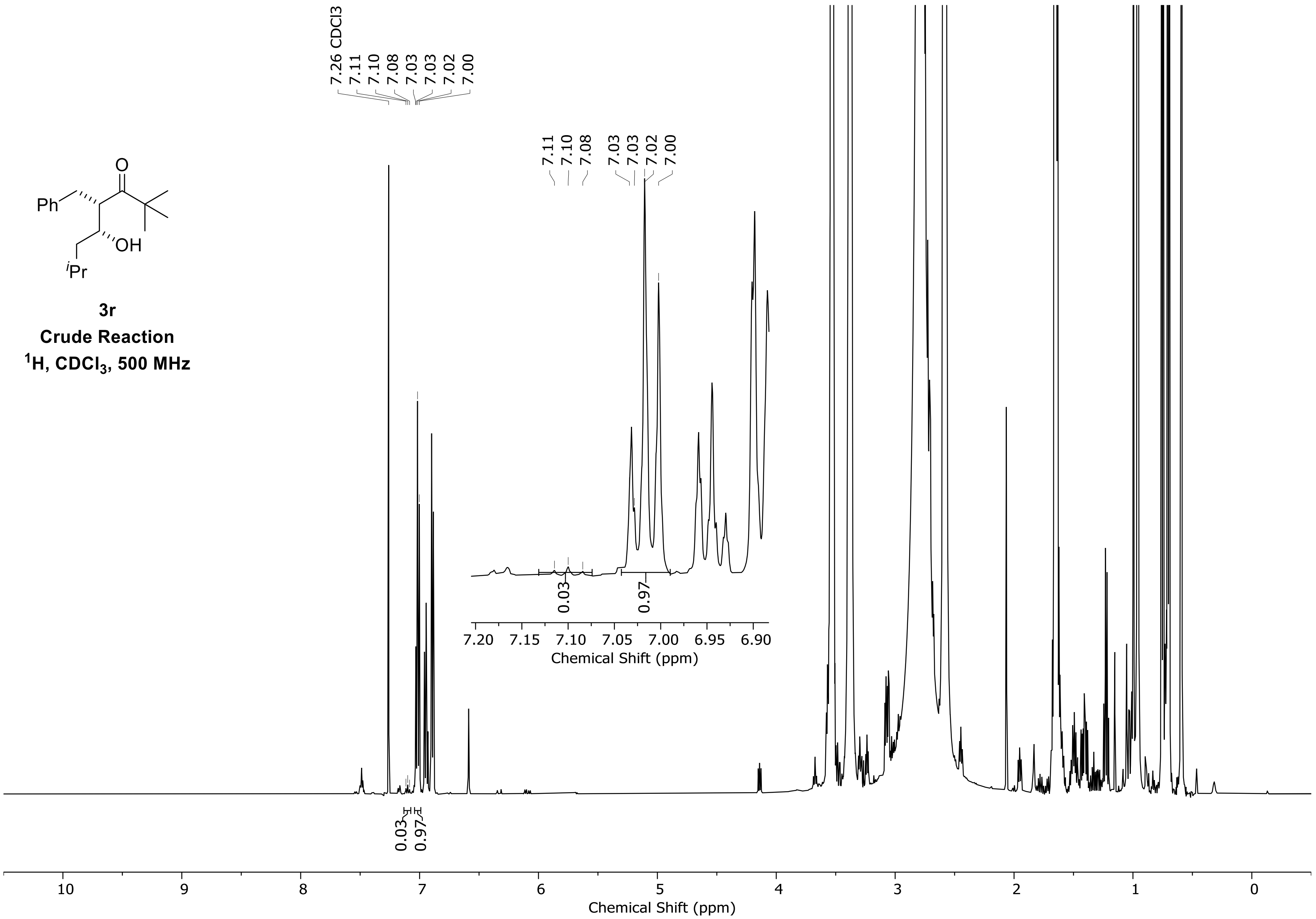

3r
Crude Reaction
1H, CDCl3, 500 MHz
7.26 CDCl3
7.11
7.10
7.08
7.03
7.03
7.02
7.00
0.03
0.97
Chemical Shift (ppm)

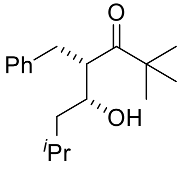

3r

^{1}H, CDCl$_3$, 500 MHz

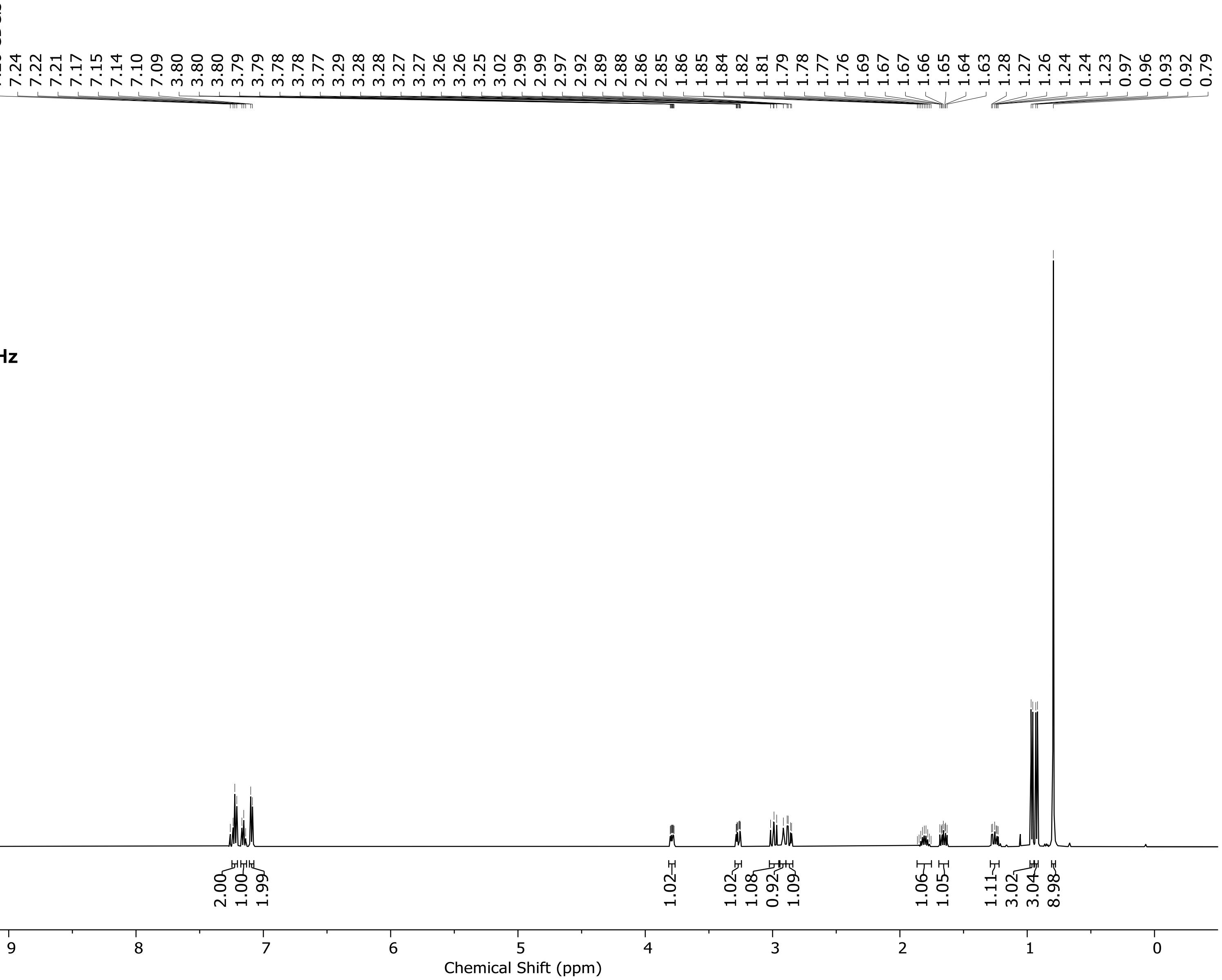

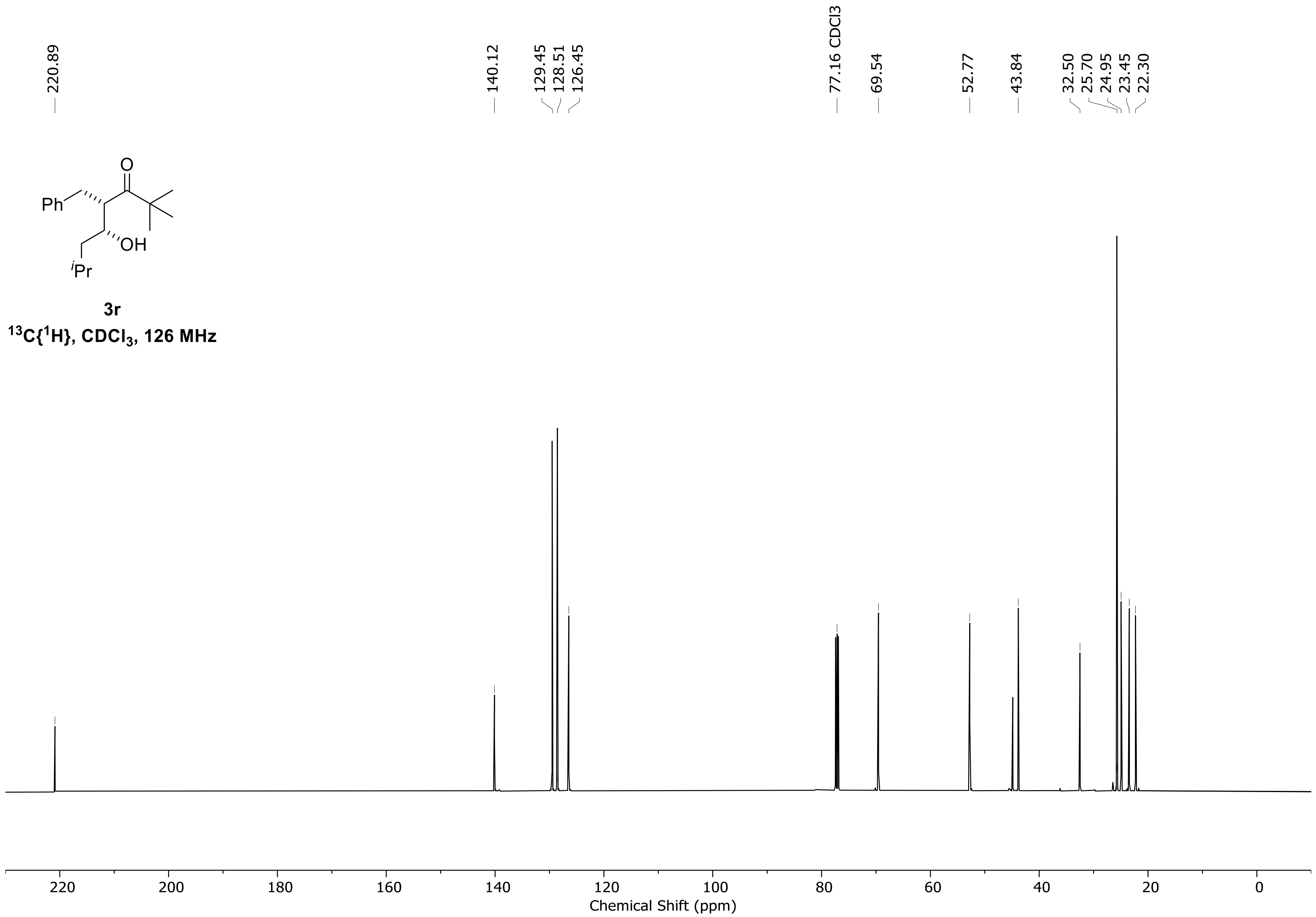
220.89
140.12
129.45
128.51
126.45
77.16 CDCl3
69.54
52.77
43.84
32.50
25.70
24.95
23.45
22.30
Ph
O
OH
^{i}Pr
3r
$^{13}C\{^1H\}$, $CDCl_3$, 126 MHz
220
200
180
160
140
120
100
80
60
40
20
0
Chemical Shift (ppm)

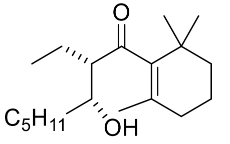

3s
Crude Reaction
^{1}H, $CDCl_3$, 600 MHz

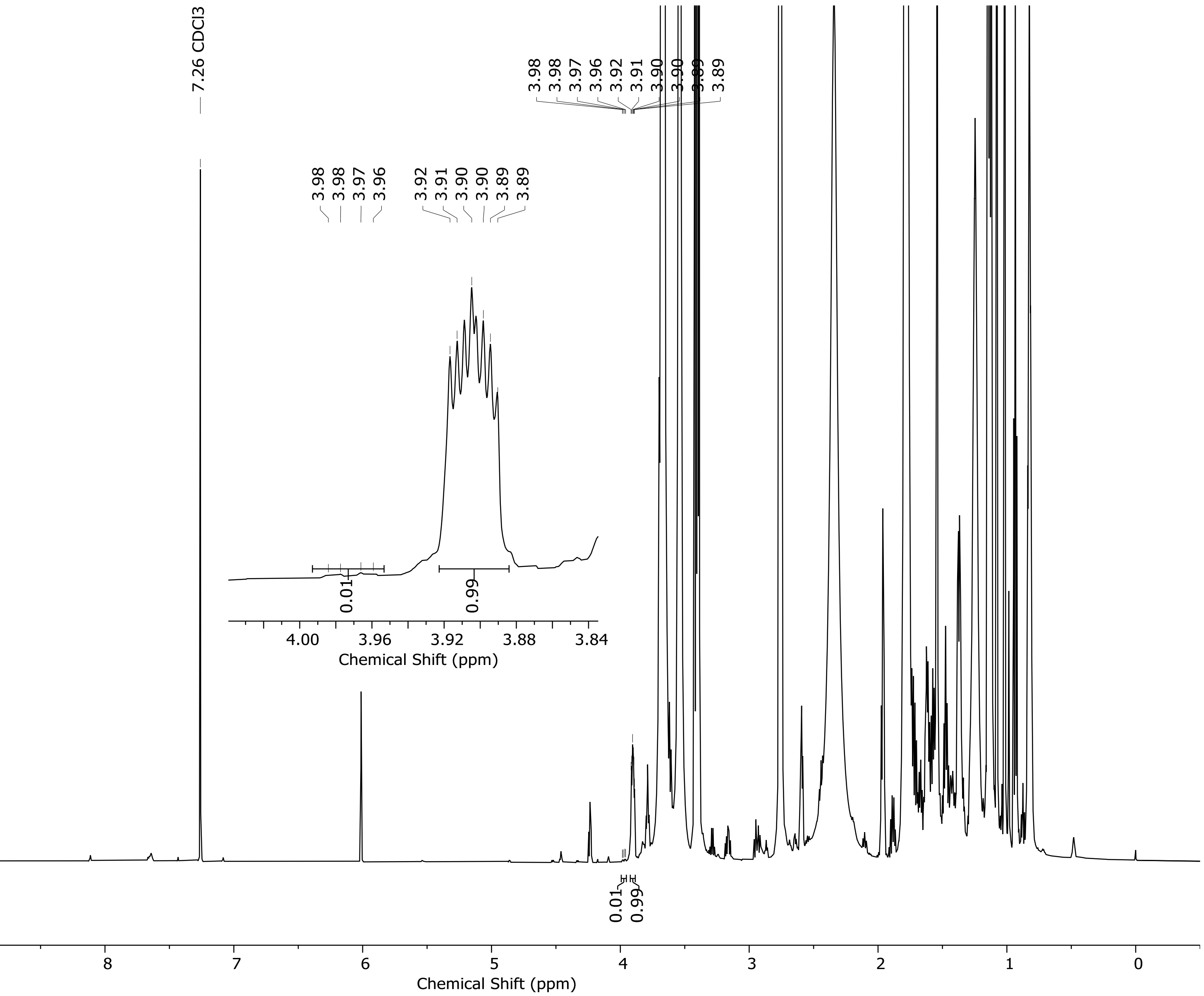

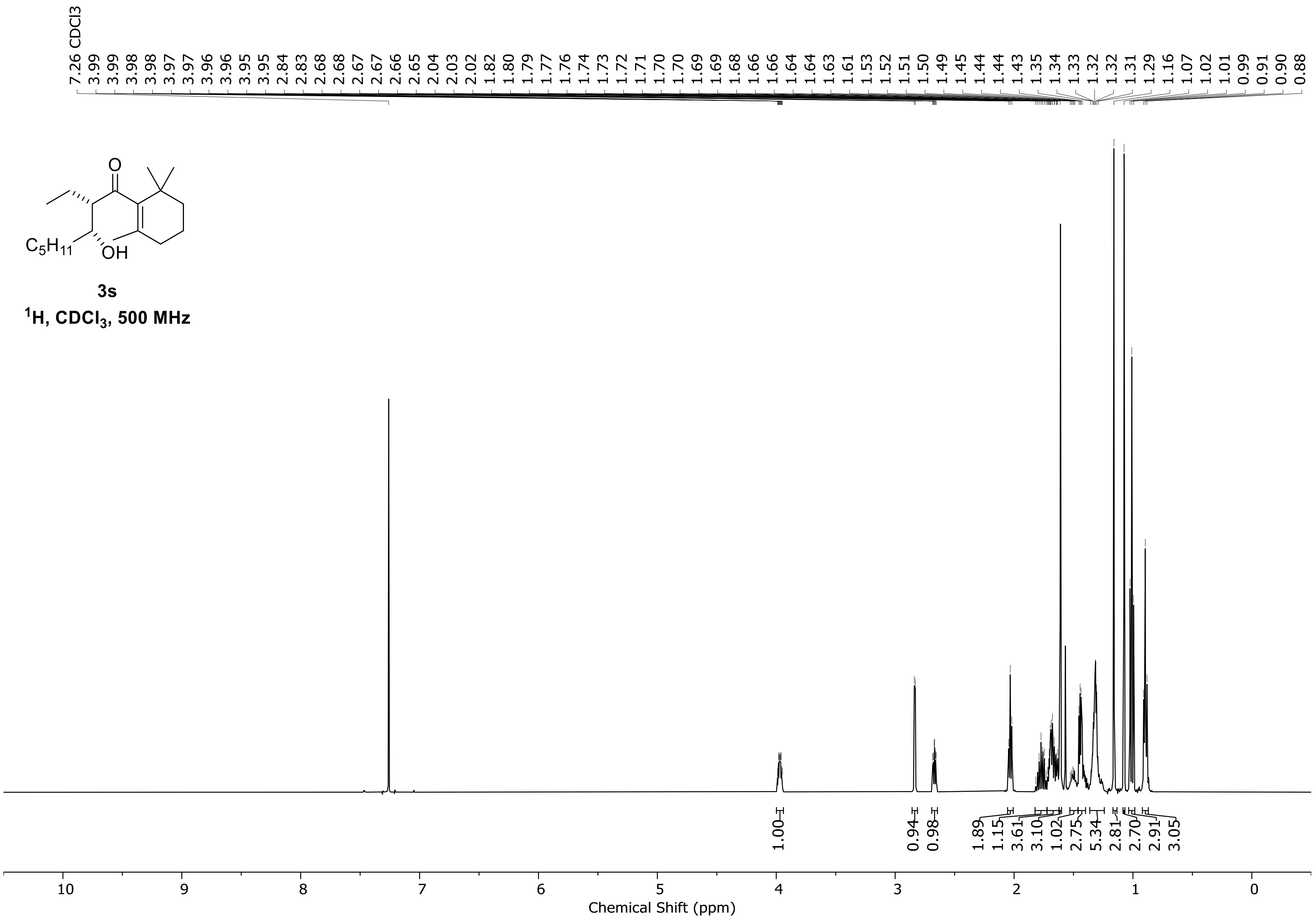

3s

^{1}H, $CDCl_3$, 500 MHz

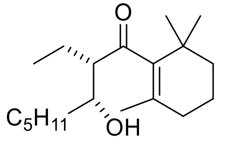

3s

$^{13}C\{^{1}H\}$, $CDCl_3$, 126 MHz

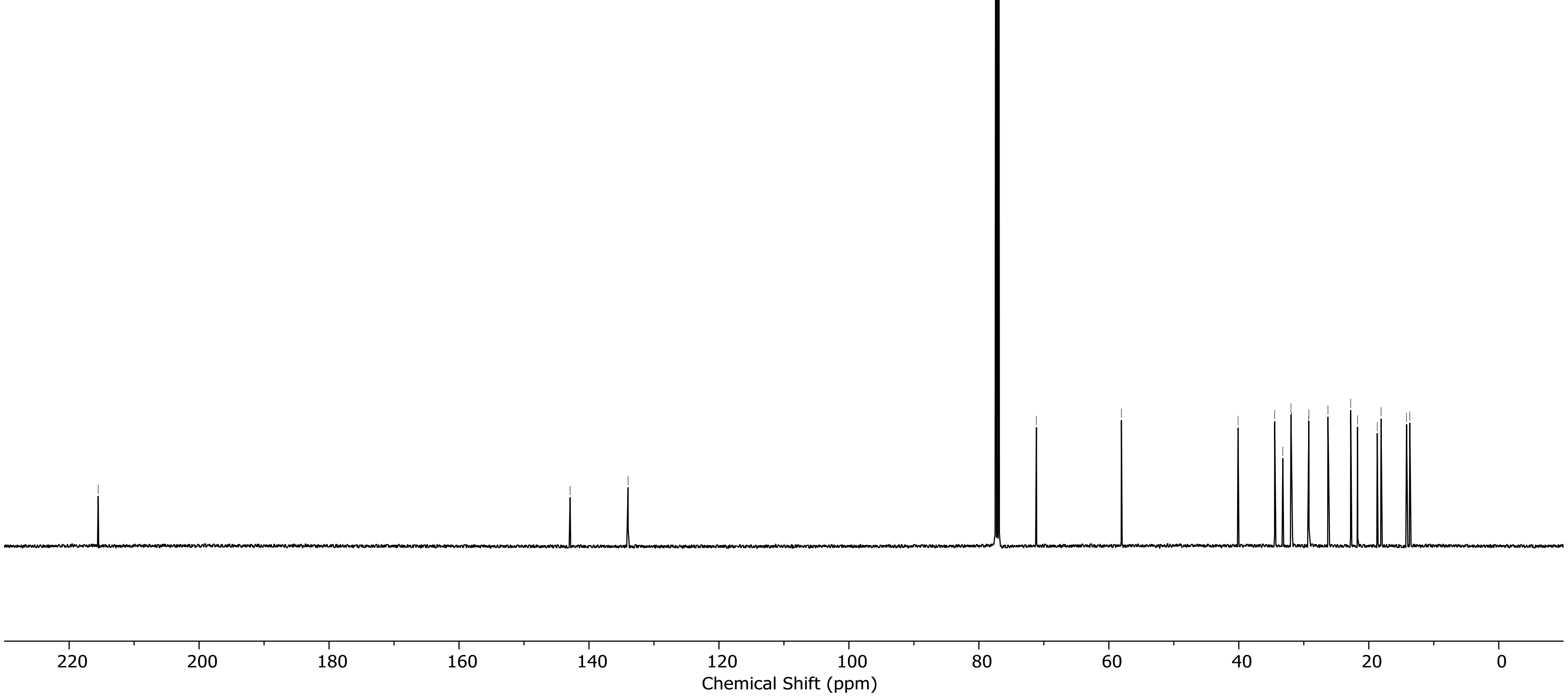

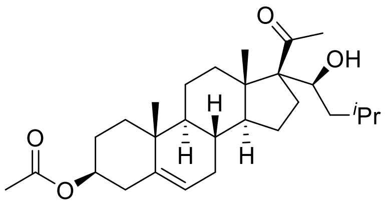

3t

^{1}H, CDCl$_3$, 500 MHz

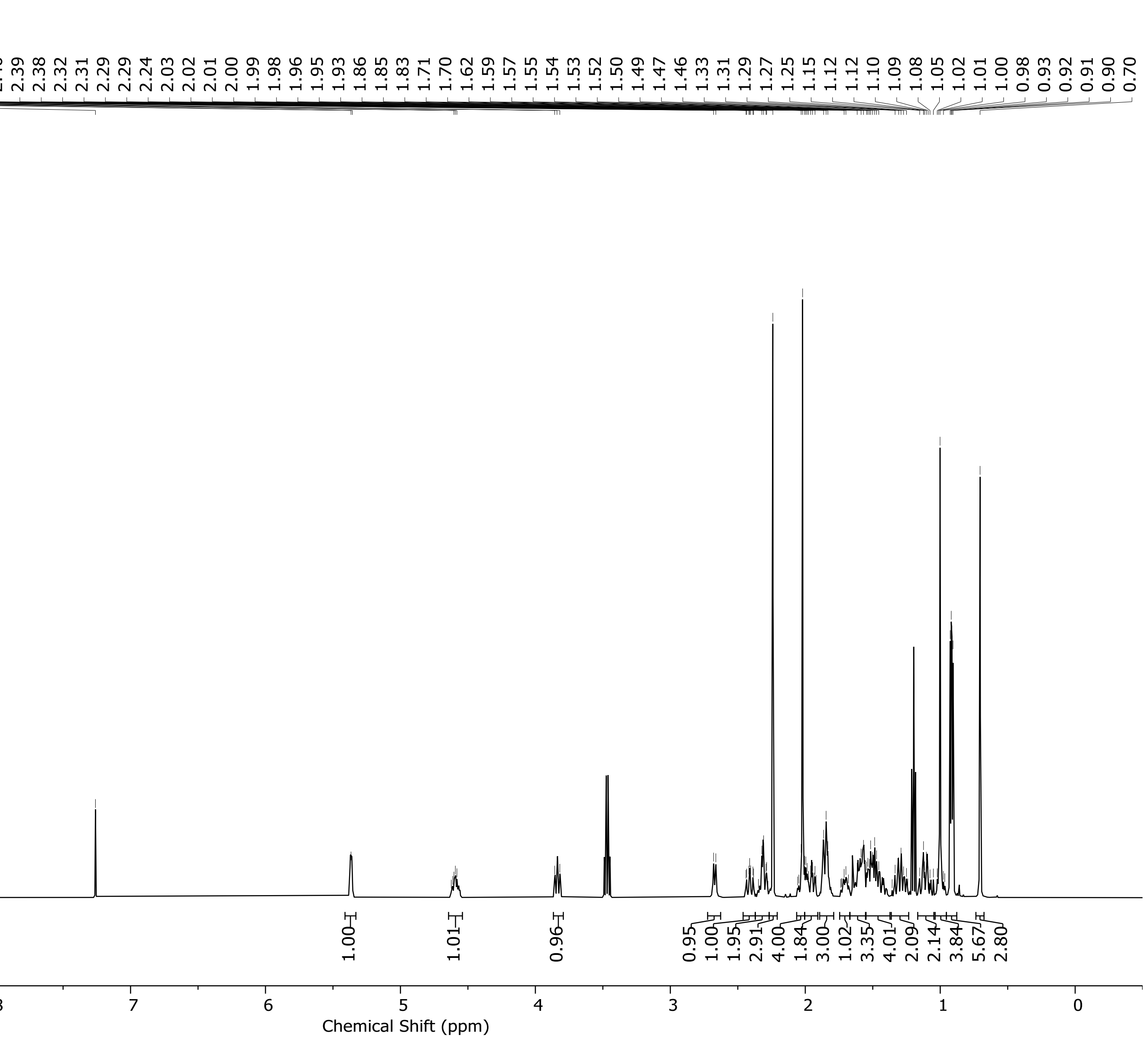

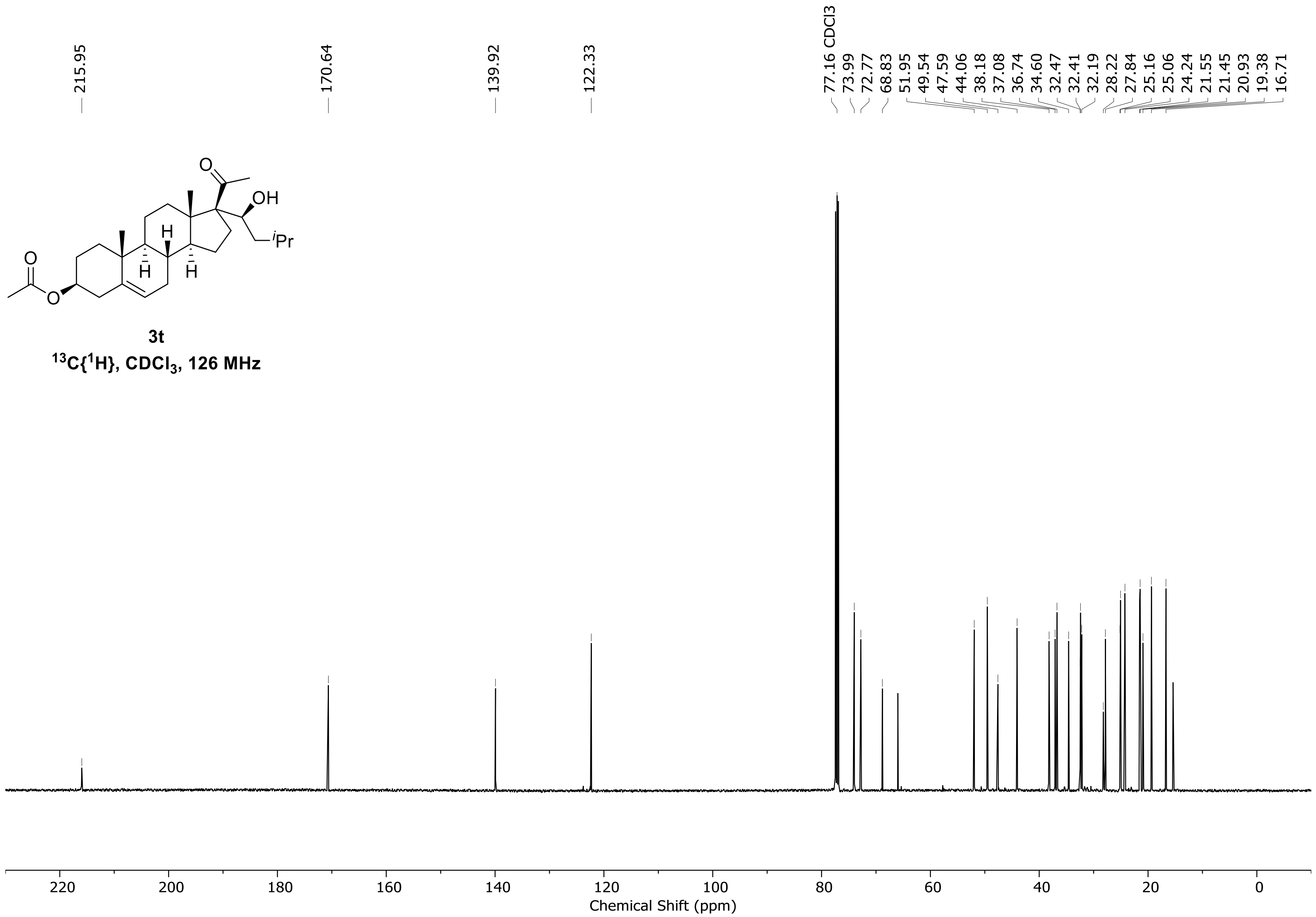

3t

$^{13}C\{^1H\}$, $CDCl_3$, 126 MHz

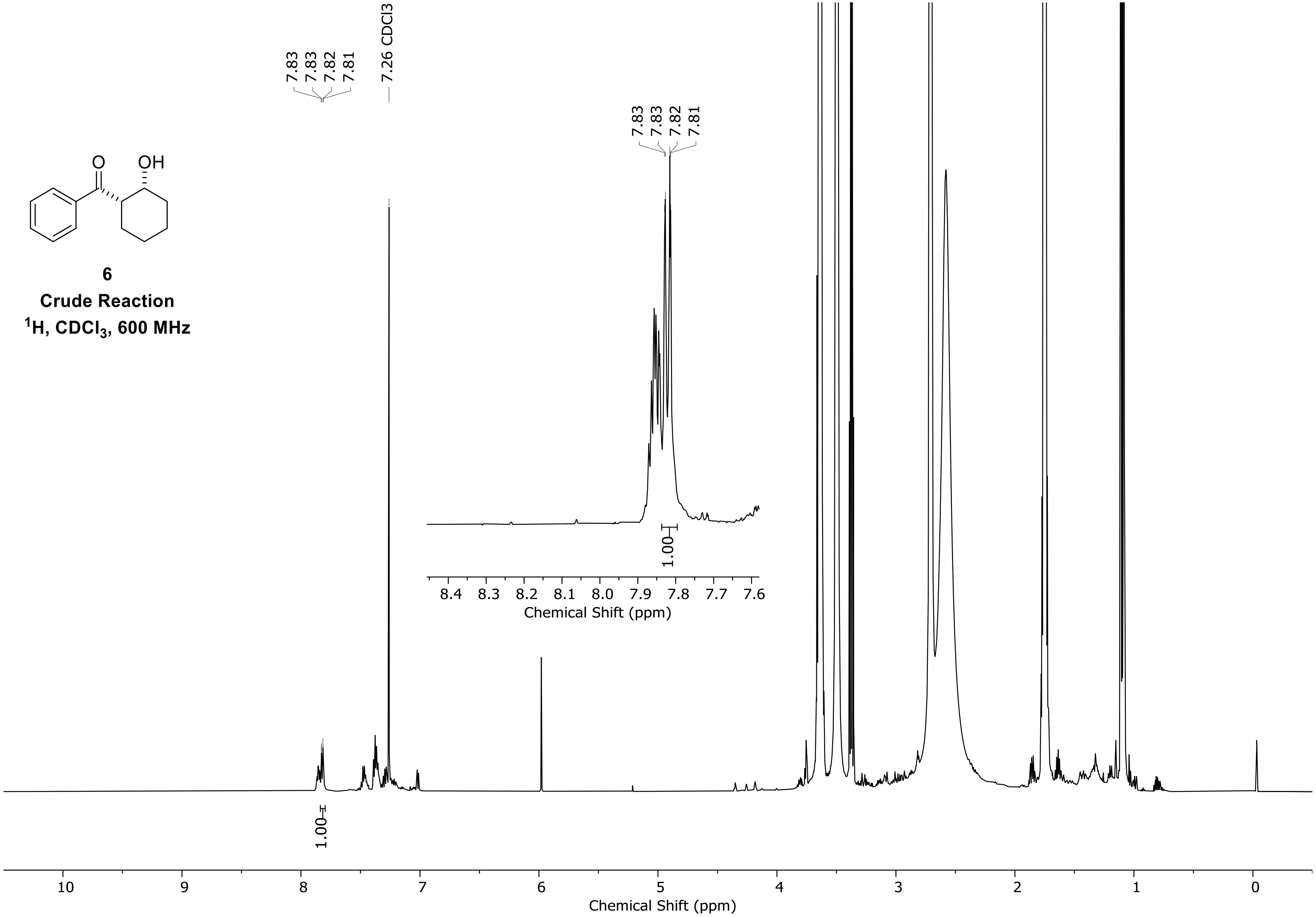

6
Crude Reaction
^{1}H, $CDCl_3$, 600 MHz

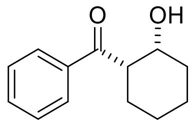

6
^{1}H, $CDCl_3$, 500 MHz

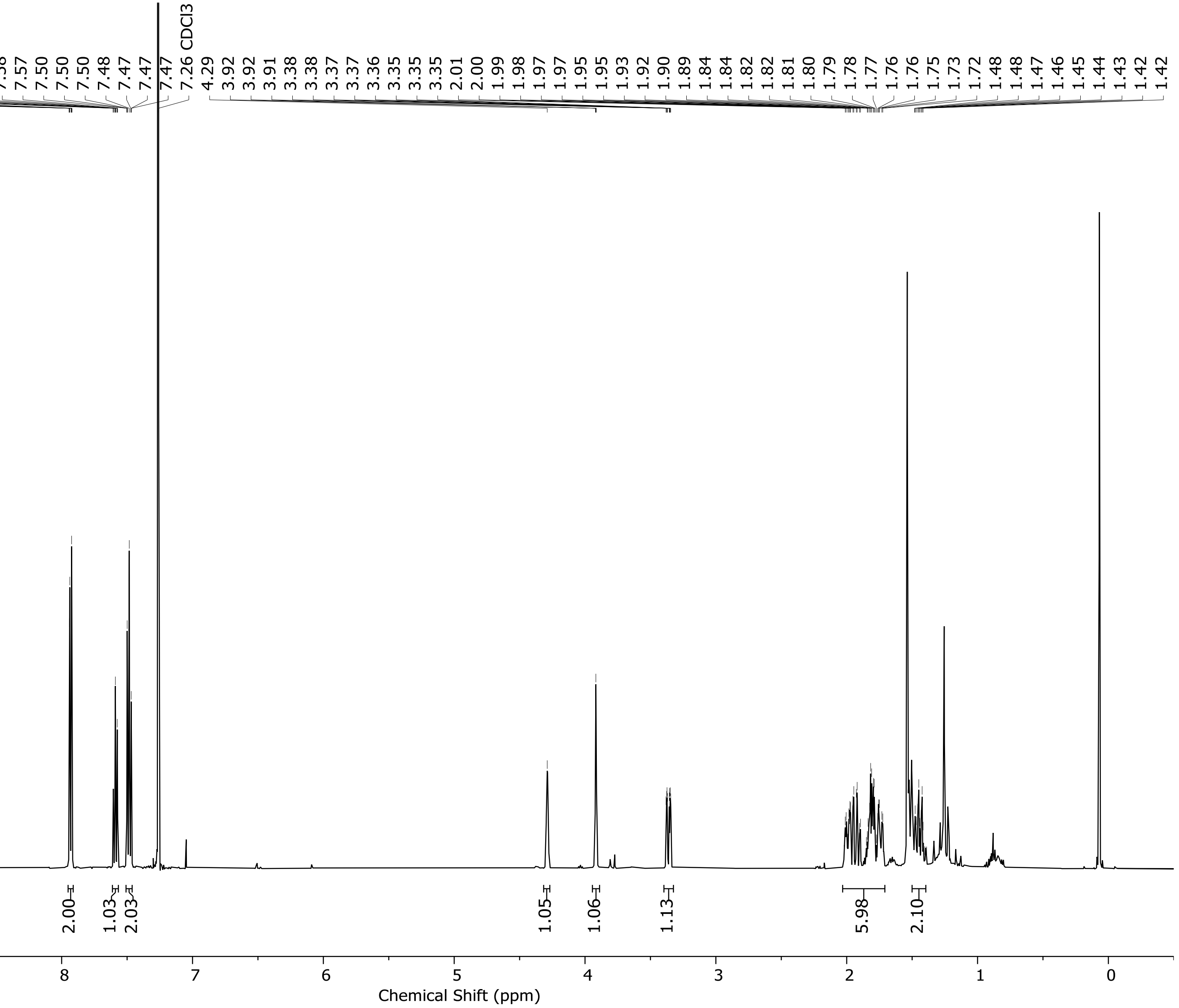

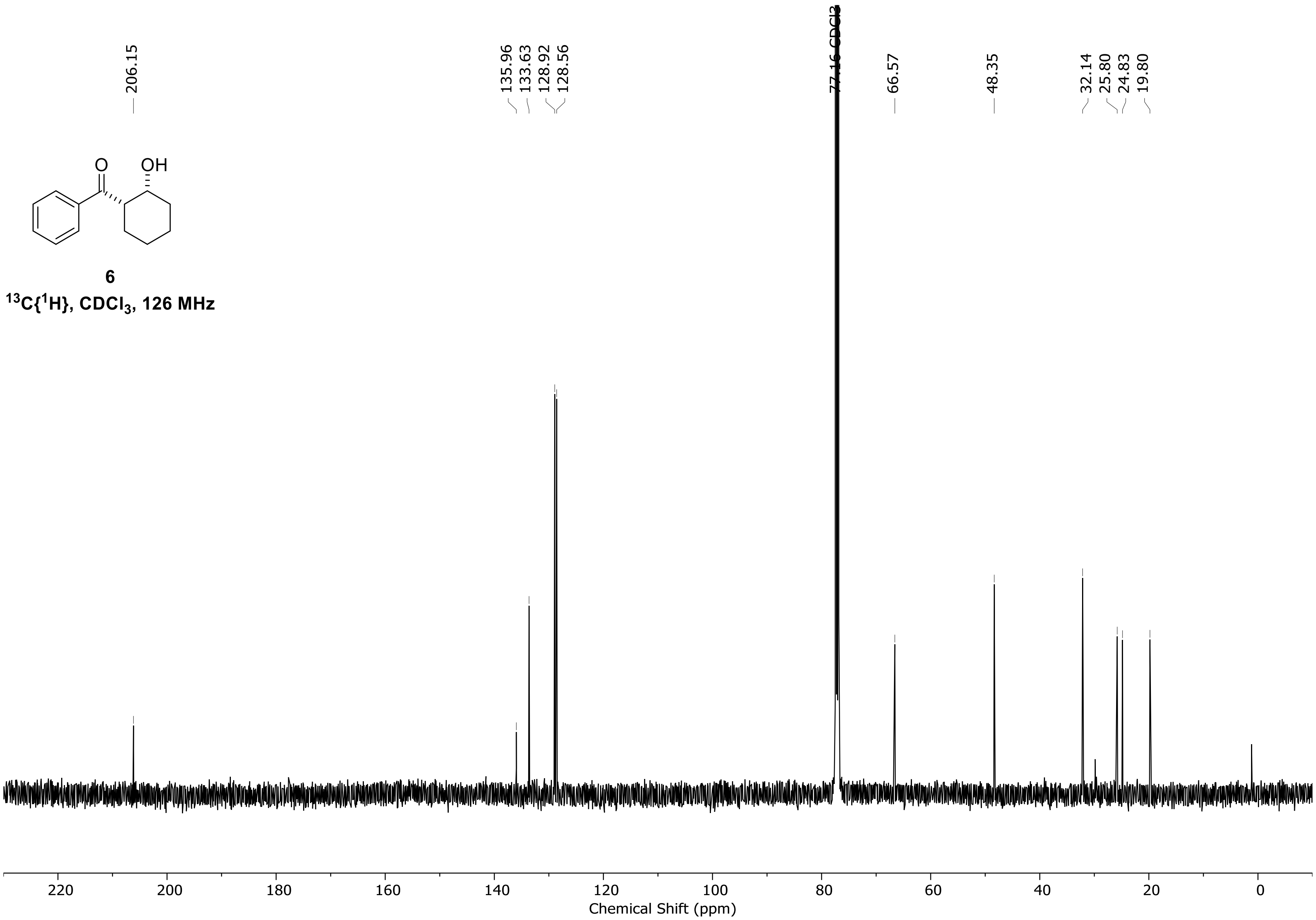
206.15
135.96
133.63
128.92
128.56
77.16 CDCl3
66.57
48.35
32.14
25.80
24.83
19.80
O
OH
6
$^{13}C\{^{1}H\}$, $CDCl_3$, 126 MHz
220 200 180 160 140 120 100 80 60 40 20 0
Chemical Shift (ppm)

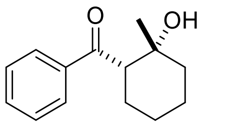

8

Crude Reaction

^{1}H, $CDCl_3$, 500 MHz

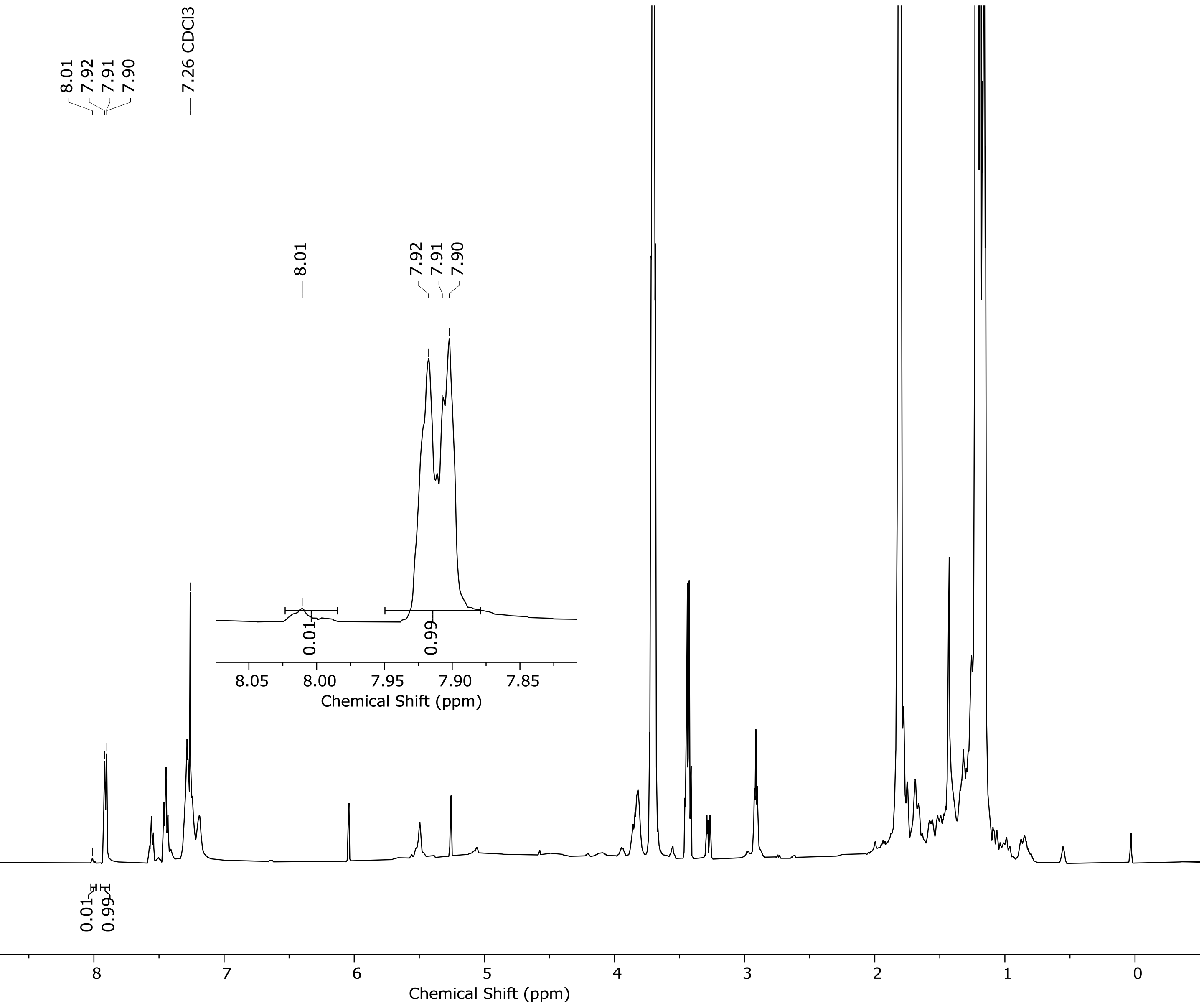

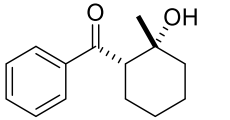

8

^{1}H, CDCl$_3$, 500 MHz

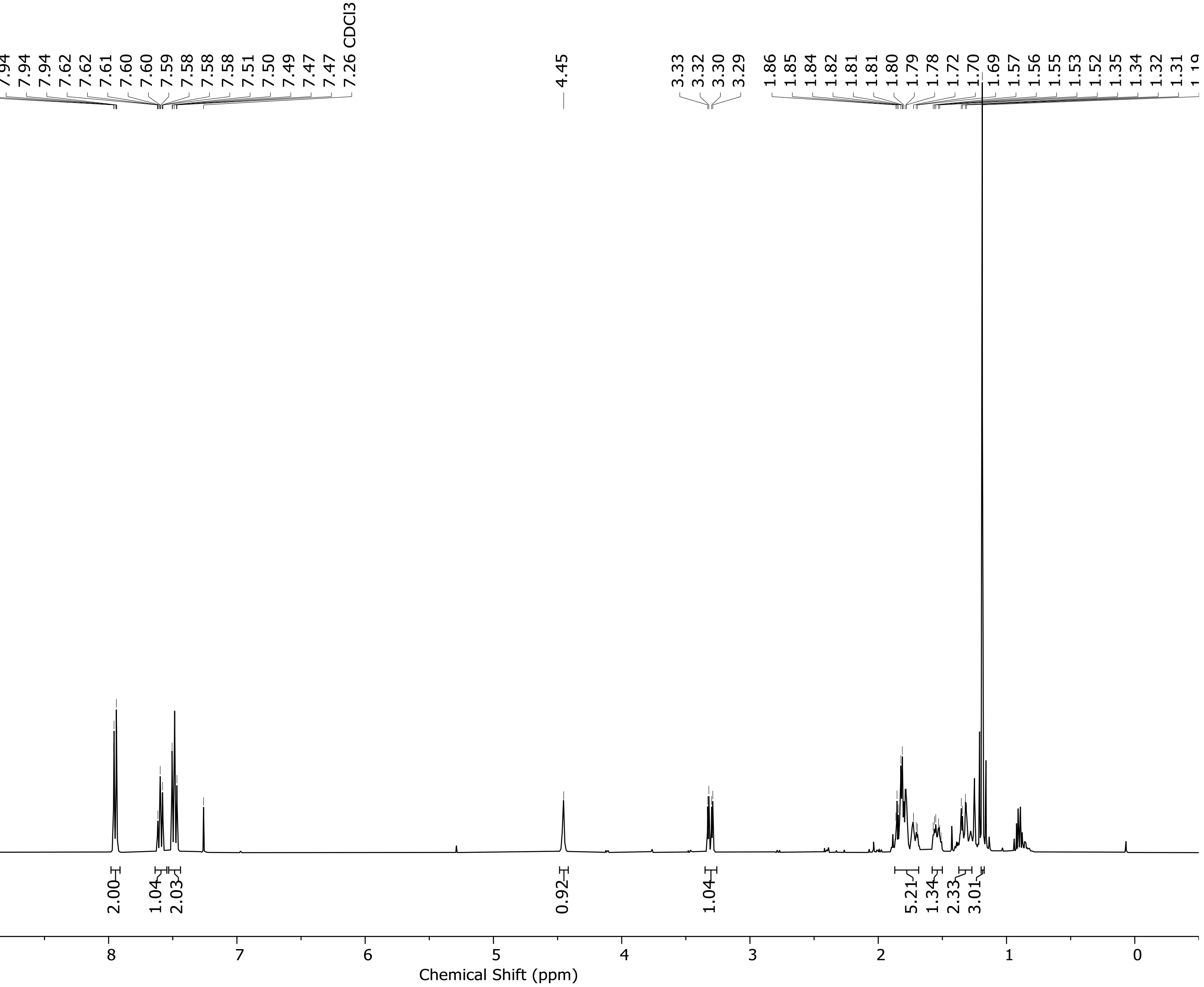

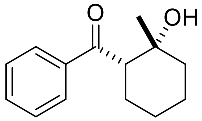

8

$^{13}C\{^{1}H\}$, $CDCl_3$, 126 MHz

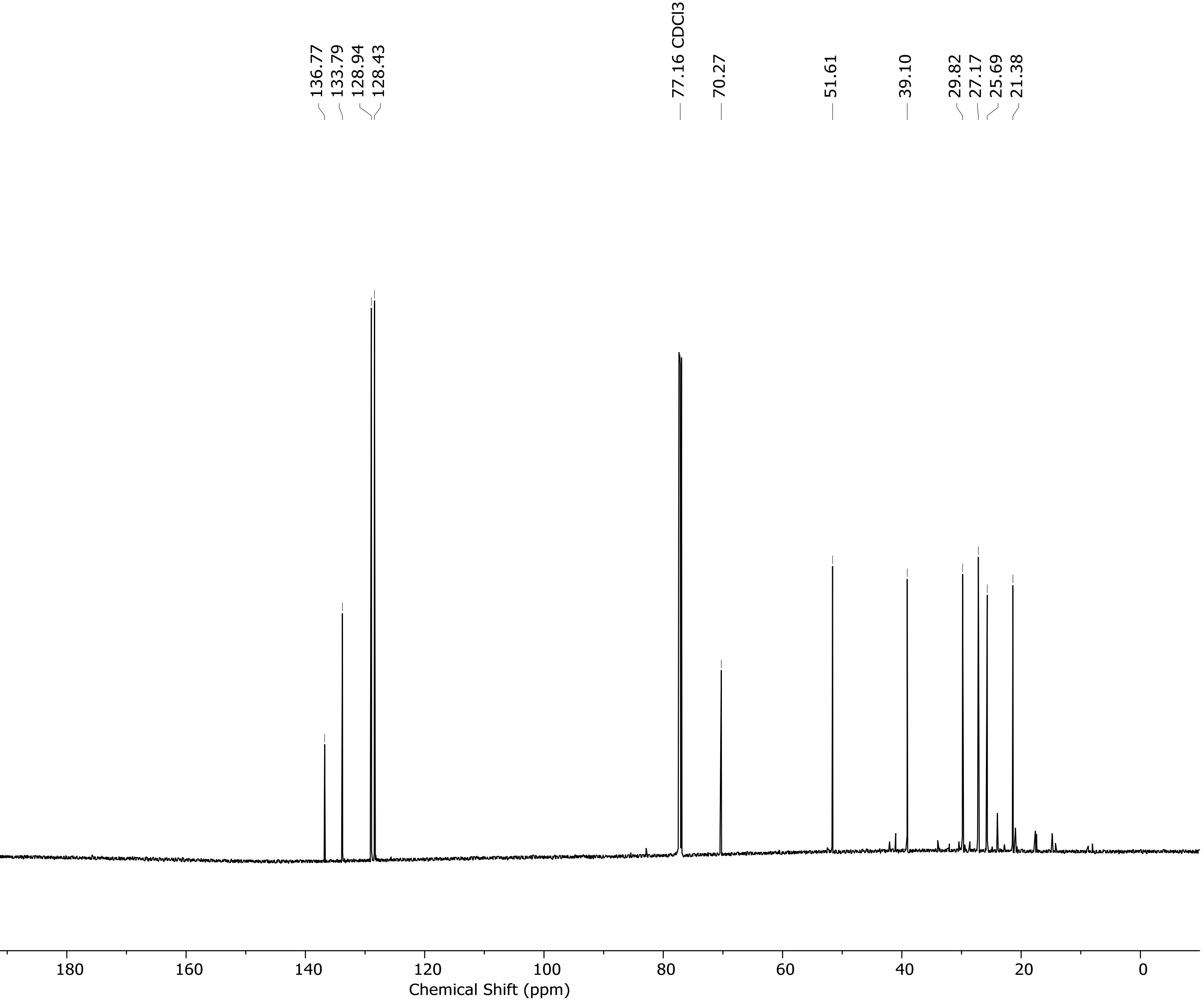

10
^{1}H, CDCl$_3$, 500 MHz

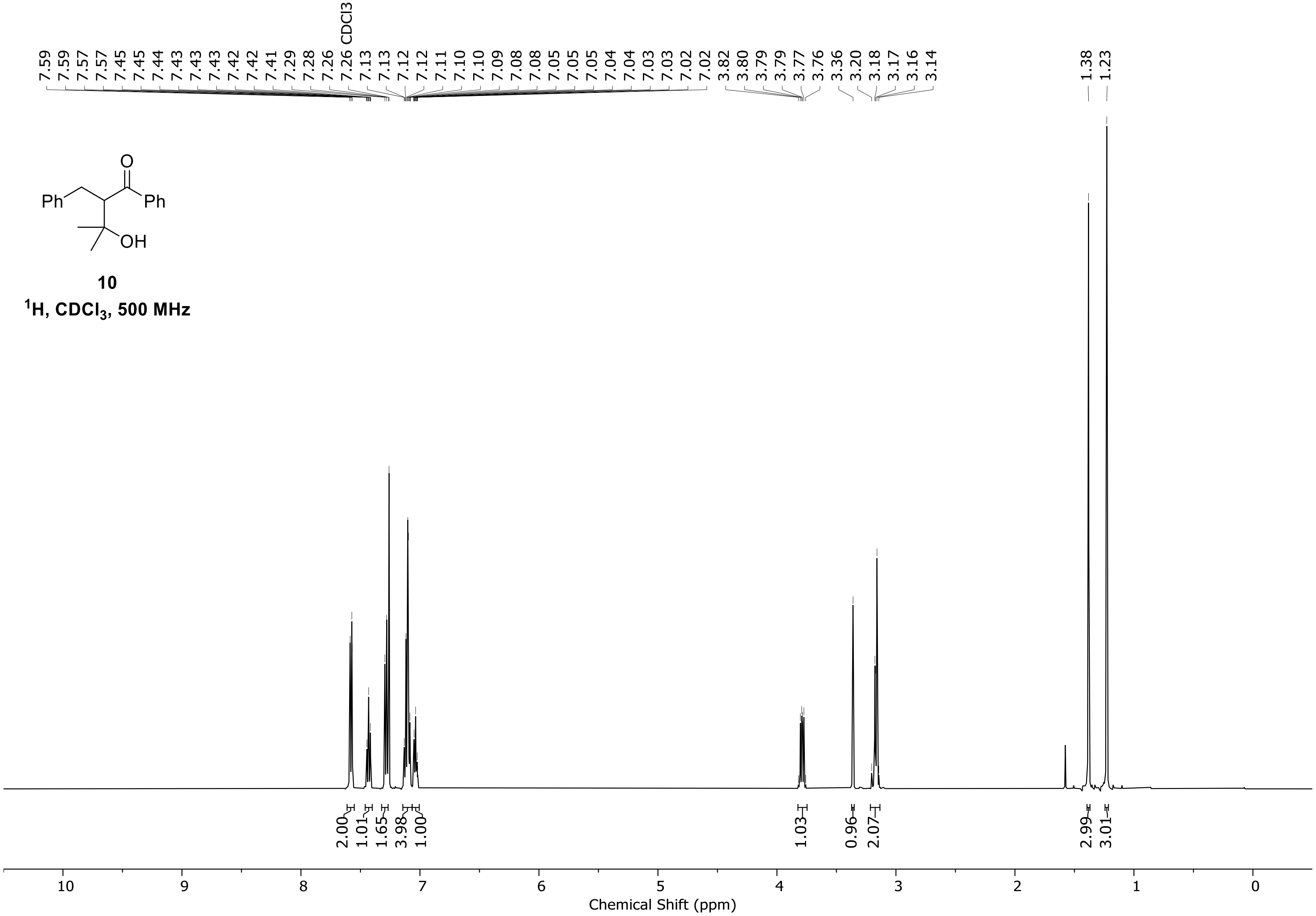

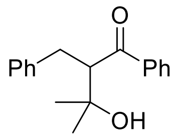

10
$^{13}C\{^{1}H\}$, $CDCl_3$, 126 MHz

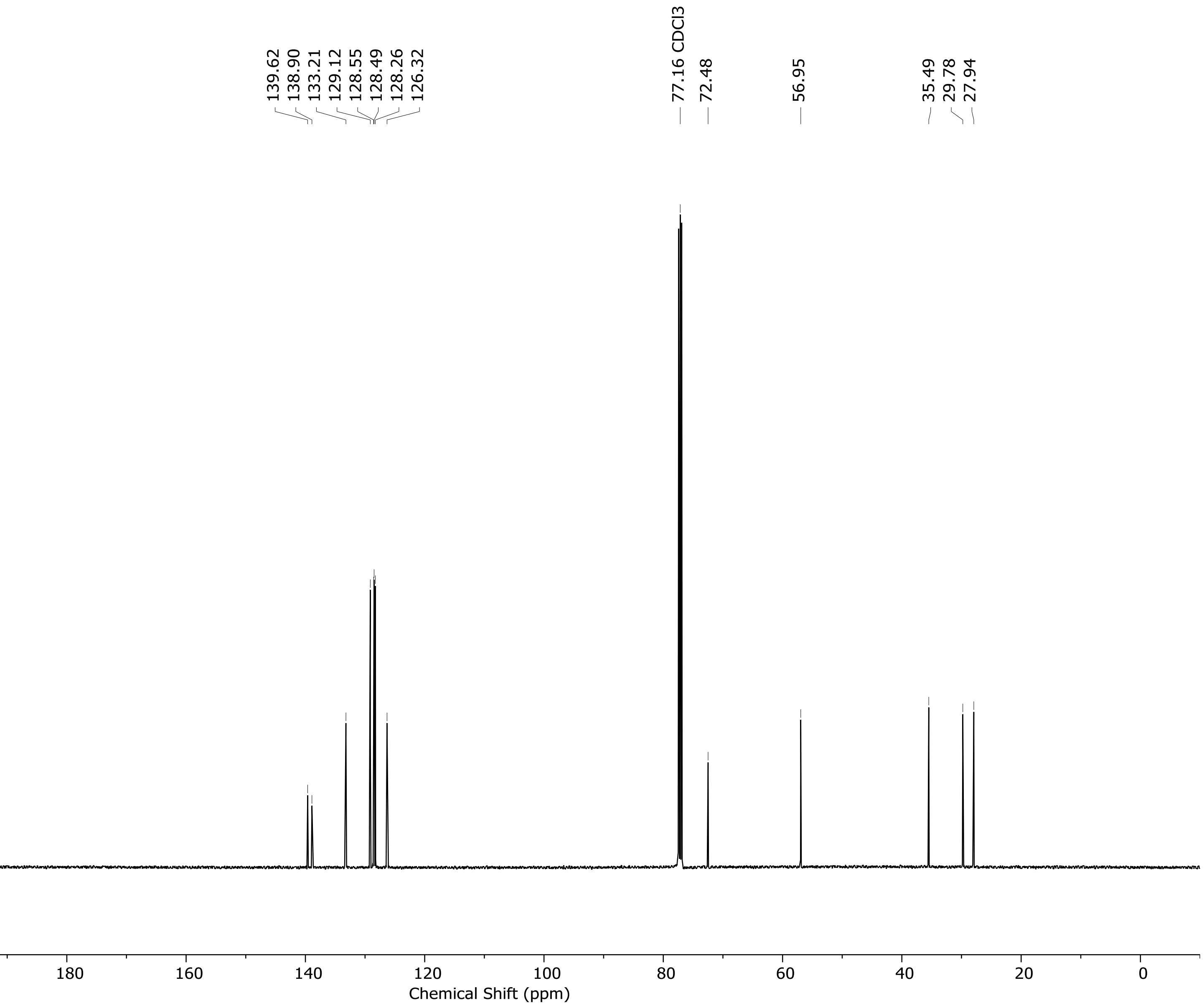

Appendix II: HPLC Traces of Novel Compounds

(2*S*,3*R*)-2-Benzyl-3-hydroxy-5-methyl-1-phenylhexan-1-one (3a)

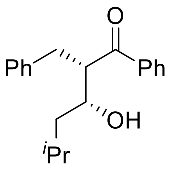

(2*S*,3*R*)-3a

CHIRALPAK IC, 95:5 *n*-hexane:isopropanol, flow rate 1.0 mL/min, 244 nm, 30 °C. t_R **(2*R*,3*S*)-3a** 7.9 min, t_R **(2*S*,3*R*)-3a** 8.9 min, 90:10 *e.r.*

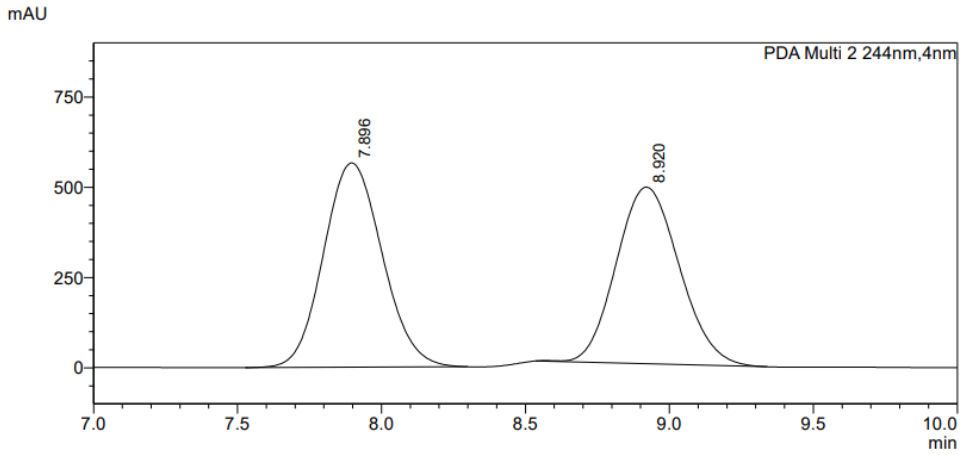

PDA Ch2 244nm

Peak#	Ret. Time	Area	Area%
1	7.896	8041998	51.825
2	8.920	7475732	48.175
Total		15517730	100.000

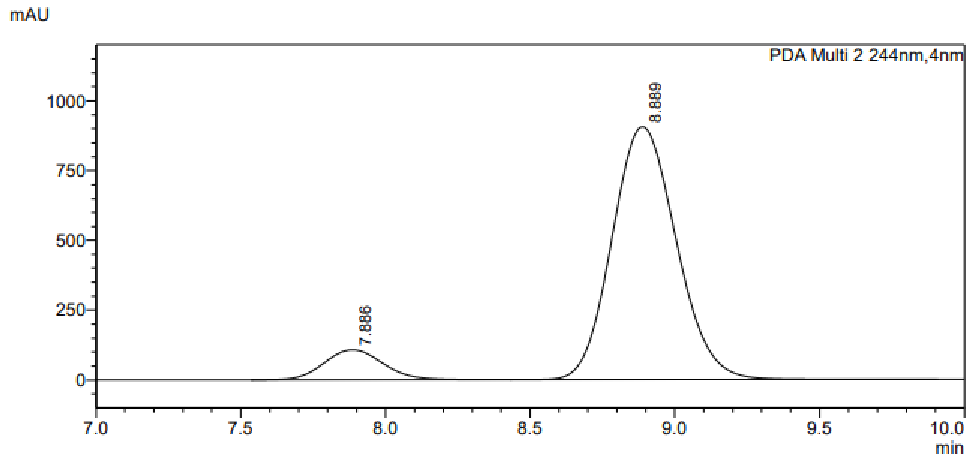

PDA Ch2 244nm

Peak#	Ret. Time	Area	Area%
1	7.886	1503823	9.713
2	8.889	13979178	90.287
Total		15483001	100.000

CHIRALPAK IB, 95:5 *n*-hexane:isopropanol, flow rate 1.0 mL/min, 244 nm, 30 °C. t_R **(2*S*,3*R*)-3a** 6.7 min, t_R **(2*R*,3*S*)-3a** 9.3 min. t_R of single crystal **(2*S*,3*R*)-3a** 6.7 min

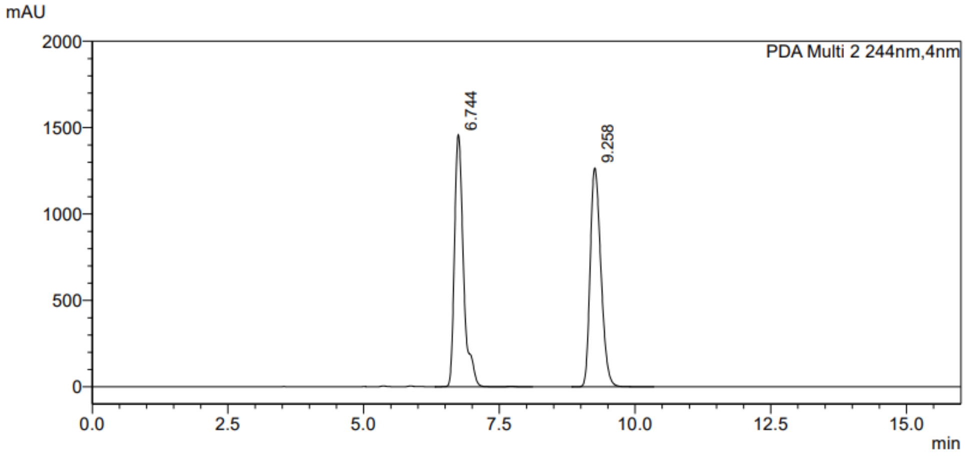

PDA Ch2 244nm

Peak#	Ret. Time	Area	Area%
1	6.744	16949549	49.326
2	9.258	17412949	50.674
Total		34362498	100.000

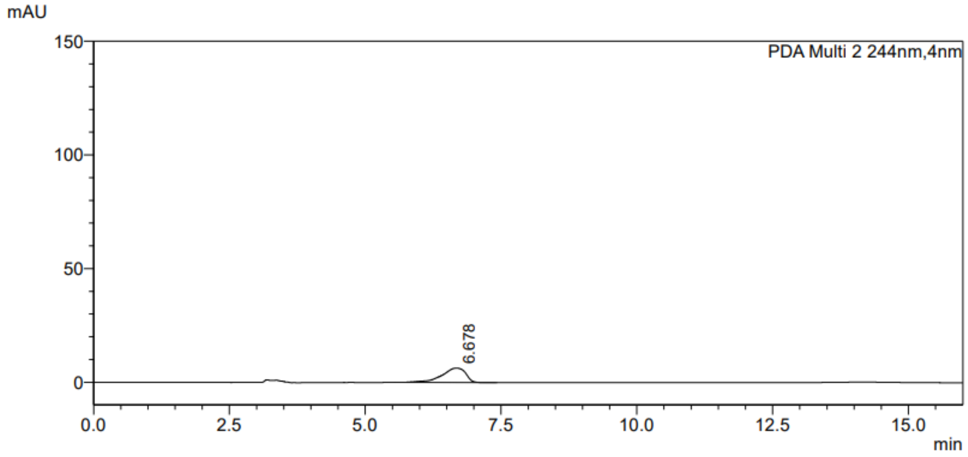

PDA Ch2 244nm

Peak#	Ret. Time	Area	Area%
1	6.678	194367	100.000
Total		194367	100.000

(2*S*,3*R*)-2-Benzyl-3-cyclohexyl-3-hydroxy-1-phenylpropan-1-one (3e)

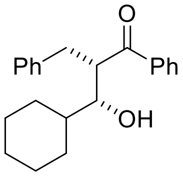

(2*S*,3*R*)-3e

CHIRALPAK IC, 95:5 *n*-hexane:isopropanol, flow rate 1.0 mL/min, 244 nm, 30 °C. t_R **(2*R*,3*S*)-3e** 10.8 min, t_R **(2*S*,3*R*)**-3e 12.7 min, 85:15 *e.r.*

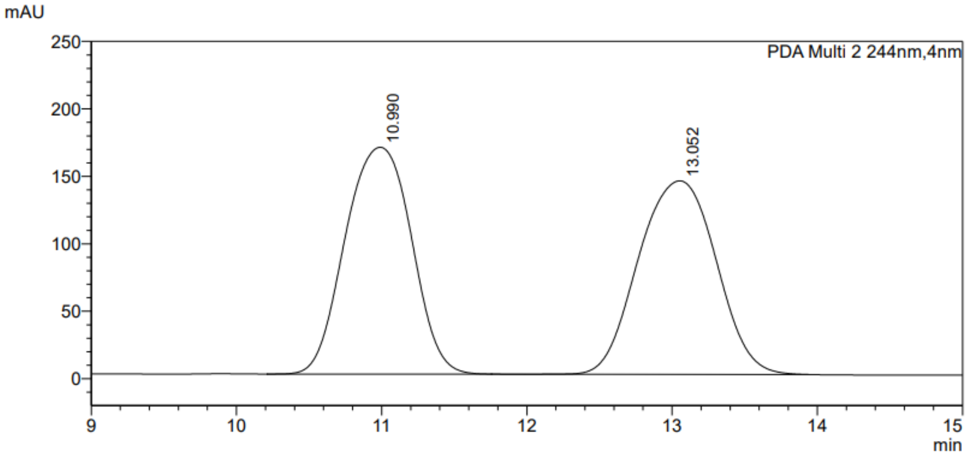

PDA Ch2 244nm

Peak#	Ret. Time	Area	Area%
1	10.990	5350815	50.013
2	13.052	5347987	49.987
Total		10698802	100.000

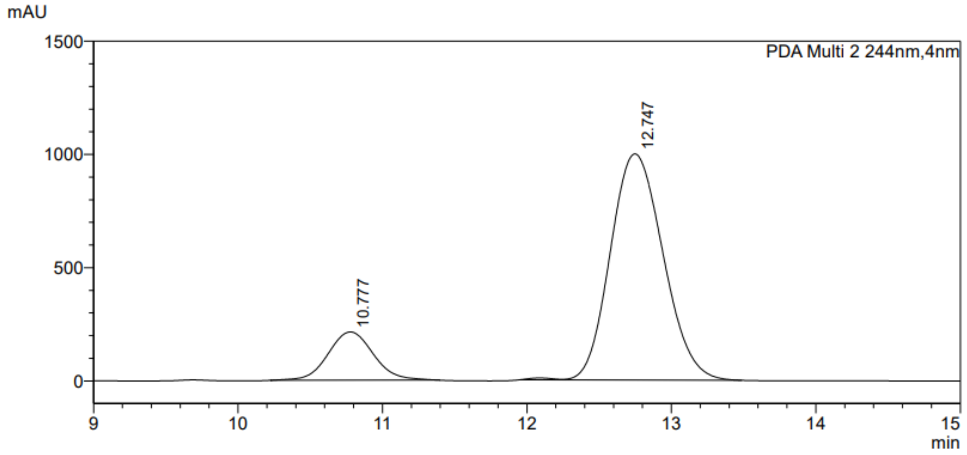

PDA Ch2 244nm

Peak#	Ret. Time	Area	Area%
1	10.777	4662294	15.486
2	12.747	25444704	84.514
Total		30106998	100.000

(2*S*,3*S*)-2-Benzyl-3-hydroxy-4,4-dimethyl-1-phenylpentan-1-one (3f)

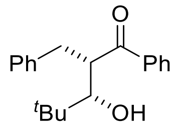

(2*S*,3*S*)-3f

CHIRALPAK IC, 95:5 *n*-hexane:isopropanol, flow rate 1.0 mL/min, 244 nm, 30 °C. t_R **(2*R*,3*R*)-3f** 6.7 min, t_R **(2*S*,3*S*)-3f** 8.6 min, 86:14 *e.r.*

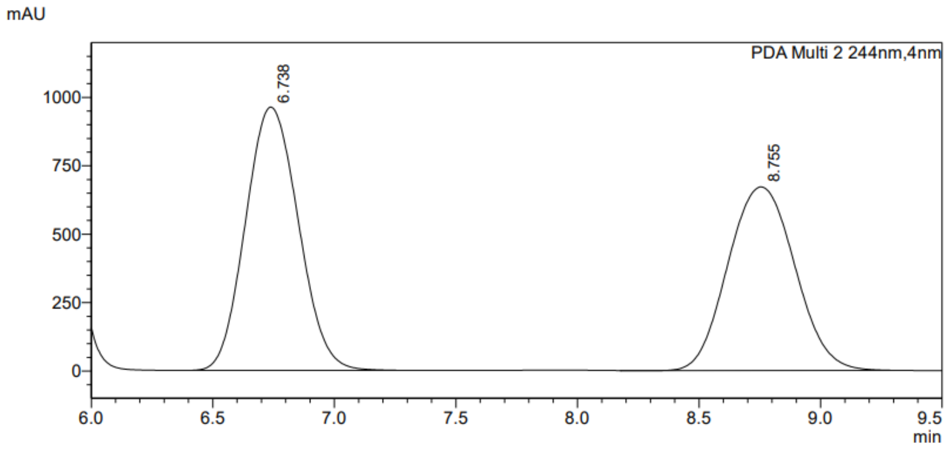

PDA Ch2 244nm

Peak#	Ret. Time	Area	Area%
1	6.738	14579647	53.110
2	8.755	12871930	46.890
Total		27451576	100.000

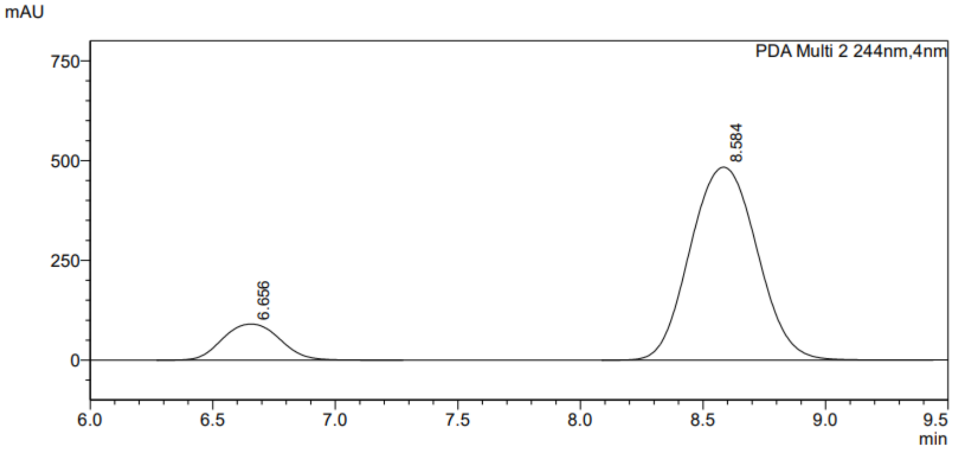

PDA Ch2 244nm

Peak#	Ret. Time	Area	Area%
1	6.656	1459899	13.724
2	8.584	9177537	86.276
Total		10637435	100.000

(2*S*,3*R*)-2-Benzyl-1-(4-fluorophenyl)-3-hydroxy-5-methylhexan-1-one (3i)

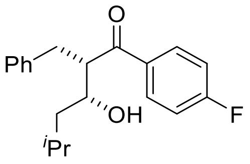

(2*S*,3*R*)-3i

CHIRALPAK IB, 95:5 *n*-hexane:isopropanol, flow rate 1.0 mL/min, 244 nm, 30 °C. t_R **(2*S*,3*R*)-3i** 6.9 min, t_R **(2*R*,3*S*)-3i** 10.7 min, 85:15 *e.r.*

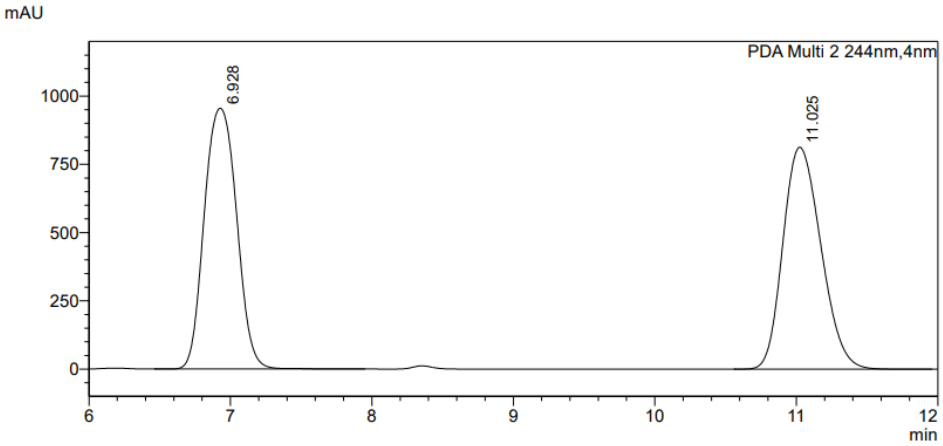

PDA Ch2 244nm

Peak#	Ret. Time	Area	Area%
1	6.928	15225278	50.053
2	11.025	15192749	49.947
Total		30418026	100.000

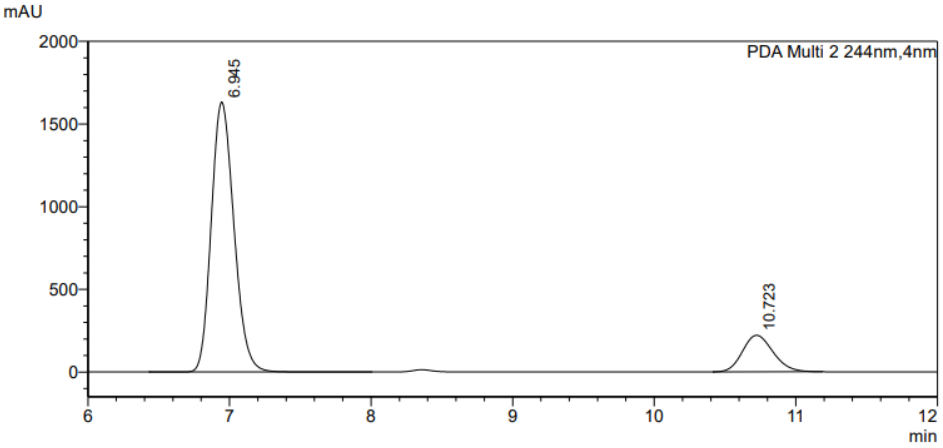

PDA Ch2 244nm

Peak#	Ret. Time	Area	Area%
1	6.945	18158699	84.525
2	10.723	3324595	15.475
Total		21483295	100.000

(2*S*,3*R*)-2-(2,4-Dimethoxy-6-methylbenzyl)-3-hydroxy-5-methyl-1-phenylhexan-1-one (3l)

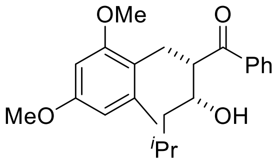

(2*S*,3*R*)-3l

CHIRALPAK IC, 95:5 *n*-hexane:isopropanol, flow rate 1.0 mL/min, 244 nm, 30 °C. t_R **(2*R*,3*S*)-3l** 12.0 min, t_R **(2*S*,3*R*)-3l** 19.3 min, 80:20 *e.r.*

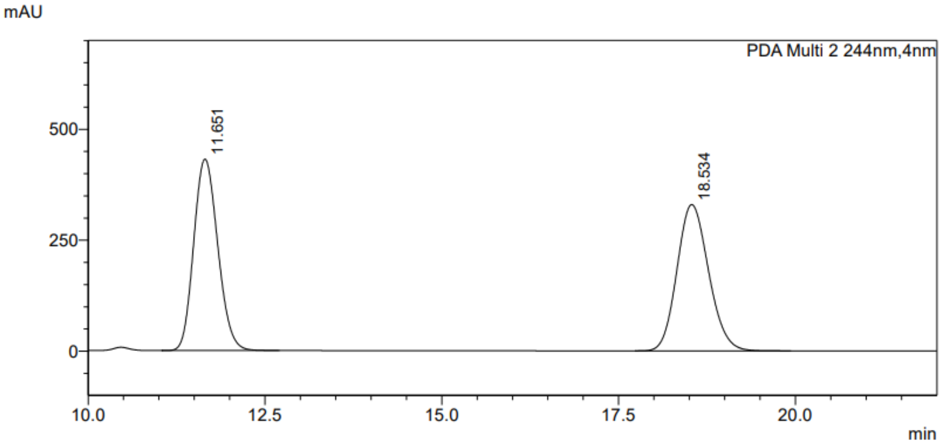

PDA Ch2 244nm

Peak#	Ret. Time	Area	Area%
1	11.651	10203904	49.881
2	18.534	10252698	50.119
Total		20456602	100.000

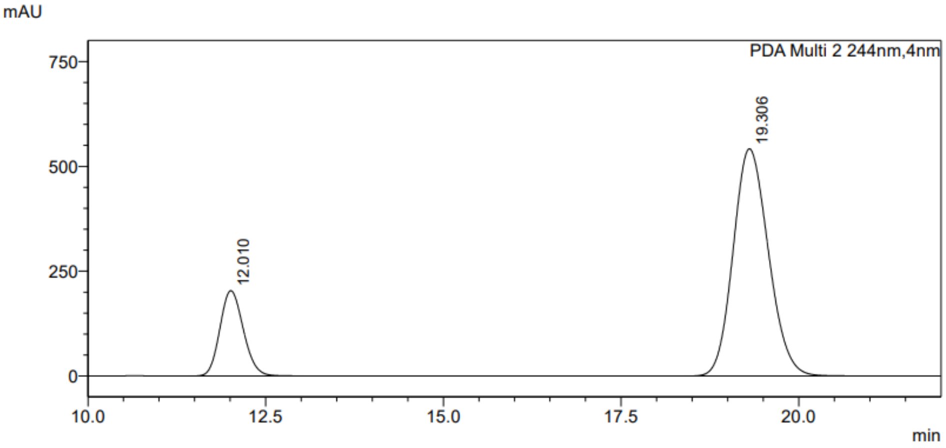

PDA Ch2 244nm

Peak#	Ret. Time	Area	Area%
1	12.010	4661684	19.937
2	19.306	18720494	80.063
Total		23382178	100.000

(2*S*,3*R*)-Methyl-4-(2-benzoyl-3-hydroxy-5-methylhexyl)benzoate (3n)

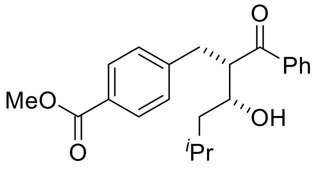

(2*S*,3*R*)-3n

CHIRALPAK IC, 95:5 *n*-hexane:isopropanol, flow rate 1.0 mL/min, 244 nm, 30 °C. t_R **(2*R*,3*S*)-3n** 14.4 min, t_R **(2*S*,3*R*)-3n** 18.3 min, 84:16 *e.r.*

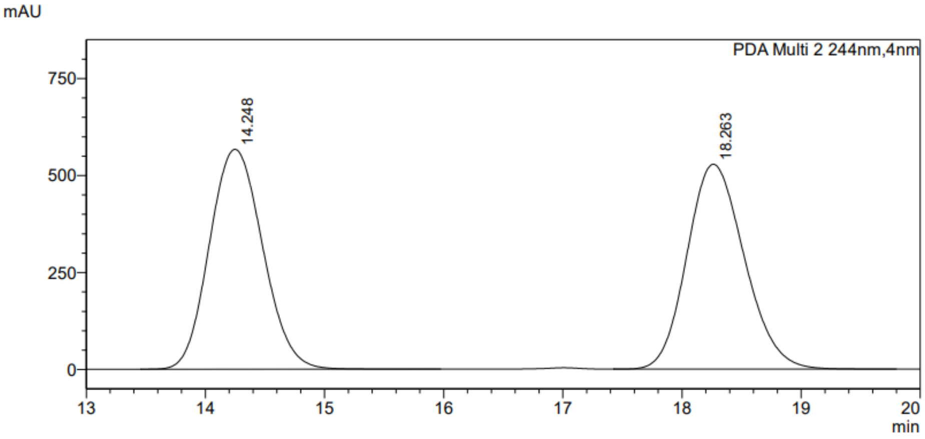

PDA Ch2 244nm

Peak#	Ret. Time	Area	Area%
1	14.248	17632944	49.898
2	18.263	17705078	50.102
Total		35338022	100.000

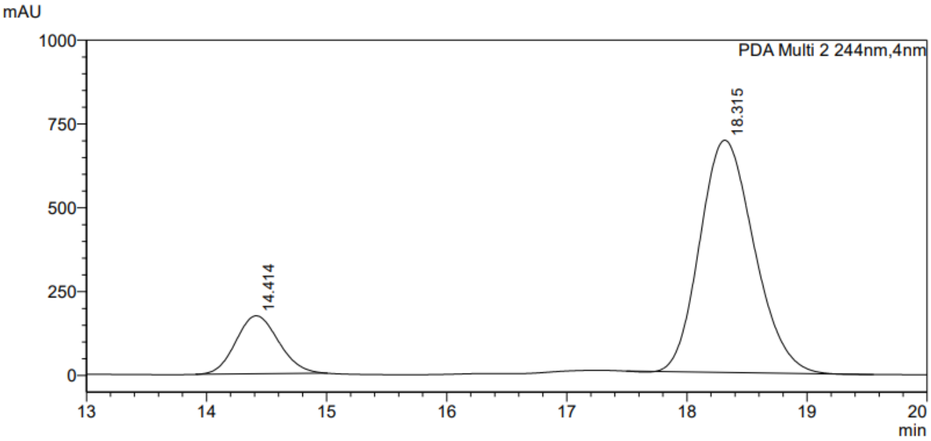

PDA Ch2 244nm

Peak#	Ret. Time	Area	Area%
1	14.414	4293887	16.459
2	18.315	21794877	83.541
Total		26088764	100.000

(2*S*,3*R*)-2-Benzyl-1-cyclopropyl-3-hydroxy-5-methylhexan-1-one (3q)

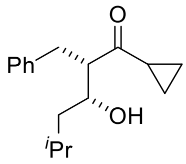

(2*S*,3*R*)-3q

CHIRALPAK IC**,** 95:5 *n*-hexane:isopropanol, flow rate 1.0 mL/min, 210 nm, 30 °C. t_R **(2*R*,3*S*)-3q** 7.4 min, t_R **(2*S*,3*R*)-3q** 9.0 min, 80:20 *e.r.*

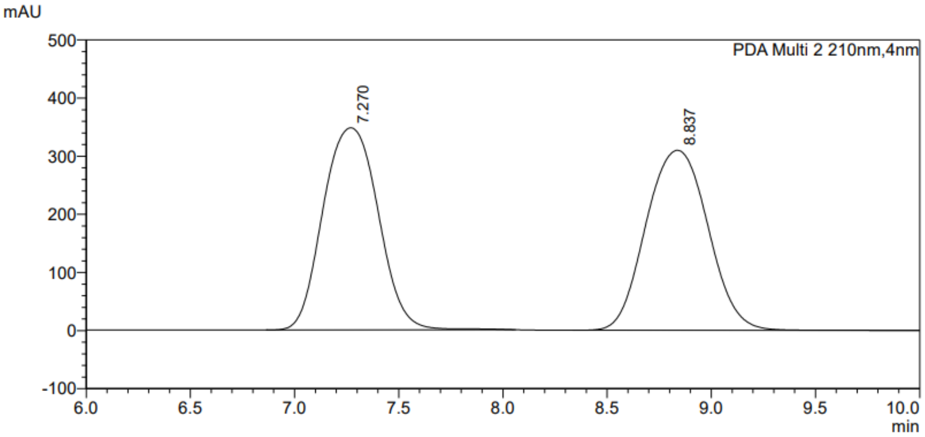

PDA Ch2 210nm

Peak#	Ret. Time	Area	Area%
1	7.270	6428394	50.034
2	8.837	6419665	49.966
Total		12848059	100.000

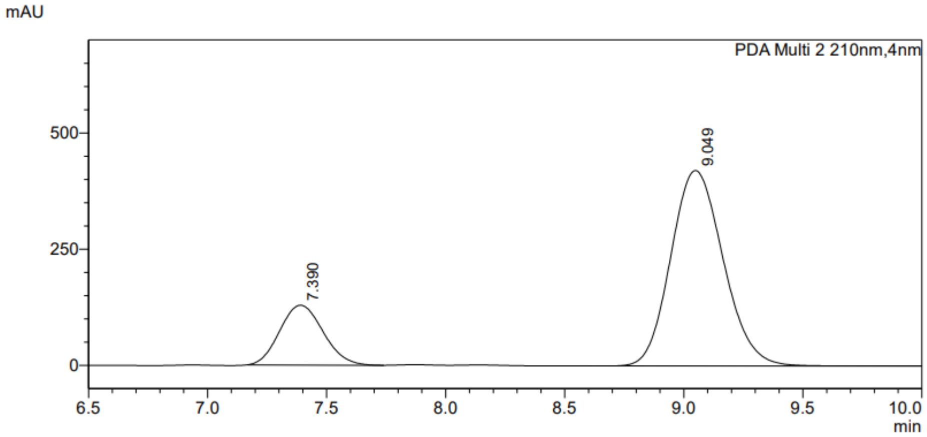

PDA Ch2 210nm

Peak#	Ret. Time	Area	Area%
1	7.390	1630536	20.213
2	9.049	6436382	79.787
Total		8066918	100.000

(2*S*,3*R*)-4-Benzyl-5-hydroxy-2,2,7-trimethyloctan-3-one (3r)

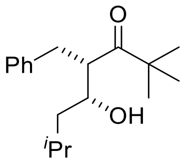

(2*S*,3*R*)-3r

CHIRALPAK IB**,** 95:5 *n*-hexane:isopropanol, flow rate 1.0 mL/min, 251 nm, 30 °C. t_R **(2*S*,3*R*)-3r** 8.3 min, t_R **(2*R*,3*S*)-3r** 10.7 min, 72:28 *e.r.*

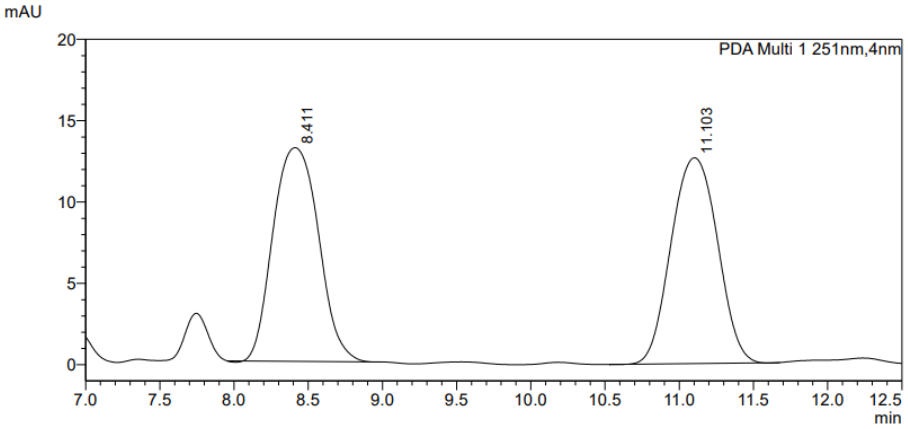

PDA Ch1 251nm

Peak#	Ret. Time	Area	Area%
1	8.411	280789	50.438
2	11.103	275917	49.562
Total		556706	100.000

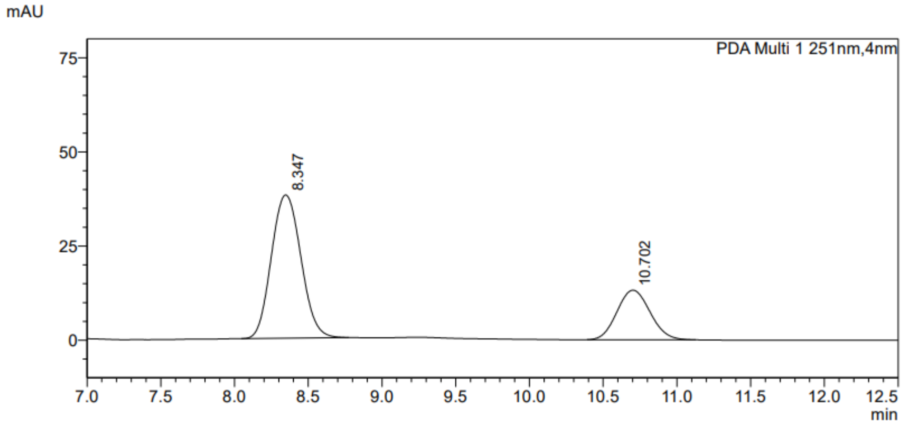

PDA Ch1 251nm

Peak#	Ret. Time	Area	Area%
1	8.347	530933	72.102
2	10.702	205427	27.898
Total		736360	100.000

(2*R*,3*R*)-2-Ethyl-3-hydroxy-1-(2,6,6-trimethylcyclohex-1-en-1-yl)octan-1-one (3s)

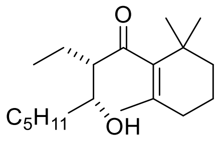

(2*R*,3*R*)-3s

CHIRALPAK IG, 99:1 *n*-hexane:isopropanol, flow rate 1.0 mL/min, 244 nm, 30 °C. t_R **(2*S*,3*S*)-3s** 17.2 min, t_R **(2*R*,3*R*)-3s** 18.5 min, 80:20 *e.r.*

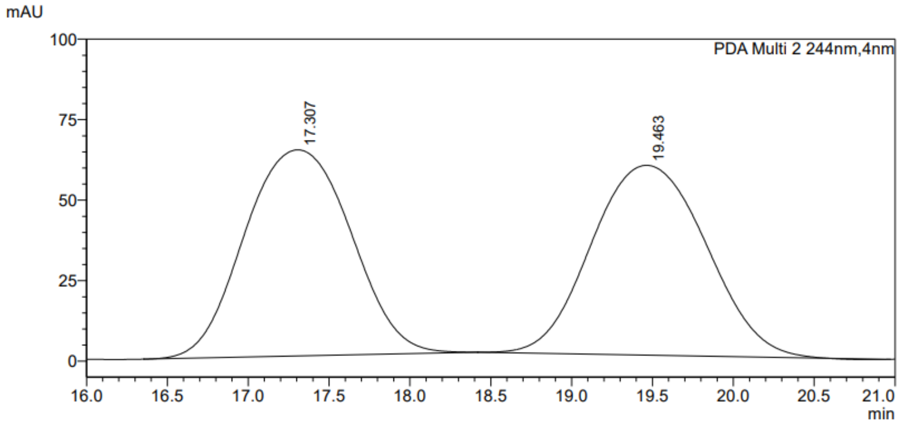

PDA Ch2 244nm

Peak#	Ret. Time	Area	Area%
1	17.307	2935266	50.212
2	19.463	2910452	49.788
Total		5845718	100.000

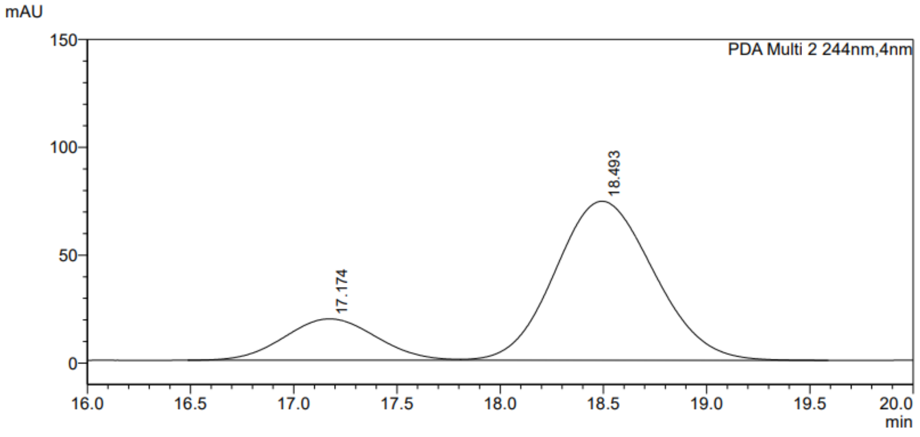

PDA Ch2 244nm

Peak#	Ret. Time	Area	Area%
1	17.174	595291	19.510
2	18.493	2455975	80.490
Total		3051267	100.000

Appendix III: Single Crystal X-ray Diffraction Data

(3S,8R,9S,10R,13S,14S,17S)-17-Acetyl-17-((S)-1-hydroxy-3-methylbutyl)-10,13-dimethyl-2,3,4,7,8,9,10,11,12,13,14,15,16,17-tetradecahydro-1H-cyclopenta[a]-phenanthren-3-yl acetate (3t)

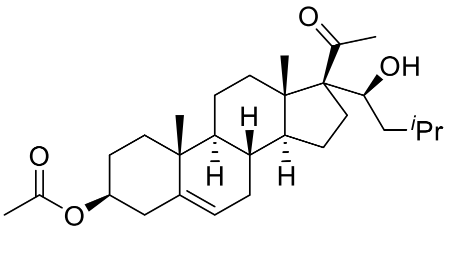

3t

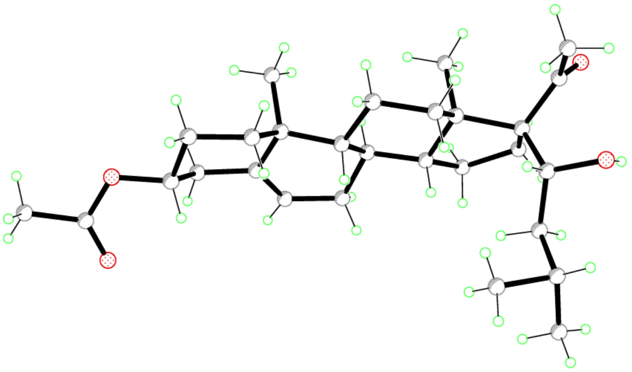

R₁=4.76%

Experimental. Single clear colourless block-shaped crystals of **3t** (CCDC 2532648) recrystallised from diethyl ether by slow cooling. A suitable crystal with dimensions 0.25 × 0.20 × 0.18 mm^3 was selected and mounted on a mitegen tip in Paratone oil. on a XtaLAB Synergy R, HyPix-Arc 100 diffractometer. The crystal was kept at a steady T = 100.01(10) K during data collection. The structure was solved with the ShelXS (Sheldrick, 2008) solution program using direct methods and by using Olex2 1.5-beta (Dolomanov et al., 2009) as the graphical interface. The model was refined with ShelXL 2018/3 (Sheldrick, 2015) using full matrix least squares minimisation on F^2.

Crystal Data. $C_{58.18}H_{95.27}O_{9.46}$, M_r = 946.04, monoclinic, $P2_1$ (No. 4), a = 18.72992(14) Å, b = 7.65685(4) Å, c = 20.61406(15) Å, b = 116.1714(9)°, a = g = 90°, V = 2653.22(4) Å^3, T = 100.01(10) K, Z = 2, Z' = 1, m(Cu K_a) = 0.616, 102478 reflections measured, 11011 unique (R_{int} = 0.0327) which were used in all calculations. The final wR_2 was 0.1366 (all data) and R_1 was 0.0476 (I≥2 s(I)).

_refine_special_details: When both molecules had been modelled it was obvious from both displacement ellipsoids and residual peaks that a disordered mix of solvent was present. This was identified as a mix of diethyl ether (from crystallization) and water (present from prior work-up), modelled with similarity restraints.

**Compound**	**3t** (CCDC 2532648)
Formula	$C_{58.18}H_{95.27}O_{9.46}$
$D_{calc.}$/ g cm^{-3}	1.184
m/mm^{-1}	0.616
Formula Weight	946.04
Colour	clear colourless
Shape	block-shaped
Size/mm^3	0.25×0.20×0.18
T/K	100.01(10)
Crystal System	monoclinic
Flack Parameter	0.02(3)
Hooft Parameter	0.03(2)
Space Group	$P2_1$
a/Å	18.72992(14)
b/Å	7.65685(4)
c/Å	20.61406(15)
a/°	90
b/°	116.1714(9)
g/°	90
V/Å^3	2653.22(4)
Z	2
Z'	1
Wavelength/Å	1.54184
Radiation type	Cu K_a
Q_{min}/°	2.388
Q_{max}/°	77.250
Measured Refl's.	102478
Indep't Refl's	11011
Refl's I≥2 *s*(I)	10705
R_{int}	0.0327
Parameters	855
Restraints	374
Largest Peak	0.601
Deepest Hole	-0.302
GooF	1.059
wR_2 (all data)	0.1366
wR_2	0.1353
R_1 (all data)	0.0486
R_1	0.0476

(2*S*, 3*R*)-2-Benzyl-3-hydroxy-5-methyl-1-phenylhexan-1-one (3a)

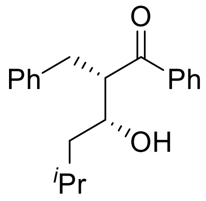

(2*S*, 3*R*)-3a

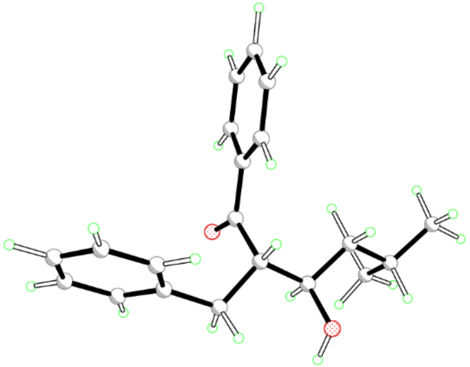

R_1=0.67%

Experimental. Single colourless block-shaped crystals of **(2*S*, 3*R*)-3a** (CCDC 2532647). Slow cooling from hot ether. A suitable crystal with dimensions 0.18 × 0.11 × 0.08 mm^3 was selected and mounted on a MITIGEN holder in Paratone oil. on a XtaLAB Synergy R, HyPix-Arc 100 diffractometer. The crystal was kept at a steady T = 100.00(10) K during data collection. The structure was solved with the ShelXS (Sheldrick, 2008) solution program using direct methods and by using Olex2 1.5-beta (Dolomanov et al., 2009) as the graphical interface. The model was refined with olex2.refine 1.5-beta (Bourhis et al., 2015) using full matrix least squares minimisation on F^2.

Crystal Data. $C_{20}H_{24}O_2$, M_r = 296.412, orthorhombic, $P2_12_12_1$ (No. 19), a = 7.83692(3) Å, b = 12.51912(4) Å, c = 16.83910(5) Å, $a = b = g$ = 90°, V = 1652.106(9) Å^3, T = 100.00(10) K, Z = 4, Z' = 1, m(Cu K_a) = 0.587, 106605 reflections measured, 3485 unique (R_{int} = 0.0206) which were used in all calculations. The final wR_2 was 0.0161 (all data) and R_1 was 0.0067 (I≥2 s(I)).

_olex2_refine_details: Refinement using NoSpherA2, an implementation of NOn-SPHERical Atom-form-factors in Olex2.Please cite:F. Kleemiss et al. Chem. Sci. DOI 10.1039/D0SC05526C - 2021NoSpherA2 implementation of HAR makes use oftailor-made aspherical atomic form factors calculatedon-the-fly from a Hirshfeld-partitioned electron density (ED) - not fromspherical-atom form factors.The ED is calculated from a gaussian basis set single determinant SCFwavefunction - either Hartree-Fock or DFT using selected funtionals- for a fragment of the crystal.This fragment can be embedded in an electrostatic crystal field by employing cluster chargesor modelled using implicit solvation models, depending on the software used.The following options were used: SOFTWARE: ORCA 5.0 PARTITIONING: NoSpherA2 INT ACCURACY: Normal METHOD: r2SCAN BASIS SET: cc-pVTZ CHARGE: 0 MULTIPLICITY: 1 DATE: 2025-03-25_09-55-17

**Compound**	**(2*S*, 3*R*)-3a** (CCDC 2532647)
Formula	$C_{20}H_{24}O_2$
$D_{calc.}$/ g cm^{-3}	1.192
m/mm^{-1}	0.587
Formula Weight	296.412
Colour	colourless
Shape	block-shaped
Size/mm^3	0.18×0.11×0.08
T/K	100.00(10)
Crystal System	orthorhombic
Flack Parameter	0.011(13)
Hooft Parameter	0.011(13)
Space Group	$P2_12_12_1$
a/Å	7.83692(3)
b/Å	12.51912(4)
c/Å	16.83910(5)
a/°	90
b/°	90
g/°	90
V/Å^3	1652.106(9)
Z	4
Z'	1
Wavelength/Å	1.54184
Radiation type	Cu K_a
Q_{min}/°	4.40
Q_{max}/°	77.29
Measured Refl's.	106605
Indep't Refl's	3485
Refl's I≥2 *s*(I)	3473
R_{int}	0.0206
Parameters	415
Restraints	0
Largest Peak	0.0393
Deepest Hole	-0.0604
GooF	1.7886
wR_2 (all data)	0.0161
wR_2	0.0161
R_1 (all data)	0.0068
R_1	0.0067
